# Barriers to cancer screening uptake and approaches to overcome them: a systematic literature review

**DOI:** 10.3389/fonc.2025.1575820

**Published:** 2025-08-06

**Authors:** R. Aguiar-Ibáñez, YPV. Mbous, Sugandh Sharma, R. Chakali, E. Chawla

**Affiliations:** ^1^ Merck Canada Inc., Kirkland, QC, Canada; ^2^ Merck & Co., Inc., Rahway, NJ, United States; ^3^ Parexel International, Chandigarh, India; ^4^ Parexel International, Bengaluru, India; ^5^ Parexel International, Mohali, India

**Keywords:** barriers, cancer screening, screening uptake, facilitators, screening intervention, public health, preventative healthcare

## Abstract

**Introduction:**

Cancer screening programs play a crucial role in early detection, improving survival rates and reducing the burden of advanced cancer. However, uptake remains inconsistent due to multifaceted barriers. This systematic review aimed to identify factors that impact cancer screening uptake across multiple tumor types and interventions to overcome barriers to cancer screening uptake.

**Methods:**

A systematic literature review (SLR) was conducted using Embase^®^ and MEDLINE^®^ (May 2012 to May 2022) to identify observational studies that reported factors associated with screening uptake in adults, worldwide, with no tumor-specific restrictions. Records identified were screened by two independent reviewers. Included studies were data extracted by two reviewers and the results were reported narratively, focusing on identifying factors that acted as barriers or facilitators to cancer screening uptake, along with potential interventions to improve screening uptake.

**Results:**

Overall, 811 studies were identified from the SLR that reported factors influencing the uptake of screening programs, with 658 studies covering screening programs for breast, cervical, lung, colorectal, gastric and prostate cancers. Barriers to cancer screening included: being unmarried, experiencing higher deprivation, lower socioeconomic status and rural living conditions. Facilitators to cancer screening included: older age, poor perception of health, previous cancer history, family history of cancer, previous cancer screening history, having knowledge of the disease, positive attitudes to screening, perceived cancer risk, higher education level, having children, higher income, higher socioeconomic status, having health insurance, urban residence, having access to care, and recommendations for screening by primary care physicians. Mixed findings were identified for race and ethnicity, employment and smoking status. Targeted educational programs were the most suggested strategy to overcome barriers to cancer screening uptake.

**Conclusion:**

Barriers to cancer screening across multiple tumor types are complex, spanning demographic and patient-level factors, social and economic factors, provider and community challenges, and access to health care. While certain barriers are shared across tumor types, others are unique, reflecting the specific requirements of screening for different tumors. Addressing these barriers requires multi-level strategies that integrate both universal and cancer-specific approaches. Targeted interventions and supportive policies can increase screening participation, facilitate earlier cancer diagnosis, and reduce disparities in cancer outcomes.

## 1 Introduction

Cancer is a major public health issue, with over 20 million estimated cases and 9.7 million cancer-related deaths in 2022 (1). It represents a growing problem, with the number of annual cases expected to rise to 35 million by 2050 (1). One of the potential strategies to minimize the growing burden associated with cancer is to diagnose and detect cancer during the early stages (2, 3). Evidence-based cancer screening programs have great potential to improve cancer outcomes, and when organized effectively and quality-assured, they can detect pre-cancer or early-stage cancer in asymptomatic individuals, facilitating timely diagnosis and early access to treatment (4). Cancer screening programs have been shown to deliver improved long-term survival outcomes and reduced cancer-related mortality, along with substantial reductions in economic burden (5–7).

Despite the benefits, uptake of cancer screening has been mixed in recent years. A 2019 report by the American Cancer Society (ACS) reported that colorectal cancer (CRC) screening (endoscopy) rates increased steadily from 46.8% in 2005, to 60.3% in 2015. However, over the same period screening rates for cervical cancer (pap test) declined (2005: 85.4%, 2015: 81.6%), breast cancer screening rates (mammography) remained stable (2005: 51.2%, 2015: 50.2%), and uptake of lung cancer screening (low-dose computerized tomography [CT]) was extremely sparse (2005: not available, 2015: 3.9%) (8). In the 2024 State of Lung Cancer report released by the American Lung Association, lung cancer screening is recognized as having the potential to save hundreds of thousands of lives and generate substantial economic savings, yet despite this, screening rates remain low, with only 16% of eligible individuals screened nationwide. Disparities are evident, with states in the United States (U.S.) like Rhode Island achieving a screening rate of 28.6%, while others, like Wyoming, lag at 8.6%. However, policy changes, such as the expanded eligibility criteria by the United States Preventative Services Task Force (USPSTF) and the removal of Medicare registry requirements for reimbursement in March 2021, have altered access and reporting trends (9). These varied trends suggest that several complex and intersecting factors influence cancer screening uptake across the different tumor types.

The implementation of effective cancer screening programs requires substantial resources at the health system level across several broad categories, including funding, infrastructure and human resources (10). Furthermore, the successful rollout of these cancer screening programs is shaped by various factors across the healthcare system level, provider level, and patient levels (11–14). At the healthcare system level, policy decisions, funding availability, and infrastructure adequacy play a crucial role in determining accessibility and reach to screening programs (13, 14). For instance, well-defined policies and dedicated funding streams can enable broad implementation, while limited infrastructure can lead to restricted access to underserved populations (13, 14). At the provider level, factors such as workforce training and capacity are instrumental (11–13). Adequate provider training ensures the accurate administration of screening protocols, while insufficient staffing can limit screening program effectiveness and reach (11–13). On the community or patient level, accessibility, awareness, and cultural factors are critical determinants of screening uptake (11–14). For example, low health literacy or culturally specific health programs can act as barriers, while tailored outreach programs might serve as facilitators. Moreover, the impact of these factors on screening programs may shift, depending on the target population or geographic context, emphasizing the need to understand how they interact (11–14). Understanding these multifaceted influences is essential when designing effective interventions that address barriers at various levels of the healthcare system. By enhancing cancer screening uptake, such interventions can ultimately reduce cancer-related mortality, alleviate the humanistic burden and lessen the financial impact associated with cancer diagnosed at later stages (15, 16). Moreover, recognizing the factors impacting cancer screening uptake is crucial for developing effective implementation strategies.

Reviews conducted to date have largely focused on perceived barriers to screening within specific sub-populations e.g., based on age, disability, or sexual orientation, geographical location, or single tumor type (17–20). Commonly cited barriers to screening uptake include social determinants, perceived bias and discrimination against minoritized populations (18), mistrust of health care professionals among minoritized populations (21, 22), low levels of health literacy and education (22–24), lack of access to health insurance and/or healthcare resources (25) and other socioeconomic barriers (20, 25). However, a comprehensive review of barriers across multiple tumor types, geographies, and populations is lacking.

This systematic literature review aimed to identify factors that act as barriers to cancer screening uptake across multiple tumor types, geographies, population categories, as well as the proposed interventions to improve cancer screening rates. While the initial focus of this study was to identify barriers that could be addressed to improve the screening uptake, some of these factors may act as barriers, others as facilitators for cancer screening, and some as both, barriers and facilitators, depending on the context. By analyzing factors affecting screening uptake across multiple tumor types, this review seeks to identify common barriers or facilitators to cancer screening uptake, providing insights that can guide the optimal use of healthcare resources to promote early cancer detection and improve clinical outcomes.

## 2 Methods

### 2.1 Data sources and search strategy

A systematic literature review (SLR) was conducted following the methodology listed by the Cochrane Handbook and the Centre for Reviews and Dissemination (26, 27). The reporting of this SLR followed the guidelines of the Preferred Reporting Items for Systematic Reviews and Meta-Analysis (PRISMA) (28). The study was conducted following the development of a protocol, which identified the objectives, the pre-defined PICOTS criteria, the methodology and reporting of results. This pre-defined protocol and remained unchanged throughout the review process, and adherence to it ensured transparency, reproducibility, and minimized bias in the conducted review process.

There were no restrictions on the tumor types, language or geographical location of the included studies. Only English search terms were employed in the search. Eligibility criteria focused primarily on studies that reported factors impacting screening uptake and associated interventions that could help mitigate the existing factors in general or high-risk population. MEDLINE^®^ and Embase^®^, Cochrane Database of Systematic Reviews (CDSR), Cochrane Controlled Register of Trials (CENTRAL) and PubMed (solely to capture in-process citations) were searched to retrieve evidence published between May 2012 and May 2022. Grey literature sources included the Open Grey. Additional searches of conference proceedings from the American Society of Clinical Oncology (ASCO), the European Society for Medical Oncology (ESMO), ESMO Breast Cancer, the American Association for Cancer Research (AACR) and the International Society for Pharmacoeconomics and Outcomes Research (ISPOR) (2020–2022, inclusive) were searched to capture any studies not yet published in full-text articles, or abstracts reporting supplementary follow-up results associated with previously published studies. Bibliographic searching of published literature reviews was also conducted.

The search strategy is provided in the **Supplementary Appendix S1**.

### 2.2 Eligibility criteria for study selection

Included were observational studies for adults (≥18 years old) eligible to undergo cancer screening. There were no restrictions on interventions or comparators. Cancer screening included different outcome definitions, such as: general cancer screening, any cancer screening participation or adherence to cancer screening, as well as cancer-specific screening tests or methods, for example, mammography for breast cancer, pap smear testing for cervical cancer, or colonoscopy for CRC. Key outcomes of interest were factors associated with adherence to available screening guidelines or programs (e.g., patient profile, familial history or biomarker assessment). Other factors such as barriers and facilitators associated with patient characteristics and beliefs (e.g., predictors of low uptake vs high uptake such as age, ethnicity, tumor type, familial history, education, patients beliefs or socio-cultural factors associated with low uptake of screening opportunities, symptom recognition, health literacy among patients, median/mean time from diagnosis to treatment), factors associated with screening access (e.g., availability and funding for diagnostic tools or screening programs, literacy and or symptom awareness among physicians, challenges attending screening), perspectives from scientific leaders on screening guidelines, and interventions or approaches that may help overcome screening barriers were included. The timeframe of interest ranged from 2012 to 2022. The detailed information on the predefined Population, Interventions, Comparisons, Outcomes, Time, and Study design (PICOTS criteria) is included in the **Supplementary Appendix S2**.

### 2.3 Study search, selection, and abstraction

The study selection (i.e., the screening of titles and abstracts followed by full text screening), was performed by two independent reviewers to identify all relevant studies. A third independent reviewer resolved any discrepancies. Data from included studies were extracted into a pre-defined extraction grid, ensuring that data was extracted uniformly and was comparable across studies. Data extraction was again conducted by two reviewers, and subsequently checked and reconciled by an independent third reviewer.

### 2.4 Risk of bias, strength, and certainty of evidence

Quality assessment of included observational studies, in particular, cohort and cross-sectional studies, was conducted using the Newcastle-Ottawa scale (NOS) (29). The NOS scale consists of three domains: the selection of the study groups, the comparability of the groups, and the ascertainment of the exposure or outcome of interest. The total maximum score is 9, with three threshold ranges for: high quality (scores from 7-9), medium quality (scores from 4-6), and low quality (scores from 0-3) (29).

### 2.5 Data analysis

This study employed a narrative SLR approach, where no statistical pooling of results was conducted. Only studies reporting statistically significant odd ratios (ORs) conveying the degree and direction of association between factors and cancer screening are discussed in the results section. For reporting purposes, we focused on visualizing through figures the identified ORs (whenever reported) across included studies using logarithmic scale (log(OR)), categorized by tumor types and indicating whether each factor was identified as a barrier or facilitator to screening uptake. This methodology allowed for a comprehensive overview of the landscape of factors influencing cancer screening uptake. The factors identified in the SLR were to be categorized into six main domains, namely: demographics, economics, patient access, patient behavior, primary care physician (PCP) access, and social barriers.

Factors identified as barriers were defined as factors with an OR<1 (log(OR)<0), which indicated a lower likelihood of screening uptake for the comparison group vs the reference group. Similarly, facilitating factors were defined as factors with an OR>1 (log(OR)>0), which were indicative of higher likelihood of screening uptake for the comparison group versus the reference group. Only those factors that demonstrated statistically significant associations (p < 0.05) with screening uptake were included. When reported, ORs based on multivariate analyses were preferred to those from univariate analyses. Univariate analyses were presented if they were statistically significant and if multivariate analyses were not provided. Statistical significance of the reported ORs was confirmed based on *p*-values, or 95% CI in cases where *p*-values were not available.

Figures representing the log(ORs) for the selected factors were then developed for factors reported in which in at least one of the tumor types there were 10 studies reporting on a specific factor, and therefore that factor was represented in the figure across all the priority tumor types. These figures were to be descriptive and to be used as a visualization aid, and no statistical analyses to pool the data were conducted. The aim was to provide an understanding on the number of studies that identified the respective factor as a barrier or facilitator, and the number of times the factor was identified, across all relevant studies with different strata used to categorize each factor. In factors with varying categorizations across studies, designated reference groups (as reported in the studies) were used as the common reference group for that factor among all relevant studies. For example, for the factor ‘age’, reference groups (e.g. <50 years for breast cancer, or <30 years for cervical cancer) were established based on the age groups/categories that served as a reference across the majority of studies focusing on the respective populations being screened for cancer.

Furthermore, interventions aimed to overcome barriers mentioned in the included studies were also identified and summarized to provide valuable insights into potential strategies for improving cancer screening uptake.

## 3 Results

### 3.1 Included studies

**Figure 1:**
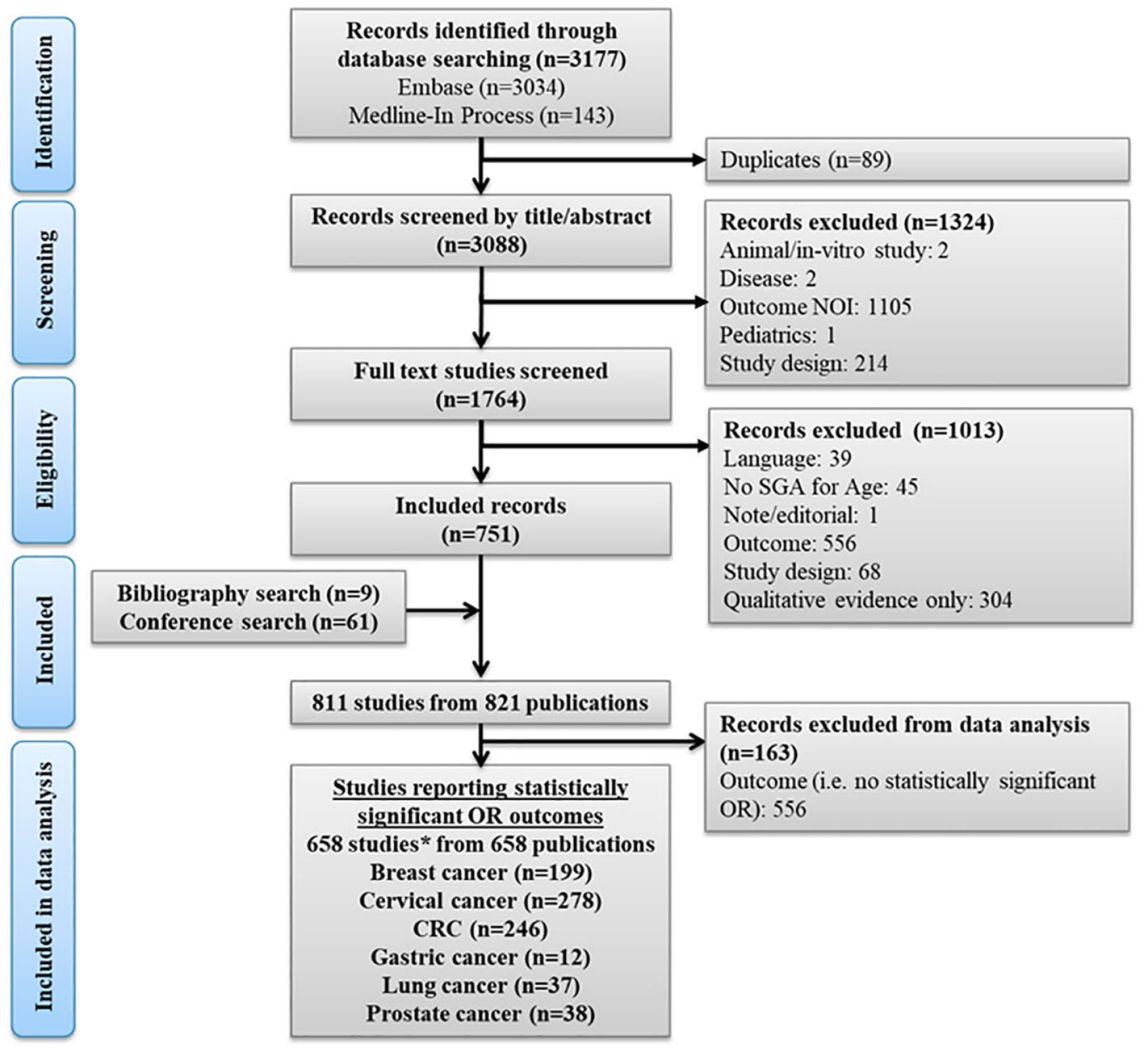
PRISMA flow diagram. *The numbers of studies per tumor type were not mutually exclusive as some studies presented results for screening programs across more than one tumor type. CRC, Colorectal cancer; log, logarithm; NOI, Not of interest; OR, Odds ratio; PRISMA, Preferred Reporting Items for Systematic Reviews and Meta-Analyses; SGA, Subgroup analysis.

A total of 811 studies from the SLR were initially included that reported factors which had statistically significant or non-significant associations with the uptake of screening programs (based on reported ORs), as well as other outcome measures such as hazard ratios. Studies on screening for breast, cervical, colorectal, lung, gastric, and prostate cancer were prioritized for reporting, as these tumor types have well established screening programs (30). Overall, 658 unique studies were included with the final number of included studies per tumor type which reported statistically significant ORs for factors influencing screening uptake being: 199 for breast cancer, 278 for cervical cancer, 246 for CRC, 37 for lung cancer, 12 for gastric cancer and 38 for prostate cancer (**Figure 1**). As studies may report on screening for multiple tumor types, the number of included studies per tumor type are not mutually exclusive. Full details of the study characteristics across the included studies are presented in the **Supplementary Appendix S5**.

A diverse range of data was identified across the included studies. To allow for identification of trends across the included data points, evidence was reported according to six main factor categories associated with screening uptake, including: demographics, economics, patient access, patient behaviour, PCP access and social factors (as detailed in section 2). The outcomes considered for inclusion in each of these categories, and their definitions, are presented in the **Supplementary Appendix S3**. The number of studies reporting factors across these 6 categories, according to the tumor types screened across the included studies, were: 384 for demographic factors, 240 for economic factors, 260 for social factors, 118 for patient behavior-related factors, 256 for patient access-related factors, and 104 for PCP access-related factors (see **Supplementary Appendix S4**).

**Figure 2:**
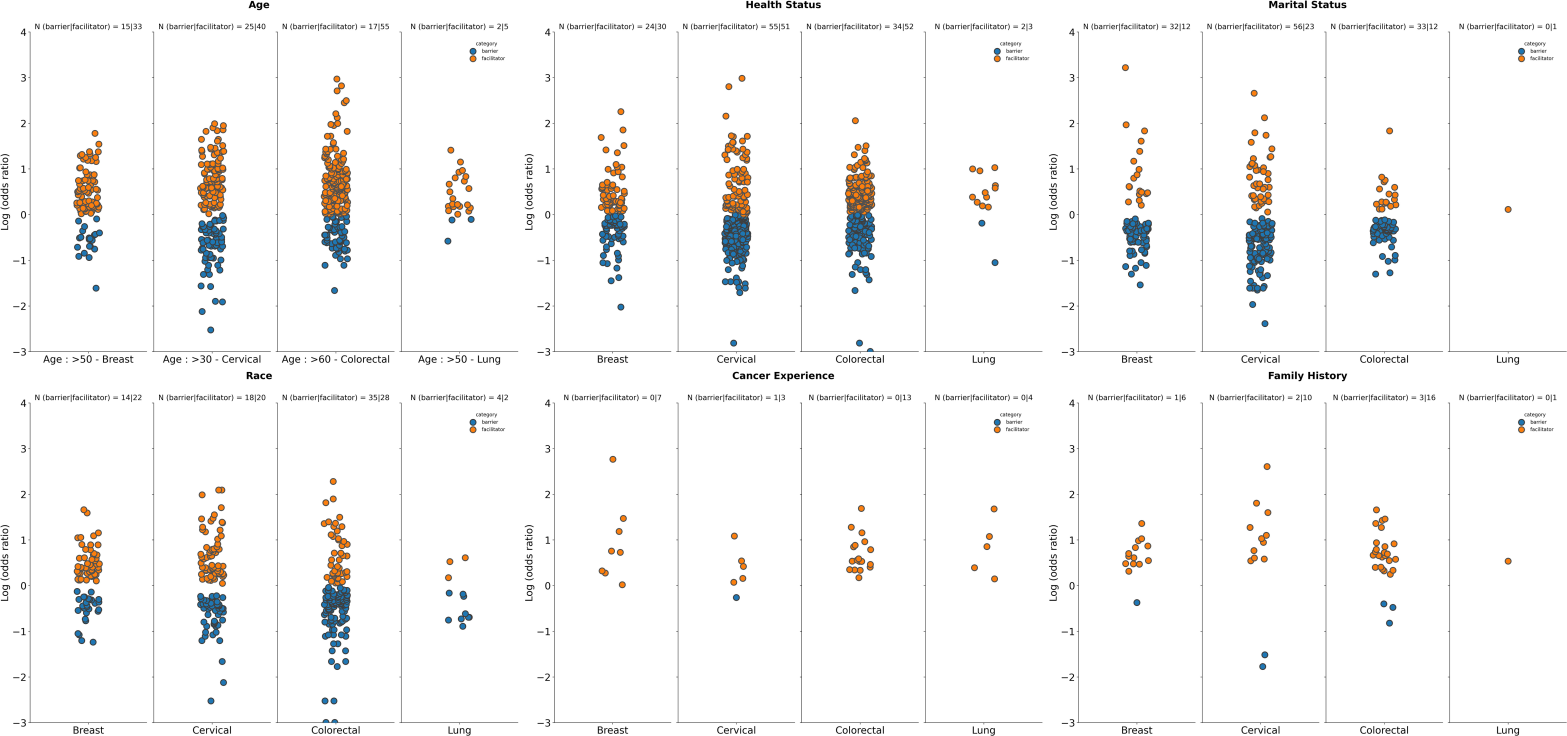
Summary of logarithm of odds ratios according to tumor type for selected demographic characteristics. Age: Datapoints represent log(ORs) for groups aged ≥50 years (vs. <50 years as reference group in studies on breast and lung cancer screening programs), ≥30 years (vs. < 30 years as reference groups for studies on screening for cervical cancer), ≥60 years (vs. <60 years as reference group in studies on screening for CRC); Health status: Datapoints represent log(ORs) for groups with perception of health rated as “poor”, or “fair” (vs. ratings of “good”, “very good”, and “normal” as reference group across included studies); Marital status: Datapoints represents log(ORs) for “single”, “divorced”, “widowed” “or “separated” groups (vs. those who were “married” or “partnered” as reference groups); Race: Datapoints represent log(OR) for non-white race groups (vs. white race groups as reference group); Cancer experience: Datapoints represents log(ORs) for groups with any previous cancer history (vs. those with no cancer history as a reference group across included studies); Family history: Datapoints represents log(ORs) for groups with family history of cancer (vs. those with no history of cancer as a reference group). The number of studies for which a specific factor acted as a barrier, or a facilitator is shown as “N” atop each panel. A single study may be categorized as both a barrier and a facilitator if different strata within it showed opposing associations with screening uptake for the same factor. Facilitator (orange): a factor with a log(OR) >0, indicating a higher screening uptake for the comparison group vs the reference group. Barrier (blue): a factor with a log(OR) <0, indicating a lower screening uptake for the comparison group vs the reference group.

**Figure 3:**
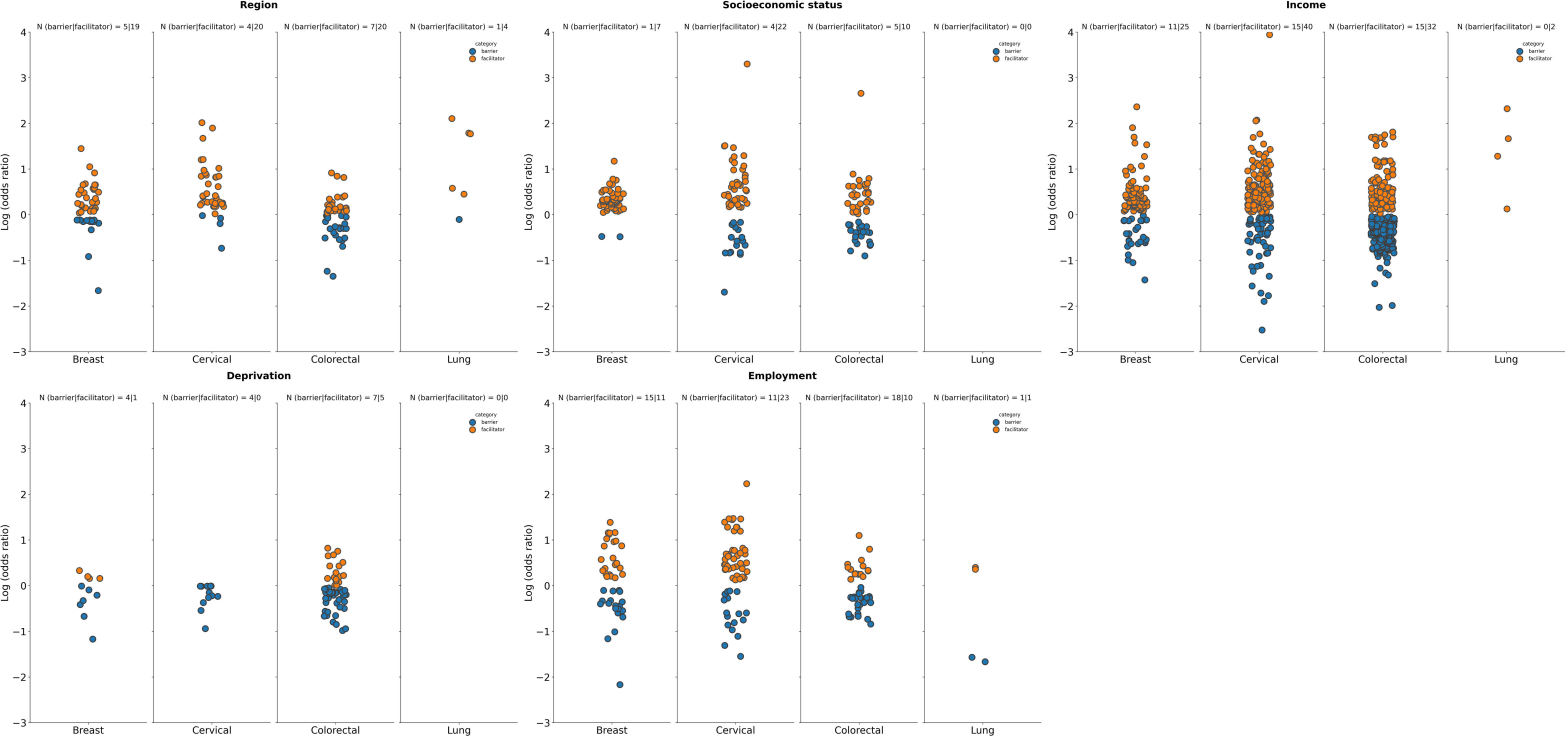
Summary of logarithm of odds ratios according to tumor type for selected economic factors. Deprivation: Datapoints represent log(ORs) for the most deprived groups (vs. least deprived as reference) Deprivation was defined via the Area/European deprivation index; Region: Datapoints represent log(ORs) for urban or suburban dwelling groups (vs. “inaccessible areas”, “rural”, or “non-metropolitan” as reference); Socioeconomic status: Datapoints represent log(ORs) for richer or wealthier groups (vs. “poorest “, “poor, or “lower class” as reference); Income: Datapoints represent log(ORs) for higher income groups (vs. lower income groups as reference); Employment: Datapoints represent log(ORs) for employed individuals/groups (vs. “unemployed”, “retired”, “homemaker”, “housewife”, or “student” groups as reference). Employed included “self-employed”, “part-time and full-time employment”, “contracted”. The number of studies for which a specific factor acted as a barrier (in blue) or a facilitator (in orange) is shown as “N” atop each panel. A single study may be categorized as both a barrier and a facilitator if different strata within it showed opposing associations with screening uptake for the same factor. Facilitator (orange): a factor with a log(OR) >0, indicating a higher screening uptake for the comparison group vs the reference group. Barrier (blue): a factor with a log(OR) <0, indicating a lower screening uptake for the comparison group vs the reference group.

**Figure 4:**
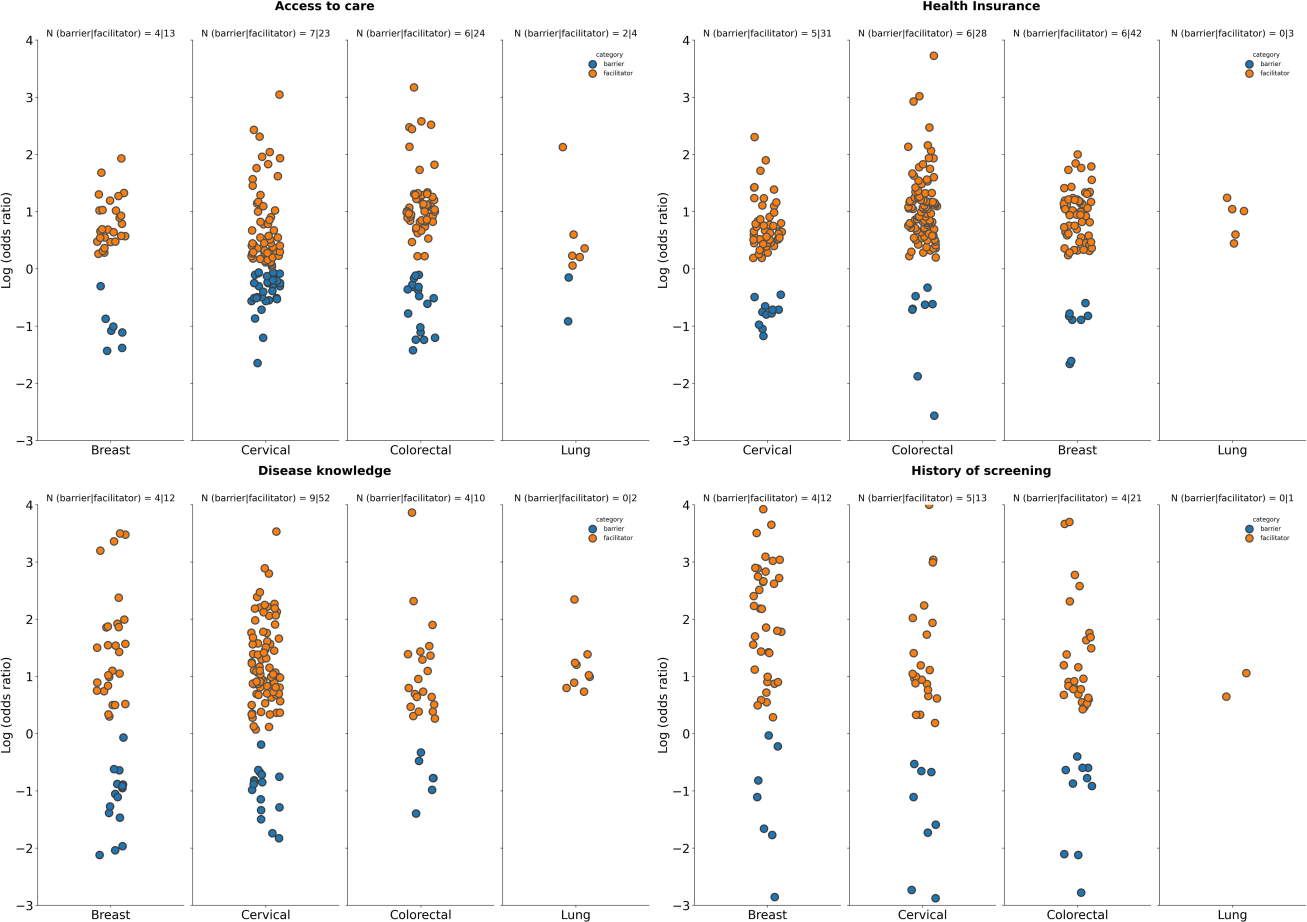
Summary of logarithm of odds ratios according to tumor type for selected patient access-related factors. Access to care: Datapoints represent log(ORs) for groups with access to care or a PCP (vs. those with “no access to care”, “no PCP”, or “health access hardship” as reference); Health insurance coverage: Datapoints represent log(ORs) for groups with any form of health insurance coverage (vs. uninsured groups as reference); Disease knowledge: Datapoints represent log(ORs) for groups with “high”, “good’, or “sufficient” knowledge about the disease (vs. groups with “low understanding”, “not knowledgeable”, “poor knowledge”, or “insufficient” knowledge as reference); History of screening: Datapoints represent log(ORs) for groups with a history of cancer screening (vs. those with no history of screening as reference). The number of studies for which a specific factor acted as a barrier (in blue), or a facilitator (in orange) is shown as “N” atop each panel. A single study may be categorized as both a barrier and a facilitator if different strata within it showed opposing associations with screening uptake for the same factor. Facilitator (orange): a factor with a log(OR) >0, indicating a higher screening uptake for the comparison group vs the reference group. Barrier (blue): a factor with a log(OR) <0, indicating a lower screening uptake for the comparison group vs the reference group.

**Figure 5:**
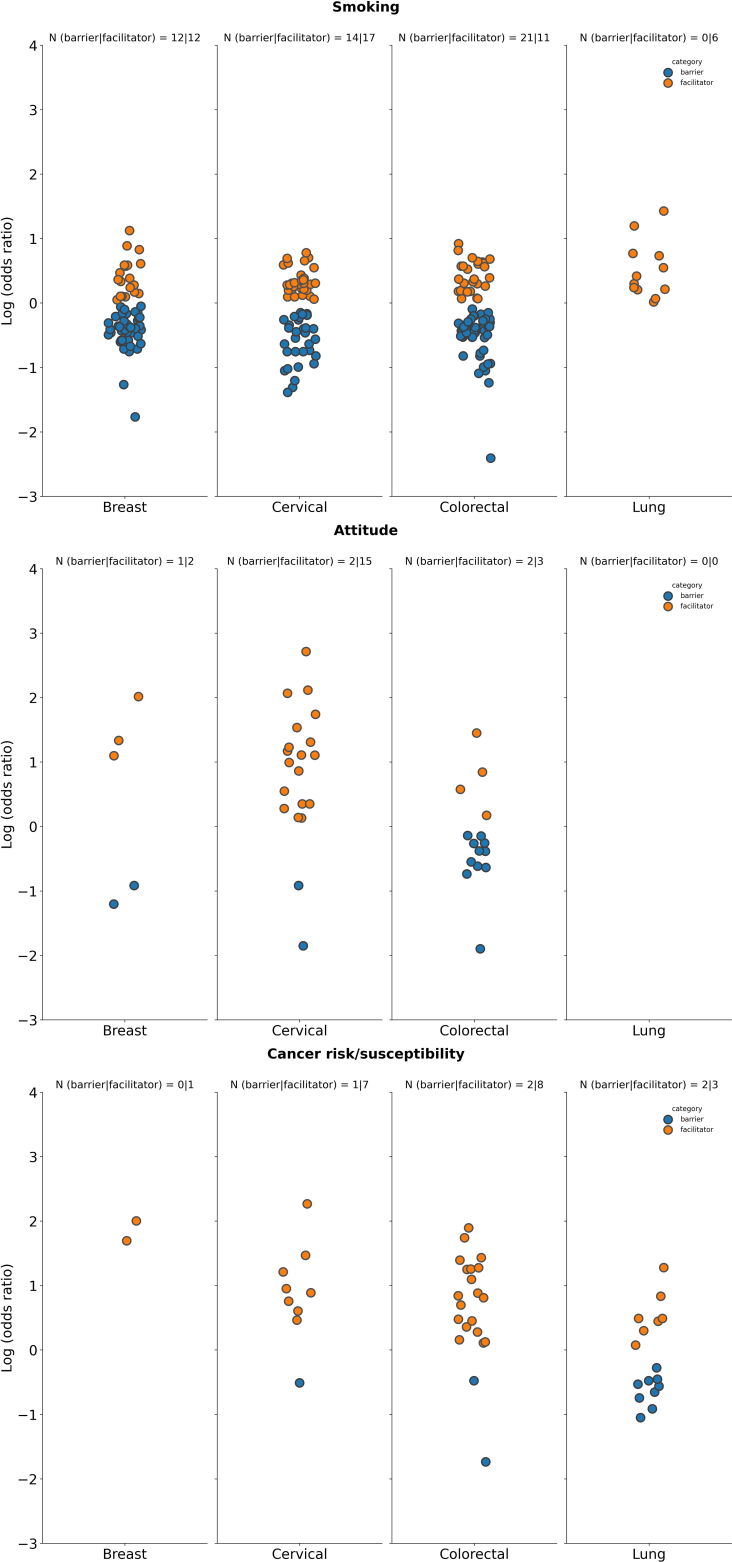
Summary of logarithm of odds ratios according to tumor type for selected patient behavior-related factors. Smoking status: Datapoints represent log(ORs) for groups of “current” smokers (vs. groups of “former”, “previous”, or “never” smokers as reference); Attitude: Datapoints represent log(ORs) for groups with “positive” or “favorable” attitude (vs. those with “negative”, or “poor” as reference); Cancer risk: Datapoints represent log(ORs) for groups with “higher”, “moderate” perceived cancer susceptibility (vs. those with “no” or “lower” perceived susceptibility to cancer as reference). The number of studies for which a specific factor acted as a barrier (in blue) or a facilitator (in orange) is shown as “N” atop each panel. A single study may be categorized as both a barrier and a facilitator if different strata within it showed opposing associations with screening uptake for the same factor. Facilitator (orange): a factor with a log(OR) >0, indicating a higher screening uptake for the comparison group vs the reference group. Barrier (blue): a factor with a log(OR) <0, indicating a lower screening uptake for the comparison group vs the reference group.

**Figure 6:**
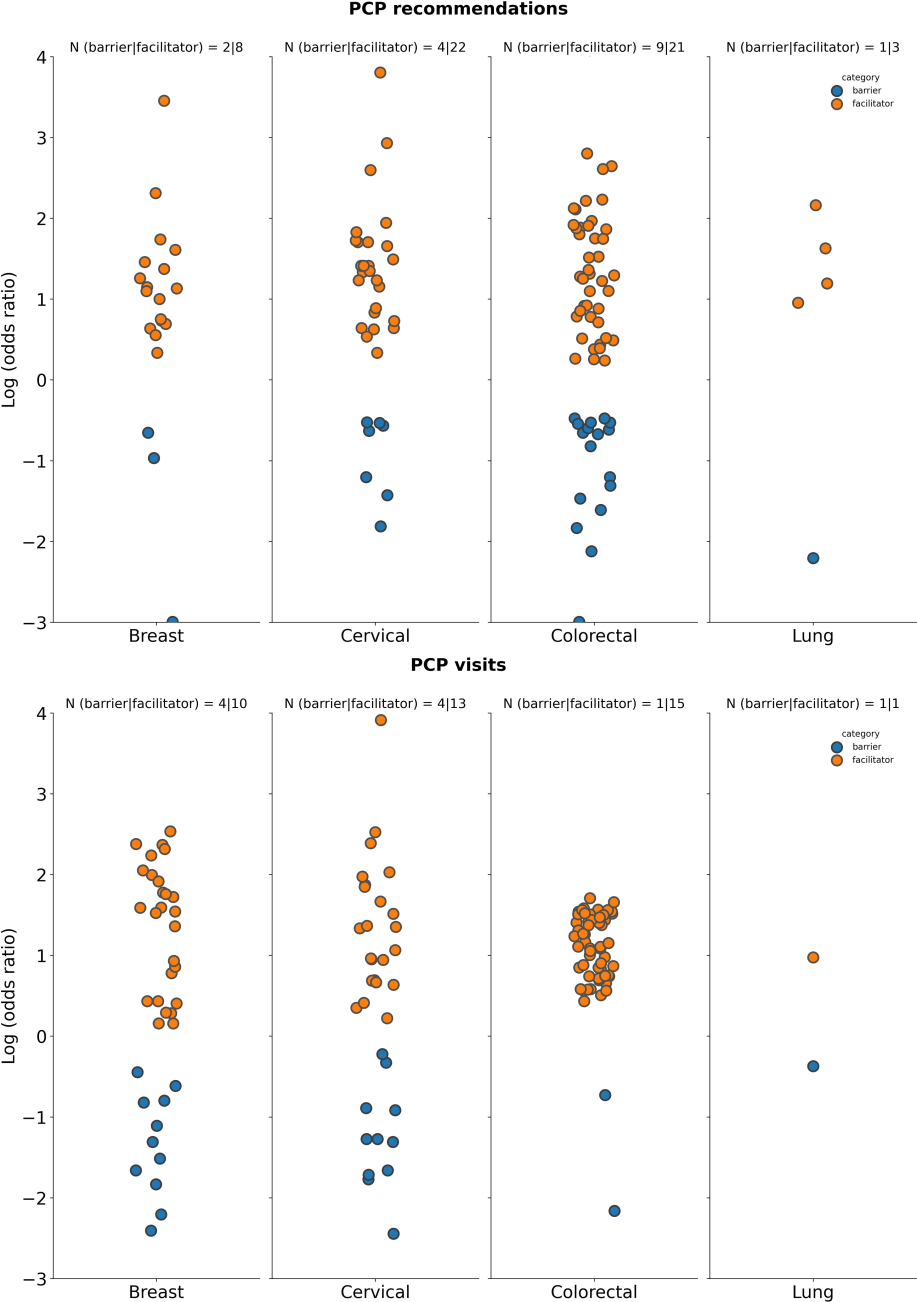
Summary of logarithm of odds ratios according to tumor type for selected PCP factors. PCP recommendations: Datapoints represent log(ORs) for groups who received a cancer screening recommendation by a PCP (vs. those who did not receive such a recommendation from a PCP as reference); PCP visits: Datapoints represents log(ORs) for groups who had no visits to the PCP or had the lowest number of visits to the PCP (vs. those that had visits or had a high number of visits to the PCP). The number of studies for which a specific factor acted as a barrier (in blue) or a facilitator (in orange) is shown as “N” atop each panel. A single study may be categorized as both a barrier and a facilitator if different strata within it showed opposing associations with screening uptake for the same factor. Facilitator (orange: a factor with a log(OR) >0, indicating a higher screening uptake for the comparison group vs the reference group. Barrier (blue): a factor with a log(OR) <0, indicating a lower screening uptake for the comparison group vs the reference group.

**Figure 7:**
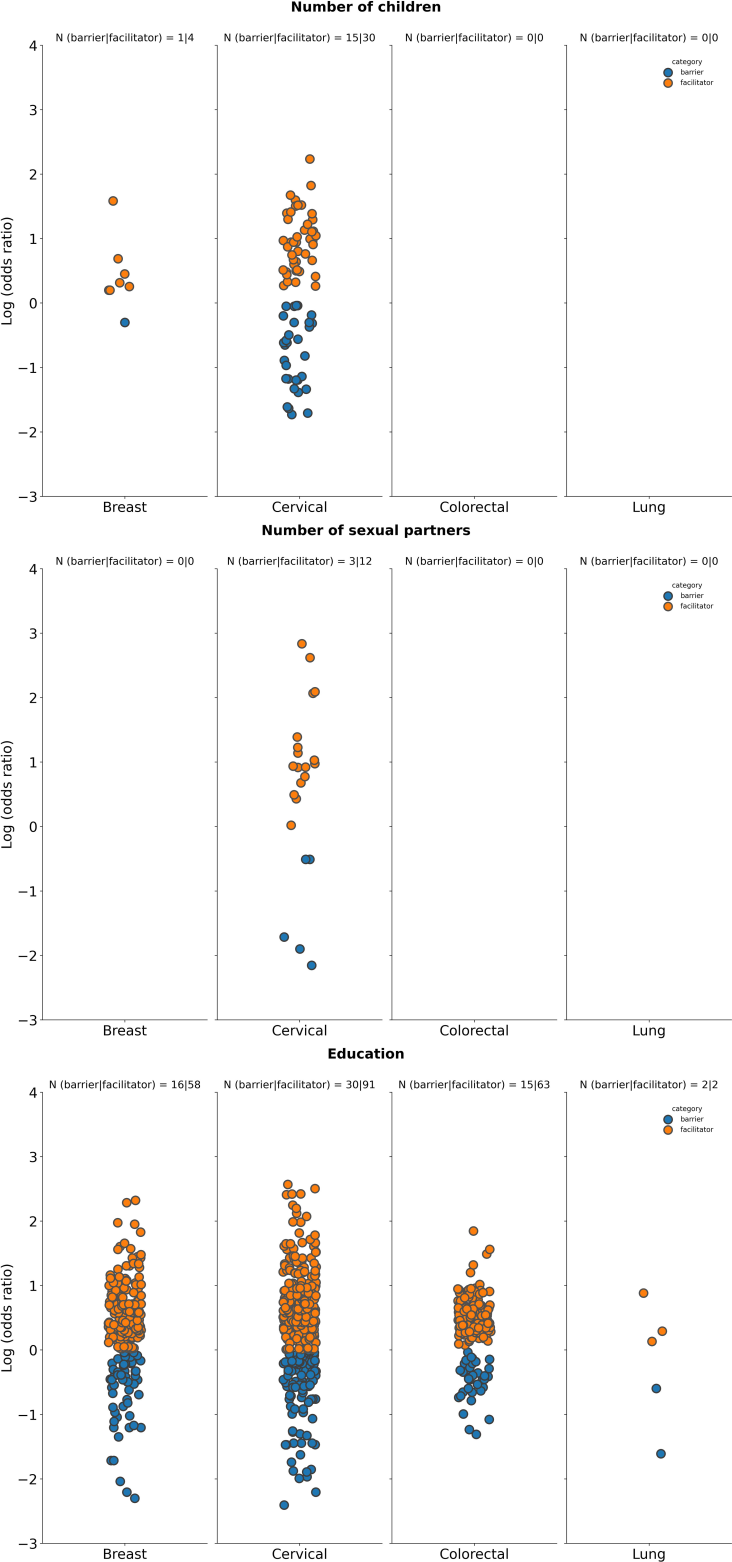
Summary of logarithm of odds ratios according to tumor type for selected social factors. Number of children: Datapoints represents log(ORs) for groups of patients with at least one child (vs. groups of patients with no children as reference); No. of sexual partners: Datapoints represents log(ORs) for those that have had more than one sexual partner (vs. those that have had 0 to 1 sexual partner as reference); Education: Datapoints represents log(ORs) for “high education”, “≥ high school graduate”, “tertiary education”, “university/college graduate” groups (vs. groups with “low education”, “no school”, “no formal education”, “illiterate”, “<high school”, “did not graduate high school”, “primary school”, or “middle school” as reference). The number of studies for which a specific factor acted as a barrier (in blue) or a facilitator (in orange) is shown as “N” atop each panel. A single study may be categorized as both a barrier and a facilitator if different strata within it showed opposing associations with screening uptake for the same factor. Facilitator (orange): a factor with a log(OR) >0, indicating a higher screening uptake for the comparison group vs the reference group. Barrier (blue): a factor with a log(OR) <0, indicating a lower screening uptake for the comparison group vs the reference group.

For data visualization purposes, the number of included studies per factor and per tumor type was established based on the availability of statistically significant OR data per factor (as detailed in section 2) and considering factors that were reported in at least 10 studies per tumor type. The OR data was presented on a logarithmic scale allowing for a symmetrical representations of equivalent associations. A total of 158, 242, 205, and 26 studies (not mutually exclusive for barriers or for facilitators) that evaluated factors associated with the uptake of screening for breast cancer, cervical cancer, CRC, and lung cancer, respectively, were represented through **Figures 2**–**7** (see section 3.2) **Figure 1**. PRISMA flow diagram. Results for gastric cancer and prostate cancer are presented in **Supplementary Appendix S6**.

### 3.2 Barriers and facilitators to cancer screening

#### 3.2.1 Demographics

Several demographic-related barriers and facilitators to cancer screening were identified, with age being the most identified factor, considered in 41% (157/384 studies) of the included studies. In general, older age is a facilitator to cancer screening across multiple tumor types [≥50 years for breast and lung cancer; ≥30 years for cervical cancer, and ≥60 years for CRC; (**Figure 2**)]. Studies reporting older age groups as a facilitator compared to the reference group were as follows: 33 out of 40 studies reported older age as a facilitator for breast cancer vs 15 out of 40 studies as a barrier; 40 out of 52 studies reported older age as a facilitator vs 25 out of 52 studies as a barrier for cervical cancer; 55 out of 67 studies reported older age as a facilitator vs 17 out of 67 studies as a barrier for CRC; and 5 out of 6 studies reported older age as a facilitator vs 2 out of 6 studies as a barrier for lung cancer. This trend was consistent with gastric and prostate tumour types (**Supplementary Appendix S6**).

Compared to individuals with less than good health status, perceived good health status was generally reported to be a barrier to cancer screening across multiple tumor types (**Figure 2**). Specifically, for breast cancer 30 out of 44 studies reported less than good health status as a facilitator to screening compared to 24 out of 44 studies identifying it as a barrier; for CRC, 52 out of 68 studies identified less than good health status as a facilitator to screening vs 34 out of 68 studies as a barrier, and for lung cancer, 3 out of 4 studies identified less than good health status as a facilitator compared to 2 out of 4 studies as barrier. Similar findings were observed for gastric and prostate cancer, with more studies indicating that less than good health status as a facilitator than a barrier to cancer screening (**Supplementary Appendix S6**). However, for cervical cancer there were less studies reporting less than good health status as a facilitator (51 out of 89 studies) vs as a barrier (55 out 89 studies) to screening uptake.

Across all tumor types, having previous experience with cancer was linked to facilitating screening uptake as opposed to this factor acting as barrier (**Figure 2**). Specifically, there were 7 out of 7 studies identified this being a facilitator vs 0 out of 7 studies as a barrier in breast cancer; there were 3 out of 4 studies identifying this as a facilitator vs 1 out of 4 studies as a barrier in cervical cancer; there were 13 out of 13 studies identifying this as a facilitator vs 0 out of 13 studies as a barrier in CRC, and 4 out of 4 studies identifying this as a facilitator vs 0 out of 4 studies as a barrier in lung cancer. This trend was consistent with gastric and prostate tumour types (**Supplementary Appendix S6**).

Similarly, there were more studies that identified having a family history of cancer as a facilitator to screening vs a barrier [breast cancer, 6 out of 7 studies as facilitators vs 1 out of 7 studies as barrier; cervical cancer, 10 out of 11 studies as facilitators vs 2 out of 11 studies as barriers; CRC, 16 out of 17 studies as facilitators vs 3 out of 17 studies as barriers; lung cancer, 1 out of 1 study as a facilitator vs 0 out of 1 studies as barriers (**Figure 2**)]. Similar results were seen with gastric and prostate cancer with more studies that identified having a family history of cancer as a facilitator to screening rather than a barrier (**Supplementary Appendix S6**).

Being an unmarried individual was generally a barrier rather than a facilitator to cancer screening for breast, cervical, and colorectal cancer [breast cancer, 12 out of 41 studies as facilitators vs 32 out of 41 studies as a barrier, cervical cancer 23 out of 76 studies as facilitators vs 56 out of 76 studies as barriers, CRC 33 out of 44 studies as facilitators vs 12 out of 44 studies as barrier (**Figure 2**)]. However, for lung cancer, one study was found to identify being unmarried or single as facilitating cancer screening uptake (with no studies identifying it as a barrier), compared to being married or in a partnership (**Figure 2**). For gastric cancer, unmarried or single status was observed to be a both a facilitator and a barrier in identified studies, while conversely, for prostate cancer, more studies identified unmarried status as a facilitator rather than a barrier for cancer screening (**Supplementary Appendix S6**).

Race and ethnicity demonstrated varying influences on screening uptake (**Figure 2**). Non-White populations had more studies identifying non-White race as a facilitator for undergoing screening for breast cancer (22 out of 26 studies) vs as a barrier (14 out of 26 studies), along with cervical cancer (20 out of 28 studies as a facilitator vs 18 out of 28 studies as a barrier) compared to White populations. Conversely, a higher number of studies for CRC and lung cancer identified non-White race as a barrier to screening (35 out of 52 studies and 4 out of 6 studies, respectively) vs a facilitator (28 out of 52 studies and 2 out of 6 studies, respectively). Similar to CRC and lung cancer, for prostate cancer, more studies identified non-White race as a barrier rather than a facilitator to cancer screening, and no studies identified race as either a barrier or facilitator for gastric cancer (**Supplementary Appendix S6**).

#### 3.2.2 Economic factors

Economic factors such as deprivation (most deprived vs least deprived), employment status (e.g., employed vs unemployed or retired), income (high vs low), socioeconomic status (low vs high), or region (e.g., urban vs rural) influenced cancer screening uptake across multiple tumor types. Individuals with high income and socioeconomic status, those least deprived, and those from urban areas were more likely to undergo cancer screening compared to their counterparts with low income and low socioeconomic status, those most deprived, and those from rural areas (**Figure 3**).

Experiencing deprivation was identified as a barrier rather than a facilitator to cancer screening. For breast cancer, only 1 out of 5 studies identified being most deprived as a facilitator to screening while 4 out of 5 studies identified being most deprived as a barrier. For cervical cancer all 4 studies identified being most deprived as a barrier to screening uptake. As with breast and cervical cancer, CRC identified deprivation as a barrier for screening uptake, with 5 out of 8 studies identifying this factor as a facilitator to screening and 7 out of 8 studies identifying this factor as barrier. No studies reported deprivation as a factor for influencing lung (**Figure 3**), gastric or prostate cancer screening uptake (**Supplementary Appendix S6**).

The relationship between employment status and cancer screening uptake varied by tumor type. In breast cancer, 11 out of 23 studies reported employment as a facilitator to screening compared to 15 out of 23 studies that reported it as a barrier. For cervical cancer screening, more studies identified employment as a facilitator (23 out of 31 studies) compared to as a barrier (11 out of 31 studies). Like breast cancer, for CRC, fewer studies identified employment as a facilitator to screening (10 out of 22 studies) compared to as a barrier (18 out of 22 studies). In lung cancer there were the same number of studies that identified employment as a facilitator (1 out of 2 studies) and as a barrier. For both gastric and prostate cancer, employment was seen as more of a barrier than a facilitator to cancer screening (**Supplementary Appendix S6**).

A higher income was linked to higher screening uptake, with all tumor types reporting more studies identifying a higher income as a facilitator (breast, 25 out of 34 studies; cervical, 40 out of 49 studies; CRC, 32 out of 44 studies; lung, 2 out of 2 studies) than as a barrier (breast, 11 out of 34 studies; cervical, 15 out of 49 studies; CRC, 15 out of 44 studies; lung, 0 out of 2 studies). This trend was also observed for gastric and prostate cancer (**Supplementary Appendix S6**).

Having a high socioeconomic status served as a facilitator for screening of breast cancer (7 out of 8 studies as a facilitator vs 1 out of 8 studies as a barrier), cervical cancer (22 out of 25 studies as a facilitator vs 4 out of 25 studies as a barrier) and CRC (10 out of 13 studies as a facilitator vs 5 out of 13 studies as a barrier). No studies on screening for lung cancer reported socioeconomic status as influencing cancer screening uptake. This trend with high socioeconomic status as a facilitator rather than a barrier to screening was consistent for gastric and prostate cancer (**Supplementary Appendix S6**).

Geographical location linked to socioeconomic factors also seemed to influence screening uptake and living in an urban area compared to a rural one was a facilitator in more studies across the majority of tumor types (breast, 19 out of 22 studies; cervical, 20 out of 23 studies; CRC, 20 out of 24 studies; lung, 4 out of 5 studies) rather than as a barrier (breast, 5 out of 22 studies; cervical, 4 out of 23 studies; CRC, 7 out of 24 studies; lung, 1 out of 5 studies). Conversely, for gastric cancer, more studies identified living in an urban area as a barrier rather than a facilitator to cancer screening, while for prostate cancer an equal number of studies identified living in an urban area as a facilitator and barrier to screening (**Supplementary Appendix S6**).

#### 3.2.3 Patient access

The relationship between patient access and cancer screening uptake depends on the particular factor, as well as the tumor type. Among factors identified in this study, having knowledge about cancer (high vs poor), health insurance coverage (insured vs non-insured), access to care (yes vs no), and history of screening (yes vs no), show that patient access is a facilitator for cancer screening for the majority of cancer types (**Figure 4**).

Regardless of tumor type, patients with some knowledge of the disease have a higher screening uptake compared to those with no knowledge (**Figure 4**). For breast cancer, disease knowledge was a facilitator to cancer screening in 12 out of 16 studies and was a barrier in 4 out of 16 studies. For cervical cancer, it was a facilitator in 52 out of 58 studies vs a barrier in 9 out of 58 studies. Similarly, in colorectal and lung cancer, disease knowledge was a facilitator in 10 out of 12 studies and 2 out of 2 studies, respectively, and was a barrier in 4 out of 12 studies and 0 out of 2 studies, respectively. For prostate cancer more studies reported disease knowledge as a facilitator than as a barrier to cancer screening (**Supplementary Appendix S6**).

As with disease knowledge, there was a much greater number of studies showing that health insurance coverage and access to care are facilitators to cancer screening uptake for all tumor types [breast cancer, 31 out of 35 studies as a facilitator vs 5 out of 35 studies as a barrier; cervical cancer, 28 out of 33 studies as a facilitator vs 6 out of 33 studies as a barrier; CRC, 42 out of 46 studies as a facilitator vs 6 out of 46 studies as a barrier; lung cancer, 3 out of 3 studies as a facilitator vs 0 out of 3 studies as a barrier (**Figure 4**)]. This included gastric cancer and prostate cancer (**Supplementary Appendix S6**).

Across all tumor types there is a greater number of studies showing that access to care is a facilitator rather than a barrier (breast cancer, 13 out of 16 studies as a facilitator vs 4 out of 16 studies as a barrier; cervical cancer, 23 out of 26 studies as a facilitator vs 7 out of 26 studies as a barrier; CRC, 24 out of 28 studies as a facilitator vs 6 out of 28 studies as a barrier; lung cancer, 4 out of 5 studies as a facilitator vs 2 out of 5 studies as a barrier [**Figure 4**]). Similar findings were seen for prostate cancer; however, for gastric cancer, there were no studies reporting this as a factor influencing screening uptake (**Supplementary Appendix S6**).

A history of cancer screening increases subsequent cancer screening uptake, with more studies across all tumor types reporting this as a facilitator influencing a higher screening uptake (breast, 12 out of 15 studies; cervical, 13 out of 17 studies; CRC, 21 out of 24 studies; lung, 1 out of 1 study) as opposed to as a barrier [breast, 4 out of 15 studies; cervical, 5 out of 17 studies; CRC, 4 out of 24 studies; lung, 0 out of 1 study (**Figure 4**)]. Similar findings were seen for prostate cancer; however, for gastric cancer, there were no studies that reported a history of cancer screening as a factor which impacts screening uptake (**Supplementary Appendix S6**).

#### 3.2.4 Patient behavior

There were a fewer number of studies that explored the relationship between patient behavior and cancer screening uptake compared with patient access (**Figure 5**). Patients with a positive attitude towards cancer screening was found to influence screening uptake [breast cancer, 2 out of 3 studies as a facilitator vs 1 out of 3 studies as a barrier; cervical cancer, 15 out of 17 studies as a facilitator vs 2 out of 17 studies as a barrier; CRC, 3 out of 5 studies as a facilitator vs 2 out of 5 studies as a barrier; no studies reported on patient attitude on the impact of lung cancer screening (**Figure 5**)]. No studies reported on the relationship between patient behavior and cancer screening in gastric and prostate cancers (**Supplementary Appendix S6**).

Moderate or high perceived cancer risk was identified as a patient behavior associated factor that generally facilitated cancer screening uptake across tumor types [breast cancer, 1 out of 1 studies as a facilitator vs 0 out of 1 studies as a barrier; cervical cancer, 7 out of 8 studies as a facilitator vs 1 out of 8 studies as a barrier; CRC, 8 out of 9 studies as a facilitator vs 2 out of 9 studies as a barrier; lung cancer, 3 out of 5 studies as a facilitator vs 2 out of 5 studies as a barrier (**Figure 5**)]. No studies were reported for gastric cancer and only one study identified perceived cancer risk as a factor influencing screening uptake in prostate cancer, reporting higher perceived cancer risk as a facilitator over a barrier (**Supplementary Appendix S6**).

The link between smoking and cancer screening is dependent on the tumor type (**Figure 5**). Breast cancer had the same number of studies identifying smoking as a barrier and facilitator (12 out of 22 studies). For screening programs targeting cervical and lung cancer, there were higher number of studies reporting smoking as a facilitator vs a barrier to screening uptake (17 studies vs 14 studies out of 27 studies assessing cervical cancer screening, and 6 studies vs 0 studies out of 6 studies evaluating lung cancer screening). However, for CRC screening programs, a greater number of studies identified smoking as a barrier vs facilitator to cancer screening uptake (21 out of 28 studies vs 11 out of 28 studies, respectively). Both gastric and prostate cancer had more studies identifying smoking as a barrier (**Supplementary Appendix S6**).

#### 3.2.5 PCP access

PCP access, represented by the number of visits (lower vs higher) or recommendations on screening (received vs not received) from PCPs, can impact patient’s adherence to screening programs. A higher number of studies indicated that PCP recommendations for cancer screening and those patients who had access to PCP visits were factors facilitating cancer screening for all prioritized tumor types (**Figure 6**). Specifically, in breast cancer, 8 out of 9 studies found that PCP recommendations were a facilitator vs 2 out of 9 studies as a barrier, for cervical cancer, 22 out of 26 studies found that it was a facilitator vs 4 out of 26 studies as a barrier, for CRC, 21 out of 28 studies as a facilitator vs 9 out of 28 studies as a barrier, and for lung cancer, 3 out of 3 studies as a facilitator vs 1 out of 3 studies as a barrier. Similarly, gastric, and prostate cancer had a higher number of studies reporting PCP recommendations for screening as a facilitator rather than a barrier to cancer screening (**Supplementary Appendix S6**).

A higher number of PCP visits was also identified across reported studies as a factor associated with facilitating screening uptake rather than as a barrier to screening across all prioritized tumor types (**Figure 6**). In breast cancer it was reported that 10 out of 12 studies identified PCP visits as a facilitator vs 4 out of 12 studies as a barrier; cervical cancer 13 out of 17 studies identified this factor as a facilitator vs 4 out of 17 studies finding it to be a barrier, and 15 out of 16 studies on CRC found that PCP visits were a facilitator compared to 1 out of 16 studies where it was a barrier. For lung cancer, there were the same number of studies identifying PCP visits as a facilitator or as a barrier; 1 out of 2 as a facilitator and 1 out of 2 studies as a barrier. No studies were analyzed commenting on PCP visits as an influencing factor on screening for gastric and lung cancer (**Supplementary Appendix S6**).

#### 3.2.6 Social factors

Various social factors may have an impact on individuals’ screening behavior. The number of children (at least one vs none), the number of sexual partners (more than 1 vs zero to one), or education (high or low) were the most frequently reported social factors impacting cancer screening. The number of sexual partners was a factor which was only assessed as either a facilitator or barrier to cancer screening uptake for cervical cancer (**Figure 7**), while the number of children reported as a facilitator or a barrier in studies focused on breast or cervical cancer only. In breast cancer, having one or more children was identified as a facilitator to screening in 4 out of 4 studies vs 1 out of 4 studies reporting it as a barrier. For cervical cancer, there were more studies that identified having at least one child and more than one sexual partner as facilitators (30 out of 39 studies and 12 out of 14 studies, respectively) rather than as barriers to cancer screening (15 out of 39 studies and 3 out of 14 studies, respectively; **Figure 7**). No studies assessing impact of the number of children or sexual partners on cancer screening were identified for gastric or prostate cancer (**Supplementary Appendix S6**).

In regard to education, breast, cervical and colorectal cancer all had more studies identifying higher education as a facilitator (62 out of 69 studies, 91 out of 111 studies, and 63 out of 74 studies, respectively) rather than a barrier (15 out of 69 studies, 30 out of 111 studies, and 15 out of 74 studies, respectively) to cancer screening (**Figure 7**). For lung cancer, there were 2 out of 4 studies that identified education as a facilitator vs 2 out of 4 studies that identified it as a barrier to cancer screening (**Figure 7**). Both gastric and prostate cancer studies also found higher education to be a facilitator to screening uptake (**Supplementary Appendix S6**).

#### 3.2.7 Other factors

**Figure 8:**
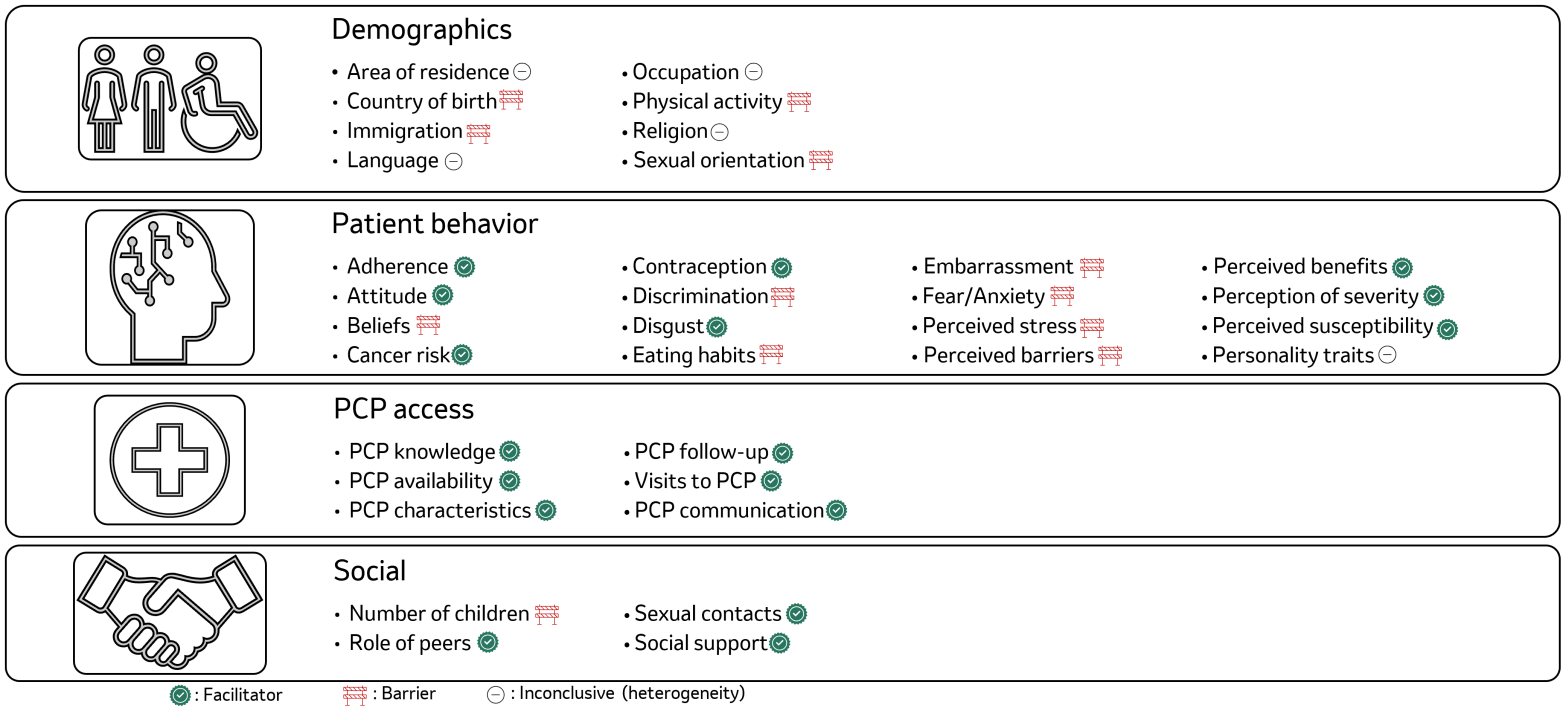
Qualitative summary of other barriers and facilitators to cancer screening uptake. Demographics are parameters that describe parameters from population perspective and impact the uptake of screening. Area of residence within this category includes factors such as country/place/region of residence, citizenship, nationality; Patients behavior includes parameters that were influenced by or were a direct result of people’s behavior; PCP access describes the hinderance associated with access to the PCP; Social barrier included the parameters that were related to society or its impact. For more information, refer to **Supplementary Appendix S3**.

Across the included studies evaluating factors associated with screening for the tumor types assessed, other factors influencing cancer screening uptake that were captured in less than 10 studies were also identified (**Figure 8**). Some of these factors were identified as barriers to cancer screening, including country of birth (31–41), being a migrant (34, 42–47), not being engaged in physical activity (34, 44–47), sexual minority identification (i.e., being transgender, gay or lesbian) (48–50), having negative patients’ beliefs (51), experiencing discrimination (i.e., racial or other forms, such as age- or gender-based discrimination) (52–57), having unhealthy eating habits (52–54), and negative emotions and perceptions (including embarrassment, fear/anxiety, perceived stress, and perceived barriers to screening) (58–76).

There were other factors that were identified as facilitators to cancer screening, including: patients’ positive perception on treatment adherence, positive attitude to cancer screening (77, 78), an understanding of cancer risk (79), lack of disgust of the screening procedure (66, 69, 80), perceived benefits of cancer screening (61, 81–85), perception of disease severity (64, 85–89), perception of the patients’ susceptibility to developing cancer (61, 83–85), having peer support or other social support advocating for cancer screening (64, 85–89), along with PCP knowledge, availability, frequent follow-ups, and positive approach to PCP communication (90–92). Specific facilitators that increased the likelihood of cancer screening included having a high number of sexual contacts (61, 88, 93) and no history of oral contraceptive use (94) for cervical cancer screening.

In contrast, factors like area of residence (43, 95–105), specific language spoken and language proficiency (96–98), type of occupation (106), type of religion or belief systems (94, 107–109), and personality traits (101, 110) exhibited a more complex influence, functioning as either barriers or facilitators depending on specific circumstances.

### 3.3 Interventions to overcome barriers to screening

Interventions aimed at overcoming barriers mentioned in the identified studies have been summarized, where details of the studies are provided in **Supplementary Appendix S5**. Across all tumor types, targeted educational programs were the most frequently discussed strategy for increasing cancer screening uptake (cervical cancer: 12 studies (49, 111–118); CRC: 6 studies (119–124); breast cancer: 3 studies (49, 125, 126); lung cancer: 1 study (127); multiple cancers: 1 study across multiple cancers (128). These programs aimed to improve knowledge on cancer risks and the benefits of early detection, reaching patients through diverse channels such as community workshops, online resources, and multilingual brochures. Tailored education was determined to be essential for underrepresented groups who face unique barriers, addressing disparities in cancer screening participation across racial, linguistic, and socioeconomic differences. For instance, culturally sensitive educational content and language-specific materials can improve understanding and engagement in cancer screening among diverse communities (118, 122, 124). Another key intervention discussed across studies in multiple tumors was increasing patients’ and healthcare professionals’ awareness of cancer screening and the benefits of early detection of cancer (cervical cancer: 9 studies (129–132); breast cancer: 4 studies (49, 75, 112, 115, 130, 132–135); CRC: 2 studies (119, 122)), particularly on awareness campaigns focusing on educating patients and healthcare professionals alike about the importance of cancer screening, dispelling misconceptions, and reducing patients’ fear associated with screening. Health professionals, including nurses, pharmacists, and health educators, play a key role in communicating the benefits of screening and it is equally important to guide them in effective communication of their explanations and demonstrations to patients about cancer and its screening process (49, 130, 136).

Specific strategies to raise such awareness through social media and community networks serve as effective tools to support cancer screening by creating spaces for individuals to discuss, learn, and share experiences related to screening and were identified in a number of studies (cervical cancer: 9 studies (49, 75, 112, 115, 130, 132–135); breast cancer: 4 studies (129–132), CRC: 2 studies (119, 122)). Platforms that promote peer discussions, educational meetings, and patient support groups allow individuals to interact with healthcare professionals and peers who have undergone screening. Such community-driven approaches reduce anxiety, increase comfort, and facilitate informed decision-making. Programs using these methods have shown promise in increasing screening participation for cancers like breast cancer, cervical cancer, and CRC. In particular, for women, authors note that breast cancer awareness programs should include oral presentations in an aim to educate women about risks for developing cancer, reducing fears, encouraging screening, and improving relationships with their healthcare providers (131). Thereby, making breast cancer screening more approachable, highlighting the benefits of early diagnosis and ultimately increasing participation rates (125, 129, 131).

National cancer screening programs and targeted governmental policies are critical strategies to increase screening uptake and reduce healthcare disparities (lung cancer: 2 studies (127, 137)); breast cancer: 1 study ( (95)). National policies that ensure insurance coverage for screening, such as the Affordable Care Act (ACA) in the U.S., can help reduce racial and economic disparities in cancer screening access (95, 127, 137). One study in breast cancer suggests that these policies ensure consistent access to healthcare providers and provide patient navigation services to prevent individuals from being missed. Such interventions are expected to effectively promote breast cancer screening and contribute to the overall reduction of disparities in healthcare outcomes (95). Of the included studies, two proposed strategies included public health and policy interventions that take into account the environmental context, which may improve effectiveness and practicality compared to patient-level approaches (132, 138).

Incorporating screening as a standard of care within national health programs and providing patient navigation services may also enhance screening uptake, especially for underserved populations (95, 132, 138). To mitigate the effect of economic barriers, specifically insurance coverage on screening uptake, suggestions were made for covering lung cancer screening under national cancer screening programs (127, 137). In other studies, the use of lung cancer screening coordinators and tracking software was also suggested to improve adherence and timeliness of cancer evaluation, in turn increasing cancer screening uptake (139). Notably, these components are recommended by the American Thoracic Society and American College of Chest Physicians in lung cancer screening (139).

Moreover, for rural and remote communities, mobile screening units and extended clinic hours have been proposed as effective means to improve access to screening. Policies that promote transportation support, accessibility of screening equipment, and nationally coordinated training for providers are also crucial in addressing the structural barriers to screening (140, 141).

Lastly, disparities across multiple sub-groups were reported in a total of 4 studies covering individuals with disabilities (1 study (138), rural populations (1 study (132)), Hispanic communities (1 study (95)), and transgender participants (1 study (49)) and specific interventions were proposed to enhance adherence to screening in these population sub-groups. For women with severe disabilities and brain-related and/or mental disabilities, access to information and equipment, transport support and access to usual care was suggested. For transgender and cisgender participants, improvements in capturing gender identity demographics, training to prevent perceived personal bias, and patient education on the importance of natal anatomy, were suggested as interventions to improve screening uptake. The promotion of adequate healthcare insurance coverage across these populations, and custom interventions such as mobile screening services, extended-hour screening in for rural and deprived communities were suggested as mechanisms to improve access to screening and adherence (49, 95, 132, 138).

### 3.4 Risk of bias

According to the NOS used to assess the risk of bias of the included studies, there was a significant variability in the quality of studies across different tumor types. The proportion of high-quality studies identified for breast cancer, cervical cancer, CRC, lung cancer, prostate cancer and gastric cancer was 22%, 25%, 15%, 11%, 16% and 25%, respectively. Most studies were included in the medium quality category, with a total score of 4-6 on the NOS scale. It was the case for 149/199 (75%) for breast cancer, 203/278 (73%) for cervical cancer, 194/246 (79%) for CRC, 29/37 (76%%) for lung cancer, 32/38 (84%) for prostate cancer and 9/12 (75%) for gastric cancer. Among the included studies, lung cancer exhibited the highest proportion of low-quality studies (14%). Detailed results are presented in the **Supplementary Appendix S7**.

## 4 Discussion

To our knowledge, this is the first review to adopt a pan-tumor approach to identify the barriers and facilitators to cancer screening uptake across multiple major tumor types – breast, cervical, colorectal, and lung cancers. This review identified factors influencing screening uptake, including patient demographic characteristics, socioeconomic factors, access to care, primary care provider involvement, and patient health behaviors, among others. Given that national screening programs exist in numerous countries for these cancers (142–146), understanding common and tumor-specific barriers and facilitators to screening is essential for improving early detection and reducing cancer-related mortality (5–7).

### 4.1 Summary of findings

The findings confirm that age and perceived health status significantly influence screening uptake across multiple tumor types. Older adults, for instance, are more likely to participate in screening for breast, colorectal, and lung cancers due to established screening guidelines that target older adults (142–146). For cervical cancer, age-specific incidence rates typically rise from age 15-19 year group and peaking in the age 30-34 year group, and then decline in subsequent age groups (147). As such, there was an observed trend for cervical cancer screening uptake declining with age (148–150), as older age can be a potential barrier given that national screening programs may have an age cut-off screening threshold (142–146), and as post-menopausal women may feel less at risk or who are no longer regularly seeing gynecologists (151). Therefore, such results indicate that tumor type-specific interventions may be needed to address screening uptake barriers by different age groups. Additionally, individuals with worse health status scores are generally more likely to adhere to screening guidelines, which suggests that a perception of personal health risk may motivate screening behaviors. However, they may also feel less urgency to undergo regular screening, seeing themselves as low risk (36, 77, 152–155). Furthermore, those with poor health or comorbidities may be less willing to screen due to competing health priorities, or conversely, they may see screening as critical to their ongoing care (153, 154).

Behavioral factors, such as having a positive attitude, the perception of being at risk of developing cancer, and smoking, were identified as notable facilitators, particularly for cervical cancer (99, 134, 136, 156–161), CRC (40, 74, 124, 154, 162–171), and lung cancer screening (128, 137, 172–181), respectively. Lung cancer screening is recommended across a number of countries, including Australia, Japan, Germany, UK, and the US for high-risk populations, with high-risk status based on age and smoking history (number of packs per year) of the individual (9, 182–184). People with chronic respiratory conditions may be more inclined to screen for lung cancer, seeing it as directly relevant to their health (127, 177, 178, 185). Additionally, a lack of awareness and knowledge about cancer risks and the benefits of screening was a barrier to screening uptake that was common across all tumor types. This is consistent with previous research indicating that awareness campaigns and health literacy are vital in promoting cancer screening, especially in underserved populations (186). However, if awareness efforts increase anxiety or fears about potential results, this can paradoxically deter people from participating in cancer screening (187). Nevertheless, addressing these informational barriers is a priority to ensure that individuals understand the importance of timely screening and its role in early detection.

Socioeconomic factors, including income, employment status, and insurance coverage, were also strongly associated with disparities in screening uptake (188). Low-income individuals and those without adequate insurance face significant barriers to screening, underscoring the need for financial and policy-level interventions (188). This aligns with existing literature showing that affordability and insurance coverage remain critical in enabling equitable access to cancer screening (189). While low socioeconomic status often acts as a barrier due to limited access to resources or insurance, higher socioeconomic status can facilitate screening by providing financial means, health literacy, and easier access to care (139, 190, 191). Conversely, some high-income groups may skip screening due to overconfidence in their health or the perception that screening is unnecessary (109, 126).

System-level, provider-level, and community-level barriers frequently interact, compounding their impact on screening participation. In resource-limited settings, for example, inadequate funding and workforce shortages lead to fewer screening facilities and trained providers, directly reducing screening capacity and access, particularly for high-cost screenings, such as those for lung and colorectal cancers. Proximity to screening centers or mobile units facilitates screening, but long wait times, scheduling issues, or inconvenient locations can make access a barrier (43, 140, 141). Providers can be powerful facilitators when they encourage and remind patients about screenings. However, lack of a strong recommendation from a provider often leads to low screening, effectively making the absence of provider engagement a barrier to screening uptake (113, 129, 192–197). Simplifying logistics often turns this barrier into a facilitator (198–205). This complex interplay underscores the need for multi-level, coordinated strategies to address these layered barriers.

Potential interventions to mitigate these barriers were identified across included studies, focusing on the roles of patient awareness, social support, and provider training. Building supportive networks for screening discussions, promoting awareness through diverse media channels, and improving provider-patient communication can foster a more supportive environment for screening uptake (129–132, 206). Healthcare providers play a central role in influencing patient decisions regarding screening; thus, interventions that improve provider skills in explaining the benefits of screening may help address patient concerns and reduce screening hesitancy (113, 129, 192–197).

Implementing tailored interventions is also critical to addressing unique barriers faced by vulnerable groups, including rural populations, ethnic minorities, and the LGBTQ+ community (23, 207, 208). For example, mobile screening units can increase access in rural areas where geographic distance is a barrier (140, 141). Linguistically and culturally adapted educational materials benefit specific populations, such as Hispanic communities, by ensuring that language and cultural values are respected (118, 122, 124, 209). Additionally, training providers in culturally competent care is essential to improve screening experiences for ethnic minorities and transgender individuals, who may face unique barriers due to stigma, lack of insurance coverage, or previous negative healthcare experiences (49, 95, 210). By prioritizing these tailored approaches, healthcare systems can more effectively promote equitable access to cancer screening for all individuals.

### 4.2 Strengths and limitations

To our knowledge, this is the first systematic review providing a broad, comparative understanding of common and unique barriers to cancer screening uptake. This study took a comprehensive approach by examining an extensive set of barriers across multiple tumor types, and identifying overlapping factors, such as socioeconomic status or provider influence. It also highlighted where interventions can be applied universally or need to be customized by tumor type. For the purpose of presenting the results, facilitators were interpreted alongside barriers. While the primary emphasis of the SLR was on studies reporting barriers to screening uptake, the identification of facilitators was deduced indirectly to include the full spectrum of factors.

However, due to the large amount of evidence identified during the searches, it was necessary for the review to focus on key themes observed in the literature, specifically, demographics, economic factors, patient behavior, PCP access, and social factors. Therefore, despite the broad inclusion criteria, it is important to note that alternative categorizations of factors contributing to cancer screening uptake could have yielded different insights or emphases in the reported findings. Additionally, although no restrictions were placed on tumor types, four key malignancies were selected for the purpose of reporting, including: breast cancer, cervical cancer, CRC, and lung cancer. These tumor types were prioritized based on the number of available studies and the existence of well-established screening programs.

Since the searches were limited to those published from 2012 to 2022, certain factors previously identified and included in studies published prior to 2012 may not have been captured. Furthermore, as the included studies were conducted in a variety of patient populations, some inter-study comparisons were limited due to the heterogeneity in study methodologies. It is also important to note that interventions to improve cancer screening uptake were not the primary objective of the study and those identified were from studies that focused on barriers to screening. As such, the study selection process for the studies that included information regarding interventions to overcome barriers to cancer screening was not systematic and only those studies that provided comprehensive details were selected for further analysis. This may have led to selection bias and there are likely to be additional studies not captured in the search that may report on other interventions. Additionally, any studies that reported on the effectiveness of the interventions were not captured, since assessing the impact of the interventions was not one of the study objectives.

While this review provides valuable insights into barriers to cancer screening uptake from the patient and healthcare practitioner (HCP) perspective, for example, it is important to acknowledge that the findings may not encompass all perspectives that could influence screening behaviors.

Additionally, the varying number of studies available for different cancer types (for example 37 for lung cancer to 278 for cervical cancer) represents a limitation in the available data. While this imbalance reflects the current state of research across different cancer screening programs, it may impact the depth and breadth of insights for less-studied cancer types. However, the consistency of factors identified across cancer types suggests that our findings remain robust despite this limitation.

Our study identified age as a potential barrier; however, evidence on age as a barrier requires careful interpretation. Screening guidelines by age ranges in adults differ across countries and cancer types. Some studies provided OR for age ranges outside the recommended screening thresholds, without accounting for country-specific screening age requirements. Lower screening rates outside these recommended ranges may reflect adherence to evidence-based guidelines rather than true barriers. Regarding this aspect, our review was limited in distinguishing between appropriate age-based screening practices and actual uptake barriers. Future research should more precisely delineate age-related barriers within the context of established guidelines and explore evidence for screening in age groups traditionally considered low-risk.

It should also be mentioned that interpretation of the results may be biased by ecological fallacy, in particular other factors or a set of different factors than those presented in this study might have an impact on patients’ behavior towards cancer screening. Therefore, caution is recommended when drawing conclusions from population-level data, and where feasible, individual patient-level data should be considered in future research to provide a more nuanced understanding of the results, while acknowledging that studies are often focused on a limited set of factors influencing screening behaviors. While our study focused on individual factors affecting cancer screening uptake, future research should explore potential interactions among these factors. For instance, investigating how economic and social factors might jointly influence screening participation could provide more nuanced insights and inform more targeted interventions. Future research should also explore the potential of artificial intelligence (AI) and machine learning in improving cancer screening uptake. AI could be leveraged to analyze patient medical data and provide personalized screening recommendations or risk profiles, implement automated reminder systems, and develop targeted outreach programs for areas with low screening rates. AI can be leveraged and developed into tools for medical decision making in the clinic, utilizing ML-based prediction models that help identify factors predictive of early stage cancer. Additionally, AI-enhanced patient education tools could be developed to improve understanding and engagement with screening programs.

Quality assurance using the NOS revealed a substantial number of medium and high-quality studies, especially in breast, cervical, and colorectal cancers, indicative of a robust foundation for evidence- based conclusions in these areas. However, significant methodological concerns across both low and medium-quality studies were also revealed, which represented the majority of the evidence base for lung cancer. Common deficiencies included lack of comparability between groups, insufficient information on selection of non-exposed cohorts or control groups, inadequate follow-up procedures, and reliance on self-reported data. These findings should be kept in mind when interpreting and generalizing the findings, particularly for tumor types with a higher proportion of low-quality studies. Additionally, while the NOS scale provided a standardized assessment of study quality, our review process revealed additional methodological considerations. Many factors influencing cancer screening uptake are interdependent, and this complexity is not always fully addressed in individual studies. Furthermore, there may be additional factors influencing screening behavior that were not consistently researched or accounted for across studies. The presence of potential confounding factors, which vary across different contexts, adds another layer of complexity to interpreting and generalizing results. These observations highlight the need for more comprehensive and nuanced approaches in future research on cancer screening uptake.

### 4.3 Implications to clinical practice and research

The interchangeable nature of barriers and facilitators across multiple tumor types has significant implications for healthcare, particularly in designing and implementing screening programs. Firstly, recognizing that a factor can act as a barrier for one tumor type, but a facilitator for another suggests that screening programs must be tailored to the specific context of each cancer. Generalized approaches may not be effective; healthcare providers should employ cancer-specific strategies that account for the unique perceptions, risks, and motivators of each tumor type. Secondly, given that factors like age, socioeconomic status, and cultural attitudes affect different cancers in varied ways, interventions must be tailored not only by tumor type, but also by demographics. For instance, older populations may need reassurance about the importance of cervical screening post-menopause, while younger populations might need more encouragement to consider lung cancer screening if they are at high risk. Finally, the variability in barriers and facilitators suggests that multi-level approaches combining patient-level, provider-level, and system-level interventions, may be more effective. Health systems should consider integrating community engagement, social support mechanisms, customized patient education initiatives along with healthcare provider training, and policy changes, such as expanding insurance coverage to bring equity to screening access, to target the full range of barriers across tumor types and demographics. Close collaboration between governments and healthcare providers, along with patient advocacy groups will be necessary to optimize their implementation; however, further research will first be required to assess cost-effectiveness and determine which interventions would offer the most effective use of finite resources.

The comprehensive analysis of barriers and facilitators to cancer screening presented in this review could serve as a foundation for developing a conceptual framework for cancer screening. Such a framework would not only guide the design of tailored, population-specific screening strategies but also inform the creation of cross-cutting, multi-cancer screening programs. By systematically addressing the various factors influencing screening uptake identified in this review and their interdependence, this conceptual framework could significantly impact screening participation rates and, ultimately, lead to earlier cancer detection and improved patient outcomes.

### 4.4 Conclusion

Barriers to cancer screening across multiple tumor types are complex, spanning demographic and patient-level factors, social and economic factors, provider and community challenges, and access to health care. While certain barriers are shared across tumor types, others are unique, reflecting the specific requirements of screening for different tumors. Addressing these barriers requires multi-level strategies that integrate both universal and cancer-specific approaches. Targeted interventions and supportive policies can increase screening participation, facilitate earlier cancer diagnosis, and reduce disparities in cancer outcomes.

## Data Availability

The raw data supporting the conclusions of this article will be made available by the authors, without undue reservation.
