# Supplementary material for: Barriers to cancer screening uptake and approaches to overcome them: a systematic literature review
Source: Front Oncol. 2025 Aug 6;15:1575820. doi: 10.3389/fonc.2025.1575820 (PMC12364675; doi:10.3389/fonc.2025.1575820)
Supplement: Supplementary file 1 [file DataSheet1.docx]

Supplementary Appendix

# **Appendix S1. Search strategy**

**Table 1. Search strategy for the SLR**

| No. | Query | Results |
| --- | --- | --- |
| 1 | (('cancer' OR 'carcinoma' OR 'neoplasm' OR 'tumor' OR 'tumour') NEAR/3 ('screening' OR 'screening test' OR screen*)):ti | 36343 |
| 2 | 'barrier*':ab,ti OR 'obstacle':ab,ti OR 'hurdle':ab,ti OR 'obstruction':ab,ti OR 'block':ab,ti OR 'poverty':ab,ti OR poor*:ab,ti OR educat*:ab,ti OR depriv*:ab,ti OR socioeconomic*:ab,ti OR income:ab,ti OR fatalistic:ab,ti OR fatalism:ab,ti OR embarrass*:ab,ti OR knowledge*:ab,ti OR relig*:ab,ti OR cultur*:ab,ti OR mistrust:ab,ti OR trust:ab,ti OR fear*:ab,ti OR communicat*:ab,ti OR language*:ab,ti OR understanding*:ab,ti OR belief*:ab,ti OR culture:ab,ti OR perception:ab,ti OR 'social perception':ab,ti OR perception*:ab,ti OR 'social perception*':ab,ti OR opinion*:ab,ti OR 'attitude to health':ab,ti OR attitude*:ab,ti OR 'social value*':ab,ti OR 'relationship':ab,ti OR 'literacy':ab,ti OR 'awareness':ab,ti OR 'abuse':ab,ti OR 'doctor':ab,ti OR 'scary':ab,ti OR 'crisis':ab,ti  OR 'uncomfortable':ab,ti OR 'rejection':ab,ti OR 'contagious':ab,ti OR 'stigma':ab,ti OR 'misguidance':ab,ti OR 'time consuming':ab,ti OR 'time':ab,ti OR 'negligence':ab,ti OR 'social stigma':ab,ti OR 'carelessness':ab,ti OR 'shy':ab,ti OR 'shyness':ab,ti OR 'lack of support':ab,ti OR 'high cost':ab,ti OR 'trust':ab,ti OR 'trustworthiness':ab,ti OR 'unapproachable':ab,ti OR 'accessibility':ab,ti OR 'scared':ab,ti | 12193011 |
| 3 | 'cohort study':ab,ti OR 'retrospective study':ab,ti OR 'cohort analysis':ab,ti OR 'longitudinal study':ab,ti OR 'prospective study':ab,ti OR 'observational study':ab,ti OR ((cohort NEXT/1 stud*):ab,ti) OR ((cohort NEXT/1 analy*):ab,ti) OR 'register':ab,ti OR 'registry':ab,ti OR (('database' NEAR/2 'study'):ab,ti) OR (('real' NEXT/1 'world'):ab,ti) OR (('healthcare' NEXT/1 'record'):ab,ti) OR 'pragmatic trial':ab,ti OR 'real-world clinical trial':ab,ti OR 'pragmatic clinical trial':ab,ti OR 'real-world':ab,ti OR 'real world':ab,ti OR 'database':ab,ti OR 'real-life':ab,ti OR 'real life':ab,ti OR 'database study':ab,ti OR 'cross sectional':ab,ti OR 'cross-sectional':ab,ti OR 'case control':ab,ti OR 'case-control':ab,ti OR 'survey':ab,ti OR 'questionnaire':ab,ti OR 'interview':ab,ti OR 'descriptive':ab,ti | 3882182 |
| 4 | #1 AND #2 AND #3 | 6697 |
| 5 | #1 AND #2 AND #3 AND ([conference abstract]/lim OR [conference paper]/lim OR [conference review]/lim OR [editorial]/lim OR [letter]/lim OR [note]/lim OR [review]/lim) | 2315 |
| 6 | #4 AND [animals]/lim NOT ([humans]/lim AND [animals]/lim) | 6 |
| 7 | #5 OR #6 | 2315 |
| 8 | #4 NOT #7 | 4382 |
| 9 | #4 NOT #7 AND [2012-2022]/py | 2870 |

# **Appendix S2. Eligibility criteria for study inclusion**

**Table 2. Summary of the inclusion/exclusion criteria for the barriers to screening review**

| **PICOTS** | **Inclusion Criteria** |
| --- | --- |
| Population(s) | Adults (≥18 years) of any gender or race, undergoing screening for cancer |
| Interventions | No restriction |
| Comparisons | No restriction |
| Outcomes | Key outcomes (not exhaustive list):   - Factors associated with adherence to available screening guidelines and program*   - Identification of patient profile/ risk assessment for recommended screening (e.g. age, smoking history, cancer history, etc.)   - Familial history of cancer   - Biomarker assessment (e.g. type of biomarker and prevalence) - Barriers associated with patient characteristics and beliefs   - Identification of predictors for low uptake (vs. high uptake) of screening (e.g. age, ethnicity/ racial group, tumor type, medical history/ comorbidities, family history of cancer, socioeconomic, education, health literacy etc.)   - Outlining patient beliefs and socio-cultural factors associated with low uptake of screening opportunities (vs. high uptake); beliefs may cover:     - The value of screening     - Fears associated with diagnosis.     - Mistrust of medical system     - Perceived value of symptomatic presentation     - Concerns regarding false-positive diagnoses     - Social perceptions on screening (e.g. for cervical cancer screening)     - Concerns over cancer fatalism and whether a patient wants to be aware of their diagnosis   - Symptom recognition among patients and physicians separately   - Health literacy among patients   - Median/ mean time from cancer diagnosis to treatment - Barriers associated with screening access   - Availability and funding for diagnostic tools and screening programs   - Literacy and/or symptom awareness among physicians   - Difficulty/ challenges attending screening (e.g. financial, mobility, etc.)     - Patient insurance coverage     - Patient rural living - Perspectives from scientific leaders (SLs) on screening recommendations/ guidelines   - Journal editorials and letters from SLs on the reasons and remedies for low update of screening - Interventions/approaches that can help overcome screening barriers, by tumor type and location   - Identification of:     - Educational interventions to improve health literacy     - Interventions that may alter patient beliefs about screening and cancer     - Less invasive/ more comfortable and more convenient screening techniques     - Programs to facilitate patient access to screening opportunities |
| Time | 10-year (2012-2022) |
| Study design | Study designs to be included are:   - Observational studies, including: - Cohort studies (prospective and retrospective) - Case control studies (prospective and retrospective) - Cross sectional studies - Longitudinal studies |
| Country | Global |
| Other (Language) | No restrictions |

Abbreviations: AJCC: American Joint Committee on Cancer; DFS: Disease-free survival; EFS: Event-free survival; FCR: Fear of cancer recurrence; NSCLC: Non-small cell lung cancer; OS: Overall survival; PFS: Progression-free survival; PICOTS: Population, intervention, comparisons, outcomes, time, study design; RCC: Renal cell carcinoma; RFS: Relapse-free survival; TNBC: Triple negative breast cancer

# **Appendix S3. Definitions of categories**

**Table 3. Definitions of categories**

| **Categories** | **Definitions** |
| --- | --- |
| **Demographic characteristics** | Demographics are parameters that describe the population and impact the uptake of screening, however, cannot be considered as a barrier. The following characteristics were grouped together in this sub-category:  Age  Area of residence (area of residence under demographics includes factors such as country/place/region of residence, citizenship, nationality, etc. Region under economic barriers includes factors such as rural/urban, city/inner region, metropolitan cities etc. which correspond to availability of healthcare and other resources in any particular region)  Cancer experience (refers to history of cancer of any type in the individual)  Country of birth  Family history (refers to history of cancer in family of individual)  Health status  Immigration  Language  Marital status  Occupation  Physical activity  Race/ethnicity  Religion/spirituality  Sexual orientation |
| **Economic barriers** | Economic barrier category included barriers related to wealth of people intended for screening.  The barriers included:  Deprivation  Employment status  Income  Region e.g., rural vs. urban (area of residence under demographics includes factors such as country/place/region of residence, immigration, citizenship, nationality, etc. Region under economic barriers includes factors such as rural/urban, city/inner region, metropolitan cities etc. which correspond to availability of healthcare and other resources in any region)  Socioeconomic status/ Social class/ Wealth/ Finances/ Poverty  Medical cost burden |
| **Patient access** | Patient access consisted of barriers that were associated with accessibility to resources.  The barriers include:  Access to health care/ Personalized care  Disease Knowledge/ awareness  Facility access/ Facilities  History of screening/checkup  Health insurance |
| **Patient’ behavior** | Behavior category of barriers included the parameters that were influenced by or were a direct result of people’s behavior.  The barriers include:  Adherence  Attitude  Belief  Cancer risk/ Susceptibility  Contraception  Discrimination  Eating habits  Embarrassment  Fear/ Anxiety/ Concern  Impact of multimedia/internet  Perceived stress  Perception of benefits  Perception of cancer risk/ susceptibility  Perception of health status  Perception of severity  Perception of treatment  Personality traits  Satisfaction  Smoking/drinking/substance abuse |
| **PCP access** | The hinderance associated with access to the PCP or factors associated with a PCP have been categorized under PCP access sub-category.  The barriers include:  Disease knowledge/ awareness by PCPs  PCP availability  PCP characteristics  PCP communication  PCP follow-up  PCP recommendations  PCP Visits |
| **Social barriers** | The category of social barrier included the parameters that were related to society or its impacts.  The barriers include:  Children status  Education  Role of peers  Social support  Working conditions |

Note: area of residence under demographics includes factors such as country/place/region of residence, immigration, citizenship, nationality, etc. Region under economic barriers includes factors such as rural/urban, city/inner region, metropolitan cities etc. which correspond to availability of healthcare and other resources in any particular region.

Abbreviations: PCP: primary care physician

# **Appendix S4. Distribution of included studies that reported factors across distinct categories of barriers according to tumor type**

**Figure 1. Distribution of included studies that reported factors across distinct categories of barriers/facilitators according to tumor type**

Abbreviations: CRC: Colorectal cancer; PT: Patient; PTS: Patient’s; PCP: Primary care provider

# **Appendix S5. Summary of included evidence**

**Table 4: Study characteristics of studies reporting factors with statistically significant ORs for single tumor types**

| **Study name** | **Study Objectives** | **Study design** | **Publication Type** | **Country** | **Data Source** | **Study setting** | **Data collection period** | **Outcome: Barriers** | **Outcomes: Interventions *[YES/NO]** |
| --- | --- | --- | --- | --- | --- | --- | --- | --- | --- |
| **Breast cancer** | | | | | | | | | |
| Abdel-Aziz 2018 (1) | This cross-sectional study aimed at investigating the perceived barriers towards BC screening in Al Hassa, Saudi Arabia. | Cross-sectional study | Journal article | Saudi Arabia | NR | National survey | 2013 | Demographics, Economic, Patient behavior, Social | No |
| Ahmadian 2012 (2) | To explore predictive factors that affect mammography adherence among Iranian women attending outpatient clinics. | Cross-sectional study | Journal article | Iran | NR | Multicenter | Jul 2009- Oct 2009 | Demographics, Economic, Patient access, Patient behavior, Social | No |
| Alatrash 2021 (3) | To examine associations of sociodemographic characteristics with perceived benefits and barriers to MS and explore relationships of MS with sociodemographic, and perceived benefits and barriers in Muslim and Christian AAW from three Arab countries, Jordan, Lebanon, and Egypt | Cross-sectional study | Journal article | USA | NR | Community- wide study | NR | Demographics, Patient behavior | No |
| Al-Azri 2020 (4) | To identify knowledge, attitudes and barriers of Omani women toward BCS | Cross-sectional study | Journal article | Oman | NR | Nationwide study | 1 Jan 2018 to 31 Mar 2018 | Patient access, Patient behavior | No |
| Al-Hanawi 2020 (5) | To investigate socioeconomic inequalities in breast cancer screening among Saudi women | Cross sectional study | Journal article | Saudi Arabia | Saudi Health Interview Survey (SHIS) | National survey | 2013 | Demographics, Economic, Social | No |
| Asgary 2014 (6) | This study aims to evaluate rates and predictors of and barriers to mammography among homeless and domicile women 50 to 74 years old who use NYC’s shelter-based clinics. | Retrospective study | Journal article | USA | New York City shelter-based clinics | Multicenter | September 2010 to December 2012 | Demographics, Patient access, Patient behavior, PCP access | No |
| Assefa 2021 (7) | To assess breast cancer screening practice and associated factors among women 20–70 years of age in an urban setting in SNNPR, Ethiopia. | Cross-sectional study | Journal article | Ethiopia | NR | Community wide study | March 1 to April 30, 2020 | Demographics, Patient access, Patient behavior, PCP access, Social | No |
| Balas 2020 (8) | To investigate the barriers to participation of a sample of Jamaican women in mammography screening. | Cohort based study | Journal article | Jamaica | We performed a retrospective analysis of collected data from a survey that was conducted in Jamaica from June to August 2013 in the four parishes served by the Western Regional Health Authority (WRHA) | Community based survey | June to August 2013 | Demographics, Patient access, Patient behavior, Social | No |
| Bao 2018a (9) | (i) to report on the current breast cancer screening rates among women in China; and (ii) to examine whether demographic factors and cultural beliefs about breast cancer and screening are associated with their screening behavior. | Cross-sectional study | Journal article | China | NR | City wide study | 2016 | Demographics, Economic, Social | No |
| Baughman 2016 (10) | The patient-centered medical home (PCMH) continues to gain momentum as a primary care delivery system. We evaluated whether medical home transformation of primary care practices is associated with the use of breast cancer screening, a broadly endorsed preventive service. | Retrospective cohort study | Journal article | USA | BWH-affiliated primary care clinics | Multicenter | Apr 2012 to Dec 2013 | Demographics, Patient access, Patient behavior, PCP access, Social | No |
| Bawazir 2019 (11) | This study assessed the breast cancer screening awareness and practices among women presenting to primary health care centers in the Ghail-Bawazir district of Yemen regarding BC screening. In addition, we seek to identify the barriers to BC screening among the study group. | Cross sectional survey | Journal article | Yemen | NR | District-wide survey | November 1, 2016, through January 31, 2017 | Demographics, Economic, Patient access, Social | No |
| Bcheraoui 2015 (12) | This study aims to investigate knowledge and practices of breast cancer screening among Saudi women aged 50 years or older in order to inform the breast cancer national health programs. | Cross-sectional study | Journal article | Saudi Arabia | The Saudi Health Interview Survey | National survey | Between April and June 2013 | Demographics, Social | No |
| Beaber 2019 (13) | To evaluate multilevel predictors of nonadherence among screened women, as these are not well known. | Prospective cohort study | Journal article | USA | National Cancer Institute-funded consortium Population-based Research Optimizing Screening through Personalized Regimens (PROSPR) (Obtained breast imaging data (imaging date, mammogram type [standard two-dimensional and/or digital breast tomosynthesis], laterality, indication for examination, BIRADS,) | Multicenter | Jan 2011- Sep 2014 | Demographics, Economic, Patient access, PCP access | No |
| Berens 2014 (14) | To investigate for the first time participation among women of Turkish origin in Germany. | Retrospective cohort study | Journal article | Germany | Screening center or population-based registries | Multicenter | 2010 to 2011 | Demographics, Patient access | No |
| Bhandari 2021 (15) | This study identified breast cancer screening behavior and factors associated with breast cancer screening intention among women in Kathmandu Valley, Nepal. | Cross-sectional study | Journal article | Nepal | Nepalese women residing in Kathmandu Valley, Nepal | Multicenter | July to September 2018 | Demographics, Patient access, Patient behavior | No |
| Cataneo 2020 (16) | NR | Retrospective cohort study | Conference abstract | USA | 2015 sample of the National Health Interview Survey (NHIS) database | Nationwide study | NR | Demographics, Economic, Patient access | No |
| Chan 2014 (17) | The objective of this population-based study was to compare receipt of a screening mammogram in Ontario between screen-eligible women with diabetes and age-matched women without diabetes, and to determine the influence of socio-economic status on this relationship. | Retrospective cohort study | Journal article | Canada | The study used linked, population-based healthcare databases, which record data on all Ontario residents covered by the universal provincial healthcare plan. The study obtained information on demographics and deaths from the Registered Persons Database.  Mammogram records were obtained from the Ontario Health Insurance Plan database | Nationwide Population-based cohort study | January 1999 to 31 December 2010. | Demographics, Economic | No |
| Choi 2015 (18) | To examine the stages of adoption for breast cancer screening to outline the extents to which breast cancer worry affect the decisions of Korean women to undergo breast cancer screening or not. | Cross-sectional study | Journal article | Korea | Korean National Cancer Screening Survey (KNCSS) 2013 | Nationwide study | Sep 26 to Octo 18, 2013 | Demographics, Economic, Patient access, Patient behavior | No |
| Choi 2017 (19) | The present study addresses the following research questions: ● What is the prevalence of breast cancer screening behaviors (i.e., clinical breast examination and mammogram) for Korean American women? ● What are some reasons for not receiving breast cancer screening services for Korean American women? ● What factors affect the use of breast cancer screening services for Korean American women? Particularly, do sociocultural factors (i.e., access to health care and cultural beliefs) influence the use of breast cancer screening services for Korean American women? | Cross-sectional study | Journal article | USA | NR | Community based survey | May 2012 to August 2012 | Demographics, Patient access, Patient behavior, Social | No |
| Dahlui 2012 (20) | To determine the level of knowledge of breast cancer and the practice of breast cancer screening among women at the sub-urban area. Factors associated with the practice of breast cancer screening were also investigated. | Cross sectional study | Journal article | Malaysia | NR | Community based survey | 0 | Demographics, Economic, Patient access, Patient behavior Social | No |
| Donnelly 2012 (21) | To investigate within the State of Qatar Arabic women’s knowledge regarding breast cancer and breast cancer screening (BCS) methods and their participation rates in BCS. This paper reports on the results of a cross-sectional survey | Quantitative, cross-sectional interview survey | Journal article | Qatar | NR | Nationwide study | March 2011 to July 2011 | Demographics, Economic, Patient access, PCP access, Social | Yes |
| Donnelly 2013 (22) | To investigate beliefs, attitudes, and BCS practices of Arabic-speaking women in Qatar | Cross-sectional quantitative survey | Journal article | Qatar | NR | Multicenter | Mar 2011 to Jul 2011 | Demographics, Patient behavior, PCP access, Social | No |
| Donnelly 2015 (23) | This study explores the influence of socioeconomic status on BCS among Arab women in Qatar. | Cross-sectional quantitative survey | Journal article | Qatar | NR | Multicenter | March 2011 to July 2011 | Demographics, Economic, Social | No |
| Duport 2012 (24) | This study aimed at (i) estimating the 2-year self-reported breast cancer screening coverage rate; and (ii) analyzing the relationships between sociodemographic characteristics and healthcare access of women and breast cancer screening (opportunistic or organized) practices | Cross-sectional study | Journal article | France | 2006 ‘French Health, Health Care and Insurance Survey’’ (ESPS) | National survey | 2006 | Demographics, Economic, Patient access, Patient behavior, PCP access, Social | No |
| Eichholzer 2016 (25) | To examine the relationship between BMI (kg/m2) and the use of mammography in Switzerland as well as separately in the German-speaking part with mainly opportunistic screening and in the French-speaking part with organized programs | Cross-sectional study | Journal article | Switzerland | Swiss health survey | Nationwide study | 2012 | Demographics | No |
| El Mhamdi 2013 (26) | The objective of this study was to assess the knowledge, attitude and practice of BCS among women and health care professionals in the region of Monastir. | Cross-sectional descriptive study | Journal article | Tunisia | NR | Multicenter | 1 March 2009 to 30 June 2009 | Demographics, Patient access, Patient behavior, Social | No |
| Elobaid 2014 (27) | The aim of this study was to assess breast cancer screening knowledge, attitudes and practices among women of screening age ($40 years old) in the city of Al Ain, United Arab Emirates (UAE). | Cross-sectional study | Journal article | UAE | 2013 Breast Cancer Awareness Measure (CAM) | Multicenter | 41334 | Demographics, PCP access, Social | No |
| Ezema 2021 (28) | This study evaluated the association of BC fear and perceived self-efficacy with BC screening (clinical breast exam [CBE] and mammography) among middle-aged Nigerian women | Cross-sectional study | Journal article | Nigeria | Middle-aged women in Enugu State, southeast Nigeria. | State wide study | September 2019 and February 2020. | Demographics, Economic, Patient behavior, Social | No |
| Farid 2014 (29) | To determine the prevalence of breast cancer screening, specifically on clinical breast examination, and the predictors of its uptake among women in Malaysia | Cross-sectional study | Journal article | Malaysia | NR | State wide study | Feb, 2014 | Demographics, Economic, Patient access, PCP access, Social | Yes |
| Fayanju 2014 (30) | To investigate perceived barriers to mammography among underserved women, we asked participants in the Siteman Cancer Center Mammography Outreach Registry – developed in 2006 to evaluate mobile mammography's effectiveness among the underserved – why they believed women did not get mammograms | Prospective cohort study | Journal article | USA | NR | Multicenter | Apr 2006- May 2011 | Demographics, Economic, Patient access | No |
| Fleming 2013 (31) | To determine why women skip rounds and factors influencing return of previous non-attenders (PNAs) to breast screening. | Retrospective, quantitative, structured questionnaire, case control study | Journal article | Ireland | BreastCheck database | Nationwide study | Screening appointment in 2007/2008 but then attended in 2010 | Patient behavior | No |
| Gan 2018 (32) | The study aims to evaluate breast cancer screening behaviors and to identify the predictors of insufficient knowledge and attitudes towards breast cancer and its screening among female residents | Cross-sectional study | Journal article | China | NR | Region wide study | April to June 2016 | Demographics, Economic, Patient access, Patient behavior, Social | No |
| Gang 2013 (33) | To explore the factors associated with adherence to guidelines for regular mammography among Chinese married women. | Cross-sectional study | Journal article | China | NR | City wide study | NR | Demographics, Economic, Patient behavior, Social | No |
| Gilfoyle 2019 (34) | To explore whether perceived susceptibility (PS) – whether an individual feels they are personally vulnerable to a health-related condition or disease – a core component of the Health Belief Model (HBM), influences individual screening behaviors for a variety of cancers (breast, prostate, and colorectal cancer) | Cross sectional study | Journal article | Canada | NR | Province based survey | 2000-2008 | Demographics, Economic, Patient behavior, Social | No |
| Gong 2023 (35) | In this study, we conducted a national survey to investigate which factors influence the likelihood of these disadvantages preventing women from attending mammography screening appointments in the Netherlands. | Cross-sectional study | Article in Press | Netherlands | NR | National survey | 2017 | Demographics, Patient access, PCP access, Social | No |
| Guilcher 2014 (36) | To address the absence in the literature of studies examining both level of disability and level of morbidity in relation to mammography | Retrospective population-based cohort study | Journal article | Canada | Clinical Evaluative Sciences (ICES) in Ontario, Canada, including the 2005 and 2007/2008 Canadian Community Health Surveys (CCHS), Ontario Health Insurance Plan (OHIP) | Nationwide study | 2005 and 2007/2008 | Demographics, Economic, Social | No |
| Hajian-Tilaki 2014 (37) | The objective of this study is to determine the role of different health belief model components in practice of breast cancer screening among Iranian women | Cross-sectional study | Journal article | Iran | Standard health belief model questionnaire was used to assess women aged 18–64 years in an urban population under the coverage of health therapeutic centers in Babol, located to the south of the Caspian Sea, in the north of Iran, in 2012 | State wide survey | 2012 | Patient behavior | No |
| Hajian-Tilaki 2015 (38) | The objective of this study was investigation the awareness, attitude and practice of breast cancer screening women. | Cross-sectional study | Journal article | Iran | NR | Multicenter | 2012 | Demographics, Economic, Patient access, Patient behavior, Social | No |
| Hassan 2017 (39) | To assess the level of knowledge about breast cancer risk factors, early warning signs, screening approaches and related predictors | Cross-sectional study | Journal article | Egypt | NR | Village wide study | Feby- May 2016 | Demographics, Economic, Patient access, Social | No |
| Hasnain 2014 (40) | To identify factors that influence Muslim women’s decision making to engage in breast cancer screening, our study had the following three primary objectives: 1. Develop a culturally relevant survey to assess screening practices and to identify factors associated with mammography use by Muslim women. 2. Confirm psychometric properties of survey subscales in differing languages. 3. Explore the associations between mammography use and predisposing, enabling, and need variables. | Cross-sectional study design | Journal article | USA | NR | Community based survey | 2009-2010 | Demographics, Economic, Patient access, Patient behavior, Social | No |
| Hippman 2016 (41) | To evaluate MMG uptake by women attending the AWHC and to explore knowledge and beliefs about breast cancer and MMG screening as well as predictors (barriers and facilitators) of MMG for this population. | Cross-sectional study | Journal article | Canada | NR | Single center | Jan- Apr 2011 | Demographics, Patient access, Patient behavior, PCP access, Social | No |
| Hsieh 2021 (42) | To assess women’s health knowledge and awareness regarding breast cancer and screening programs, and their perceived HBM constructs and the uptake of biannual mammography, while controlling for demographic and breast cancer risk factors and health behaviors. | Cross-sectional study | Journal article | Taiwan | NR | Multicenter | Jan 2018 to Jul 2018 | Demographics, Patient access, Patient behavior, Social | No |
| Jadav 2015 (43) | To identify and quantify individual-level sociodemographic and health-related factors that contribute to racial-ethnic disparities in breast cancer screening using the nonlinear Blinder-Oaxaca decomposition method. | Retrospective pooled cross-sectional study | Journal article | USA | Medical Expenditure Panel Survey (MEPS) | Nationwide study | 2000 to 2010 | Demographics, Economic, Patient access, Social | Yes |
| Jin 2019c (44) | To examine: 1) rates of mammography screening among a sample of KA women; and 2) factors associated with having a mammogram within the past year among KA women | Cross sectional study | Journal article | USA | NR | City-wide survey | May 2015 to February 2016 | Demographics, Economic, Patient access, Patient behavior, PCP access, Social | No |
| Jin 2021 (45) | The present study employed the Precaution Adoption Process Model (PAPM) as a theoretical framework to examine characteristics and investigate factors associated with the decisional stage for mammography adoption. The research questions of this study included (a) how do characteristics of decisional stage for mammography adoption differ by stages? and (b) what factors distinguish individuals who have decided to uptake mammography from those who have not yet decided to uptake mammography? The findings of this study provide implications for interventions and practice aimed at facilitating mammography adoption among Korean American women. | Cross-sectional study | Journal article | USA | NR | Community based, city-wide survey | May 2015 to February 2016 | Demographics, Patient access, Patient behavior, PCP access | Yes |
| Kadaoui 2012 (46) | To describe physician practices with regard to opportunistic screening for breast cancer in women aged 35 to 49 years and 70 years of age and older, and to identify the determinants associated with the practice of prescribing screening mammography | Cross-sectional study | Journal article | Canada | NR | City wide general practitioners based study | 2009 | Demographics, Patient access, Patient behavior, PCP access, Social | No |
| Kangmennaang 2019 (47) | Breast cancer contributes substantially to morbidity and mortality in Namibia as is the case in most countries in Sub-Saharan Africa (SSA). However, there is a dearth of nationally representative studies that examine the odds of screening for breast cancer in Namibia and SSA at large. This paper aims to fill this gap by examining the determinants of breast cancer screening guided by the Health Belief Model. | Cross-sectional study | Journal article | Namibia | Namibia Demography and Health Survey (NDHS) [face-to-face questionnaire interviews] | Nationwide study | 2013 | Demographics, Economic, Patient access, PCP access, Social | No |
| Kardan-Souraki 2019 (48) | To investigate the factors that affect the screening of women for breast cancer in the northern part of Iran. | Cross-sectional study | Journal article | Iran | NR | Providence based survey | 2016 | Demographics, Economic, Patient behavior, Social | No |
| Khaliq 2015 (49) | The purpose of study was to explore and quantify the socio-demographic and clinical variables associated with non-adherence to breast cancer screening among hospitalized women. We hypothesized that a combination of socio-demographic variables and comorbidities would best explain the association of non-adherence with breast cancer screening. | Cross-sectional study | Journal article | USA | General medical service at Johns Hopkins Bayview Medical Center | Single center | October 2011 - January 2012 | Demographics, Economic, Patient access, Patient behavior, PCP access Social | No |
| Kim 2014 (50) | To identify knowledge, health beliefs, and behaviors related to breast cancer screening in immigrant women living in Korea, as well as to provide a starting point for further systematic investigation | Descriptive, cross-sectional study | Journal article | Korea | NR | National survey | Between March and July 2012 | Demographics, Economic, Social | No |
| Kim 2022 (51) | To explore the association between diabetes status and mammography screening rates. In recognition of the racial disparities that exist for both diabetes prevalence and breast cancer outcomes, analyses will further assess whether this association varies between racial groups, ethnic groups, and across geographic regions. | Cross-sectional study | Journal article | USA | Nationally representative data from Behavioral Risk Factor Surveillance System (BRFSS), and a national telephone survey that collects data on health behaviors, chronic conditions. | National survey | 2012, 2014, 2016, and 2018 | Demographics | No |
| Kirag 2019 (52) | To examine the projected risk of breast cancer in Turkish women academician, determine the levels of their breast cancer screening behaviors and uncover the relationship between their health beliefs and screening behaviors | Cross-sectional descriptive study | Journal article | Turkey | NR | Province wide study | March to July 2018 | Demographics, Patient access, Patient behavior, Social | No |
| Kırca 2018 (53) | To evaluate breast cancer screening behaviors of women who are first-degree relatives of women with breast cancer and factors affecting these behaviors. | Cross-sectional study | Journal article | Turkey | NR | Single center | March and May, 2014 | Demographics, Economic, Patient behavior, Social | No |
| Kiyang 2015 (54) | To assess the intention of family physicians to provide women with this support and the determinants of this intention, and to identify factors that might influence family physicians adopting this behavior | A cross-sectional quantitative survey | Journal article | Canada | NR | Providence based survey | 2010 | Patient behavior, Social | No |
| Kosog 2020 (55) | To identity an association between sociodemographic factors and breast cancer screening adherence in FQHC patients that included the homeless | Exploratory, cross-sectional, retrospective study | Journal article | USA | Encounter level data from a FQHC in a major metropolitan (Chicago, IL) area | City-wide survey | January 1st, 2017 through December 31st, 2018. | Demographics, Patient access | No |
| Kriaucioniene 2019 (56) | To determine the trend in the attendance for mammography screening during 2006–2014 and to identify the factors that are predictive for participation in it. | Cross-sectional biennial postal surveys | Journal article | Lithuania | National Population Register, National breast cancer screening program, National cervical cancer screening program | Nationwide study | 2006 to 2014 | Demographics, Economic, Patient behavior, PCP access, Social | No |
| Kwok 2014 (57) | This study aimed to report breast cancer screening practices among Hong Kong Chinese women and to examine the relationship between (1) demographic factors and (2) the modified Chinese Breast Cancer Screening Beliefs Questionnaire (CBCSB) score and women’s breast screening behaviors. | Cross-sectional study | Journal article | China | NR | Region wide study | May-October 2008 | Demographic, Economic, Social | No |
| Kwok 2016 (58) | To report breast cancer screening practices among Arabic women in Australia and to examine the relationship between (1) demographic factors and (2) the Arabic version of the Breast Cancer Screening Beliefs Questionnaire (BCSBQ) score and women’s breast screening behaviors. | Cross-sectional study | Journal article | Australia | The Breast Cancer Screening Beliefs Questionnaire (BCSBQ) score, the Arabic version of the BCSBQ | Community wide study | June to December 2014. | Demographics, Economic, Social | No |
| Kwok 2022 (59) | To examine the role of educational levels and compare the cultural beliefs associated with breast cancer screening practices among immigrant Korean women in Australia with those of their counterparts in Korea. | Cross-sectional study | Journal article | Australia, Korea | NR | International wide study | 2015 in Australia, 2017 in Korea | Demographics, Economic, Social | No |
| Lam 2018 (60) | To evaluate breast cancer screening (BCS) practice and explore the relationship between sociodemographic factors and breast awareness (BA), clinical breast examination (CBE) and mammography in migrant-Australian women. | Cross-sectional study | Journal article | Australia | NR | Community based survey, Secondary analysis | Between January 2009 and March 2016 | Demographics, Economic, Social | No |
| Lawson 2021 (61) | To determine factors associated with receipt of screening mammography by insured women before breast cancer diagnosis, and subsequent outcomes. | Retrospective cohort study | Journal article | USA | Using claims data from commercial and federal payers linked to a regional SEER registry | State wide study | Jan 1, 2007- Dec 31, 2017 | Demographics, Economic, Patient behavior | No |
| Lee 2017 (62) | This study investigated breast cancer screening rates and its associated factors in Korean American immigrant women using three breast cancer screening methods such as mammogram, Clinical Breast Examination (CBE), and Breast Self-Examination (BSE) | Cross-sectional study | Journal article | USA | NR | Community based survey | NR | Demographics, Patient access, Patient behavior, Social | No |
| Lee 2018c (63) | To (a) describe BC screening utilization and preventive lifestyle behaviors and (b) examine factors associated with BC utilization among Korean American (KA) women. | Cross-sectional study | Journal article | USA | California Health Interview Survey | State wide study | 2015 to 2016 | Demographics, Patient access | No |
| Lemogne_2018 (64) | To examine whether mammography use would be influenced by Type A personality and various facets of hostility in a large prospective cohort of women. | Prospective observational cohort study | Journal article | France | GAZEL cohort study | Nationwide study | NR | Demographics, Patient behavior, PCP access | No |
| Leung 2014 (65) | To describe longitudinal patterns of mammogram service use, CBE, and BSE, and how these change over time. A further aim was to examine whether these behaviors differ by geographical area of residence. | Prospective study | Journal article | Australia | Women were selected from the Australian national health insurance database (Medicare). Data were drawn from the Australian Longitudinal Study on Women’s Health | Nationwide study | 2001, 2004, 2007, 2010 | Demographics, Economic, Patient access, Patient behavior, Social | No |
| Llaneza 2022 (66) | To evaluate differences in yearly mammogram screening by smoking status in a sample of US women. We also examined differences in mammogram screening by race/ethnicity, age, and health care coverage. | Retrospective, cross-sectional population study | Journal article | USA | 2018 Health of Houston Survey study | City wide study | Jun 2017- Feb 2018 | Demographics, Patient access, Patient behavior | No |
| Lopez 2020 (67) | To evaluate mammography screening engagement in U.S. territories compared with all of the U.S. states. | Retrospective analysis of previously collected cross sectional survey data | Journal article | US territories and USA | Behavioral Risk Factor Surveillance System (BRFSS) | Nationwide study | 2016 | Demographics, Economic, Patient access, Social | No |
| Ma 2012 (68) | To apply a Sociocultural Health Behavior Model to determine the association of factors proposed in the model with breast cancer screening behaviors among Asian American women. | Cross sectional study | Journal article | USA | NR | Community based survey | NR | Demographics, Patient access, Patient behavior, PCP access, Social | No |
| Martín-López 2013 (69) | The aim of this study is to estimate the adherence to recommended preventive practices for breast cancer (mammography) in Spain and to identify predictors of uptake according to sociodemographic variables, health related variables and lifestyles. | Cross-sectional study | Journal article | Spain | European Health Interview Survey for Spain | Nationwide study | 2009-2010 | Demographics, Economic, Patient behavior, PCP access, Social | No |
| Miles 2019 (70) | To assess the current association between increasing BMI and use of mammography screening | Cross-sectional study | Journal article | USA | 2016 Behavioral Risk Factor Surveillance System | State-based national survey | 2016 | Demographics | No |
| Mohan 2021 (71) | This study aimed to identify the practice of BSE and BC screening and assess the barriers to BC detection among women aged 40 years and above from different ethnic backgrounds in a semi-rural setting in Malaysia. | Cross-sectional study | Journal article | Malaysia | Health and demographic surveillance site (HDSS) | Multi district wide study | June - September 2017 | Demographic, Economic, Social | No |
| Morere 2019 (72) | To gain clearer insight into the characteristics of women who have had at least one screening examination but have not returned after the recommended two-year interval for a repeat mammography. | Cross-sectional study | Journal article | France | French nationwide observational survey EDIFICE 6 | Nationwide study | 26 Jun- 28 Jul 2017 | Patient behavior, Social | No |
| Mukem 2014 (73) | To determine uptake rates of breast cancer screening including breast self-examination (BSE), clinical breast examination (CBE), and mammography screening, and to identify enabling factors and barriers associated with screening uptake. | Cross sectional study | Journal article | Thailand | Two population-based household surveys, the 2009 Reproductive Health Survey (RHS) and the 2007 Health and Welfare Survey (HWS). | National survey | 2009 and 2007 | Demographics, Economic, Patient access, Social | No |
| Nandam 2018 (74) | To evaluate mammography rates in women with CP and to identify strengths and barriers with their screening experience. | Cross-sectional study | Journal article | USA | NR | Multicenter | 2015 to 2016 | Demographics | No |
| Ngan 2022 (75) | To examine current breast cancer (BC) screening practices among Vietnamese women and the factors associated with the uptake of clinical breast examination (CBE) | Cross sectional study | Journal article | Vietnam | NR | City-wide survey | August, 2019 | Demographics, Economic, Patient access, Patient behavior, Social | No |
| Nojomi 2014 (76) | To identify the factors associated with breast cancer screening (BCS) and with an intention to have screening among women in Tehran, Iran | Cross-sectional study | Journal article | Iran | NR | Multicenter | January to July 2011 | Demographics, Patient access, Patient behavior, Social | No |
| O’Hara 2018 (77) | To explore if women from CALD backgrounds with lower health literacy reported greater emotional, knowledge, or structural barriers that may inhibit their participation in breast cancer screening. A further aim was to identify if women from CALD backgrounds with lower health literacy were more likely to report under-screening than women with higher health literacy | Cross-sectional study | Journal article | Australia | NR | City-wide | March 2016 to September 2016 | Demographics, Patient access, Patient behavior, Social | No |
| Ogunsiji 2017 (78) | To report breast cancer screening status among African migrant women in Australia; to investigate the association between demographic factors and their breast cancer screening practices and also to investigate the relationship between these women’s cultural beliefs and their breast cancer screening practices | Cross-sectional descriptive study | Journal article | Australia | NR | City wide study | Oct 2013 to Dec 2014 | Demographics, Economic, Social | Yes |
| Okui 2021 (79) | To investigate predictors for participation in breast cancer screening and analyzed the trend of participation rate depending on the predictors using nationally representative survey data in Japan | Cross-sectional study | Journal article | Japan | Comprehensive Survey of Living Conditions | National survey | 2001-2013 | Demographics, Economic, Patient behavior, PCP access, Social | No |
| Pagán 2012 (80) | This study analyzes the role of functional health literacy on mammography screening behavior and adherence of Hispanic women. | Prospective observational study | Journal article | USA | NR | State wide study | 2008 | Demographic, Economic, Patient access, Social | No |
| Paranjpe 2022 (81) | To determine whether breast cancer screening practices were different between Asian and NHW women in a national population-based study | Cross-sectional study | Journal article | USA | The 2015 National Health Interview Survey (NHIS) | National survey | 2015 | Demographics, Economic, Patient access, Social | No |
| Park 2020 (82) | To assess the relationship between women's depression and mammography adherence. | Cross-sectional study | Journal article | USA | behavioral Risk Factor Surveillance System and employed the Health Belief Model (HBM) | Nationwide study | 2016 | Demographics, Economic, Patient access, Patient behavior, Social | No |
| Patel 2014 (83) | The current study examines socio-demographic factors that influence decisions to use mammography and other breast cancer screenings in low-income African Americans. In addition, this study examines the differences in obstacles to screening by geographic region. | Cross sectional study | Journal article | USA | Meharry CNP community survey database | Community survey | 2005 | Demographics, Economic, Patient access, Patient behavior, PCP access, Social | No |
| Racine 2022 (84) | To further explore the Muslim Syrian refugee women’s breast self-examination (BSE), utilization of clinical breast examination (CBE) and mammography. | Cross-sectional study | Journal article | Canada | NR | Province wide study | Jul to Dec 2018 | Demographics, Patient access, Patient behavior, Social | No |
| Radhakrishnan 2018 (85) | To investigate broad range of attitudes and beliefs towards mammography screening, using factor analysis to group them into underlying themes, to investigate whether these themes varied according to which guidelines physicians trusted the most and physician specialty and to examine whether attitudes and beliefs were associated with physician breast cancer screening recommendations for women of different age groups | Cross sectional study | Journal article | USA | Breast Cancer Social Networks study- a national survey of primary care physicians. | National survey | Between May and September 2016 | Demographics, Patient access, Patient behavior, Social | No |
| Rollet 2021 (86) | To evaluate socio-territorial inequities in the FNBCSP 2013–2014 campaign by studying individual and contextual factors in a single model, in a large sample of the eligible population residing in 41 départements of metropolitan France. | Prospective observational study | Journal article | France | FNBCSP campaign | Multicenter | 2013–2014 | Demographics, Patient access, Social | No |
| Rondet 2013 (87) | To investigate the association between social contact and social support and women's breast cancer screening (BCS) practices, taking their socioeconomic status (SES) into account | Cross-sectional study | Journal article | France | SIRS (French acronym for Health, Inequalities and Social Ruptures) survey | City wide study | 2010 | Demographics, Economic, Patient access, Social | No |
| Ross 2020 (88) | The aims of this study are to: (1) quantify the relationship between self-reported chronic poor mental health and breast screening uptake in the UK. (2) examine the extent to which self-reported chronic poor mental health explains the lower breast screening uptake observed in women who are not married, those of lower socio-economic status, and those living in large conurbations. | Cohort study | Journal article | UK | 2011 Census records within the Northern Ireland Longitudinal Study | National study | April 2011 and March 2014 | Demographics, Patient access, Social | No |
| Ross 2022 (89) | This study examines the association between practice-level decision aid use and mammography use among older women. This analysis fills a critical gap in evidence by analyzing a national sample of physician practices and claims data from eligible, attributed Medicare fee-for-service (FFS) beneficiaries | Cohort study | Journal article | USA | National Survey of Healthcare Organizations and Systems were linked to 2016 and 2017 Medicare fee-for-service beneficiary data from eligible beneficiaries | National survey | 2017/2018 | Demographics, Economic, Patient access, PCP access | No |
| Sabgul 2021 (90) | To investigate the potential effect of husbands’ knowledge on their wives’ attitudes and practices related to breast cancer screening in the holy city of Makkah in the Makkah region of western Saudi Arabia, located 70 kilometers inland from Jeddah | Cross-sectional study | Journal article | Saudi Arabia | NR | City-wide survey | May 6 to July 7, 2020 | Demographics, Economic, Patient access, Social | No |
| Satoh 2021 (91) | This study examined the relationships between tendencies in decision-making under conditions of uncertainty, health behaviors, demographics, and breast cancer screening participation in Japanese women | Cross-sectional study | Journal article | Japan | Keio Household Panel Survey (KHPS) | Nationwide study | 2017 | Demographics, Economic, Patient behavior, Social | No |
| Sheppard 2015 (92) | To examine factors that are associated with higher endorsement of screening | Cross-sectional study | Journal article | USA | NR | Community wide study | NR | Demographics, Patient access | No |
| Shin 2020a (93) | The objective of the current study was to investigate: 1) whether disparities exist in breast cancer screening rates among women with disabilities compared with women without disabilities; 2) whether breast cancer screening rates vary according to the type and severity of disabilities; and 3) trends in breast cancer screening disparities according to disability status over time. | Retrospective cohort study | Journal article | South Korea | National Health Information Database (NHID) | Nationwide study | 2006-2015 | Demographics, Economic | Yes |
| Solikhah 2018 (94) | To investigate knowledge, barriers, and behaviors of women regarding breast cancer screening among rural and urban Indonesian women. | Cross-sectional study | Journal article | Indonesia | NR | Multicenter | Mar to May 2016 | Demographics, Economic, Patient access, Patient behavior, Social | No |
| Solikhah 2019 (95) | To comprehensively investigate the Indonesian women’s level of knowledge about breast cancer risk factors, barriers, attitude and breast cancer screening. | Cross-sectional population based study | Journal article | Indonesia | Self-administered questionnaire | Multi provinces based study | March to May 2016 | Demographics, Economic, Social | No |
| Son 2017 (96) | To examine the association between shift work types and participation in breast cancer screening (BCS) programs by comparing rates of participation for BCS among regular daytime workers and alternative shift workers using data from a nationally representative, population-based survey conducted in Korea | Cross-sectional study | Journal article | Korea | Korean National Health and Nutritional Examination Survey 2012 | National survey | 2012 | Demographics, Economic, Patient access, Social | No |
| Subramanian 2013 (97) | This study aimed to assess the knowledge of breast cancer risk factors and early detection measures among women in a high risk group | Cross-sectional study | Journal article | Malaysia | Breast Clinic and Oncology Clinic of University Malaya Medical Centre, Kuala Lumpur. | Single center | NR | Demographics, Economic, Patient access, Social | No |
| Tapera 2019a (98) | This study explored the Health Belief Model (HBM) constructs in explaining factors associated with breast cancer screening amongst female students in Botswana. | Cross sectional study | Journal article | Botswana | NR | Single center | January 2017 and April 2017 | Patient behavior | No |
| Tran 2021 (99) | To assess degrees of perceived risk and cancer worry for breast cancer according to knowledge and awareness of one’s own breast density status among Korean women and to investigate how awareness and knowledge of breast density could be related to intentions to undergo mammography screening | Cross sectional study | Journal article | Korea | Korean National Cancer Screening Survey | National survey | August 26 to September 29, 2017. | Demographics, Economic, Patient access, Patient behavior, Social | No |
| Tsunematsu 2013 (100) | To identify psychological and personal characteristics of women concerning their participation in breast cancer screening using the Health Belief Model (HBM) | Cross-sectional study | Journal article | Japan | NR | Town-wide survey | As of April 1 2012 | Demographics, Patient behavior | No |
| Vang 2022 (101) | To examine the relationship between language preference and screening mammogram adherence in medically underserved women in New York City | Cross-sectional study | Journal article | USA | NR | Community wide study | Feb 2018- Feb 2019 | Demographics, Patient access, Social | No |
| Wang 2022 (102) | To (i) investigate the practices of Chinese-Australian women living in Sydney in relation to the main breast cancer screening tests (CBE and mammography); and (ii) identify any factors associated with their breast cancer screening behaviors | Cross-sectional quantitative survey | Journal article | Australia | NR | City-wide survey | July to November 2016. | Demographics, Economic, Patient behavior, Social | No |
| Warner 2019 (103) | This study examines the interplay between complex multimorbidity, age, and subjective assessments of health and longevity for screening mammography receipt. | Cross-sectional study | Journal article | USA | U.S. Health and Retirement Study (HRS) | Nationwide study | 2012 | Demographics, Economic, Patient access, Patient behavior, Social | No |
| Wilson 2014 (104) | To examine whether having sick leave is associated with increased breast cancer screening among female employees. | Cross-sectional study | Journal article | USA | 2006–10 Medical Expenditure Panel Survey | Nationwide study | 2006-2010 | Demographics, Economic, Patient access, Social | No |
| Yilmaz 2013 (105) | To determine the health belief perceptions of breast cancer screening programs among residential women | Cross-sectional study | Journal article | Turkey | NR | Community based survey | Between May 30 and October 15, 2011 | Demographics, Economic, Patient access, Patient behavior, Social | No |
| Zhang 2012 (106) | To examine associations between baseline worries of developing breast cancer and screening behaviors at one-year follow up for these women with at least one first-degree relative diagnosed with breast and/or ovarian cancer. | Prospective cohort study | Journal article | Canada | Ontario site of the Breast Cancer Family Registry (BCFR) | Provence wide study | Nov 2005- Mar 2007 | Patient behavior | No |
| **Cervical cancer** | | | | | | | | | |
| Abdullah 2016 (107) | The objectives of this study were to determine the prevalence and predictors of Pap smear screening among women aged 50 years and older | Cross-sectional study | Journal article | Malaysia | NR | District | Between February and April 2014 | Demographics, Economic, Patient behavior, PCP access, Social | No |
| Abebaw 2022 (108) | To assess knowledge, attitudes, and practices of cervical cancer screening and predictors among female healthcare providers. | Cross-sectional study | Journal article | Ethiopia | NR | Multicenter | 4 March to 20 April 2019 | Patient behavior, PCP access | No |
| Abera 2020 (109) | This study focused on assessment of the demand of adult women for cervical cancer screening using the VIA method in the Tigray region. It also provides information about predictors of demand for cervical cancer screening. | Cross-sectional study | Journal article | Ethiopia | NR | Community wide study | Jan to Apr 2019 | Demographics, Economic, Patient access, Patient behavior, PCP access, Social | Yes |
| Agboola 2021 (110) | To identify determinants of knowledge of CC, attitude towards cervical cancer screening (CCS) and practice of CCP among antenatal attendees in a tertiary hospital in Southwest Nigeria as they are sexually active women. | Cross-sectional study | Journal article | Nigeria | NR | Single center | NR | Demographics, Economic, Patient access, Patient behavior, PCP access, Social | No |
| Agénor 2014 (111) | To investigate how one dimension of sexual orientation, sex of sexual partners, and race/ethnicity jointly influence Pap test use among black, Latina and white U.S. women aged 21e44 years | Cross-sectional study | Journal article | USA | 2006-2010 National Survey of Family Growth (NSFG) | Nationwide study | 2006-2010 | Demographics, Patient access, Patient behavior, Social | No |
| Akinlotan 2017 (112) | This study aimed to assess correlates of cervical cancer risk factor knowledge and examine socio-demographic predictors of self-reported barriers to screening among a group of low-income uninsured women. | Cross-sectional study | Journal article | USA | Cancer Prevention and Research Institute of Texas (CPRIT), | State wide study | NR | Demographics, Patient access, Social | No |
| Akinlotan 2018 (113) | To examines the combined effect of individual and county-level characteristics on the use of cervical cancer screening tests such as Papanicolaou (Pap) tests in Texas. | Cross-sectional study | Journal article | USA | Texas Behavioral Risk Factor Surveillance System (BRFSS) Telephone based survey | State wide study | 2014 to 2015 | Demographic, Economic, Patient access, PCP access, Social | No |
| Aktaş 2021 (114) | To examine OBGYNs’(Obstetrician-gynecologists) adherence to guidelines in the management of women with HPV-positive test results. | Cross-sectional study | Journal article | Turkey | NR | Province wide study | May to Sept 2018 | PCP access | No |
| Al-Amro 2020 (115) | The aim of this study was to determine the factors associated with cervical cancer screening uptake among Jordanian women. | Cross-sectional study | Journal article | Jordan | NR | Community based survey | October and December 2013 | Demographics, Economic, Patient access, PCP access | No |
| Amin 2020 (116) | This study aimed to investigate the disparities in cervical cancer screening participation in Iran. | Cross-sectional study | Journal article | Iran | Non-Communicable Risk Factors Survey in 2016 (STEPs 2016). | Nationwide study | 2016 | Demographics, Economic, Patient access, Social | No |
| Aminisani 2012 (117) | To evaluate participation in cervical screening by Middle Eastern and Asian migrant women in Australia in comparison with that in Australian born women, taking into account potential confounding effects of socioeconomic status. | Cross-sectional study | Journal article | Australia | De-identified data from the NSW Midwives Data Collection (MDC) and from the NSW Admitted Patients Data Collection (APDC) ; each of these data collections includes country of birth. Each was linked separately to the NSW Pap Test Register (PTR) through the NSW Centre for Health Record Linkage (CHeReL) | Single center | 1996–2006 | Demographics, Economic, Patient behavior, Social | No |
| Aminisani 2016 (118) | To identify which women characteristics are potentially associated with and may have an important influence on the uptake of cervical cancer screening in Kurdish women living in the west of Iran | Cross-sectional study | Journal article | Iran | NR | City wide study | 2014 | Demographics, Patient access, Patient behavior, Social | No |
| Ararsa 2021 (119) | To assess knowledge towards cervical cancer screening and associated factors among urban health extension workers in Addis Ababa, Ethiopia, 2020. | Cross-sectional study | Journal article | Ethiopia | NR | City wide study | February to May 2020 | Demographics, Economic, Patient behavior, Social | No |
| Aredo 2021 (120) | To assess the knowledge of cervical cancer screening and its associated factors among women attending maternal health services at Aira hospital, West Wollega, Ethiopia | Cross-sectional quantitative study | Journal article | Ethiopia | NR | City-wide survey | 7 February to 30 March 2018 | Demographics, Economic | No |
| Arulogun 2012 (121) | To assess and document the perception and utilization of cervical cancer screening services among female nurses of the University College Hospital, Ibadan, Nigeria. | Descriptive cross-sectional study | Journal article | Nigeria | NR | Single center | NR | PCP access | No |
| Assefa 2019 (122) | To assess the utilization of CCS and associated factors among HIV positive women in public health facilities, Hawassa town. | Cross-sectional study | Journal article | Ethiopia | NR | City wide study | Mar 2 - Apr1 /2019 | Demographics, Economic, Patient access, Patient behavior, Social | Yes |
| Atnafu 2021 (123) | To assess women’s satisfaction with cervical cancer screening services and factors associated with it in public health facilities of Jimma town, Southwest Ethiopia. | Cross-sectional study | Journal article | Ethiopia | NR | Multicenter | 20 Mar to 20 May 2020 | Demographics, Patient access, Patient behavior, PCP access, Social | No |
| Ayanore 2020 (124) | To examine breast and cervical screening practices among adult and older women in Ghana | Cross-sectional study | Journal article | Ghana | WHO study on global AGEing and adult health (SAGE) Wave 1 (2007–2008) in Ghana. | International study | 2007-2008 | Demographics, Economic, PCP access, Social | No |
| Aynalem 2020 (125) | To investigate the utilization of cancer screening and its associated factors among women in Debremarkos town, Amhara region, Ethiopia | Community-based cross-sectional study | Journal article | Ethiopia | NR | Town-wide and community based survey | From July 1 to August 30, 2018 | Demographics, Economic, Patient access, Patient behavior, Social | No |
| Babazadeh 2018 (126) | To assess the cognitive determinants of Cervical Cancer Screening Behavior (CCSB) among housewife women in Islamabad County, Iran. | Cross-sectional study | Journal article | Iran | NR | Multicenter | Mar to May 2015 | Demographics, Patient access, Patient behavior | No |
| Badre-Esfahani 2019 (127) | To analyze the association between non-adherence to HPV vaccination and non-participation in cervical cancer screening for the total population and stratified by native background and parental education | Retrospective closed cohort study | Journal article | Denmark | Danish Civil Registration System, Danish National Health Service Register, Danish National Prescription Registry, Danish Pathology Register, | Nationwide study | 1 Oct 2008 to 31 Dec 2017 | Demographics, Economic, Social | No |
| Badre-Esfahani 2020 (128) | To investigate differences in combined non-attendance by nativity and region of origin, and to analyze the association between country of origin and combined non-attendance adjusted for socio-economic status. | Retrospective closed cohort study | Journal article | Denmark | Danish National Prescription Registry | Nationwide study | 1 June 2007 to 31 Dec 2016 | Demographics | No |
| Bao 2018b (129) | To investigate cervical cancer screening rates in relation to both individual-level and geographical measures of socioeconomic status (SES). | Cross-sectional study | Journal article | China | Chinese Chronic Disease and Risk Factor Surveillance (CCDRFS), | Nationwide study | Aug 2013- Jul 2014 | Demographics, Economic, Patient access, Social | No |
| Barrett-Harrison 2018 (130) | To assess the extent to which six sociodemographic variables and three lifestyle practices of women are associated with Pap smear testing, given that cervical cancer is the second leading cause of women’s cancer mortality in Jamaica and that this cancer is preventable with the use of screening methods such as the Pap smear | Cross-sectional study | Journal article | Jamica | Jamaica’s 2008 Reproductive Health Survey | National survey | 2008 | Demographics, Economic, Patient behavior, Social | No |
| Baruch 2022 (131) | To examine the association between physical disability and Pap smear receipt in Israel | Cross-sectional study | Journal article | Israel | Electronic medical record of institutional database | Single center | 2012–2017 | Demographics, Economic, Patient behavior | No |
| Bayu 2016 (132) | To determine cervical cancer screening service uptake and its associated factor among age eligible women in Mekelle zone, northern Ethiopia, 2015. | Cross-sectional study | Journal article | Ethiopia | NR | Community wide study | Feb- Jun 2015 | Demographics, Economic, Patient access, Patient behavior, Social | No |
| Belay 2020 (133) | To assess cervical cancer screening service utilization and associated factors among women aged 30 to 49 years in Dire Dawa, eastern Ethiopia | Cross-sectional study | Journal article | Ethiopia | NR | City wide study | Feb 01- Mar 01, 2017 | Demographics, Economic, Patient access, Patient behavior, PCP access | No |
| Bermedo-Carrasco 2015 (134) | This study identify factors associated with whether women in Colombia have had a Pap test, evaluate differences in risk factors between rural and urban residence, and evaluate the contextual effect of the lack of education on having ever had a Pap test | Cross sectional survey | Journal article | Columbia | 2010 Colombian National Demographic and Health Survey | National survey | 2010 | Demographics, Economic, Patient access, PCP access, Social | No |
| Bianco 2017 (135) | To explore breast and cervical CS participation and to acquire information regarding access to healthcare services during pregnancy, childbirth and the postpartum period among age eligible immigrant women in Southern Italy | Cross-sectional study | Journal article | Italy | NR | Community based survey | May 2012 to April 2013 | Demographics, Economic, Patient behavior, Social | Yes |
| Boni 2021 (136) | To assess Cervical Cancer screening uptake and its associated factors in Abidjan in 2018 | Cross-sectional, observational study | Journal article | Côte d'Ivoire | NR | Multicenter | Jul to Sep 2018 | Demographics, Economic, Patient access, Patient behavior, Social | No |
| Bou-Orm 2018 (137) | The aim of this paper was to assess the prevalence of Pap smear screening for cervical cancer among Lebanese women and to determine associated sociodemographic and psychosocial characteristics. | Cross-sectional study | Journal article | Lebanon | NA | Nationwide Population-based study | NR | Demographics, Patient access, Patient behavior, Social | No |
| Brown 2016 (138) | To compare cervical cancer screening rates in women with and without intellectual and developmental disabilities (IDD) who had had a pregnancy. | Retrospective cohort study | Journal article | Canada | Ontario health and social services administrative database | Nationwide population based | April 1, 2007 and March 31, 2010 | Demographics, Economic, Patient access | No |
| Brown 2019 (139) | To examine whether the cervical cancer screening barriers and facilitators identified by women were related to their screening history, from November 2015 and January 2016 | Cross-sectional study | Journal article | Australia | NR | City-wide survey | November 2015 to January 2016 | Demographics, Patient access, Patient behavior | No |
| Bruera 2020 (140) | To estimate cervical cancer screening rates in women with recently diagnosed SLE, and to identify characteristics associated with decreased screening. | Case-control study | Journal article | USA | United States Market Scan Commercial Claims and Encounter (CCAE, age 18-64) administrative claims database | Nationwide study | 2001-2005 | Demographics | No |
| Brzoska 2020 (141) | To examine the role that different predisposing, enabling and need factors have for the participation of migrant and non-migrant women in cervical cancer screening in Germany | Cross-sectional study | Journal article | Germany | ‘German Health Update 2014/2015 | National survey | November 2014 and July 2015 | Demographics, Economic, Social | No |
| Budkaew 2014 (142) | To identify factors associated with women’s decisions to attend cervical cancer screening and to explore those linked with intention to attend in the coming year and to continue regular screening. | Case-control study | Journal article | Thailand | Medical record in CCMU, | Community wide study | Oct-Dec 2013 | Demographics, Economic, Patient access, Patient behavior, PCP access, Social | No |
| Bussière 2015 (143) | To explore the rate and determinants of the likelihood of cervical cancer screening participation among disabled women living in institutions in France | Cross-sectional study | Journal article | France | French national Health and Disability Survey | Nationwide study | 2009 | Demographics, Economic, Patient access, Social | No |
| Calys-Tagoe 2020 (144) | This study assesses the cervical cancer screening practices among women in Ghana. | Cross-sectional study | Journal article | Ghana | World Health Organization’s (WHO) multi-country Study on AGEing and adult health (SAGE) wave 2 conducted between 2014 and 2015 | International wide survey | 2014- 2015 | Demographics, Economic, Patient access, Patient behavior, PCP access | No |
| Cerigo 2013 (145) | The objectives of this study were to determine Pap smear utilization rates and to determine factors associated with time-inappropriate use of cervical cancer screening among a cohort of Inuit women from Nunavik, Quebec, Canada. | Retrospective chart review | Journal article | Canada | NR | Region wide study | Jan 2002 to Dec 2007 | Demographics, Economic, Patient behavior, Social | No |
| Chan 2022b (146) | To assess the cervical cancer screening uptake rate (Pap test) of South Asian (Indian, Pakistani and Nepalese) women in Hong Kong, their perceived barriers to cervical cancer screening, and the association of these factors with their overall screening uptake. | Cross-sectional study | Journal article | China | NR | Community based survey | April to November 2017 | Demographics, Economic, Patient access, Patient behavior, PCP access, Social | No |
| Chandrika 2020 (147) | To assess the awareness about cervical cancer, willingness, and barriers for undergoing screening of cervical cancer among women in urban Pondicherry | Cross-sectional study | Journal article | India | NR | Community wide study | Jan to Jul 2019 | Demographics, Economic, Patient access, Patient behavior, Social | No |
| Chaowawanit 2016 (148) | To assess knowledge, attitudes and cervical cancer screening behavior of Bangkok Metropolitan women. | Cross-sectional study | Journal article | Thailand | NR | Multicenter | Mid of September until the end of December 2014 | Demographics, Economic, Patient access, Patient behavior, Social | No |
| Chiou 2014 (149) | To examine changes in the magnitude of social inequality in the uptake of cervical cancer screening between 2001 and 2009 in Taiwan | Cross-sectional study | Journal article | Taiwan | 2001 and 2009 Taiwan National Health Interview Surveys | Nationwide study | 2001, 2009 | Demographics, Economic, Social | No |
| Chirwa 2022 (150) | To examine the socioeconomic inequality in cervical cancer screening uptake using concentration indices, in Malawi. Furthermore, it decomposes the concentration index to examine how each factor contributes to the level of inequality in the uptake of cervical cancer screening | Cross-sectional study | Journal article | Malavi | Nationally representative Malawi Population HIV Impact Assessment (MPHIA) household survey | National survey | Between November 2015 and August 2016 | Demographics, Economic, Social | No |
| Choi 2022 (151) | To describe the prevalence and predictors of both screening and positive HPV results among women attending these campaigns. | Cross-sectional study | Journal article | Kenya | NR | Community wide multi‑disease health campaign | April to Jun 2018 | Demographics, Economic, Patient access, Patient behavior, Social | No |
| Cofie 2018 (152) | To examine whether chronic comorbidities are associated with cervical cancer screening recommendation and adherence, and whether associations vary between foreign-born and US-born women. | Cross-sectional household survey | Journal article | USA | National Health Interview Survey (NHIS) | Nationwide study | 2013 to 2015 | Demographic, PCP access | No |
| Compaore 2016 (153) | To evaluate barriers to cervical screening among patients in the gynecology department of the university hospital | Cross-sectional study | Journal article | Burkina Faso | Centre Hospitalier Universitaire Yalgado Ouedraogo de Ouagadougou (CHUYO), the university teaching hospital in Ouagadougou | Single center | May to Aug 2014 | Demographics, Economic, Social | No |
| Cunningham 2015 (154) | To determine cervical cancer screening coverage and the knowledge, attitudes and barriers toward screening tests among women in rural and urban areas of Tanzania, as well as explore how they view the acceptability of the HPV vaccine and potential barriers to vaccination. | Cross-sectional study | Journal article | Tanzania | NR | Region wide study | May to July 2012 | Demographics, Economic, Patient access | No |
| Da Silva 2022 (155) | To identify the factors associated with the non-performance of cervical cancer screening in rural riverside populations along the Rio Negro River, Manaus, Amazonas, covered by the FHT | Cross sectional survey | Journal article | Brazil | NR | Community based survey | January 2016 to May 2019 | Demographics, Economic, Patient access, Patient behavior, Social | No |
| Danan 2022 (156) | To determine whether a history of sexual assault is associated with reduced cervical cancer screening completion among women Veterans. | Observational retrospective cohort study | Journal article | USA | VA electronic health record (EHR) data | Nationwide study (telephone survey) | January to March 2015 | Demographics, Patient access, Patient behavior | No |
| De Prez 2021 (157) | To examine: (1) whether the magnitude of income and education-based inequalities in CCS uptake is different according to the accessibility of the healthcare system, and the broader level of social protection; (2) under which healthcare system access and social protection conditions an organized CCS program relates to lower levels of inequality in CCS uptake. | Cross-sectional study | Journal article | Norway, Iceland, and Switzerland | European Health Interview Survey’s second wave, | International survey | 2013-2015 | Economic, Patient access, Social | No |
| De Vito 2014 (158) | The aims of this study were to compare the characteristics of women who got a Pap-test during the mass media campaign, carried out in an Italian region by broadcasts advertising, and two years later and to identify the determinants of knowledge of cervical cancer etiology and of the adherence to the mass media campaign. | Cross-sectional study | Journal article | Italian | NR | Regional wide study | Jan to Mar 2006 | Demographics, Economic, Patient access, Patient behavior | No |
| Deguara 2021 (159) | To assess the knowledge of 25-64  year-old females on cervical cancer and attitudes towards screening. | Cross-sectional study | Journal article | Malta | NR | Nationwide study | 2017 | Demographics, Economic, Social | No |
| Desta 2022 (160) | To assess cervical cancer screening utilization and associated factors among women 30–65 years in north shoa Ethiopia. | Cross-sectional study | Journal article | Ethiopia | NR | Community wide study | March one to May one, 2021. | Demographics, Economic, Patient access, Patient behavior, Social | No |
| Destaw 2021 (161) | To assess uptake and associated factors with the cervical cancer screening “see and treat approach” among eligible women in public health facilities in Gondar town, Northwest Ethiopia. | Cross-sectional study | Journal article | Ethiopia | NR | Multicenter | 2019 | Demographic, Patient access, Patient behavior, Social | Yes |
| Do 2015 (162) | To determine the predictors of Cervical Cancer Screening Among Vietnamese American Women | Cross-sectional study | Journal article | USA | NR | National survey | 2010 | Demographics, Patient access, Patient behavior, PCP access, Social | No |
| Drolet 2013 (163) | To identify the sociodemographic characteristics of women who reported greater sexual activity and/or screening underuse | Cross-sectional study | Journal article | Canada | Canadian Community Health Survey (CCHS Cycle 3.1, 2005) and the Psychosocial impact of abnormal smear pap and condylomas in Canada | Nationwide study and Multicenter study | Jan 2005 to Dec 2005 | Demographics, Economic, Social | No |
| Dulla 2017 (164) | To assess the knowledge and practice of cervical cancer screening among female health care workers in southern Ethiopia. | Cross-sectional study | Journal article | Ethiopia | NR | Multicenter | March to April 2015 | Demographics, Economic, Patient access, PCP access, Social | No |
| Ebu 2018a (165) | This study hypothesized that more HIV-positive women with high cues about cervical cancer screening, high perceived susceptibility to cervical cancer, high perceived seriousness of cervical cancer, high perceived benefits of cervical cancer screening, and low perceived barriers about cervical cancer screening have intention to seek cervical cancer screening than HIV-positive women with low cues, low perceived susceptibility, low perceived seriousness, low perceived benefits, and high perceived barriers | Descriptive cross-sectional study | Journal article | Ghana | NR | Province based survey | 2014 | Patient behavior | No |
| Ebu 2018b (166) | To determine the socio-demographic factors influencing intention to seek cervical cancer screening by HIV-positive women in the Central Region of Ghana. | Descriptive cross-sectional study | Journal article | Ghana | NR | Multicenter | NR | Demographics, Economic, Social | No |
| El Mhamdi 2012 (167) | To investigate women’s knowledge, attitudes, and practices of cervical cancer screening in the region of Monastir (Tunisia). | Cross-sectional study | Journal article | Tunisia | NR | Multicenter | 1 Mar to 30 Jun 2009 | Demographics, Economic, Patient access, Patient behavior, Social | No |
| Elit 2012 (168) | To determine the sociodemographic factors associated with cervical cancer screening and follow-up of abnormal results. | Retrospective cohort study | Journal article | Canada | Cyto base, Ontario Health Insurance Plan (OHIP) database, Ontario Cancer Registry, Canadian Institute for Health Information’s Discharge Abstract Database, Registered Persons Database (RPDB) | Province wide study | 2004-2005 | Demographics, Economic, Patient access, Patient behavior | No |
| Elit 2013 (169) | To determine whether progress has been made in screening coverage and follow-up of high grade Pap tests since that era. Also assess how patient factors (i.e., patient age, socioeconomic class (SEC)) and regional variation influenced coverage and follow-up | Retrospective population-based cohort study | Journal article | Canada | Ontario Cancer Registry (OCR). The Canadian Institute for Health Information (CIHI) Discharge Abstract Database (DAD), | Provence wide study | 2008-2010 | Demographics, Economic, Patient access | No |
| Emmanuel 2016 (170) | To determine the predictors and factors related to the uptake of cervical cancer screening test among female secondary school teachers in Sagamu | Cross-sectional study | Journal article | Nigeria | NR | Region wide study | May and June 2013 | Demographics, Patient access, Patient behavior, Social | No |
| Enyan 2022 (171) | This study investigated Muslim women’s participation in, intention to engage in, and self-efficacy about CCS | Cross-sectional study | Journal article | Ghana | NR | Community wide study | Feb- Apr 2021 | Demographics, Economic, Patient access, Patient behavior, PCP access, Social | No |
| Erku 2017 (172) | To assess the level of knowledge of about cervical cancer and uptake of screening among HIV infected women in Gondar, northwest Ethiopia | Cross sectional study | Journal article | Ethiopia | NR | City-wide survey | March 1 to 30, 2017. | Demographics, Patient access, Patient behavior, Social | No |
| Eshete 2020 (173) | This study aimed at assessing cervical cancer screening acceptance and determinant factors among women in Dabat district of Northwest Ethiopia | Community-based cross-sectional survey | Journal article | Ethiopia | NR | Community based survey | January 2017 to March 2017 | Demographics, Economic, Patient access, Patient behavior, Social | No |
| Faye 2017 (174) | The purpose of this survey is to study the factors of cervical cancer screening. | Cross‑sectional, descriptive and analytical survey | Journal article | Senegal-West Africa | NR | Multicenter | NR | Economic, Patient access, PCP access, Social | No |
| Fokom 2019 (175) | To determine the prevalence and predictors of cervical cancer screening with co-testing among women 30 years or older in Texas. | Cohort study | Journal article | USA | Population based state wide survey 2018, representative sample of the Texas population | State-wide survey | 2018 | Demographics, Economic, Patient access, Patient behavior, Social | No |
| Ford 2021 (176) | To study differences in screening adherence and follow-up after an abnormal Pap test in Non- Hispanic Black (Black) and Non-Hispanic White (White) women. | Cross-sectional study | Journal article | USA | National Health Interview Survey cancer module | Nationwide Population-based study | 2010 | Patient access | No |
| Galvin 2021 (177) | To identify the associations of the three health literacy domains (accessing, understanding, and appraising) with cervical cancer guideline adherence, which represents a fourth health literacy domain (applying) | Cross sectional study | Journal article | USA | Centers for Disease Control and Prevention | National survey | May to June 2018 | Demographics, Economic, Patient access, Patient behavior, Social | No |
| Gan and Dahlui 2013 (178) | To assess the cervical screening practices of rural women in Malaysia and to examine the factors associated with such practices. | Cross-sectional study | Journal article | Malaysia | NR | State wide study | NR | Demographics, Economic, Patient access, Patient behavior, PCP access, Social | No |
| Gatumo 2018 (179) | To assess women’s knowledge and attitudes towards cervical cancer and cervical cancer screening in Kenya’s Isiolo and Tharaka Nithi counties | Cross-sectional quantitative survey | Journal article | Kenya | NR | County wide study | Jan to March 2017 | Demographics, Economic, Social | No |
| Gebisa 2022 (180) | To assess knowledge, attitude, and practice toward cervical cancer screening among women attending health facilities in Ambo town, central Ethiopia. | Cross-sectional study | Journal article | Ethiopia | NR | Town-wide survey | From August to September 2020 | Patient access, Patient behavior, Social | No |
| Gebreegziabher 2016 (181) | To assess the magnitude and factors affecting the practices of cervical cancer screening among female nurses in Mekelle Town, Tigray, Northern Ethiopia, 2014 | Cross-sectional study | Journal article | Ethiopia | NR | Multicenter | 2014 | Demographics, Economic, Patient access, Patient behavior, Social | No |
| Gemeda 2020 (182) | To determine the prevalence and predictors of cervical cancer screening service uptake among women aged 25 years and above in Sidama zone, southern Ethiopia, 2019 | Cross-sectional study | Journal article | Ethiopia | NR | Region wide study | 2019 | Demographics, Patient access, Patient behavior, Social | No |
| Gerend 2017 (183) | To examine women's acceptance of less frequent cervical cancer screening now that the 2012 guidelines have been in place for several years | Cross-sectional study | Journal article | USA | NR | National survey | 2014 | Demographics, Economic, Patient access, PCP access, Social | No |
| Getachew 2019 (184) | To assess cervical cancer screening knowledge and barriers for screening uptake among women in Addis Ababa Ethiopia | Cross-sectional facility-based study | Journal article | Ethiopia | NR | City wide study | Feb to March 2015 | Demographics, Economic, Patient access, PCP access, Social | Yes |
| Ghimire 2021 (185) | To find out the determinants of uptake of cervical cancer screening among women. | Descriptive, cross-sectional study | Journal article | Nepal | NR | Single center | 04 to 30 Sep 2016 | Demographics, Patient access, Patient behavior, Social | No |
| González 2015 (186) | To assess the prevalence of cervical cancer screening through Papanicolaou (Pap) test utilization and its association with sexual behaviors among a population-based sample of Hispanic women in Puerto Rico (PR) | Cross-sectional study | Journal article | Puerto Rico | NR | State wide study | Aug 2010 to May 2013 | Demographics, Economic, Patient access, Patient behavior, Social | No |
| González 2022 (187) | To identify factors associated with CC screening program attendance for women living in Colombia’s Amazon region | Prospective study | Journal article | Columbia | NR | Town-wide survey | Qualitative phase: September 2015 to March 2016; Quantitative phase: April to November 2016 | Demographics, Economic, Patient access, Patient behavior, PCP access, Social | No |
| Gottschlich 2019 (188) | This study compared responses between Buddhist and Muslim women using two-sided t-tests and χ2 tests for continuous and categorical variables, respectively. A similar procedure was used to compare women who had previously been screened for cervical cancer with those who had not. Multivariate logistic models were then run to examine potential predictors for prior screening, after adjusting for confounders. Finally, a descriptive analysis was conducted to assess acceptability of the self-swab test, where an α<0.05 was considered significant. All analyses were conducted using R V.3.4.4. | Cross sectional study | Journal article | Thailand | Data collection took place in two districts within the Songkhla Province of Southern Thailand | Province based survey | July to December in 2017 | Demographics | No |
| Grillo 2012 (189) | To describe the individual characteristics associated with the absence of cervical smear (CCST); to investigate the role of residential neighborhood, particularly practitioner density; and to explore changes in individual and contextual determinants after taking regular consulting in primary care for gynecological health (RCGH) into account. | Cross-sectional study | Journal article | France | SIRS (French acronym for Health, Inequalities and Social Ruptures) survey | City wide study | 2005 | Demographics, Economic, Patient access, Patient behavior, PCP access, Social | No |
| Gu 2012 (190) | The aim of this study was to describe women’s knowledge and perceptions of cervical cancer risk and factors influencing utilization of cervical cancer screening using PMT framework. Objectives of the study were: (i) To examine Chinese women’s cervical screening pattern (ii) To examine Chinese women’s knowledge and perception of personal risk and risk factors of cervical cancer (iii) To examine Chinese women’s perception of body health beliefs and knowledge about cervical screening (iv) To identify factors that were associated with Chinese women’s screening behavior | Cross-sectional study | Journal article | Chinese | NR | City wide study | 2017 | Demographics, Patient access, Patient behavior, Social | No |
| Gyulai 2015 (191) | To study the socioeconomic and lifestyle factors related to participation in the organized cervical cancer screening program | Cross-sectional survey | Journal article | Hungary | NR | Province (11 Hungarian counties) survey | 2008 | Demographics, Economic, Patient behavior, Social | No |
| Haas 2021 (192) | This study aim to understand how multi-level factors influence the cancer screening processes. | Cross-sectional study | Journal article | USA | Research Optimizing Screening through Personalized Regimens (PROSPR) | Multicenter | 42339 | Demographics, Economic, Patient access, Patient behavior, PCP access, Social | No |
| Harder 2018a (193) | To identify determinants for participation in HPV self-sampling in a Danish population of nonattenders at routine screening. | Cross-sectional study | Journal article | Denmark | NR | Region wide study | May 2014 to April 2015 | Demographics, Economic, Patient access, Patient behavior, Social | No |
| Harder 2018b (194) | To describe nonparticipants in the Danish cervical cancer screening program, including potential differences in socio-demographic characteristics between active and passive non-participants. Furthermore, to assessed the role of socio-demographic factors, reproductive history, and mental and physical health for passive non-participation in cervical cancer screening in Denmark. | Cross-sectional study | Journal article | Denmark | Pathology Databank , National Patient Register, Cancer, Register, Medical Birth Register, Prescription Register,, Psychiatric Central Research Register, Psychiatric outpatient contacts, Databases in Statistics Denmark, | Nationwide study | 2008-2009 | Demographics, Economic, Patient access, Patient behavior, Social | No |
| Harder 2020 (195) | To investigate associations between general health, lifestyle and sexual behavior, and non-participation in cervical cancer screening in Denmark – while taking into account established sociodemographic risk factors for low screening participation | Prospective cohort study | Journal article | Denmark | A national register, the Pathology Databank | Nationwide study | 2011-2012 | Demographics, Patient behavior, Social | No |
| Harper 2022 (196) | To evaluate the predictors of cervical cancer screening among MENA, White and Black women of southeast Michigan | Cross-sectional study | Journal article | Southeast Michigan | Southeast Michigan White, Black, and MENA populations-National Institute on Minority Health and Health Disparities Research Framework (NIMHD), | State wide study | May 1 and October 28, 2019, | Demographics, Economic, Patient access, Patient behavior, PCP access, Social | No |
| Holt 2021 (197) | The objective of our study was focused on evaluating uptake of cervical cancer screening services in migrant workers compared to non-migrant workers in major metropolitan areas of China | Cross-sectional study | Journal article | China | NR | Multicenter | 42071 | Demographics, Patient access, Social | No |
| Idehen 2017 (198) | To explore factors associated with cervical screening participation among women of Russian, Somali, and Kurdish origin in Finland. | Cross-sectional study | Journal article | Finland | Migrant Health and Well-being Survey, 2010-2012 | Nationwide study | 2010-2012 | Demographics, Economic, Social | No |
| Idehen 2020 (199) | To examine cervical cancer screening participation and factors associated with it in the Finnish mass screening program during 2008–2012 in women of Russian, Somali, and Kurdish origin compared with the general Finnish population in Finland. | Retrospective cohort study | Journal article | Finland | Finnish screening registry (1) National Population Register, Mass Screening Registry, Care Register for Health Care, medical birth register, register of included abortions, statistics Finland, social insurance institute of Finland. The data from different sources for persons in the Maamu and Health 2011 Survey samples were linked using the unique personal identity codes given to Finland’s legal residents. | Nationwide study | 2008 to 2012 | Demographics, Economic, Patient access, Social | No |
| Idowu 2016b (200) | The study objectives were to assess women awareness level, their knowledge about cervical cancer and cervical cancer screening, and their attitudes to cervical cancer screening. It also sought to identify factors influencing uptake of screening programs among women residing in Ilorin, Kwara State. | Cross sectional study | Journal article | Nigeria | NR | Community based survey | 2006 | Demographics, Economic, Patient behavior | Yes |
| Isabirye 2020 (201) | To examine the demographic, social, and economic predictors of cervical cancer screening in Central Uganda with the aim of informing targeted interventions to improve screening. | Cross-sectional study | Journal article | Uganda | NR | Region wide study (Multi-center) | June to July 2019 | Demographics, Economic, Patient access, Patient behavior, PCP access, Social | No |
| Islam 2015 (202) | To investigate the awareness of CCa and CCa screening, and factors associated with women’s preparedness to be screened | Cross-sectional study | Journal article | Bangladesh | NR | Nationwide study | Sept 2013 to Mar 2014 | Demographics, Economic, Social | No |
| Issa 2021 (203) | The aim of this study was to investigate factors related to cervical cancer screening behavior of women in Kazakhstan. | Cross-sectional survey-based study | Journal article | Kazakhstan | NR | Multicenter | 25 May 2019 to Feb 2020 | Demographics, Patient access, Social | No |
| Jabbari 2019 (204) | To assess post-menopausal women behavior regarding cervical cancer screening | Cross -sectional population-based telephone survey | Journal article | Iran | Telephone survey, conducted among women in the post-menopausal period in Tabriz, Iran | Providence based survey | 42675 | Demographics, Economic, Patient behavior, PCP access, Social | No |
| Jang 2021 (205) | This study examines (1) the association between medical tourism and cervical cancer screening among immigrant women and (2) whether the association varies across years in the U.S. | Retrospective study of longitudinal cohort study | Journal article | USA | New Immigrant Survey (NIS) | Nationwide study | 2003 to 2004 (Round 1) and 2007 to 2009 (Round 2) | Demographics, Patient access, Social | No |
| Johnson 2016 (206) | To measure the prevalence of, and identify factors associated with, cervical cancer screening among a sample of lesbian, bisexual and queer women, and transgender men. | Cross-sectional study (A convergent-parallel mixed methods design.) | Journal article | USA | NR | Nationwide study | Aug 2014–Dec 2014 | Demographics, Patient access, Patient behavior, PCP access | No |
| Judah 2022 (207) | The aim of this study was to understand the demographic, and individual factors associated with self-reported attendance at cervical screening in London. Using a survey we aim to establish the strongest barriers and facilitators predicting past CS attendance. | Cross-sectional study | Journal article | UK | NR | City-wide survey | June-July 2017 | Economic, Patient behavior, Social | No |
| Kaneko 2018 (208) | To assess the prevalence of and factors correlated with cervical cancer screening among unmarried and sexually active Japanese women aged 20–29 years. | Cross-sectional study | Journal article | Japan | NR | Country wide study | 42090 | Demographics, Patient access, Patient behavior, Social | No |
| Kasim 2020 (209) | To assess cervical cancer screening utilization and associated factors among women in the Shabadino district, Southern Ethiopia | Cross-sectional study | Journal article | Ethiopia | NR | Community wide study | Feb to Mar 2018. | Demographics, Patient access, Social | No |
| Kaso 2019 (210) | To determine the factors related to undergoing cervical cancer screening among Japanese women of childrearing age by focusing on the presence or absence of children | Cross-sectional study | Journal article | Japan | Comprehensive Survey of Living Conditions 2013 | National survey | 2013 | Demographics, Economic, PCP access, Social | No |
| Kasting 2017 (211) | The present study examined the relationship between HPV vaccination and Pap testing using responses to an exploratory cross-sectional survey of mostly minority women. Used both quantitative and qualitative methodology to: 1) examine if cervical cancer screening rates differed between those who had and had not been vaccinated and 2) assess if women understood the purpose of, and current recommendations for, Pap testing. | Cross-sectional study | Journal article | USA | NR | Community based survey | Conducted in July 2015, analyzed in 2015 and 2016 | Demographics, Patient access, Social | No |
| Khanna 2019 (212) | Aims to determine the factors deciding the uptake of cervical screening amongst women in rural India | Cross-sectional, observational study | Journal article | India | NR | Single center | Oct 2016 - Oct 2017 | Demographics, Economic, Patient access, Patient behavior, Social | Yes |
| Kileo 2015 (213) | To investigate utilization of cancer screening services, and its associated factors among female primary school teachers in Ilala Municipality, Dares Salaam. | Cross sectional study | Journal article | Tanzania | NR | Community based survey (Municipality) | May – August 2011 | Demographics, Patient access, Patient behavior, Social | No |
| Kouyoumdjian 2018 (214) | To determine cervical cancer screening rates for women in provincial prison in Ontario, Canada, and to compare these data with data for the general population. | Retrospective cohort study | Journal article | Canada | Ontario Ministry of Community Safety and Correctional Services (MCSCS) | Multicenter | 2005 - 2015 | Demographics | No |
| Kue 2017 (215) | This study present findings on following research questions: (1) What is the cervical cancer screening status among Bhutanese–Nepali women? (2) What are barriers and facilitators to cervical cancer screening among Bhutanese–Nepali women? (3) What are Bhutanese–Nepali women’s beliefs about Pap smears? and (4) What are post migration living difficulties for Bhutanese–Nepali women? | Cross-sectional study | Journal article | USA | NR | Community wide study | Between July to October of 2015 | Demographics, Patient behavior, PCP access | No |
| Lee 2012 (216) | To compare health beliefs related to cervical cancer screening utilization between older (65 years or older) and younger (between 40 and 64 years old)Korean American (KA) women to understand older KA women’s lower screening rates. | Cross-sectional study | Journal article | USA | NR | Community wide study | NR | Demographics, Patient access, Social | No |
| Lee 2013b (217) | To investigate the change in cervical cancer screening rates, the level of socioeconomic disparities in cervical cancer screening participation, and whether there was a reduction in these disparities between 1998 and 2010 | Cross-sectional study | Journal article | Korea | Korean Health and Nutrition Examination Survey | National survey | 1990-2010 | Demographics, Economic, Social | No |
| Lee 2019c (218) | This study aimed to investigate cervical cancer screening behaviors among college-aged females by (1) determining AAPIs" and NLWs" screening rates, (2) assessing their knowledge about Pap tests, and (3) discovering factors associated with Pap test receipt. Andersen’s Health Behavioral Model was used as a theoretical framework. | Cross-sectional study | Journal article | USA | NR | Single center | NR | Demographics, Patient access, PCP access, Social | No |
| Leinonen 2017 (219) | To assess to what extent the characteristics of the regular general practitioner (GP), sociodemographic factors, and screening and disease history of women in Norway were associated with non-adherence and reminded adherence to cervical screening | Cross-sectional study | Journal article | Norway | Norwegian Cervical Cancer Screening Program (NCCSP) | Nationwide study | 2008-2012 | Demographics, Economic, Patient access, PCP access, Social | No |
| Lemma 2022 (220) | To assess the predictors of cervical cancer screening usage among women aged 30–49 years in Ambo Town | Case-control study | Journal article | Ethiopia | NR | Town-wide and community-based survey | February 1–March 30, 2020 | Demographic, Economic, Patient access, Patient behavior, Social | No |
| Liang 2022 (221) | To estimate colposcopy non-attendance among screen-positive women from a population-based, real-world screening study involving co-testing and to examine the potential factors associated with attendance. Additionally, the study described non-attendance reasons | Prospective cohort study | Journal article | Germany | NR | Country wide study | 2005-2007 | Demographics, Economics, Patient access, Patient behavior, PCP access, Social | No |
| Lin 2021 (222) | To detect the changes of cervical cancer screening rate and willingness among female migrants, and the associated socio-demographic factors in Shenzhen city. | Cross-sectional epidemiologic study | Journal article | China | NR | City wide study | 2011 to 2014 (1st survey: 2011 to 2014 and 2nd survey: Sep to Oct 2014) | Demographics, Economic, PCP access, Social | No |
| Lin 2022 (223) | To examine the previous uptake of cervical cancer screening and intention to be screened and its associated factors among women living with HIV in China. | Cross-sectional study | Journal article | China | NR | Multicenter | Jan 2019 to Jun 2019 | Demographics, Economic, Patient access, Social | No |
| Liu 2017 (224) | To assess knowledge and attitudes about cervical cancer and its screening among rural women aged 30 to 65 years in eastern China. | Cross-sectional study | Journal article | Eastern China | NR | City wide study | 42217 | Demographics, Economic, Social | Yes |
| Lyimo 2012 (225) | To identify the most important factors related to the uptake of cervical cancer screening among women in a rural district of Tanzania | Cross-sectional study | Journal article | Tanzania | NR | Region wide study | 2002 | Demographics, Patient access, Patient behavior, PCP access, Social | No |
| Mabotja 2021 (226) | To determine the associations between women’s beliefs about cervical cancer and screening and the uptake of Papanicolaou (Pap) smears in Johannesburg, where cervical screening uptake is suboptimal. | Cross-sectional study | Journal article | South Africa | NR | Multicenter | Jun- Aug 2017 | Demographics, Economic, Patient access, Patient behavior, Social | No |
| Maharjan 2020 (227) | To explore and identify differences and factors affecting the health beliefs and practices of cervical cancer screening of women in the mountainous and the Terai regions. | Cross-sectional descriptive study | Journal article | Nepal | NR | Region wide study | June to Sept 2017 | Demographics | No |
| Marlow 2017a (228) | The aims were: (1) to establish the percentage of British women classified into each cervical cancer screening non-participant type, as outlined by the PAPM and (2) to identify sociodemographic correlates with each nonparticipant type. | Cross-sectional study | Journal article | UK | TNS (a market research agency) as part of their Omnibus survey | Nationwide study | Jan/Feb 2016 | Demographics, Economic, Social | No |
| Marques 2022 (229) | This study aims to characterize migrant women’s participation in CCS and determine factors associated with non-attendance to CCS. | Cross-sectional study | Journal article | Portugal | NA | Nationwide study | February 2021 - July 2021 | Demographics, Economic, Patient access, Patient behavior, PCP access, Social | No |
| Martín-López 2012 (230) | The aims of this study were to assess the use of Pap smears in Spain in 2009 to identify factors associated with screening adherence (predictors) and assess the trend from 2003 to 2009. | Cross-sectional study | Journal article | Spain | European Health Interview Survey for Spain (EHISS) | Nationwide study | Data collection period: Apr 2009 to Mar 2010. Analysis period: Jan 2011 | Demographics, Economic, Patient behavior, PCP access, Social | No |
| Mboineki 2020 (231) | To assess the predictors in the uptake of cervical cancer screening (CCS) among women in Tanzania | Community-based cross-sectional study | Journal article | Tanzania | NR | Province based survey | 14th August 2019 to 29th December 2019 | Demographics, Patient access, Patient behavior | No |
| McDaniel 2021 (232) | To evaluate cervical cancer screening behaviors among females with and without diabetes across the U.S. by investigating their HPV testing practices in 2016, 2018, and 2020. | Cross-sectional study | Journal article | USA | Behavioral Risk Factor Surveillance System (BRFSS) | Nationwide study (telephone survey) | 2016, 2018, 2020 | Demographics, Economic, Patient access, Patient behavior, Social | No |
| Mengistu 2022 (233) | To identify determinants of positive cervical cancer screening among reproductive-age women in the South Wollo Zone, Amhara region, northeast Ethiopia. | Unmatched case-control study | Journal article | Ethiopia | NR | Region wide study | 28 Jan 2020 to 12 Apr 2020 | Demographics, Patient access, Social | No |
| Midaksa 2022 (234) | To determine whether sexual autonomy determines lifetime CC screening uptake using a direct comparison | Unmatched case-control study | Journal article | Ethiopia | Institutional-based study | Multicenter | 2019 | Demographics, PCP access, Social | No |
| Miles-Richardson 2017 (235) | The objective of this study was to examine the factors associated with cervical cancer screening among women 18 years of age and older in the United States (U.S.). | Cross sectional study | Journal article | USA | Behavioral Risk Factor Surveillance System (BRFSS); | National survey | 2012 | Demographics, Economic, Social | No |
| Moore 2015 (236) | To explore beliefs, attitudes, socio-economic, and cultural factors influencing Hispanic women’s decisions about cervical cancer screening. | Cross-sectional study | Journal article | USA | NA | Multicenter | NR | Demographics, Patient behavior | No |
| Mpamani 2019 (237) | To identify factors associated with participation in screening at a PHF and referral to a high-level facility, which provides better understanding of lost follow-up after screening. | Cross-sectional study | Journal article | Uganda | NR | Single center | Feb- Mar 2018 | Demographics, Patient access, Patient behavior, Social | No |
| Ncube 2015 (238) | The objectives of this study were to determine the prevalence of cervical cancer screening (Pap smear) and identify factors associated with the uptake of screening among women in the mostly rural parish of Portland, Jamaica. These findings could be used to inform public policy, develop and implement strategies to increase screening activities, thereby reducing incidence, morbidity, and mortality from the disease. | Cross-sectional, population-based survey study | Journal article | Jamaica | NR | Province based survey | May through August of 2005 | Demographic, Patient access, Patient behavior, Social | Yes |
| Ndejjo 2016 (239) | To assess the uptake of cervical cancer screening and associated factors among women in rural Uganda. | Descriptive cross-sectional survey | Journal article | Uganda | NR | Region wide study | NR | Demographics, Economic, Patient access, Patient behavior, PCP access, Social | Yes |
| Nega 2018 (240) | To assess the uptake of cervical cancer screening and its associated factors among HIV positive women | Institution- based cross-sectional study | Journal article | Ethiopia | NR | Single center | April 16 to May 15, 2016 | Demographics, Economic, Social | No |
| Nessler 2019 (241) | Our study aims to answer the following questions: 1. What is the PHC patient’s experience with pap smear testing? 2. What is the level of the PHC patient’s willingness to undergo screening at PHC offices? 3. Are there any associations between the professional characteristics of PHC physicians and their patients which could influence the readiness for cytological screening in a PHC setting? | Cross-sectional questionnaire-based survey | Journal article | Poland | NR | Region wide study | Jan to Dec 2017 | Demographics, Economic, Patient access, PCP access, Social | No |
| Ng'ang'a 2018 (242) | This study explored relationship between uptake of cervical cancer screening, socio-demographic, behavioral and biological risk factors. | Nested cross-sectional study | Journal article | Kenya | STEPS survey | National survey | April and June 2015 | Demographic, Economic, Patient access, Patient behavior, Social | No |
| Ngwenya 2018 (243) | This study objectives were to assess the level of knowledge and attitude on cervical cancer and screening among men and women, to determine the practices of women regarding cervical cancer screening and to determine the perceived barriers to cervical cancer screening in Swaziland | Cross-sectional study | Journal article | Swaziland | NR | Community wide study | 27 July to 31 August 2015 | Demographics, Economic, Patient access, Patient behavior, PCP access, Social | No |
| Nigussie 2019 (244) | To assess cervical cancer screening service utilization and associated factors among age-eligible women in Jimma town, South West Ethiopia, 2017. | Cross-sectional study | Journal article | Ethiopia | NR | Town-wide survey | June, 2017 | Demographics, Patient access, Patient behavior, PCP access, Social | No |
| Nunes 2021 (245) | To assess the prevalence and factors associated with cervical cancer screening (CCS) nonadherence in Portugal. | Cross-sectional study | Journal article | Portugal | Portuguese National Health Survey 2014 (2014 NHS) | Nationwide study | 2014 | Demographics, Economic, Patient access, Patient behavior, PCP access, Social | No |
| Nwabichie 2018 (246) | To fill that gap by providing evidence on some of the factors that may be associated with the cervical cancer screening behaviors among this growing population of immigrants in Malaysia | Cross-sectional study | Journal article | Malaysia | NR | Community- wide study | NR | Demographics, Economic, Patient access, Patient behavior, PCP access, Social | No |
| Olesen 2012 (247) | This study addresses several methodological limitations of previous research into the determinants of cervical cancer screening and other preventative health services by combining the strengths of self-report and administrative approaches, We link self-reported psychosocial data with administrative records for screening service use. By doing so we are able to (1) estimate the rates of cervical cancer screening, (2) verify the characteristics thought to be associated with cervical cancer screening, (3) investigate whether these characteristics vary with women’s age, and (4) quantify the effects of these inequalities with greater reliability | Cross sectional study | Journal article | Australia | NR | Province based survey | 2003-2004 | Demographics, Economic, Patient access, Patient behavior, Social | No |
| Oliveira 2014 (248) | To describe the use of cytology for cervical cancer screening by Portuguese women and to identify the determinants of its non-use or underuse in this setting, using data from the 2005/2006 National Health Survey | Cross sectional study | Journal article | Portugal | Fourth National Health Survey | National survey | Between February 2005 and January 2006 | Demographics, Economic, Patient access, Patient behavior, PCP access, Social | No |
| Orang'O 2016 (249) | To identify factors associated with uptake of cervical cancer screening among women seeking care at gynecology clinics in western Kenya. | Cross-sectional study | Journal article | Kenya | Moi University, Moi Teaching and Referral Hospital in Eldoret, Kenya, and the Ministry of Health of Kenya | Multicenter | Apr 2014 -Sep2014 | Demographics, Economic, Patient access, Patient behavior, Social | No |
| Osingada 2015 (250) | To establish the determinants of uptake of CCSS offered at a no-cost clinic managed by female nurse-midwives at a referral hospital in Uganda | Cross sectional survey | Journal article | Uganda | NR | Single center | Daily from Monday to Friday over a period of 30 days in May 2012 | Demographics, Economic, Patient access, Patient behavior, PCP access, Social | Yes |
| Östensson 2015 (251) | To identify possible barriers to and facilitators of cervical cancer screening by (a) estimating time and travel costs and other direct non-medical costs incurred in attending clinic-based cervical cancer screening, (b) investigating screening compliance and reasons for noncompliance, (c) determining women’s knowledge of human papillomavirus (HPV), its relationship to cervical cancer, and HPV and cervical cancer prevention, and (d) investigating correlates of HPV knowledge and screening compliance. | Population based cohort study (Prospective study) | Journal article | Sweden | NR | Multicenter | Mar 2013 to Apr 2014 | Demographics, Economic, Patient access, Social | No |
| Ouk 2020 (252) | To compare the likelihood and frequency of cervical cancer screening in community-dwelling women with and without a history of bipolar disorder or schizophrenia from the general population in Ontario | Retrospective population-based matched case-cohort study | Journal article | Canada | Canadian Institute for Health Information Discharge Abstract Database, National Ambulatory Care Reporting System and Ontario Health Insurance Plan (OHIP) database | Nationwide study | Between 2002 and 2013, Termination of follow-up Dec 15 2015 | Demographics | No |
| Padela 2014 (253) | To assess rates of Papanicolaou (Pap) testing and associations between religion-related factors and these rates among a racially and ethnically diverse sample of American Muslim women | Cross-sectional quantitative survey | Journal article | USA | NR | Community based survey | Between March and June 2012 | Demographics, Patient access, Patient behavior, Social | No |
| Perng 2013 (254) | To investigate promoters and barriers for cervical cancer screening in rural Tanzania. | Cross-sectional study | Journal article | Tanzania | NR | City wide study | Mar 14- Jun 8 2012 | Demographics, Economic, Patient access, Patient behavior, Social | No |
| Petkeviciene 2018 (255) | To estimate the trend in the uptake of cervical cancer (CC) screening in Lithuania during 2006e2014 and to identify sociodemographic and lifestyle factors associated with non-attendance for screening. | Cross-sectional study | Journal article | Lithuania | The data were obtained from five cross-sectional surveys of Lithuanian Health behavior Monitoring | Nationwide study | 2006 to 2014 | Demographics, Economic, Patient behavior, PCP access, Social | No |
| Phaiphichit 2022 (256) | This study aimed to identify factors associated with uptake of cervical cancer screening among women aged 25–60 years | Case control study | Journal article | Laos, Vientiane Capital and Luang Prabang province | NR | Province based survey | March 15 to May 31, 2018 | Demographics, Patient access, Patient behavior, PCP access, Social | No |
| Poliquin 2013 (257) | To describe the population characteristics of women attending Manitoba Pap Test Week clinics, to determine factors associated with women being under-screened prior to attending these clinics, and to discover whether under-screened women attending these clinics are more likely to be rescreened in the future compared to a cohort population | Cohort study | Journal article | Canada | CervixCheck Manitoba registry and an ancillary database of demographic information collected from clinic attendees | Province based survey | 2006 | Demographic, Patient access, Patient behavior, Social | No |
| Pope 2021 (258) | To address this gap of knowledge by using the MHS Data Repository (MDR) to examine the utilization of cervical cancer screening in active duty women according to the USPSTF’s most recent recommendations, and to investigate whether there are differences in screening rates among active duty women of different race (White, Black, Asian, Native American/Alaskan, and Other), and among armed service branches (Army, Air Force, Navy, and Marines). | Retrospective cross-sectional prevalence study | Journal article | USA | MHS Data Repository | Nationwide study | 2011 to 2016 | Demographics, Economic | No |
| Portero de la Cruz 2022 (259) | To examine the evolution of cytology screening adherence from 2017 and 2020 and to identify the factors associated with uptake among women in Spain | Cross-sectional study | Journal article | Spain | 2017 Spanish National Health Survey and the 2020 European Health Survey for Spain | Nationwide study | October 2016–October 2017 and July 2019–July 2020 | Demographics, Economic, Patient access, Patient behavior, PCP access, Social | No |
| Qayum 2021 (260) | To assess the level of knowledge, attitude and practice regarding cervical cancer and VIA screening among women. | Cross-sectional study | Journal article | Bangladesh | NR | District wide study | 2017 | Demographics, Economic, Patient access, Patient behavior, Social | No |
| Ranjit 2016 (261) | To estimate awareness and prevalence of cervical smear testing among women in Nepal | Cross-sectional study | Journal article | Nepal | NR | National survey | May 25 and June 14, 2015 | Demographics, Economic, Social | No |
| Reichheld 2020 (262) | This study aimed that among the women living in our low-income urban community in South India, we sought to determine the prevalence of screening and to assess women’s knowledge of cervical cancer | Community-based cross-sectional survey | Journal article | India | Prevalence Survey (Demographic data and screening prevalence was acquired from women ages 25–65 who live in the low-income urban population of Vellore, Tamil Nadu) | Providence based survey | June to July of 2019 | Demographics, Economic, Social | No |
| Reiter 2015 (263) | To examine Pap testing among these women and their acceptability of HPV self-testing at home, a potential cervical cancer screening strategy. | Cross-sectional study | Journal article | USA | NR | Nationwide study | 2013 | Demographics, Economic, Patient access, Patient behavior, PCP access, Social | No |
| Richard 2015 (264) | This study aimed to examine the relationship between lifestyle and health-related factors and the attendance to CC screening in Switzerland. | Cross-sectional study | Journal article | Switzerland | Swiss Health Survey (SHS) 2012 | National survey | 2012 | Demographics, Patient behavior | No |
| Rosser 2015 (265) | To assess knowledge, personal risk perception, stigma, and screening uptake among women enrolled in the FACES clinic 5 years after the start of the CCSP program. | Cross-sectional study | Journal article | Kenya | NR | Single center | On four separate clinic days over the course of 1 month in April 2013 | Demographics, Patient access, Social | No |
| Rouge 2019 (266) | To gain clearer insight into the characteristics of women who have had at least one Pap smear tests but have not returned after the recommended 3-year interval for a repeat test. | Cross-sectional study | Journal article | France | French nationwide observational survey EDIFICE 6 | Nationwide study | 26 Jun- 28 Jul 2017 | Patient behavior, Social | No |
| Ruddies 2020 (267) | To assess knowledge and attitude towards cervical cancer and its prevention, as well as practice of cervical cancer screening | Cross-sectional community- based study | Journal article | Ethiopia | NR | Community wide study | 2018 | Demographics, Economic, Patient access, Patient behavior, Social | No |
| Sallah 2019 (268) | To characterize cervical cancer screening rates among women tested for HIV or diagnosed with STIs in 5 African countries. In addition, sociodemographic determinants of screening uptake were explored | Cross-sectional study | Journal article | Sub-Saharan Africa (SSA) | Demographic Health Survey (DHS) | International wide study | Cote d’Ivoire (2011), Lesotho (2014), Namibia (2013), Kenya (2014), Zimbabwe (2015) | Demographics, Economic, Patient access, Social | No |
| Sarah 2022 (269) | To determine the prevalence and factors associated with cervical cancer screening among HIV-positive women attending an urban HIV care center in Uganda. | Cross-sectional study | Journal article | Uganda | The AIDS Support Organization (TASO) located at Mulago in Kampala, the capital of Uganda | Single center | 2017 | Demographics, Economic, Patient access, Patient behavior, PCP access, Social | No |
| Sawadogo 2014 (270) | To describe women's practices regarding cervical cancer screening and to assess their knowledge and beliefs. | Cross-sectional study | Journal article | Burkina Faso | NR | State wide study | 1 Dec to 31 Dec 2012 | Demographics, Patient access, Patient behavior | No |
| Seay 2017 (271) | To understand experiences with and preferences for cervical cancer screening among transgender men; the study also sought to understand how sociodemographic characteristics and previous experiences with seeking healthcare might relate to cervical cancer screening history and preferences | Cohort based study | Journal article | USA | National Transgender Discrimination Survey | Community based survey | NR | Demographics, Economic, Patient access, Patient behavior, Social | No |
| Seo 2018 (272) | To identify factors responsible for potentially clinically unnecessary cervical cancer screenings in women with prior hysterectomy. | Retrospective Cross sectional study | Journal article | USA | NA | Nationwide Population-based survey | 2014 | Demographics, Economic, Patient access, PCP access, Social | No |
| Shin 2018 (273) | This study investigated (1) whether cervical cancer screening participation differed by the presence of varying degrees and types of disability; (2) trends in the cervical cancer screening rate relative to disabilities over time; and (3) factors associated with cervical cancer screening | Cohort study | Journal article | Korea | This study linked national disability registration data with national cancer screening program data (National Health Information Database) | National survey | 2006-2015 | Demographics, Economic | No |
| Shrestha 2022 (274) | To investigate knowledge, attitude, preventive practices and utilization of cervical cancer screening among women in a semi-urban area of Pokhara Metropolitan City of Nepal. | Cross-sectional study | Journal article | Nepal | NR | Community wide study | April to June 2019 | Demographics, Economic, Social | No |
| Silvera 2020 (275) | This study seeks to expand the literature to examine the effects of socioeconomic and demographic characteristics on cancer prevention behaviors among a racially/ethnically and geographically diverse population of limited resource women in New Jersey, which has one of the highest cancer rates in the nation. Further, given the limited knowledge about the impact of perception of access to cancer screening and how that influences cancer screening behavior, this study will examine the association of perception of access to screening services on cervical screening behaviors among low-income NHW, NHB, and Hispanic women in New Jersey | Cross-sectional study | Journal article | USA | NR | Community based survey | November 2013 and February 2016 | Demographics, Economic, Patient access, Patient behavior | No |
| So 2017 (276) | To compare the uptake of pap smear tests by the general public and by South Asian women in Hong Kong, and identify the factors associated with the uptake rates of these two groups of women | Cross sectional survey | Journal article | China | NR | Community based survey | NR | Demographics, Patient behavior, PCP access, Social | No |
| Solomon 2019 (277) | To assess predictors of cervical cancer screening practice among HIV-positive women by applying health belief model concepts | Cross-sectional study | Journal article | Ethiopia | NR | Multicenter | 15 Jan to 5 Apr 2018 | Demographics, Economic, Patient access, Patient behavior, Social | No |
| Songsiriphan 2020 (278) | To evaluate the knowledge, attitudes, and practices of HIV-infected women in Thailand with regard to cervical cancer screening and to evaluate the factors that affect whether or not these patients undergo screening. | Cross-sectional study | Journal article | Thailand | NR | Single center | April to November 2019 | Demographics, Economic, Patient behavior, PCP access, Social | No |
| Stenzel 2022 (279) | To compare cervical cancer screening rates by sexual orientation, as well as intersectional analyses with race/ethnicity. | Cross-sectional study | Conference abstract | NR | National health interview survey | Nationwide study | 2015-2018 | Demographics | No |
| Studts 2013 (280) | This study aims were (a) to explore a wide range of barriers preventing middle-aged and older rural Appalachian women from obtaining ICC screening, and (b) to apply the conceptual PRECEDE/PROCEED framework to identify the most frequently endorsed barriers and their sociodemographic correlates | Cross-sectional study | Journal article | USA | NR | Community based survey | 0 | Demographics, Economic, Patient access, Social | Yes |
| Tapera 2017 (281) | The objective of this study was to assess the knowledge and attitudes of University of Botswana female students on cervical cancer screening | Cross-sectional study | Journal article | Botswana | University of Botswana female students | Single center | February, 2016 | Demographics, Patient access, Patient behavior | Yes |
| Tapera 2019b (282) | To investigate socio-demographic inequities in cervical cancer screening and utilization of treatment among women in Harare, Zimbabwe | Cross-sectional study | Journal article | Zimbabwe | NR | City wide study | Jan- Apr 2018 | Demographics, Economic, Patient access, Patient behavior, PCP access, Social | No |
| Tawiah 2022 (283) | To determine the proportion of women screened for cervical cancer, modalities of screening utilized, and factors influencing screening among women in Asokore-Mampong, Ghana, where screening is offered free of charge. | Cross-sectional study | Article in Press | Ghana | NR | Community wide study | Oct to Dec 2018 | Demographics, Economic, Patient access, Social | No |
| Tchounga 2019 (284) | The aim of this study was to estimate the uptake of cervical cancer screening and its correlates among WLHIV in Abidjan, the economic capital of Côte d’Ivoire. | Cross-sectional survey | Journal article | Abidjan, Côte d’Ivoire (Western Africa) | NR | National based survey | May to August 2017 | Demographics, Patient access, Social | No |
| Teame 2019 (285) | To determine factors affecting utilization of cervical cancer screening services among women attending public hospitals in Tigray region in 2018 | Hospital based unmatched case control study | Journal article | Ethiopia | NR | Region wide study | 2018 | Demographics, Economic, Patient access, Patient behavior | No |
| Tekle 2020 (286) | This study is designed to assess knowledge, attitude, and practice towards cervical cancer screening in Wolaita Zone, Southern Ethiopia. | Cross sectional study | Journal article | Ethiopia | NR | Single center | January-February, 2017 | Demographics, Economic, Patient access, PCP access, Social | No |
| Thapa 2018 (287) | To find out the knowledge, attitude, practice, and barriers of cervical cancer screening in mid-western rural, Nepal | Hospital-based cross-sectional study | Journal article | Nepal | NR | Single center | June to September 2017 | Demographics, Patient access, Patient behavior, Social | No |
| Thompson 2020 (288) | To assess information, motivation, and behavioral skills associated with willingness to receive an HPV test instead of a Pap test among women | Cross-sectional study | Journal article | USA | NR | Nationwide study | May to June 2018 | Demographics, Patient access, Patient behavior, PCP access, Social | No |
| Traoré 2020 (289) | To estimate and discuss the role of individual and contextual factors on participation in preventive health-care activities (smear screening) in the Greater Paris area focusing on the characteristics of daily visited neighborhoods in terms of medical densities and social deprivation | Cross-sectional study | Journal article | France | SIRS (a French acronym for “health, inequalities and social ruptures”) | National survey | 2010 | Demographics, Economic, Patient access, PCP access, Social | No |
| Ubah 2022 (290) | This survey examined the barriers to cervical cancer screening uptake by adult women in Nnewi, a town located in southeast Nigeria | Cross-sectional study | Journal article | Nigeria | The Health Belief Model Scale | Town wide study | NR | Economic, Patient access, Patient behavior, PCP access | No |
| Vajda 2022 (291) | To explore knowledge, habits and motivation to cervical cancer screening among women aged between 25 and 65 years. | Cross-sectional study | Conference abstract | Hungary | NR | Nationwide study | 2021 | Demographics, Patient access, Social | No |
| Visanuyothin 2015 (292) | To examine the determinants of cervical cancer screening adherence in urban areas of Nakhon Ratchasima Province, Thailand | Cross-sectional survey | Journal article | Thailand | NR | Province based survey | 2007 to 2009 | Demographics, Economic, Patient access, Patient behavior, Social | No |
| Vora 2020 (293) | To understand the knowledge/awareness and practices of the community, to understand the predictors of cervical cancer screening, and to assess the acceptance of the HPV test in the community. | Cross-sectional study | Journal article | India | NR | City wide study | Aug 2018- Oct 2019 | Demographics, Economic, Social | No |
| Watson 2017 (294) | This study examined national percentages of cervical cancer screening, and we examined use of co-testing as an option for screening | Cross-sectional study | Journal article | USA | The 2015 U.S. National Health Interview Survey (NHIS) | National survey | 2015 | Demographic, Economic, Patient access, Social | No |
| Weitlauf 2013 (295) | The author we examined the association between PTSD and cervical cancer screening in cross sectional study capitalizing on a large national sample of women veterans using VHA facilities for healthcare between 2003 and 2007. To evaluate our hypothesis that women with PTSD would be at highest risk for under-screening (no screening over three consecutive years), we compared their receipt of cervical cancer screening to that of VHA female patients with depression, and to VHA female patients with no psychiatric conditions. To evaluate our hypothesis that heavy use of primary care services could offset the effects of psychiatric illness (e.g., PTSD or depression) on screening, we evaluated the interaction of psychiatric diagnosis (PTSD or depression) and primary care use on women’s receipt of cervical cancer screening during the study observation period | Cross-sectional study | Journal article | USA | VHA National Patient Care Database | National survey | October 2003-September 2004 | Demographics | No |
| Woldetsadik 2020 (296) | To determine the influence of sociodemographic characteristics and related factors on screening. | Cross-sectional study | Journal article | Ethiopia | NR | Single center | Jul - Sepr 2017 | Demographics, Economic, Patient access, Patient behavior, PCP access, Social | No |
| Wongwatcharanukul 2014 (297) | The purpose of this cross-sectional study was to study factors related to cervical cancer screening uptake by Hmong hilltribe women in Lomkao District, Phetchabun Province | Cross-sectional study | Journal article | Thailand | NR | District wide study | 40969 | Demographics, Patient access, Patient behavior, Social | No |
| Zhang 2020 (298) | To disentangle the association of cervical cancer screening with healthcare access and HIV testing among women at a high risk of HIV infection. | Cross-sectional telephone survey | Journal article | USA | 2016 Behavioral Risk Factor Surveillance System | National survey | 2016 | Demographics, Patient access | No |
| **Colorectal cancer** | | | | | | | | | |
| Ahmed 2013 (299) | This study explored factors associated with racial/ethnic differences in rates of screening recommendation. | Cross-sectional study | Journal article | USA | National health interview survey (NHIS) | Nationwide study | 2000 | Demographics, Economic, Patient access, PCP access, Social | No |
| Almadi 2019 (300) | To examine the acceptance of the public to undergo CRC screening and to explore potential barriers to CRC screening using the Health Belief Model (HBM), through a nationwide survey using an electronic platform to assess possible uptake of screening if a national program would be launched in Saudi Arabia. | Cross-sectional study | Journal article | Saudi Arabia | NR | Nationwide study | NR | Demographics, Economic, Patient access, Patient behavior, Social | No |
| Azimi 2020 (301) | To determine the uptake of CRC screening among American-born and foreign-born adults (age ≥ 45) in the USA and to evaluate the association of the highest formal education attained with CRC screening. | Cross-sectional study | Journal article | USA | 2018 Health Information National Trends Survey (HINTS) 4 cycle 3 | Nationwide study | 2018 | Demographics, Economic, Patient behavior, Social | No |
| Bae 2014 (302) | To explore strong factors linked to repeated participation in FOBT in the prior decade (2002-2011) among adults using the Health Belief Model (HBM) after controlling for other covariates | Cross sectional study | Journal article | Republic of Korea | NR | City-wide survey | October 29 to the December 31, 2011 | Demographics, Economic, Patient behavior, Social | No |
| Bardach 2012 (303) | This study sought to determine the relationship between colorectal cancer screening knowledge, specifically regarding recommended screening intervals, and receipt of screening among residents of rural Appalachian Kentucky | Cross-sectional study | Journal article | USA | NR | State wide study | November 20, 2009 and April 22, 2010 | Demographics, Economic, Patient access, Social | No |
| Bernardo 2018 (304) | To assess individual and neighborhood-level predictors of guideline-concordant CRC screening within two cohorts of individuals located within CRC mortality geographic hotspot regions in the U.S. | Prospective cohort study | Journal article | USA | Southern community cohort study and Ohio Appalachia CRC screening study | Multicenter | 2009-2013 | Demographics, Economic, Patient access, Patient behavior, Social | No |
| Bhimla 2020 (305) | To determine whether neighborhood walkability and transit accessibility were associated with CRC screening among underserved Vietnamese Americans. | Cross-sectional study | Conference abstract | USA | NR | Community wide study | NR | Demographics, Economic, Patient access, Patient behavior | No |
| Bocci 2017 (306) | The aim of our study was to investigate the beliefs, the feelings and the psychological factors that could influence the participation of women in colorectal cancer screening | Cross-sectional study | Journal article | Italy | NR | Single center | October-December 2011 | Demographics, Economic, Patient behavior, PCP access | No |
| Brandt 2012 (307) | The aim was to describe the association of awareness and knowledge with participation in colorectal cancer (CRC) screening. | Cross-sectional study | Journal article | USA | Telephone survey research | State wide population based | 2009 | Demographics, Economic, Patient access, Social | No |
| Brown 2015 (308) | To determine whether current breast and cervical cancer screening behaviors remain consistent with the high rates observed in 2006, to identify current colorectal cancer (CRC) screening practices and to ascertain whether the HCSS program has positively influenced CRC screening behavior among Hopi women. | Cross-sectional, population-based survey study | Journal article | USA | NR | Community based survey | 2012 | Demographics, Economic, Patient access, Patient behavior, PCP access, Social | No |
| Bui 2018b (309) | This study aimed to investigate the stages of adopting CRC screening in Korea according to screening modality. In this study, we applied the TTM to examine the distribution of and factors associated with stages of adoption for FOBT and colonoscopy for CRC screening in Korea. | Cross sectional study | Journal article | Korea | Korean National Cancer Screening Survey | National survey | 2014 | Demographics, Economic, Patient access, Patient behavior, Social | No |
| Bujang 2021 (310) | To determine factors associated with colorectal cancer screening using iFOBT among the average risk Malaysian population | Cross-sectional study | Journal article | Malaysia | NR | Multicenter | May 1, 2019- Jul 31, 2019 | Demographics, Patient access, Patient behavior, PCP access | No |
| Buron 2017 (311) | To describe the distribution of the main outcomes of the first round of a population-based Barcelona colorectal cancer screening program (BCRCSP) by socioeconomic level, taking into account age and sex | Cohort study | Journal article | Spain | Barcelona colorectal cancer screening program (BCRCSP) | City-wide community based survey | Between 12.01.2009 and 12.31.2011 | Demographics, Economic | No |
| Bynum 2012 (312) | To Identify the influence of medical mistrust, fears, attitudes, and sociodemographic characteristics on unwillingness to participate in colorectal cancer (CRC) screening. | Cross-sectional study | Journal article | USA, Puerto Rico | NR | Nationwide study | NR | Demographics, Economic, Patient access, Patient behavior, Social | No |
| Calo 2015 (313) | To examine the associations between multiple tract-level socioeconomic measures and adherence to colorectal cancer screening (CRCS) in Harris County and the City of Houston, Texas. | Cross-sectional multilevel study | Journal article | USA | 2010 Health of Houston Survey | Country wide population based study | 2010 | Demographics, Economic, Patient access, Social | No |
| Cardoso 2020 (314) | Aimed at providing harmonized and highly comparable data across EU countries to support health policies and address health inequalities and social exclusion | Cross-sectional study | Journal article | Europe | EHIS study | International wide study | 2013 to 2016 | Demographics, Economic, Patient behavior, PCP access, Social | No |
| Castañeda-Avila 2021 (315) | To access current estimates of colorectal cancer (CRC) screening practices in relation to cardiovascular disease (CVD) status, and whether this association varies by race/ethnicity. | Cross-sectional study | Journal article | USA | Behavioral Risk Factor Surveillance System data | National survey | 2012, 2014, 2016 and 2018 | Patient's access | No |
| Cataneo 2022 (316) | To analyze the association between LEP and rates of colorectal cancer screening. | Retrospective cohort study | Journal article | USA | NHIS survey database | Nationwide study | NR | Demographics, Economic, Patient access, PCP access | No |
| Chaiarch 2021 (317) | To investigate factors associated with participation in CRC screening using a FIT in the Thai population age 45 to 74 years in the Namphong District of Khon Kaen Province | Case-control study | Journal article | Thailand | NR | City wide study | Mar 26- Apr 10, 2018 | Demographics, Patient behavior, PCP access, Social | No |
| Chan 2022a (318) | To examine current CRC screening uptake in Hong Kong and identify the factors associated with it using Andersen’s behavioral Model as a guiding framework. | Cross-sectional study | Journal article | Hong Kong | NR | Region wide study | Aug2019- Dec 2020 | Demographics, Economic, Patient access, Patient behavior, PCP access, Social | No |
| Changoor 2018 (319) | To use administrative insurance claims data to classify patterns of CRC screening within a national subset of the US population that is universally insured from the age of 50 years with a specific focus on potential differences in screening rates between black and white patients | Cross-sectional, descriptive study | Journal article | USA | Claims data from TRICARE (insurance coverage for active, reserve, and retired members of the US Armed Services and their dependents) provided by the Department of Defense | National survey | 2007-2010 | Economic, Patient access | No |
| Chatterjee 2015 (320) | To investigate the incidence and determinants of colorectal cancer (CRC) and its screening in District of Columbia (DC) and identify modifiable risk factors. | Cross-sectional study | Journal article | USA | (1) the Washington DC cancer registry (DCCR) (years 2000–2009); (2) the Behavioral Risk Factor Surveillance System (BRFSS 2002–2008); and (3) the US Census Bureau. Data from Surveillance Epidemiology and End Results (SEER) | Nationwide study | 200-2009 | Demographics, Economic, Patient access, Social | No |
| Chido-Amajuoyi 2019 (321) | To examine the prevalence and correlates of CRC screening using FOBT/FIT in physicians' office vs at home | Cross-sectional study | Journal article | USA | Cancer Control Supplement of the 2015 National Health Interview Survey | Nationwide study | 2015 | Demographics, Patient access, Patient behavior, Social | No |
| Choi 2018c (322) | To identify moderators of cancer worry and perceived risk for developing CRC among several known risk factors. | Cross-sectional study | Journal article | Korea | Korean National Cancer Screening Survey (KNCSS) | Nationwide study | 2013 | Demographic, Patient access, Patient behavior | No |
| Chuang 2021 (323) | This study focused on the factors influencing whether a patient with a CRC diagnosis chooses to undergo referral follow-up. | Cross-sectional study | Journal article | Taiwan | NR | District wide study | NR | Demographics | No |
| Chudy-Onwugaje 2020 (324) | To investigate the association between prior incarceration and adherence to CRC screening in average-risk AA men in the US. | Cross-sectional study | Journal article | USA | NR | Single center | NR | Demographics, Economic, Patient access, Patient behavior, PCP access, Social | No |
| Clarke 2021 (325) | This study aimed the associations between uptake of FIT-based screening and (i) colorectal cancer knowledge and health literacy, (ii) cancer beliefs and emotional attitudes to cancer and FIT-based screening and (iii) social influences. Our secondary aim was to determine if these associations differed between consistent versus inconsistent screening participants | Cross-sectional study | Journal article | Ireland | Tallaght Hospital/Trinity College Dublin Colorectal Cancer Screening Program (TTC-CRC-SP) | City-wide survey | September 2015 | Demographics, Economic, Patient access, Patient behavior, Social | No |
| Cofie 2020 (326) | To examine gender and racial/ethnic variations in CRCS, including the impact of insurance, self-rated health, length of US residency and citizenship status, among foreign-born older adults in the US. | Cross-sectional study | Journal article | USA | National Health Interview Survey (NHIS) | Nationwide study | 2018 | Demographic, Patient access | No |
| Cohen 2012 (327) | To examine whether body mass index (BMI) is associated with reduced colorectal cancer (CRC) screening in a large population of black and white adults | Cross-sectional study | Journal article | USA | NA | Multicenter (Community health centers) | 2002–2009 | Demographics | No |
| Courtney 2013a (328) | To determine the proportions and predictors of first-degree relatives (FDRs) of colorectal cancer (CRC) patients (i) ever receiving any CRC testing and (ii) receiving CRC screening in accordance with CRC screening guidelines. | Cross-sectional, population based study | Journal article | Australia | Population-based Victorian Cancer Registry, Victoria, Australia | State wide study | 2009 to 2011 | Demographics, Economic, Patient access, PCP access, Social | No |
| Courtney 2013b (329) | The purpose of the current study was to identify factors associated with (i) ever receiving colorectal cancer (CRC) testing; (ii) risk-appropriate CRC screening in accordance with guidelines; and (iii) recent colonoscopy screening | Prospective Longitudinal cohort study | Journal article | Australia | Hunter Community Study (HCS) participants were randomly selected from the NSW State electoral roll | Region wide study | December 2004 and December 2007 | Demographics, Economic, Patient access, Patient behavior, PCP access, Social | No |
| Davis 2017 (330) | To investigate the role of the emotion of disgust as a predictor of screening intention additional to embarrassment and fear | Cross-sectional study | Journal article | Australia | NR | Country wide study | Dec 1, 2013- Mar 31, 2014 | Demographics, Patient access, Patient behavior | No |
| Decker 2015b (331) | To examine the trends in colorectal cancer (CRC) screening (fecal occult blood test (FOBT), colonoscopy, and flexible sigmoidoscopy (FS)) and differences in CRC screening by income in a population with an organized CRC screening program and universal health-care coverage. | Cross-sectional study | Journal article | Canada | Provincial Physician Claims database and the population-based colon cancer screening registry | Provence wide study | 1995-2012 | Economic | No |
| Decker 2016 (332) | This study examined organized colorectal cancer (CRC) screening program and non-program fecal occult blood test (FOBT) use from 2008 to 2012 for individuals living in Winnipeg, Manitoba, by area level income | Cohort study | Journal article | Canada | Manitoba Health Population Registry, the Medical Claims Database, the ColonCheck Registry, and Statistics Canada 2006 census data | Province based survey | 2008-2012 | Economic | No |
| Deding 2019 (333) | This study examines the participation of men and women with health educations compared to other educations | Cross-sectional population study | Journal article | Denmark | NR | National survey | 2014-2015; Follow-up until July 2016 | Social | No |
| Diaz 2013 (334) | To examine the association between Latino race/ethnicity, gender, and English proficiency and CRC screening uptake. | Cross-sectional study | Journal article | USA | Behavioral Risk Factor Surveillance System (BRFSS) | National survey | 2008 | Demographics | No |
| Dodd 2019 (335) | To investigate the relationships between PLE and each of: 1) intention to participate in FOBt screening in the future, 2) ‘ever’ uptake of FOBt screening, and 3) repeated uptake of FOBt screening for colorectal cancer. | Cross-sectional study | Journal article | UK | Second wave of the Attitudes, behavior and Cancer UK Survey (ABACUS II) | National based survey | April 2015. | Demographics | No |
| Douma 2019 (336) | To examine the relationship between people’s goal-orientation or focus on advantages or disadvantages and their CRC screening participation, as this could provide insights for supporting people in making this complex decision. | Cross-sectional study | Journal article | Netherlands | NR | Nationwide study | 42795 | Demographics, Patient behavior, Social | No |
| Eke 2019 (337) | To quantify the segment of the CRC screening-eligible population that had passed through inpatient admission setting and received screening for CRC during hospitalization with any CRC screening methods from 2005 to 2014, in the USA. Predictors of inpatient CRC screening will also be determined. | Cohort based study | Journal article | USA | Nationwide Inpatient Sample HCUP National Inpatient Sample (NIS), 2011 database | National survey | 2005 to 2014 | Demographics, Economic, Patient access | No |
| Elangovan 2021 (338) | To assess the FIT completion rates and adenoma detection rate (ADR) of positive FIT-colonoscopy (FIT-C) in an urban safety-net system | Cross-sectional study | Journal article | USA | Electronic health records of The MetroHealth System | Province based survey | Between September 01, 2017 and August 30, 2018 | Demographics, Economic, Patient access, Patient behavior, PCP access | No |
| Ellis 2018 (339) | To examine whether the relation between “I don’t know” (DK) response and colorectal cancer (CRC) screening behavior differed, depending on the construct queried (knowledge vs. beliefs). | Cross-sectional study | Journal article | USA | Awareness and Beliefs about Cancer-a population-based telephone survey | Nationwide study | NR | Demographics, Economic, Patient access, Patient behavior | No |
| Eze 2019 (340) | To determine uptake of CRC screening (with fecal occult blood test or endoscopy)among US-born and foreign-born adults in the United States and evaluate the relationship of the highest formal education attained on this relationship. Methods | Cross-sectional study | Journal article | USA | 2013 Health Information National Trends Survey (HINTS)4 cycle 3 | Nationwide study | 2013 | Demographics | No |
| Farr 2022 (341) | To explore which demographic characteristics, healthcare access factors, and cancer-related beliefs were associated with colorectal cancer screening completion among U.S. and foreign-born women adherent to mammography screening recommendations. | Cross-sectional study | Journal article | USA | 2015 National Health Interview Survey | Nationwide study | 2019 | Demographics, Economic, Patient access, Patient behavior, Social | No |
| Fawns-Ritchie 2022 (342) | This study examined whether psychological factors were associated with CRC screening uptake. | Cross sectional observational study | Journal article | Scotland | Healthy Ageing In Scotland (HAGIS) pilot study, | National survey | 2016-2017 | Demographics, Patient behavior, Social | No |
| Fiala 2022 (343) | To determine what factors are associated with CRC screening recommendation adherence among FDRs of individuals with CRC. | Cross-sectional study | Journal article | USA | 2015 National Heath Information Survey (NHIS) Cancer Control Supplement (CCS) | Nationwide study | 2015 | Demographics, Economic, Patient access, Social | No |
| Gale 2015 (344) | This study investigated whether participation in screening varies according to cognitive ability and personality. In addition, it examined whether cognitive ability helps to explain the link between health literacy and screening. | Cohort study | Journal article | UK | English Longitudinal Study of Ageing (ELSA) | National survey | 2002-2003, 2010-2011 | Economic, Patient behavior, Social | No |
| Ghai 2020 (345) | To assess the primary care provider (PCP) perceptions of colorectal cancer (CRC) screening test effectiveness and their recommendations for testing intervals influence patient screening uptake | Cohort study | Journal article | USA | NR | Province based survey | Between 2017 and 2018 | PCP access | No |
| Gofine 2018 (346) | To assess use of colorectal cancer screening (CRCS) as per United States Preventive Task Force guidelines among people with mobility disability using a nationally representative data set | Cross-sectional study | Journal article | USA | The 2013 National Health Interview Survey | National survey | 2013 | Demographics | No |
| Gonzalez 2020 (347) | To determine the rate of CRCS and to identify barriers and facilitators to screening in the Hispanic population of Flint, Michigan | Cross-sectional study | Journal article | USA | NR | Community wide study | Nov 2017- May 2018 | Demographics, Patient access, PCP access | No |
| Gray 2021 (348) | To determine colorectal cancer (CRC) screening knowledge, attitudes, behaviors, and preferences for a future CRC screening educational intervention among adults (companions) waiting for outpatients undergoing a colonoscopy | Cross-sectional study | Journal article | USA | NR | Multicenter | Mar 2017 to Jul 2017 | Demographics, Patient access, Patient behavior, Social | No |
| Greene 2012 (349) | The objective of this study was to examine population characteristics relevant to the design and implementation of a state-sponsored colorectal cancer screening program that is responsive to medically underserved populations. | Cross-sectional, observational study | Journal article | USA | NR | State wide survey | 2006 | Demographics, Economic, Patient access, PCP access, Social | No |
| Halbert 2016 (350) | To evaluate the association between social determinants (e.g., psychological characteristics, perceived social environment, cultural beliefs such as present temporal orientation) and colorectal cancer (CRC) screening among African Americans | Cross-sectional study | Journal article | USA | NR | Community based survey | 2010 | Demographics, Economic, Patient access, Patient behavior, PCP access, Social | No |
| Hategekimana 2016 (351) | To examine the association of self-perceived mental health status (SPMH) and FOBT uptake for CRC screening using a population-based, national health survey of Canadians | Cross-sectional study | Journal article | Canada | Canadian Community Health Survey (CCHS) | National survey | 2011-2012 | Demographics, Social | No |
| He 2020 (352) | To identify factors associated with colonoscopy compliance based on Health Belief Model (HBM). | Prospective cohort study | Journal article | China | NR | Community wide study | 2015 | Patient access, Patient behavior, PCP access, Social | No |
| Homayoon 2013 (353) | The aims of this study were to compare CRCS between Asians and whites and to evaluate for clinical predictors of CRCS | Cross-sectional population based study | Journal article | USA | California Health Interview Survey | State wide study | 2007 | Demographics, Economic, Patient behavior, PCP access, Social | No |
| Horner-Johnson 2014b (354) | To determine whether urban/rural disparities in colorectal cancer screening exist among people with disabilities, similar to the disparities found in the general population. | Cross-sectional study | Journal article | USA | Medical Expenditure Panel Survey (MEPS) | Country wide study | 2002-2008 | Demographics, Economic, Patient access, Social | No |
| Huang 2019a (355) | To determine the uptake rate, barriers and predictors of CRC screening among cancer survivors. | Cross-sectional study | Journal article | Singapore | National University Cancer Institute, Singapore | Single center | Jul 2013 to Sep 2016 | Economic, Patient behavior, PCP access | No |
| Huang 2019b (356) | To evaluate the effectiveness of a pilot program for colorectal cancer screening on increasing the enabling factors of screening in a large Chinese population, and identified factors associated with its participation based on variables pertinent to the Health Belief Model. | Cross-sectional study | Journal article | China | NR | Territory wide study | 2016, 2017, and 2018 | Demographics, Economic, Patient access | No |
| Huang 2019c (357) | To investigate the social-demographic factors associated with readiness to CRC screening using the SOC | Cross-sectional study | Journal article | NR | NR | NR | NR | Demographics, Social | No |
| Huang 2020a (358) | To examine whether the theory of planned behavior (TPB) could predict the uptake of fecal immunochemical test to inform novel strategies for enhancing CRC screening participation in population-based programs | Cross-sectional study | Journal article | China | NR | National based survey | October 2017 to November 2018 | Demographics, Economic, Patient behavior, Social | Yes |
| Huang 2020b (359) | To inform strategies to enhance the CRC screening uptake this study examined the its participation rate and evaluated the association between socio-demographics/health-related factors and CRC screening uptake among Chinese population. | Cross-sectional study | Journal article | Hong Kong | NR | Territory wide study | 2017-2018 | Patient's access | No |
| Huang 2021a (360) | To assess the awareness, attitude and barriers of colorectal cancer screening among high-risk populations in China. | Cross-sectional study | Journal article | China | Nine hospitals from Hunan province | Multicenter | April - August 2019. | Demographics, Patient access, Patient behavior, PCP access | No |
| Huang 2021c (361) | To evaluate how the knowledge and perception towards colorectal cancer (CRC) screening had been changed in a large population, and identified factors associated with its participation based on factors pertinent to the Health Belief Model (HBM). | Cross-sectional study | Journal article | Hong Kong | NR | Region wide study | Dec 2016 to Nov 2018 | Demographics, Economic, Patient access, Patient behavior, PCP access, Social | No |
| Hughes 2015 (362) | To investigate reasons for lower CRC screening rates among Nebraska residents, especially among rural residents. | Cross-sectional study | Journal article | USA | NR | Community wide study | 2014 | Demographics, Economic, Patient access, Patient behavior, PCP access | No |
| Hughes 2018 (363) | To highlight how geospatial EHR data linkages can enrich patient-level data employed in colorectal cancer screening research | Retrospective cohort study | Journal article | USA | The Parkland Hospital and Health System (hereafter, “Parkland"), the safety-net healthcare system in Dallas County, Texas | Single center | January 1, 2010, and July 31, 2012 | Demographics, Economic, Patient access, PCP access, Social | No |
| Idowu 2016a (364) | This study investigated the association of place of birth (US- versus foreign-born) and cancer beliefs with being current with CRC screening guidelines using FOBT, sigmoidoscopy and colonoscopy. | Cross-sectional study | Journal article | USA | Health Information National Trends Survey | National survey | 2007 | Demographics, Patient behavior | No |
| Ilgaz 2018 (365) | To determine CRC risk levels, status of participation in CRC screenings, and the factors affecting participation in screenings among individuals between 50 and 70 years old working in agriculture | Cross-sectional study | Journal article | Turkey | NR | Community wide study | July and October 2014 | Demographics, Patient access, Patient behavior, Social | No |
| Jin 2019a (366) | This study investigated factors that enable KAs to adhere to CRC screening guidelines using Andersen’s Behavioral Model of Health Services Utilization. | Cross sectional survey | Journal article | USA | NR | Community based survey | May 2015 and February 2016 | Demographics, Economic, Patient access, Patient behavior, PCP access, Social | No |
| Jin 2019b (367) | To examine factors predicting decisional stage of CRC screening adoption among older KAs using the precaution adoption process model | Cross sectional survey | Journal article | USA | NR | Community based survey | Between May 2015 and February 2016 | Demographics, Patient access, Patient behavior, PCP access | No |
| Jun_ 2013 (368) | To explore fatalistic attributions of colon cancer development among Asian and Hispanic Americans in comparison with non-Hispanic whites; also to examine the impacts of fatalism on adherence to the colon cancer screening guideline | Cross-sectional study | Journal article | USA | The 2005 Health Information National Trends Survey | National survey | 2005 | Patient behavior | No |
| Juon 2018 (369) | To investigate the change in cervical cancer screening rates, the level of socioeconomic disparities in cervical cancer screening participation, and whether there was a reduction in these disparities between 1998 and 2010 | Cross-sectional study | Journal article | Korea | Korean Health | Nation wide | 1990 - 2010 | Demographics, Economic, Patient access, Social | No |
| Kang 2017 (370) | To determine the behaviors for and predict the factors influencing self-reported colorectal cancer screening in Korean adults. | Cross-sectional, observational study | Journal article | Korea | 2012 Community Health Survey conducted by the Korean Centers for Disease Control and Prevention. | Nationwide study | Aug to Oct 2012 | Demographics, Economic, Patient access, Patient behavior, Social | No |
| Kearns 2018 (371) | To examine the association between having an LTC and uptake of CRC screening in England with the guaiac fecal occult blood test, with a particular focus on common mental disorders | Preregistered secondary analysis of two cohorts | Journal article | UK | Yorkshire Health Study (YHS) and the National Health Service; National Bowel Cancer Screening Program (BCSP, years 2006–2014) and national English Longitudinal Study of Ageing (ELSA, years 2014–2015) | Nationwide survey | National Health Service National Bowel Cancer Screening Program (BCSP, years 2006–2014), the national English Longitudinal Study of Ageing (ELSA, years 2014–2015) | Demographics | No |
| Kendall 2013 (372) | The objectives of this study were (1) to describe CRC screening rates by BMI status with a null hypothesis of equal rates of CRC screening by obesity status and (2) to evaluate if obesity is a predictive factor for CRC screening among Medicare beneficiaries in the US | Cross-sectional study | Journal article | USA | The 2005 Medicare Current Beneficiary Survey (MCBS) | National survey | 2005 | Demographics, Economic, Patient access, Social | No |
| Khoja 2018 (373) | To measure colorectal cancer screening (CRCS) utilization in Saudi Arabia ’s elderly population and to assess the factors associated with CRCS | Cross-sectional study (Population based) | Journal article | Saudi Arabia | Saudi national survey | Nationwide study | 2006 to 2007 | Demographics, Economic, Patient access, Patient behavior, PCP access, Social | No |
| Klabunde 2015 (374) | To provide insights about the screening-eligible population ages 76-84 that could be used to inform strategies to facilitate patient-centered discussions about CRC screening. | Cross-sectional study | Journal article | USA | Nationwide household survey of a representative sample of the U.S. civilian | Nationwide study | 2010 | Demographics, Economic, Patient access, PCP access, Social | No |
| Knight 2015 (375) | The objective of this study was to provide information for state, regional, and local-level partners throughout Kentucky and in states with similar populations that can be used to develop and implement effective strategies to reduce these barriers and increase CRC screening rates | Cross-sectional study | Journal article | USA | KyBRFSS (Kentucky Behavioral Risk Factor Surveillance System survey) | State wide survey | 2008 and 2012 | Demographics, Economic, Patient access, Social | No |
| Kobayashi 2014 (376) | To determine the association between health literacy and participation in publicly available colorectal cancer (CRC) screening in England using data from the English Longitudinal Study of Ageing (ELSA) | Prospective longitudinal cohort study | Journal article | England | English longitudinal study of aging | Nationwide study | 2010 to 2011 | Demographics, Economic, Social | No |
| Koo 2012 (377) | This study investigated the association of screening test participation with knowledge of, attitudes toward, and barriers to CRC and screening tests in different cultural and sociopolitical contexts. | Cross-sectional study | Journal article | Hong Kong, Australia, Brunei, China, India, Indonesia, Japan, Korea, Malaysia, Pakistan, Philippines, Singapore, Taiwan, and Thailand | NR | Multicenter (Study setting; author reported) | 2007 | Demographics, Economic, Patient access, Patient behavior, PCP access, Social | No |
| Kroupa 2019 (378) | To acquire independent opinions from the target population for CRC screening regarding CRC screening information sources and the reasons for and against participation in CRC screening. Clients’ personal feelings regarding screening perceptions and potentially problematic points in the screening process were also surveyed. The effect of certain factors on initial and longitudinal screening uptake was assessed | Cross sectional study | Journal article | Czech Republic | NR | Community based survey | 2013 to 2015 | Demographics, Patient access, Patient behavior, Social | No |
| Laiyemo 2019 (379) | To determine uptake of CRC screening (with fecal occult blood test or endoscopy) among adult men and women in the United States and evaluate whether screening uptake differs by sex after a diagnosis of any cancer. | Cross-sectional study | Journal article | USA | 2013 Health Information National Trends Survey (HINTS) 4 cycle 3 | State wide study | 2013 | Demographics, Economic, Patient access, Social | No |
| Le 2012 (380) | To determine whether physician-related factors influenced patient participation in colorectal cancer (CRC) screening programs and to identify patient characteristics associated with lower participation in order to facilitate the development of targeted actions to improve participation | Retrospective cohort study | Journal article | France | Statutory health insurance (SHI) program | State wide study | June 2007- May 2010 | Demographics, Economic, PCP access | No |
| Lee 2018b (381) | To assess socio-demographics, access to health care, health and cultural beliefs, and FOBT utilizations for CRC screening, and to identify factors associated with FOBT among KAs aged 50 and older | Cross-sectional survey | Journal article | USA | Community-based organizations such as Korean churches and community centers in the Chicago metropolitan area | Community based survey | NR | Patient access, Patient behavior | No |
| Lee 2018d (382) | The aim of this study was to (a) assess CRC screening rates, including fecal occult blood test (FOBT), flexible sigmoidoscopy, and colonoscopy and (b) explore factors related to these tests among KAs by location of CRC screening. M | Cross-sectional study | Journal article | USA | NR | Community wide study | NR | Demographics, Economic, Patient access, Social | No |
| Lee 2019b (383) | To describe the influence of sociodemographic, health characteristics, health beliefs, and spousal support on CRC screening practices, CRC screening utilization rates, and predictors for CRC screening utilization among Thai immigrants in the United States. | Cross-sectional study | Journal article | USA | NR | Community wide study | NR | Demographics, Patient access, Patient behavior, Social | No |
| Leung 2016 (384) | To describe the proportion of the use of CRC screening by community-dwelling older people and to examine the determinants of participation in CRC screening based on the theoretical model | Cross sectional survey | Journal article | China | NR | Community based survey | May-July in 2012 | Demographics, Economic, Patient access, Patient behavior, Social | No |
| Lin 2013 (385) | To explore the prevalence of CRC screening and related factors in rural south Taiwan | Cross-sectional study | Journal article | Taiwan | NA | Region wide study (based on 2 rural areas in Taiwan, Kaohsiung and Pintung) | NR | Demographics, Economic, Patient access | No |
| Lin 2017 (386) | To ascertain national CRC screening rates and to explore the relations between sociodemographic characteristics and patient-provider communication on the receipt of CRC screening among HC patients. | Cross-sectional study | Journal article | USA | Health Center Patient Survey data | Multicenter | 2014 | Demographics, Economic, Patient access, PCP access | Yes |
| Llanos 2015 (387) | To investigate whether CRC worry was associated with screening and determine whether CRC worry modified the associations between other covariates and being within CRC screening guidelines. An additional aim of this study was to determine the correlates of higher levels of CRC worry. | Cross-sectional study | Journal article | USA | NR | Region wide study | Sep 2009 - Mar 2010 | Demographics, Economic, Patient access, Patient behavior, PCP access, Social | No |
| Lo 2015 (388) | This study examined if and how sociodemographic differences in colorectal cancer (CRC) screening uptake can be explained by social cognitive factors | Cross-sectional study | Journal article | UK | The data were collected as part of a TNS Research International population-based omnibus survey | International survey | January and March 2014 | Demographics, Economic, Patient behavior | No |
| Majeed 2022 (389) | To examine differences in cancer screening behaviors based on Muslim affiliation | Prospective Longitudinal cohort study | Article in Press | USA | Chicago Multiethnic Prevention and Surveillance Study (COMPASS) | State wide study | NR | Demographics, Patient access | No |
| Maly 2014 (390) | To describe the demographics, health status, prevalence of modifiable CRC risk factors, and use of CRC screening modalities in a Pacific Northwest AI tribe. | Cross-sectional study | Journal article | USA | Behavioral Risk Factor Surveillance System (BRFSS) questionnaire | Community wide study | 2010 | Demographics, Patient access | No |
| Mansfield 2018 (391) | To provide more detailed information on preferences for the features of CRC screening tests and how those preferences and reported screening behavior correlate with personal characteristics. | Cross-sectional study | Journal article | USA | 2010 National Health Interview Survey | Nationwide study | Sep 2014 to Feb 2015 | Demographics, Economic, Patient access, Patient behavior, Social | No |
| Mansouri 2013 (392) | To examine, in an area of multiple deprivation, the impact of age, sex and socioeconomic deprivation not only on uptake, but throughout all stages of the screening process | Cohort study | Journal article | Scotland | Database held by Public Health Screening Unit in NHS GG&C | National survey | April 2009 to March 2011 | Demographics, Economic | No |
| Marucci 2022 (393) | To describe whether the racial disparities in CRC screening rates persist for Hispanics who have reliable access to PC. | Cross-sectional study | Journal article | USA | 2018 Behavior Risk Factor Surveillance System dataset | Nationwide study | 2018 | Demographics, Economic, Patient access | No |
| Mastrokostas 2018 (394) | To compare the views of healthy eligible unscreened adults, to those of primary care providers in Greece, about colorectal cancer (CRC) screening perceived barriers. | Cross-sectional study | Journal article | Greece | EMENO (National Morbidity and Risk Factors Survey) | National survey | April 2014 to November 2015 | Patient access, Patient behavior, PCP access | No |
| May 2017 (395) | To examine CRC screening rates for Veterans that pursue healthcare from various coverage settings and to determine whether there are associations between the type of primary healthcare coverage a Veteran uses and CRC screening status. A second aim was to determine additional demographic and health-related predictors of CRC screening uptake among Veterans. | Cross-sectional study | Journal article | USA | Centers for Disease Control and Prevention’s (CDC) Behavioral Risk Factor Surveillance System survey | National survey | 2014 | Demographics, Economic, Patient access, PCP access, Social | No |
| May F 2019 (396) | To determine the overall CRC screening rate, rates by patient sociodemographic and clinical factors, and predictors of screening adjusting for patient and system factors. Also to determine whether disparities in screening exist in VA. | Retrospective cohort study | Journal article | USA | Patient-level data from the VA External Peer Review Program (EPRP), an audit program administered by the VHA Office of Reporting, Analytics, Performance, Improvement & Deployment (RAPID). EPRP data are collected annually by external contractors who manually abstract VA data from a sample of electronic medical records at each health care facility | Nationwide Population-based (patient-level data from the Veterans Health Administration External Peer Review Program (EPRP) that collected annually by external contractors who manually abstract Veterans Health Administration (VA) data from a sample of electronic medical records at each health care facility) | 2014 fiscal year (October 2013 to September 2014) | Demographics, Economic, Patient access, Patient behavior | No |
| May_2014 (397) | To examine rates and predictors of CRC screening uptake as well as time to screening in a population of African Americans and non-African Americans in a healthcare system that minimizes variations in insurance and access. | Retrospective cohort study | Journal article | USA | VA electronic medical records, the Computerized Patient Record System (CPRS) | State wide study (southern California) | Jan 1996 to Oct 2012 | Demographics, Economic, Patient access, Patient behavior, PCP access | No |
| Mayhand 2021 (398) | To expand and comprehensively explore which neighborhood and individual-level factors influence colorectal cancer screening adherence in a multiethnic cohort from the Philadelphia area. Significant associations found in this study will be beneficial in identifying characteristics of low adherence populations to target for colorectal cancer screening and educational interventions. | Cross-sectional study | Journal article | USA | The Population Health Assessment in Cancer Center Catchment Areas (PHA) study | National survey | 2017-2018 | Demographics, Economic, Patient access, Patient behavior | No |
| McEvoy 2021 (399) | To determine the proportion of individuals at average risk who utilized a recommended initial screening test in a universal healthcare coverage system. | Retrospective cohort study | Journal article | USA | MHS Data Repository (MDR) | Nationwide study | NR | Demographics, Economic, Patient access | No |
| McKinney 2014 (400) | To examine if gender differences exist among African Americans who are not adherent to CRC recommendations in terms of CRC knowledge, cancer worry, perceived risk, and intention to screen. | Cross-sectional study | Journal article | USA | NR | Multicenter | NR | Demographics, Economic, Patient access, Social | No |
| Menéndez 2020 (401) | To analyze the effect of socio-familial support in the participation of CRC population screening program. | Case-control study | Conference abstract | Spain | Clinical records | Multicenter | Mar and Sep 2019 | Demographics | No |
| Menéndez 2022 (402) | To identify the benefits and barriers perceived by the population when participating in a CRC screening program with FOBT | Case-control study | Journal article | Spain | NR | Multicenter | Mar to Sep 2019 | Demographics, Economic, Patient access, Patient behavior, Social | No |
| Mitsutake 2012 (403) | The present study examined associations between eHealth literacy, knowledge of CRC, and CRC screening practices. | Cross-sectional study | Journal article | Japan | NR | Nationwide study | 2009 | Demographics, Economic, Social | No |
| Molina-Barceló 2014 (404) | To gain insight into the social inequalities with regard to participation in a colorectal cancer screening program in order to design strategies to reduce barriers by incorporating the needs of various social groups | Cross-sectional study | Journal article | Spain | NR | Community based survey | Between October, 2009, and September, 2010 | Patient access, Patient behavior | No |
| Momplaisir 2012 (405) | To evaluate 1) the proportion of patients with HIV who had any type of colorectal cancer (CRC) screening and 2) whether having a primary care physician (PCP) or seeking care in an integrated care practice is associated with higher CRC screening. | Cross-sectional study | Journal article | USA | Medical Monitoring Project (MMP) database | Community survey | From March to August 2010 | Demographics, Economic, Patient behavior, PCP access | No |
| Monet 2021 (406) | To estimate the rate of up-to-date CRC screening among cancer survivors five years after diagnosis, and to identify the personal and institutional barriers to CRC screening in these survivors. | Cross-sectional study | Journal article | France | The French national VICAN survey (Patient interviews performed two and five years after diagnosis (2012/2015) to gather information on various health issues, including health condition, preventive health behaviors, etc.; The French national medico-administrative database (SNIIRAM), which records all care consumption data (generated inside and outside healthcare centers)) | Nationwide survey | Interviews performed two and five years after diagnosis 2012/2015 | Demographics, Economic, Patient access, Patient behavior | No |
| Mosli 2017 (407) | To evaluate the knowledge, attitudes, and practices of PHPs regarding CRC screening and to identify the factors associated with nonadherence of PHPs to screening recommendations | Cross-sectional study | Journal article | Kingdom of Saudi Arabia (KSA) | NR | Multicenter | Oct to Dec 2016 | PCP access | No |
| Myong 2012a (408) | To assess the impact of household income and economic recession on participation in CRC screening, we estimated annual participating proportions from 2007 to 2009 for different CRC screening modalities according to household income levels | Cross-sectional study | Journal article | Korea | Health Interview Survey dataset of the fourth Korean National Health and Nutrition Examination Survey (KNHANE IV) in 2007-2009. | Nationwide study | 2007 to 2009 | Economic | No |
| Myong 2012b (409) | To identify associations between relevant risk factors and the uptake of screening in Korea. | Cross-sectional study | Journal article | Korea | Fourth Korean National Health and Nutrition Examination Survey (KNHANES IV) | National survey | 2007-2009 | Demographics, Economic, Patient access, Patient behavior, Social | No |
| Nagelhout 2017 (410) | To identify differences in the endorsement of barriers to CRC screening and to evaluate the association between provider recommendation and CRC screening adherence among Hispanic, Pacific Islander and White patients | Cross-sectional study | Journal article | USA | NR | Single center | 2-month period in 2015. | Demographics, PCP access | No |
| Nápoles 2014 (411) | The purpose of this paper is to examine whether specific clinician counseling behaviors pertaining to CRC screening are associated with receipt of CRC screening among underserved Latino men and women age 50 and older. Specifically, we sought to examine whether explanations of CRC risk and screening tests, elicitation of patients’ barriers to screening, responsiveness to patients’ CRC screening concerns, and encouragement of CRC screening by primary care physicians were associated with adequate CRC screening. Given time constrained primary care visits, identification of specific physician CRC counseling components that are more strongly associated with screening might facilitate more focused discussions with a higher likelihood of CRC screening compliance | Cross-sectional study | Article in Press | USA | NR | Community based survey | Between October 2008 and May 2009 | Patient access, Patient behavior, PCP access | No |
| Narayan 2021a (412) | To estimate the proportion of patients who have undergone CT examinations who have not received recommended CRC screening. | Cross-sectional study | Journal article | USA | National Health Interview Survey 2015 | Nationwide study | 2015 | Demographics, Economic, Patient access, Social | No |
| Nieves-Jimenez 2022 (413) | N/A | Cross-sectional study | Conference abstract | NR | 2019 National Health Interview Survey | Nationwide study | 2019 | Demographics, Patient access | No |
| Ojinnaka 2015 (414) | To examine the associations between limited health literacy, patient communication habits, and ever having had a CRC screening or being adherent to screening guidelines, among low-income uninsured patients in a primary care setting | Cohort study | Journal article | USA | NR | City-wide survey | Between 2011 and 2014 | Demographics, Economic, PCP access, Social | No |
| Ooi 2019 (415) | To improve screening rates for CRC by assessing the current level of knowledge and practice of CRC screening among PCPs in Malaysia. | Cross-sectional study | Journal article | Malaysia | NR | Multicenter | July to Aug 2014 | Patient's access | No |
| Otiniano 2013 (416) | To examine the association between sociodemographic, knowledge, attitude and behavior factors with colon cancer screening among low-income Hispanic patients from an urban family medicine clinic in San Antonio, Texas | Cross-sectional study | Journal article | USA | NR | Community based survey | NR | Demographics, Economic, Patient access, Patient behavior, PCP access, Social | No |
| Pancar 2021 (417) | To determine the frequency of participation in colorectal cancer (CRC) screenings, the factors affecting participation and the association between CRC screening behaviors and health literacy | Cross-sectional study | Journal article | Turkey | NR | Community- wide study | August 2019 and November 2019 | Demographics, Economic, Patient access, Patient behavior, Social | No |
| Parsons 2012 (418) | To obtain national-level baseline data regarding Canadians’ attitudes towards and awareness of CRC screening. | Cross-sectional study | Journal article | Canada | NR | Nationwide study | 10 March 2009 to 17 April 2009 | Demographics, Patient access, Patient behavior, PCP access | No |
| Patel 2012 (419) | The present study examines the sociodemographic factors that influence decisions to getting screened for colorectal cancer in low-income African Americans. In addition, this study examines the differences in obstacles to screening by geographic region. T | Cross-sectional study | Journal article | USA | Meharry Medical College Community Health Centers Community Networks Program (Meharry CNP) | Community wide study | 2005 | Demographics, Economic, Patient access, PCP access, Social | No |
| Pausawasdi 2022 (420) | To assess Thai physicians’ recommendations for CRC screening, and the awareness of and adherence to international guidelines. | Cross-sectional study | Journal article | Thailand | NR | Nationwide study | NR | Demographics, Patient access, Patient behavior | No |
| Poroes 2020 (421) | To compare their self-reported preventive practices with the objectives of the program, namely to inform patients about CRC screening and present the choice of colonoscopy and FIT, and to identify factors associated with presenting a choice of tests. | Cross-sectional study | Journal article | Switzerland | NR | City wide study | 08 June 2018 to 29 June 2018 (Participants were FPs from the canton of Vaud who had included at least one patient in the CRC screening program since 2015. In 2016 there were 512 FPs registered to practice in the canton of Vaud) | PCP access | No |
| Puthashanan 2021 (422) | To explore the extent, associated factors and reasons of refusal of iFOBT in Kedah state, | Cross-sectional study | Conference abstract | Malaysia | NR | Multicenter | First quarter of 2019 | Demographics, Patient behavior, PCP access | No |
| Qumseya 2014 (423) | To understand attitudes and barriers that contribute to the low rate of CRC screening among Palestinians in the West Bank. | Cross-sectional study | Journal article | Palestine | NR | Nationwide study | NR | Demographics, Economic, Patient access, Patient behavior, Social | No |
| Ramai 2019 (424) | To investigate the individual characteristics associated with CRC screening using the FIT in an urban population Brooklyn, New York. In addition, to use cluster analysis within ArcGIS to determine the impact of geographic factors on FIT participation. | Retrospective study | Journal article | USA | Electronic medical records of 1500 Cancer Services Participants (CSP) at The Brooklyn Hospital Center that resided in Kings County(Brooklyn, NY) | Single center | Jan 2014- Dec 2016 | Demographics, Patient access, PCP access, Social | No |
| Ramazani 2021 (425) | To investigate the related factors in fecal occult blood test for screening of colorectal cancer based on health belief model constructs in high-risk population in east of Iran. | Cross-sectional study | Journal article | Iran | NR | City wide study | NR | Demographics | Yes |
| Rastogi 2019 (426) | To further characterize the relationship between sociodemographic factors and up‐to‐date colonoscopy use in a diverse urban center using the 2014 New York City Community Health Survey (NYCCHS) | Cross sectional study | Journal article | USA | New York City Community Health Survey (NYCCHS) | City-wide survey | 2014 | Demographics, Economic, Social | No |
| Swaminathan 2020 (427) | To identify the impact of geographic region on the preference of CRC screening modality among Louisiana adults. | Retrospective cohort study | Journal article | USA | 2016 Behavioral Risk Factor Surveillance System database | State wide study | 2016 | Demographics, Economic, Patient access, PCP access, Social | No |
| Ricardo-Rodrigues 2015b (428) | To estimate adherence to the recommendation for FOBT as screening for CRC in Spain, using the national health surveys conducted in 2009 and 2011. We also intended to identify predictive factors for adherence, on the basis of sociodemographic variables, in relation to health and lifestyle | Cross sectional study | Journal article | Spain | European Health Survey 2009 and the Spanish National Health Survey 2011 | National survey | 2009 and 2011 | Demographics, Social | No |
| Rogers 2020 (429) | To investigate CRC screening rates of Lao Americans in Minnesota, and how predisposing characteristics, enabling resources, and perceived need are associated with screening | Cross-sectional study | Journal article | USA | NR | Community wide study | NR | Demographics, Patient access | No |
| Rogers 2021 (430) | This study hypothesized that greater masculinity barriers to medical care (MBMC) would be negatively associated with CRC screening uptake. | Cohort study | Journal article | USA | NR | Community based survey | March and December 2020 | Demographics, Patient access, Patient behavior, Social | No |
| Rogers 2022 (431) | To determine whether masculinity barriers to medical care influenced ever completing CRC early-detection screening among Black and AIAN men aged 45 to 75 years compared with their White counterparts. | Cross-sectional study | Journal article | USA | NR | Nationwide study | Dec 2020 to Jan 2021 | Demographics, Patient behavior | No |
| Samuel 2021 (432) | To determine the most significant facilitators and barriers to CRC screening in an outpatient clinic in rural North Carolina. The results of this study can then be used for quality improvement to increase the rate of patients ages 50 to 75 who are up to date on CRC screening. | Cross-sectional study | Journal article | USA | The East Carolina University Internal Medicine clinic | Single center | July 1, 2018 and June 30, 2019 | Demographics, Economic, Patient access, Patient behavior, PCP access, Social | No |
| Schonberg 2015 (433) | To examine receipt of colorectal cancer (CRC) screening according to age and life expectancy (LE) in U.S. adults aged 65 and older | Cross sectional study | Journal article | United states | National Health Interview Survey | National survey | 2008-2010 | Demographics | No |
| Seibert 2017 (434) | To characterize the prevalence of guideline-adherent CRC screening among obese adults using nationally representative data, assess trends in screening strategies, and identify obesity-specific screening barriers. | Cross-sectional study | Journal article | USA | National Health Interview Survey (NHIS) 2010 | Nationwide study | 2015 | Demographics, Patient access, Patient behavior, PCP access, Social | No |
| Sekhon 2021 (435) | To evaluate the relationship between ethnicity and uptake of CRC screening in West London | Retrospective cohort study | Journal article | UK | (CWHHE) Electronic health record systemOne | Region wide study | 2012 to 2017 | Demographics, Economic, Patient access, Social | No |
| Sentell 2013 (436) | To examine the relationships among LHL, LEP, and CRC screening for Asian Americans and Whites. | Cross-sectional study | Journal article | USA | California Health Interview Survey | State wide study | 2007 | Demographics, Economic, Patient access, Social | No |
| Shahidi 2013 (437) | To: (1) compare colorectal cancer screening (CRCS) among US born citizens (USBs), naturalized citizens (NACs), and noncitizens (NOCs) and (2) evaluate clinical factors and potential barriers associated with CRCS in these populations | Cross sectional study | Journal article | USA | 2007 California Health Interview Survey | State wide survey | 2007 | Demographics, Economic, Patient access, Patient behavior, PCP access, Social | No |
| Shariff-Marco 2013 (438) | This study examines geographic variation in CRC screening and the extent to which multilevel SDOH explain its use in California, the most populous and racially/ethnically diverse state in the U.S | Cross sectional study | Journal article | USA | California Health Interview Survey | Province based survey | 2005 | Demographics, Economic, Patient access, Patient behavior, Social | No |
| Shin 2017 (439) | The aim of this study was to investigate perceptions of CRC screening modalities and recommendation behaviors among physicians in Korea. | Cross-sectional study | Journal article | Korea | NR | Multicenter | Nov 2013 to Feb 2014 | Demographics, Patient access, Patient behavior, PCP access, Social | No |
| Shin 2020b (440) | To investigate (1) whether CRC screening practices differ by the presence of disability and according to various types and severity of disability, (2) the trend of CRC screening participation rate in relation to disabilities over time; and (3) the factors associated with adherence to CRC screening. | Retrospective cohort study | Journal article | South Korea | National Health Information Database (NHID) | Nationwide study | 2006 to 2015 | Demographics, Economic | No |
| Siantz 2017 (441) | The present study analyzed population-based data from the 2007 wave of the California Health Interview Survey (CHIS) [16] to evaluate whether having a mental illness was associated with a decreased likelihood of obtaining CRC screening. | Cross-sectional observational study | Journal article | USA | California Health Interview Survey | State wide study | 2007 | Demographics, Patient access, Patient behavior, PCP access, Social | No |
| Simkin 2019 (442) | To estimate up-to-date colorectal cancer (UTD-CRC) screening across income strata by levels of urbanization. | Cross-sectional study | Journal article | Canada | Canadian Community Health Survey (2013/2014) | Nationwide study | 2013/2014 | Demographics, Economic, Social | No |
| Sing 2013 (443) | To analyze relationships between socio-demographic characteristics, healthcare access, and behavior with regard to participation in organized colorectal cancer (CRC) screening | Cross-sectional study | Journal article | France | 2010-ESPS survey, conducted by the Institute for Research and Information in Health Economics, is drawn from a permanent representative sample of the population protected by the French Health Insurance | National survey | 2010 | Demographics, Economic, Patient access, Patient behavior, PCP access, Social | No |
| Singal 2013 (444) | To examine whether having a primary care physician (PCP) is associated with reduced ethnic disparities for colorectal cancer (CRC) screening and whether clustering of minorities within PCPs contributes to the disparities | Retrospective cohort study | Journal article | USA | Texas Medicare beneficiaries (Medicare beneficiary summary files, Medicare Provider Analysis and Review (MedPAR) files, Outpatient Standard Analytical Files (OutSAF), and Medicare Carrier files. Provider information was obtained from the American Medical Association (AMA) physician Masterfile) | State wide study | 2000-2009 | Demographics, Patient access, PCP access | No |
| Skau 2022 (445) | To investigate the association between socioeconomic status (SES) and the risk of having an incomplete colonoscopy (IC) in the Danish Colorectal Cancer (CRC) Screening Program. | Register-based cross-sectional study | Journal article | Denmark | Danish Colorectal Cancer Screening Database (DCCSD) and Statistics Denmark | Nationwide study | Mar 2014 to Feb 2017 | Demographics, Economic, Social | No |
| So 2012 (446) | To assess the uptake rate of colorectal cancer screening among average-risk Chinese people aged 50 or above, and to identify factors associated with the likelihood that this population will undergo colorectal cancer screening tests | Cross-sectional study | Journal article | China | NR | Region wide survey | 2 to 28 May 2007 | Demographics, Economic, Patient behavior, Social | No |
| Solís-Ibinagagoitia 2020 (447) | The aim of the present study is to identify socio-demographic and lifestyle factors related to non-participation in the CRC Screening Program of the Basque Country (Spain). | Cross-sectional descriptive study | Journal article | Basque Country | Basque Country BCSP Database | Community wide study | 2015 to 2017 | Demographics, Economic, Patient behavior, PCP access | No |
| Stanley 2019 (448) | To look at the differences in colorectal cancer screening awareness between two rural communities in Texas. | Cross-sectional study | Journal article | USA | BehaviorRisk Factor Surveillance System (BRFSS) was given to patients at primary care clinics in Clifton and Haskell | Multicenter | July 2014 and February 2016 | Demographics, Patient access | No |
| Stevens 2019 (449) | This study aimed to investigate whether first participation in the English NHS FOBT cancer screening program is associated with spontaneous lifestyle changes among attenders, compared with non-attenders, in a screening-naive population of men from an English prospective cohort study. | Cohort study | Journal article | UK | English Longitudinal Study of Ageing (ELSA)The cohort was originally sampled from the Health Survey for England | National survey | 2014-2015 | Demographics, Economic, Social | No |
| Suh 2015 (450) | To investigate the association between different indicators of SES and CRC screening rates in Korea | Cross-sectional study | Journal article | Korea | Korean National Cancer Screening Survey (KNCSS) | Nationwide study | 2010 to 2012 | Demographics, Economic, Patient access, Social | No |
| Swaminathan 2019 (451) | To identify the impact of social determinants of health on the choice of CRC screening among Louisiana adults. | Retrospective cohort study | Conference abstract | USA | Behavioral Risk Factor Surveillance System(BRFSS) database from 2016 | State wide study | NR | Demographics, Economic, Patient access, Patient behavior | No |
| Swaminathan 2020 (427) | To identify the impact of geographic region on the preference of CRC screening modality among Louisiana adults. | Retrospective cohort study | Conference abstract | USA | 2016 Behavioral Risk Factor Surveillance System database | State wide study | 2016 | Demographics | No |
| Sy 2018 (452) | The purpose of this study was to compare CRCS predictors among AAs who comprise the largest subgroups in the United States. AA adults age 50-75 years were examined using the Medical Expenditure Panel Survey (MEPS). We hypothesized that CRCS prevalence among AA ethnicities is heterogeneous and that the reasons related to CRCS among AA ethnic subgroups are associated with social demographics, acculturation, health care access and satisfaction, and health attitudes. | Cross-sectional study | Journal article | USA | Medical Expenditure Panel Survey | National and community based survey | 2009-2014 | Demographics, Economic, Patient access, Patient behavior, Social | No |
| Tabaac 2018 (453) | To contribute to the nascent cancer prevention literature among trans and gender-nonconforming individuals by ascertaining rates of breast, cervical, prostate, and colorectal cancer screening behaviors by gender identity | Cross-sectional study | Journal article | USA | Publicly available population-level data from the 2014, 2015, and 2016 BRFSS | Nationwide study | 2018 | Demographics | No |
| Taheri-Kharameh 2015 (454) | To determine the factors associated with colorectal cancer screening adherence among Iranians 50 years and older using the Health Belief Model. | Cross-sectional study | Journal article | Iran | NR | Multicenter | June 2012 to May 2013 | Demographics, Patient access, Patient behavior, Social | No |
| Taş 2019 (455) | This study was conducted to determine the effect of the knowledge and health beliefs of individuals about colorectal cancer on their screening behavior. | Cohort study | Journal article | Turkey | NR | Nationwide study | 24 April and 31 July 2018 | Demographics, Economic, Patient access, Patient behavior, Social | No |
| Tastan 2013 (456) | To evaluate of the knowledge, behavior and health beliefs of individuals over 50 regarding colorectal cancer screening, with a descriptive and cross-sectional design at Karabuk Life and Health Center in Turkey. | Cross-sectional study | Journal article | Turkey | Karabuk life and health center in turkey | Single center | Jun 2012- Aug 2012 | Demographics, Patient access, Patient behavior, PCP access | Yes |
| Thompson 2013 (457) | To determine whether the items could be integrated into a comprehensive measure comprising several sub-scales representing the different attitudinal dimensions. Item difficulties and ordering of response thresholds were analyzed for the different subscales and scores from the subscales were used to examine the predictive validity of the measure. | Cross-sectional study | Journal article | USA | NR | Community based survey | NR | Demographics, Economic, Patient access, Patient behavior, Social | No |
| Thompson 2014 (458) | To identify a streamlined set of issues important for colorectal cancer communication and interventions with older African Americans. | Cross-sectional study | Journal article | USA | NR | Nationwide study | 2009 to 2010 | Demographics, Patient access, Patient behavior, PCP access | No |
| Todorov 2018 (459) | To examine trends in knowledge, recent use and reasons for use or non-use of fecal occult blood testing (FOBT) for colorectal cancer (CRC) screening from 2011 to 2014. Screening awareness and demographic factors related to non-use were also examined. | Cross-sectional study | Journal article | Australia | South Australian Health Omnibus Survey (HOS) | Region wide study | 2011,2012 and 2014 | Demographics, Economic, Patient access | No |
| Torosian 2021 (460) | To evaluate knowledge of and attitudes toward CRC and screening programs in Armenia. | Cross-sectional study | Journal article | Armenia | NR | Multicenter | NR | Demographics, Economic, Social | No |
| Vanaclocha-Espi 2017 (461) | To analyze the sociodemographic and organizational factors influencing participation in population-based colorectal cancer screening programs (CRCSP) in Spain | Cohort based study | Journal article | Spain | NR | Province based survey | 2000-2012 | Demographics, Economic, Patient access | No |
| Varlow 2014 (462) | To describe self-reported bowel cancer screening participation, beliefs and attitudes in a sample of New South Wales (NSW) adults, and to identify beliefs and demographic factors associated with self-reported bowel cancer screening participation. | Cross-sectional study | Journal article | Australia | International Cancer Benchmarking Partnership Module 2 | State wide study | May to Sep 2011 | Demographics, Patient behavior | No |
| Viramontes 2019 (463) | To determine the current screening rate, screening modalities, and predictors of screening among U.S. Hispanics as well as regional variation in Hispanic-White screening disparities. | Cross-sectional study | Conference abstract | USA | 2016 Behavioral Risk Factor Surveillance System (BRFSS) survey | Nationwide study | 2016 | Demographics, Economic, Patient behavior, Social | No |
| Viramontes 2020 (464) | The objective of the study is, 1) to compare CRC screening rates in Hispanics and whites in the U.S. 2) to compare screening modalities used by Hispanics and whites, 3) to determine predictors of screening among U.S. Hispanics and 4) to quantify regional (state-level) variation in CRC screening rates among Hispanics in the U.S. | Cross-sectional study | Journal article | USA | behavioral Risk Factor Surveillance System (BRFSS) survey | National and community-based survey | 2016 | Demographics, Economic, Patient access, PCP access, Social | No |
| Von Wagner 2019 (465) | This prospective study aimed to identify predictors of intention and subsequent attendance of flexible sigmoidoscopy screening using constructs derived from the Health Belief Model (HBM). | Prospective observational study | Journal article | UK | English General Practices | Multicenter | May 2015 - April 2016 | Demographics, Economic, Patient access, Patient behavior | No |
| Von Wagner 2020 (466) | Type 2 diabetes has been identified as a risk factor for colorectal cancer, but little is known about whether it influences participation in colorectal cancer screening programs. This study tested the extent to which Type 2 diabetes is negatively associated with colorectal cancer screening uptake | Cross-sectional study | Journal article | UK | ELSA’s wave 6 questionnaire and nurse visit, which were collected in 2012 and 2013. | Nationwide study | 2012 to 2013 | Demographics, Economic, Social | No |
| Wallace 2012 (467) | The purpose of this study is to examine whether there were regional differences in CRC screening among US adult AAs and Whites. Our findings will advance the literature and inform targeted CRC screening interventions individually and regionally. We tested the following two hypotheses: Hypothesis 1. The odds of residents in the south, having had a colorectal screening, are lower than those in other regions. Hypothesis 2. The odds of AAs, having had a colorectal screening, are lower than Whites having had a colorectal screening. | Observational (cohort) study | Journal article | USA | NHIS | National and community-based survey | 2010 | Demographics, Patient access, Social | No |
| Walsh 2013 (468) | To evaluate factors associated with willingness to undergo CRC screening for personal and public health benefit among women from diverse race/ethnic groups | Cross-sectional study | Journal article | USA | NR | Multicenter | Oct 2003- Dec 2005 | Demographics, Economic, Patient access, Patient behavior, Social | No |
| Wang 2017 (469) | To investigate the potential factors across urban-rural groups on the usage of CRC screening | Cross-sectional study | Journal article | USA | California Health Interview Survey | Statewide study | 2019 | Demographics, Economic, Patient behavior, Social | No |
| Wangmar 2018 (470) | The aim of the present study was to investigate anxiety levels related to the decision to participate or not in a CRC screening program among both screening participants and non-participants. Further, we aimed to explore associations between higher levels of anxiety related to the decision and individuals’ sociodemographic and personal characteristics. | Cross-sectional study | Journal article | Sweden | Screening of Swedish Colons (SCREESCO) | Nationwide study | Oct 2015 to Jun 2016 | Demographics, Patient access, Social | No |
| Weiss 2013 (471) | We evaluated patient, provider, and clinic factors that predict variation in CRC screening among primary care clinics and primary care providers (PCPs). | Retrospective study | Journal article | USA | Electronic medical record (EMR) data and survey | Multicenter | 2009 | Patient access, PCP access | No |
| Wilcox 2015 (472) | This study examined disparities in blood stool test (BST) compliance and colonoscopy use by race/ ethnicity (Haitian, NHW, non-Hispanic Black [NHB], and Hispanic) among randomly selected households in Little Haiti, Miami-Dade County, Florida | Cross-sectional study | Journal article | USA | NR | Community wide study | Nov 2011-Dec 2012 | Demographics, Economic, Patient access, Patient behavior, Social | No |
| Wong 2013a (473) | This study was conducted to determine the prevalence of uptake of colorectal cancer screening and knowledge about CRC among adults aged 50 years or more in the general population in Singapore. In addition, we applied the HBM to compare gender differences in the factors associated with CRC screening | Cross-sectional study | Journal article | Singapore | A nationwide representative household survey | National survey | 2007 to 2008 | Demographics, Patient access, Patient behavior, PCP access, Social | No |
| Wong 2013c (474) | To evaluate the proportion of self-referred screening participants having various psychological barriers and the factors associated with these barriers. | Cross-sectional study | Journal article | Hong Kong | NR | Territory-wide | May 2008- Sep 2012 | Demographics, Economic, Patient behavior, Social | No |
| Yager 2014 (475) | To (1) assess for sex differences in colorectal cancer screening (CRCS) within a large, contemporary population-based sample in California; and (2) examine the impact of income, education, and insurance status on sex differences in CRCS | Cross-sectional study | Journal article | USA | California Health Interview Survey (CHIS) | Statewide population based | 2007 | Demographics, Economic, Patient access, Patient behavior, PCP access, Social | No |
| Zajac 2017 (476) | The primary purpose of this study was to explore anticipated regret (AR) and its relationship to future bowel cancer screening intentions in an Australian sample. Our aim was to establish whether AR might be a useful target for behavioral interventions designed to improve bowel screening compliance in an Australian population. A secondary aim was to examine whether AR differed across individuals to determine whether it should be targeted in specific groups as opposed to all individuals. | Cross-sectional study | Journal article | Australia | NR | Province based survey | NR | Demographics, Economic, Patient access, Patient behavior, Social | No |
| Zamorano-Leon 2020 (477) | To assess the temporal trend of colorectal cancer (CRC) screening uptake according to the year of screening implementation in each region and to identify predictors for the uptake of CRC screening. | Cross-sectional study | Journal article | Spain | Spanish National Health Surveys 2011 and 2017 | National survey | July 2011 to July 2012 for the SNHS 2011 and from October 2016 to October 2017 for the 2017 SNHS | Demographics, Patient behavior, Social | No |
| Zhu 2021 (478) | To examine barriers to utilization of three commonly used screening options (FIT/gFOBT, mt-sDNA, and screening colonoscopy) and assessed differences by socio-demographic characteristics, healthcare access, and health status. | Cross-sectional study | Journal article | USA | NR | Nationwide study | 43770 | Demographics, Economic | No |
| Zhu 2022 (479) | To 1) characterize clinicians’ routine CRC screening recommendations of guideline-endorsed screening methods among average-risk patients, 2) examine how myriad factors (e.g., scientific evidence, clinical practice guidelines) may be associated with these recommendations, and 3) identify barriers to recommending each CRC screening method among clinicians who do not routinely recommend these options to average-risk patients. | Cross sectional study | Journal article | USA | Data were collected via a web survey developed by the authors and implemented in November and December 2019 by the National Opinion Research Center at the University of Chicago (http://www.norc.org) using a third-party vendor, Dynata, | National survey | November and December 2019 | PCP access | No |
| **Gastric** **cancer** | | | | | | | | | |
| Chang 2015 (480) | To assess individual and area-level determinants of gastric cancer screening participation | Cross-sectional study | Journal article | Korea | 2007 to 2009 KNHANES IV | Nationwide study | 2007 to 2009 | Demographics, Economic, Patient access, Patient behavior, PCP access, Social | No |
| Kim 2020 (481) | To examine (1) trends in the gastric cancer screening rate among people with disabilities over time, and (2) whether gastric cancer screening participation and modalities difered according to presence, severity, and type of disability. | Retrospective cohort study | Journal article | South Korea | National Health Information Database (NHID) | Nationwide study | 2006 to 2015 | Demographics, Economic | No |
| Lee 2015 (482) | This study was conducted to assess trends in the use of either upper gastrointestinal series (UGIS) or endoscopy to screen for gastric cancer, as well as to assess factors strongly associated with changes therein, over a 10-year period. | Cross-sectional prospective study | Journal article | Korea | NCSP database, which contains information on Medical Aid Program (MAP) recipients and on National Health Insurance (NHI) beneficiaries invited to participate in the NCSP | Nationwide study | 2002 to 2011 | Demographics, Economic | No |
| Liu 2019 (483) | To assess the knowledge of risk factors and warning symptoms and attitude towards gastric cancer screening among the general population in China | Cross-sectional study | Journal article | China | NR | Providence based survey | Between March and July 2018 | Demographics, Economic | No |
| Park 2013 (484) | To elucidate the differential effect of obesity on the compliance with stomach cancer screening according to sex and the screening methods in a large representative sample of Korean adults | Cross-sectional study | Journal article | Korea | The Korea National Health and Nutrition Examination Survey (KNHANES) | Nationwide study | 2007-2009 | Demographics | No |
| Shim 2019 (485) | To explore whether inequities in the use of preventative services are associated with employment conditions. This study used gastric cancer screening as a surrogate for the use of preventative health services. | Cross-sectional study | Journal article | Korea | Korea National Health and Nutrition Examination Survey | Nationwide study | 2007-2008 | Demographics, Economic, Social | No |
| Shin 2012 (486) | We explored barriers to and predictors of gastric cancer screening participation among a nationally representative sample. | Cross-sectional study | Journal article | Korea | Fourth Korea National Health and Nutrition Examination Survey 2008 KNHANES IV | Nationwide study | 2007 to 2009 | Demographics, Economic, Patient access, Patient behavior, Social | No |
| Shin 2016 (487) | To investigate the association between participation in gastric cancer screening and salt preference, adding to known relevant factors including sociodemographic and cognitive factors among a nationally representative Korean population | Cross-sectional study | Journal article | Korea | Korean National Cancer Screening Survey (KNCSS) | Nationwide study | 2006-2007 | Demographics, Economic, Patient behavior, Social | No |
| Yu 2022 (488) | To assess the utility of the risk stratification and the feasibility of the endoscopy screening in high-risk individuals in northeast China and further to provide valid references for UGC screening strategy design in the future | Prospective cohort study | Journal article | China | National cancer screening programme in China (CanSPUC) | Providence based survey | Between October 2016 and August 2017 | Demographics, Patient access, Social | No |
| **Lung cancer** | | | | | | | | | |
| Abeyweera 2019 (489) | To access General Practitioners’ Knowledge and Practice of Lung Cancer Screening | Cross-sectional study | Journal article | Australia | NR | Nationwide study | NR | PCP access | No |
| Barta 2021 (490) | To characterize attitudes and beliefs toward lung cancer and lung cancer screening (LCS) and to identify factors associated with LCS adherence. | Cross-sectional study | Journal article | USA | NR | Community wide study | March 15, 2018 - December 5, 2019 | Demographics, Patient access, Patient behavior, Social | No |
| Broadbent 2022 (491) | The aim of this study was to investigate levels of willingness to undergo LCS in HL survivors, and to identify the psycho-social factors associated with screening hesitancy | Retrospective study | Journal article | UK | Postal questionnaire, ≥5 year lymphoma survivors (ADAPT) | Nationwide study | 43160 | Demographics, Economic, Patient behavior | No |
| Bui 2018a (492) | To examine willingness to be screened among Korean males using LDCT and to determine factors associated with lung cancer screening intentions (LCS) based on the Health Belief Model (HBM) | Cross-sectional study | Journal article | Korea | Korean National Cancer Screening Survey (KNCSS) | Nationwide study | 2015 | Demographics, Economic, Patient access, Patient behavior, PCP access, Social | Yes |
| Cam 2015 (493) | To assess changes in intentions to undergo lung cancer screening in response to being informed about exposure to radiation during low-dose computed tomography (LDCT) tests and to identify factors with the greatest influence thereon among Korean men. | Cross-sectional study | Journal article | Korea | Korean National Cancer Screening Survey (KNCSS) | Nationwide population based | 2013 | Demographics, Economic, Patient access, Patient behavior, Social | No |
| Cataldo 2016 (494) | The aims for this study were to (1) describe older smokers’ health risk beliefs related to cigarette smoking and lung cancer; (2) identify demographic, smoking history, health risk perceptions, knowledge, and attitude factors related to whether a smoker would agree to a LDCT scan; and (3) using binary logistic regression, provide a predictive model of factors to explain an older smoker’s willingness to have a LDCT scan. | Cross-sectional descriptive, correlational study | Journal article | USA | NR | Nationwide study | 41821 | Patient behavior | No |
| Delmerico 2014 (495) | To identify past use of CT scanning and reasons for having or not having the screening done among f adult current and former smokers. | Cross-sectional study | Journal article | USA | NR | Nationwide study | 2011 | Demographics, Patient behavior | No |
| Doria-Rose 2012 (496) | This study examine the use of chest x-ray and CT in the United States in 2010. We also estimate the size of the U. S. population that might be considered for lung cancer screening with LDCT, based on population characteristics that correspond to NLST eligibility criteria | Cross-sectional study | Journal article | USA | The 2010 National Health Interview Survey | National survey | 2010 | Demographics, Economic, Patient access, Patient behavior | No |
| Ferguson 2020 (497) | To determine if geographic distance to a Veterans Administration LCS referral center affected completion rates of LDCT screening in Veterans | Observational prospective cohort study | Journal article | UK | NR | Single center | Dec 2013 to Sep 2019 | Patient access | No |
| Gudina 2021 (498) | To identify factors associated with the uptake of lung cancer screening in high-risk individuals in the U.S population | Cross-sectional study | Journal article | USA | Behavioral Risk Factor Surveillance System (BRFSS) | Nationwide study | 2017 to 2019 | Demographics, Economic, Patient access, Patient behavior, PCP access, Social | No |
| Guo 2020 (499) | This study aimed to assess the participation rate and detection rate of lung cancer in a population-based screening program and the factors associated with participation. | Cross-sectional study | Journal article | China | Cancer Screening Program in Urban China | Multicenter | October 2013 to October 2019 , with follow-up until March 10, 2020 | Demographics, Patient behavior, Social | No |
| Kim 2021 (500) | To identify factors associated with health check-up and cancer screening participation among family caregivers of patients with dementia | Cross-sectional study | Journal article | Korea | Korea Community Health Survey | National survey | 2017 | Demographics, Economic, Social | No |
| Lake 2020 (501) | To extract demographic and clinical characteristics, smoking history, and lung cancer screening outcomes | Retrospective cohort study | Journal article | USA | Clinical databases of the Jane and Leonard Korman Respiratory Institute Lung Cancer Screening Program | Single center | May 2015- July 2017 | Demographics, Patient behavior, Social | No |
| Lewis 2019 (502) | To test the hypothesis that low provider knowledge of LCS guideline recommendations would be associated with less provider-reported screening with LDCT. | Cross-sectional study | Journal article | USA | NR | Multicenter | Feb to May 2017 | PCP access | No |
| Lowenstein 2022 (503) | This study aims to describe barriers to lung cancer screening (LCS) among family medicine and general internal medicine primary care physicians (PCPs) and assess the association of barriers with discussion and referral for screening | Cross-sectional study | Journal article | USA | Random sample of primary care physicians (PCPs) in California | Providence based survey | May-October 2017 | Economic, PCP access | No |
| Mukthinuthalapati 2020 (504) | To assess knowledge, attitudes, and practices pertaining to lung cancer screening among primary care physicians in a public urban health network | Cross-sectional prospective survey | Journal article | USA | NR | Region wide study | Feb 2019 to May 2019 | PCP access | No |
| Narayan 2021b (412) | The objective of the current study was to estimate recent LCS use using cross-sectional survey data from the 2018 Behavioral Risk Factor Surveillance System (BRFSS) survey. | Cross-sectional study | Journal article | USA | Behavioral Risk Factor Surveillance System; Telephone survey | Nationwide study | 2018 | Demographics, Economic, Patient access, Patient behavior, PCP access, Social | No |
| Neslund-Dudas 2021 (505) | To determine whether individual or neighborhood level factors were associated with completion of a baseline screening after an order for LCS low dose CT (LDCT) was placed. | Retrospective cohort study | Conference abstract | USA | Lung Population-based Research to Optimize the Screening Process (PROSPR) Consortium | Multicenter (five health systems) study | Jan 2014 to Jun 2019; Follow-up through Sep 2019 | Demographics, Patient behavior | No |
| Nunez 2021 (506) | To characterize first, how often Veterans decline LCS after a SDM discussion, and second, what patient clinical and demographic factors are associated with declining LCS. | Retrospective cohort study | Journal article | NR | Administrative data from the VA’s Corporate Data Warehouse | Nationwide study | 2013 to 2021 | Demographics, Economic, Patient access, PCP access | No |
| Núñez 2021 (507) | To analyze adherence to follow-up based on standardized follow-up recommendations in a national cohort and to identify factors associated with delayed or absent follow-up. | Retrospective cohort study | Journal article | USA | VHA’s Corporate Data Warehouse (CDW) | Multicenter | Jan 1, 2015, to Nove 30, 2019 | Demographics, Economic, Patient access, Patient behavior, PCP access | Yes |
| Percac-Lima 2019 (508) | To assess perceptions about lung cancer and awareness of, interest in, and barriers to lung screening among older current and former smokers | Cross-sectional study | Journal article | USA | NR | Multicenter | Jul 2015 to Jun 2016 | Demographics, Patient access | No |
| Quaife 2018 (509) | The present study examined interest in a national lung cancer screening program and modifiable attitudinal factors that may affect participation by smokers. | Cross-sectional study | Journal article | UK | Attitudes, behavior and Cancer UK Survey (ABACUS) | National survey | 42095 | Patient behavior | No |
| Quaife 2021a (510) | To evaluate psychological correlates of LDCT lung cancer screening uptake behavior across three annual screening rounds. | Prospective longitudinal cohort study | Journal article | UK | SUMMIT- Study a multicenter screening implementation trial | Multicenter | April 2019 to September 2019 | Patient access, Patient behavior | No |
| Raju 2020 (511) | The first aim of this study was to identify differences in demographic, clinical, and socioeconomic characteristics between patients who qualified for but did not participate and patients who did participate in our LCS program. The second aim was to identify potential barriers to participation in our LCS program. | Retrospective case-control study and Survey | Journal article | USA | NR | Single center | Apr 2015 to Aug 2016 | Demographics, Patient access, Patient behavior, Social | Yes |
| Rajupet 2017 (512) | To assess the knowledge and attitudes of PCPs versus specialists (oncologists, pulmonologists, radiologists) towards lung cancer screening with LDCT as well as their likelihood to recommend LDCT screening. | Cross-sectional study | Journal article | USA | NR | Single center | Oct to and 2014 | PCP access | No |
| Raz 2018 (513) | To determine whether utilization of LDCT and perceived barriers to LDCT varied based on understanding the USPSTF guidelines for LCS. | Cross-sectional study | Journal article | USA | NR | City wide study | Jan to Oct 2015 | PCP access | No |
| Rennert 2020 (514) | To identify factors, including characteristics of patients and referring clinicians, that influence LDCT screening completion following participation in SDM. | Retrospective cohort study | Journal article | USA | Prisma electronic health record (EHR) (Grren ville health system) | Single center | 2016 to 2017 | Demographics, Patient access, Patient behavior, PCP access | No |
| Rustagi 2022 (515) | To determine whether health status is associated with LCS and whether racial or ethnic disparities are associated with LCS independently of health status. | Cross-sectional study | Journal article | USA | Behavioral Risk Factor Surveillance System annual surveys | Nationwide study | Aug 2021 to Nov 2021 | Demographics | No |
| See 2020 (516) | The aims of the study were to gauge the preference for lung cancer screening among Australian ever-smokers, identify any association between perceived lung cancer risk and LDCT screening eligibility on screening preference, and assess relative importance of possible screening drivers and barriers. | Cross-sectional study | Journal article | Australia | NR | Multicenter | Jan 2017 to Jul 2017 | Demographics, Patient access, Patient behavior, Social | No |
| Spalluto 2022 (517) | To test the hypothesis that among Veterans who undergo initial low dose computed tomography (LDCT) lung cancer screening, rural Veterans would be less likely to complete annual repeat low dose computed tomography (LDCT) lung cancer screening than nonrural Veterans. | Retrospective cohort study | Journal article | USA | 10 Veterans Affairs medical centers- The Veterans Affairs (VA) Corporate Data Warehouse; and the VA Informatics and Computing Infrastructure data warehouse. | Multicenter register-based study | 2015 to 2019 | Demographics, Economic, Patient access, Patient behavior, PCP access | No |
| Stowell 2020 (518) | To compare eligibility for and utilization of Lung Cancer Screening between Transgender and gender diverse (TGD) and cisgender persons in the United States. study also examined if the utilization of LCS varied by smoking status within each gender identity group. | Cross-sectional study | Journal article | USA | Behavioral Risk Factor Surveillance System (BRFSS) | Nationwide study | 2017 to 2018 | Demographics, Patient access, Patient behavior | No |
| Stowell 2021 (519) | This study aimed to identify factors associated with delayed adherence to follow-up in lung cancer screening | Retrospective study | Journal article | USA | A data warehouse and lung cancer screening registry | Single center | 1 January 2016 to 17 October 2018 | Demographics, Economic, Patient access, Patient behavior, PCP access | No |
| Tanner 2013 (520) | To assess the role of beliefs and attitudes toward LC screening among veterans | Cross-sectional study | Journal article | USA | NR | Single center | April 2012 to May 2012 | Patient behavior, PCP access | No |
| Veliz 2019 (521) | To compare eligibility for lung cancer screening and receipt of a CT scan for lung cancer among sexual minorities. | Cross-sectional study | Journal article | USA | Behavioral Risk Factor Surveillance System survey during the 2017 | Nationwide study | 2017 | Demographics | No |
| Xie 2021 (522) | To examine the social determinants of receiving lung cancer screening and explore potential solutions to eliminate the health disparities in lung cancer. | Cross-sectional study | Conference abstract | USA | Behavioral Risk Surveillance System survey in 2018 | Nationwide study, Secondary analysis | 2018 | Demographics, Economic, Patient behavior | No |
| **Prostate cancer** | | | | | | | | | |
| Abuadas 2015 (523) | To explore strong factors linked to participation in prostate cancer screening among older Jordanian adults using the Health Belief Model (HBM) | A cross-sectional quantitative survey | Journal article | Jordan | NR | Multicenter | May 17th, 2014, to August 31th 2014 | Demographics, Economic, Patient access, Patient behavior, PCP access | No |
| Arega 2020 (524) | To investigate implications of changes in screening guidelines including disparities in prostate cancer screening across various demographics factors | Cross-sectional study | Conference abstract | USA | Center for Disease Control Behavioral Risk Factor Surveillance System (BRFSS), | Nationwide study | 2013-2018 | Demographics | No |
| Bilgili 2019 (525) | To determine the personal attitudes and beliefs of Turkish men related to PCa and screening. | Cross-sectional study | Journal article | Turkey | NR | Region wide study | NR | Demographics | No |
| Bugoye 2019 (526) | This study aimed to determine knowledge and perceived risk of prostate cancer, and the utilization of prostate cancer screening services, and associated factors, among men in Dares Salaam, Tanzania in order to inform ongoing and new intervention measures. | Cross-sectional study | Journal article | Tanzania | NR | City wide population based | May and August, 2018 | Demographics, Economic, Patient access, Patient behavior, Social | No |
| Burns 2012 (527) | The objectives of this paper are to analyze the determinants of prostate cancer screening uptake in the Republic of Ireland and to compare the role of non-need factors in uptake of screening among those in and outside the age range recommended as cost-effective for screening according to the European Randomized Study of Screening for Prostate Cancer (ERSPC). | Cross-sectional survey | Journal article | Ireland | Irish health and wellbeing survey, SLAN 2007 | National survey | 2007 | Demographics, Economic, Patient access, Social | No |
| Cobran 2014 (528) | The research questions for this study were as follow: (1) What are the associations between nativity, perceptions of CaP fatalism, and CaP screening with PSA testing within the last year? (2) What are the strongest predictors of PSA testing within the last year and do these predictors vary by perceptions of CaP fatalism and nativity? | Cross-sectional study | Journal article | USA | NR | Multicenter | Jan 2012- Mar 2012 | Demographics, Economic, Patient access, Patient behavior, PCP access, Social | No |
| Ddumba 2019 (529) | The study aimed at determining the individual characteristics and health system factors that influence uptake of PC screening among men aged 50 years and above. | Cross-sectional study | Conference abstract | Uganda | NR | NR | NR | Patient access, Patient behavior | No |
| Dean 2015 (530) | The primary aim of this study was to examine the link between social capital and prostate cancer preventive screening behaviors for African American men in Philadelphia, Pennsylvania neighborhoods. position) | Cross-sectional study | Journal article | USA | Public Health Management Corporation (PHMC) random-digit dialing survey data (2004, 2006, and 2008) and 2000 U.S. Census Data | National survey | Public Health Management Corporation (PHMC) random-digit dialing survey data (2004, 2006, and 2008) and 2000 U.S. Census Data | Demographics, Economic, Patient access, Patient behavior, Social | No |
| Frego 2022 (531) | To assess the association between self-reported alcohol use and prostate cancer (PCa) screening using the U.S.-based Behavioral Risk Factor Surveillance System (BRFSS) survey. | Cross-sectional study | Journal article | USA | Centers for Disease Control and Prevention’s 2018 BRFSS | Nationwide study | 2018 | Demographics, Economic, Patient access, Patient behavior, PCP access, Social | No |
| Gift 2020 (532) | To determine the knowledge, practice and attitude towards prostate cancer screening at Kitwe Teaching Hospital (KTH). | Cross-sectional study | Journal article | Zambia | NR | Single center | Apr - Sep 2019 | Demographics, Patient access, Patient behavior, Social | No |
| Glenn 2012 (533) | To examine potential ethnic differences in prostate cancer screening behavior and correlates of screening in an ethnically diverse sample of first-degree relatives of prostate cancer cases | Cross-sectional study | Journal article | USA | California Cancer Registry (CCR) cancer patient families/ Care givers | Province based survey | Between 1997 and 2000 | Demographics, Patient access, PCP access, Social | No |
| Haider 2017 (534) | To determine the relationship between prostate-specific antigen (PSA)-related information obtained from the provider and PSA test uptake. | Cross-sectional study | Journal article | USA | Fourth edition of the Health Information National Trends Survey, a nationally-representative US survey | Nation wide study | Oct 2011 to Jan 2012 (cycle 1); Octo 2012 to Jan 2013 (cycle 2); Sep 2013 to Oct 2013 (cycle 3) | Demographics, Patient access, PCP access, Social | No |
| Kangmennaang 2016a (535) | To examine the factors that influence men’s decision to screen for prostate cancer in Namibia. | Cross-sectional study | Journal article | Namibia | 2013 Namibia Demographic and Health Survey (NDHS) | Nation wide study | 2013 | Demographics, Economic, Patient access, PCP access, Social | No |
| Kangmennaang 2016b (536) | To examine the influence of health insurance coverage, access to knowledge, and information on men decision to screen for prostate cancer in the DR. T | Cross-sectional study | Journal article | Dominican Republic | Dominican Republic Demographic and Health Survey (DRDHS, 2013) | Nation wide study | 2013 | Demographics, Economic, Patient access, Patient behavior, Social | No |
| Lee 2013a (537) | To investigate (1) the current rate of prostate cancer screening of Korean American immigrant men and (2) the role of cultural variables on prostate cancer screening adherence among this group | Cross-sectional survey | Journal article | USA | NR | Community based survey | 2009 | Demographics, Economic, Patient access,Patient behavior, Social | No |
| Ma 2020 (538) | To evaluate self-reported PSA screening and decision making among LGBT populations | Cross-sectional study | Conference abstract | USA | Behavioral Risk Factor Surveillance System database 2014-2016 and 2018 | Nationwide study | 2014-2016 and 2018 | Demographics | No |
| Mbugua 2021 (539) | The study aimed to assess the awareness of prostate cancer and screening among men aged 40–69 years in a rural community in Kenya. | Cross-sectional mixed-method survey | Journal article | Kenya | NR | Community wide study | 43556 | Demographics, Patient access,PCP access, Social | No |
| Morlando 2017 (540) | To assess knowledge, attitudes and behaviors regarding prevention of prostate cancer and determinants associated with these outcomes, in a sample of adult men in southern Italy | Cross sectional study | Journal article | Italy | NR | Province based survey | January to April 2011 | Demographics, Patient access,Patient behavior, PCP access, Social | No |
| Ogunsanya 2016 (541) | The purposes of the study were to examine the prevalence of prostate cancer screening (PCS) in the United States and to identify predictors of PCS guided by Andersen’s Behavioral Model of Health Services Use (ABM). | Cross- sectional study | Journal article | USA | Behavioral Risk Factor Surveillance System (BRFSS) database | Nationwide study (50 states); landline and cellular telephones-based interview | 2014 | Demographics, Economic, Patient access,Patient behavior, PCP access, Social | No |
| Opondo 2022 (542) | This study aimed to assess the effect of perceived self-vulnerability to prostate cancer on screening uptake among male health workers in Kisumu County, western Kenya | Cross-sectional study | Journal article | Kenya | NR | Multicenter | Nov 2018 to Jan 2019 | Demographics, Economic, Patient access,Patient behavior, Social | No |
| So 2014 (543) | To investigate the uptake rate of prostate specific antigen (PSA) testing among Hong Kong Chinese males aged 50 or above, and identify factors associated with the likelihood of undergoing a PSA test. | Cross-sectional study | Journal article | Hong Kong | NR | Region wide study | 2007 | Demographics, Economic, Patient behavior, PCP access, Social | No |
| Tasian 2012 (544) | We sought to identify patterns in physician knowledge of and attitudes towards PSA screening and to determine how these patterns along with patient and provider demographics influence PSA screening practices. | Cross-sectional study | Journal article | USA | NR | Multicenter | 2003 to 2004 | PCP access | No |
| Wong 2020 (545) | To determine the association between sexual orientation and prostate cancer screening among men 40 years of age and older | Cross-sectional study | Conference abstract | USA | Behavioral Risk Factor Surveillance System (BRFSS) 2016 | Nationwide study | 2016 | Demographics | No |
| Yeboah-Asiamah 2017 (546) | This study examined perceptions and knowledge about PC and attitudes towards screening among male teachers in the Sunyani Municipality | Cross sectional study | Journal article | Ghana | NR | Community based survey | May to July 2015 | Demographics, Patient access, Patient behavior | No |

Abbreviations: AA: African American; AAW: Arab American women; ADR: Adenoma detection rate; AIAN: American Indian/Alaska Native; AMA: American Medical Association; APDC: Admitted Patients Data Collection; AIDS: Acquired Immunodeficiency Syndrome; AWHC: African Women's Health Center; BA: Breast awareness; BC: Breast cancer; BCS: Breast cancer screening; BCRCSP: Barcelona colorectal cancer screening program; BCSBQ: Breast Cancer Screening Beliefs Questionnaire; BCSP: the National Health Service; National Bowel Cancer Screening Program; BCSR: Breast Cancer Family Registry; BMI: Body mass index; BRFSS: Behavioral Risk Factor Surveillance System; BSE: Breast self exam; BWH: Brigham and Women’s Hospital; CALD: Culturally and linguistically diverse; CAM: Cancer Awareness Measure; CBE: Clinical breast exam; CC: Cervical cancer; CCAE: Commercial Claims and Encounters; CCDRFS: Chinese Chronic Disease and Risk Factor Surveillance; CCHS: Canadian Community Health Survey; CCMU: Chatapadung Contracting Medical Unit; CCP: Cervical cancer prevention; CCS: Cervical cancer screening; CCSB: Cervical Cancer Screening Behavior; CCST: Cervical Cancer Screening and Treatment; CheReL: Centre for Health Record Linkage; CIHI: Canadian Institute for Health Information; COMPASS: Chicago Multiethnic Prevention and Surveillance Study; CPRIT: Cancer Prevention and Research Institute of Texas; CPRS: Computerized Patient Record System; CRC: Colorectal cancer; CRCS: Colorectal cancer screening; CVD: Cardiovascular disease; DAD: Discharge abstract database; DC: District of Colombia; DCCR: Washington DC cancer registry; DCCSD: Danish Colorectal Cancer Screening Database DHS: Demographic Health Survey; DK: “I don’t know”; EHR: Electronic health record; ELSA: English Longitudinal Study of Ageing; ERPR: External Peer Review Program; ESPS: French Health, Healthcare and Insurance Survey (French); EU: Europe; FDR: First degree relatives; FFS: Fee-for-service; FHT: Family health team; FIT: Faecal immunochemical test; FOBT: Faecal occult blood test; FNBCSP: French National Breast Cancer Screening Programme; FQHC: Federally Qualified Health Centre; FS: Flexible sigmoidoscopy; GG&C: Greater Glasgow and Clyde; GP: General practitioner; HBM: Health Belief Model; HDSS: Health and demographic surveillance site; HCS: Hunter Community Study; HINTS: Health Information National Trends Survey; HIV: Human immunodeficiency virus; HOS: Health Omnibus Survey; HPV: Human papillomavirus; HRS: Health and Retirement Study (US); HWS: Health and Welfare Survey; ICC: Immunocytochemistry; ICES: Clinical Evaluative Sciences; IDD: Intellectual and developmental disabilities; KA: Korean-American; KSA: Kingdom of Saudi Arabia; KNCSS: Korean National Cancer Screening Survey; KHPS: Keio Household Panel Survey; LE: Life expectancy; LEP: Limited English proficiency; LHL: low health literacy; LTC: Long-term condition; MBMC: Masculinity barriers to medical care; MCBS: Medicare Current Beneficiary Survey; MDR: Military Data Repository; MENA: Middle East and North Africa; MEPS: Medical Expenditure Panel Survey; MDC: Midwives Data Collection; MedPAR: Medicare Provider Analysis and Review; MHS: Military Health System; MMG: Mammography; MPHIA: Malawi Population HIV Impact Assessment; MS: Mammography screening; NAC: Naturalized citizen; NDHS: Namibia Demography and Health Survey; NHID: National Health Information Database; NHIS: National Health Interview Survey; NHS: National Health Service; NHS: National Health Survey; NHW: Non-Hispanic White; NIMHD: National Institute on Minority Health and Health Disparities Research Framework; NOC: Non-citizen; NR: Not reported; NSFG: National Survey of Family Growth; NSW: New South Wales; NYC: New York City; NYCCHS: New York City Community Health Survey; OBGYN: Obstetrician-gynecologists; OCR: Ontario Cancer Registry; OHIP: Ontario Health Insurance Plan; OutSAF: Outpatient Standard Analytical Files; PAP: Papanicolaou; PAPM: Precaution Adoption Process Model; PCMH: Patient-centred medical home; PCP: Primary care provider; PHC: Primary healthcare; PHF: Public health facility; PLE: Perceived life expectancy; PMT: Protection motivation theory; PNA: Previous non-attender; PR: Puerto Rico; PSA: Prostate-specific antigen; PTR: Pap Test Register; PTSD: Post-traumatic stress disorder; RAPID: Reporting, Analytics, Performance, Improvement & Deployment; RPDB: Registered Persons Database; RHS: Reproductive Health Survey; SAGE: Study on AGEing and adult health; SDOH: Social determinant of health; SEC: Socio-economic class; SEER: Surveillance, Epidemiology and End Results; SES: Socio-economic status; SHIS: Saudi Health Interview Survey; SHS: Swiss Health Survey; SIRS: Health, Inequalities and Social Ruptures (French); SLE: Systemic lupus erythematosus; SNNPR: Southern Nations, Nationalities, and Peoples' Region; SOC: States of Change model; SPMH: Self-perceived mental health status; SSA: Sub-Saharan Africa; STI: Sexually transmitted infection; TASO: AIDS Support Organization; TTC-CRC-SP: Tallaght Hospital/Trinity College Dublin Colorectal Cancer Screening Program; TTM: transtheoretical model; UAE: United Arab Emirates; UK: United Kingdom; US: United States; USA: United States of America; USB: US born citizens; USPSTF: United States Preventive Services Task Force; UTD-CRC: Up-to-date colorectal cancer; VA: Virginia; VHA: Veteran’s Health Association; VIA: Visual inspection with acetic acid; WHO: World Health Organisation; WLHIV: Women living with human immunodeficiency virus; WRHA: Western Regional Health Authority; YHS: Yorkshire Health Study ;

*It refers to whether the study reported information on interventions that could help overcome the barriers to screening: YES/NO

**Table 5. Study characteristics of studies reporting OR outcomes for more than one tumor type**

| **Study name** | **Indication** | **Study Objectives** | **Study design** | **Publication Type** | **Country** | **Data Source** | **Study setting** | **Data collection period** | **Outcomes: Barriers** | **Outcomes: Interventions [YES/NO]** |
| --- | --- | --- | --- | --- | --- | --- | --- | --- | --- | --- |
| Akinyemiju 2012 (547) | Breast cancer, Cervical cancer | To assess the influence of household socio-economic status (SES), healthcare access and country level characteristics on breast and cervical cancer screening among women in developing countries. | Cross-sectional study | Journal article | 15 developing countries | The 2002–2003 World Health Survey (WHS) | International survey | 2002-2003 | Demographics, Economic, Patient access, PCP access, Social | No |
| Akinyemiju 2016 (548) | Breast cancer, Cervical cancer | To examine individual, parental and life-course SES differences in breast and cervical cancer screening among women in India, China, Mexico, Russia and South Africa | Cross-sectional study | Journal article | China, India, Mexico, Russia and South Africa | WHO’s Study on Global Ageing and Adult Health (SAGE) 2007–2008 | International study | 2007-2008 | Economic, Social | No |
| Al Rifai 2015 (549) | Breast cancer, Cervical cancer | This study sought to assess the inequalities in breast and cervical cancers screening rates among ever-married women aged 20-49 years according to the household wealth status in Jordan and to analyze the possible social predictors contribute substantially to a low cancer screening rate. | Cross sectional study | Journal article | Jordan | Jordan Breast cancer program | National survey | 2012 | Demographics, Economic, Patient access, Patient behavior, Social | No |
| Anwar 2018 (550) | Breast cancer, Cervical cancer | To evaluate potential determinants of awareness about and participation in breast and cervical cancer screening, and breast self-examination (BSE) in women using survey data from Indonesia. | Cross-sectional longitudinal household survey | Journal article | Indonesia | Indonesian Family Life Survey (IFLS) | Nationwide study | 2014 to 2015 | Demographics, Economic, Patient access, Patient behavior, Social | No |
| Aschwanden 2019 (551) | Breast cancer, Cervical cancer, Colorectal cancer, Prostate cancer | The present study investigated the cross-sectional associations between personality traits and the probability of obtaining a recent preventive screening for breast, cervical, prostate, and colorectal cancer. | Cross-sectional study | Journal article | USA | Health and Retirement Study | Nationwide study | 2010–2012 | Patient behavior | No |
| Azhar 2022 (552) | Breast cancer, Cervical cancer | To answer the following research questions: (1) Is perceived ethnic discrimination of Muslim American women associated with the likelihood of obtaining an up-to-date breast or cervical cancer screening? (2) Does spiritual health locus of control impact the likelihood of obtaining an up-to-date breast or cervical cancer screening? | Cross-sectional study | Journal article | USA | NR | Community wide study | Mar 2017 to Jun 2018 | Demographics, Patient access, Patient behavior, Social | No |
| Batai 2020 (553) | Colorectal cancer, Prostate cancer | To evaluate cancer screening behaviors in 102 Hopi men who were 50 years of age or older from the Hopi Survey of Cancer and Chronic Disease. | Cross-sectional, population-based survey | Journal article | USA | Hopi Survey of Cancer and Chronic Disease, a population-based survey of randomly selected adult Hopi members (age 18 or older) living on the reservation | Region wide study | Jun 2012 to Dec 2012 | Demographics, Economic, Patient access, Patient behavior, PCP access, Social | No |
| Bertaut 2018 (554) | Breast cancer, Multiple cancers | This study aimed to determine participation rates and factors associated with participation in colorectal (fecal occult blood test) and cervical cancer (Pap-smear) screening among a population of women participating in breast cancer screening | Cross-sectional study | Journal article | France | NR | Community based survey | August to October 2015 | Demographics, Patient access, PCP access | No |
| Beyer 2016 (555) | Breast cancer, Colorectal cancer | This study examines the relationship between several aspects of the perceived neighborhood environment and breast and colorectal cancer screening behavior among a population-based sample of Wisconsin residents. | Cross-sectional study | Journal article | USA | Survey of the Health of Wisconsin (SHOW) | Statewide study | 2008-2012 | Patient behavior, Social | No |
| Bringedal 2019 (556) | Breast cancer, Cervical cancer, Colorectal cancer, Lung cancer, Ovarian cancer, Prostate cancer | This study examines the guidelines for cancer screening have been debated and are followed to varying degrees. We wanted to study whether and why doctors recommend disease specific cancer screening to their patients. | Cross-sectional study | Journal article | Norway | Representative sample of doctors practicing in Norway | National survey | 2014/2015 | PCP access | No |
| Bussière 2014 (557) | Breast cancer, Cervical cancer | To disentangle the effects of obesity and mobility limitation on cervical and breast cancer screening among community dwelling women. This study contributes to the literature by exploring the joint and separate effects of obesity and mobility limitation on participation in cervical and breast cancer screening programs among community-dwelling women in France. | Cross sectional study | Journal article | France | French national Health and Disability Survey - Household Section | National survey | April to September of 2008 | Demographics, Economic, PCP access, Social | No |
| Camacho-Rivera 2019 (558) | Colorectal cancer, Prostate cancer | The aims of this study were (1) to identify social and demographic patterns of prostate cancer and colorectal cancer screening and (2) to examine the associations between smartphone and health app use and screening behaviors among CHIP participants. | Cross-sectional study | Journal article | USA | Cancer Health Impact Program (CHIP) (National Cancer Institute’s Health Information National Trends Survey (HINTS) Cycles 2 and 4, ) | Nationwide study | May 2017- Feb 2018 | Demographics, Economic | No |
| Carrasco-Garrido 2014 (559) | Breast cancer, Cervical cancer, Colorectal cancer, Prostate cancer | To describe levels of awareness and uptake of colorectal, breast, cervical and prostate cancer screening tests and to analyze the association to socio-demographic and health-related variables. | Cross-sectional population-based study | Journal article | Spain | Home-based personal interview survey-Oncobarometro Survey (OS) | Nationwide study | Nov 2010 to Dec 2010 | Demographics, Economic, Patient access, PCP access, Social | No |
| Carrozzi 2015 (560) | Breast cancer, Cervical cancer, Colorectal cancer | To estimate actual population coverage for the three types of screening the extent of spontaneous screening needs to be known. | Cross-sectional study | Journal article | Italy | PASSI a national telephone-interview surveillance system | National survey | 2010 to 2013 | Demographics, Economic, Social | No |
| Charkhchi 2019 (561) | Breast cancer, Cervical cancer, Colorectal cancer | To assess the impact of sexual orientation and gender identity (SOGI) on breast, cervical, and colorectal cancer screening adherence, we conducted an analysis of the Behavioral Risk Factor Surveillance System (BRFSS) | Cross sectional survey | Journal article | USA | Behavioral Risk Factor Surveillance System (BRFSS) | National survey | 2016 | Demographics, Economic, Patient access, Social | No |
| Constantinou 2016 (562) | Breast cancer, Cervical cancer | To identify chronic conditions associated with adherence to cervical and breast cancer screening recommendations in France and to investigate whether these associations were modified by several major cancer screening determinants. Our secondary objective was to explore whether the associations between chronic conditions and breast cancer screening participation were specific to opportunistic or organized screening. | Cross-sectional study | Journal article | France | Healthcare and Health Insurance Survey (Enquête Santéet Protection Sociale), | National survey | 2008 | Demographics | No |
| Dallo 2015 (563) | Breast cancer, Cervical cancer | To estimate and compare the age adjusted prevalence of not receiving a flu or pneumonia vaccine, clinical breast examination, mammogram or Pap smear among U.S.- and foreign-born White women by region of birth and examine associations while controlling for potential confounders. | Cross-sectional study | Journal article | USA | 12 years of National Health Interview Survey data | Nationwide study | 2000-2011 | Demographics | No |
| Damiani 2012 (564) | Breast cancer, Cervical cancer | The aim of this study was to evaluate the impact of socioeconomic disparities in the uptake of female screening in Italy, with a specific focus on different types of screening programs. | Cross-sectional study | Journal article | Italy | National health interview survey | Nationwide study | Dec 2004 to Mar 2005 | Demographics, Economic, Patient behavior, Social | No |
| Dawidowicz 2020 (565) | Breast cancer, Cervical cancer, Colorectal cancer, Multiple cancers | The primary objective of the present study was to describe patterns and rates of non-participation or partial participation in combinations of screening programs for all three cancers by French women aged 50 to 65 years. The secondary objective was to investigate individual and contextual factors associated with non-participation or partial participation. | Retrospective cohort study | Journal article | France | ADOC94 databases | Multicenter | Nov 2010 and Oct 2012 | Demographics, Economic, Patient access | No |
| Deshpande 2012 (566) | Breast cancer, Colorectal cancer | The objective of the current study was to examine the independent associations between three commonly used health status indicators—perceived health status, number of chronic health conditions (as a measure of chronic disease burden), and the presence of a functional limitation due to a chronic condition—and breast and CRC screening. Findings from this study may help identify specific population subgroups that are under- or over-screened for cancer based on health status and inform clinical and public health initiatives on using health status indicators to target cancer screening efforts for those most likely to benefit. We sought to answer two main research questions: 1) is each of the health status indicators independently associated with cancer screening behavior after controlling for each other and other correlates of breast and CRC screening; and 2) are observed associations similar across health status indicators and cancer screening type | Cross-sectional study | Journal article | USA | National Health Interview Survey (NHIS) | National survey | 2005 | Demographics | No |
| Documet 2015 (567) | Breast cancer, Cervical cancer | To a) determine if an association exists between social support and compliance with mammography and Pap test screening guidelines; and b) test whether social support moderates the effect of education on breast and cervical cancer screening | Cross-sectional study | Journal article | USA | NR | County and community-based survey | 2009-2010 | Social | No |
| Donley 2020 (568) | Breast cancer, Cervical cancer | It seeks to determine (1) whether there are any differences in uptake in breast and cervical cancer screenings within and between groups on the basis of nativity, (2) what factors are associated with breast and cervical cancer screenings among U.S.- and foreign-born women, and (3) whether perceived risk or history of cancer predicts cancer screening rates among U.S.- and foreign-born women | Cross-sectional study | Journal article | USA | National Health Interview Survey | Nationwide study | 2019 to 2015 | Demographics, Economic, Patient access, Patient behavior, PCP access, Social | No |
| Eng 2020 (569) | Breast cancer, Cervical cancer, Colorectal cancer | To assess the dose-dependent relationship between smoking history and cancer screening rates or staging of cancer diagnoses | Prospective, population-based cohort study | Journal article | USA | Questionnaire responses from the Women’s Health Initiative (WHI) Observational Study. | Multicenter | 1 Oct 1993 to 31 Dec 1998 | Patient behavior | No |
| Farzaneh 2017 (570) | Breast cancer, Cervical cancer | To assess BSE, CBE, mammography, and Pap smear screening behaviors among Azeri females (living in Ardabil, northwest Iran) and to study associations among these screening behaviors with demographic and cognitive variables, such as self-efficacy, barriers, and attitudes toward breast and cancer screening. | Cross-sectional study | Journal article | Iran | NR | Community wide study | March–June 2016 | Demographics, Economic, Patient behavior, Social | No |
| Fujiwara 2018 (571) | Breast cancer, Cervical cancer, Colorectal cancer, Gastric cancer, Lung cancer | The objectives of the current study were: 1) to investigate the association between serious PD (SPD) and participation in cancer screening in the general population, and 2) to explore the modifying effect of SES on the association between SPD and participation in cancer screening. | Cross-sectional study | Journal article | Japan | Comprehensive Survey of Living Conditions (CSLC) | Nationwide study | 2010 | Demographics, Economic | No |
| Gawron 2021 (572) | Breast cancer, Cervical cancer, Colorectal cancer | To use the Medical Expenditure Panel Survey (MEPS) to identify potential difficulties with access to CRC screening. | Cross-sectional study | Journal article | USA | Medical Expenditure Panel Survey | Nationwide study | 200-2016 | Demographics, Economic, Patient access, Patient behavior, PCP access | No |
| Goodwin 2020 (573) | Breast cancer, Cervical cancer, Colorectal cancer, Prostate cancer, Skin cancer | To identify whether cancer-related health behaviors including participation in cancer screening vary by geographic location in Australia. | Cross-sectional study | Journal article | Australia | Data were obtained from the 2014-2015 Australian National Health Survey; telephone interview-based study | Nationwide study | 2014 to 2015 | Economic, Patient access | No |
| Guo 2021b (574) | Breast cancer, Colorectal cancer, Multiple cancers | This study examined the influence of sociodemographic factors on cancer fatalistic beliefs and test whether the fatalistic beliefs are associated with undergoing breast and colorectal cancer screening utilization among individuals residing in a large southeastern academic medical university's catchment area in north-central Florida region | Cross-sectional study | Journal article | USA | NR | Community based survey | June-September 2017 | Demographics, Economic, Patient access, Patient behavior, Social | No |
| Gutarra 2021 (575) | Breast cancer, Cervical cancer, Colorectal cancer | To assess perspective on cancer information-seeking behavior, demographic, economic, healthcare access and COVID-related factors among Latino adults (>18 living in Indiana able to read and write in Spanish or English. | Cross-sectional study | Journal article | USA | NR | Statewide study | NR | Demographics, Economic, Patient access, Social | No |
| Haas 2016 (576) | Breast cancer, Cervical cancer | To characterize women’s primary health care provider attitudes towards screening and changes in practice in response to recent revisions in guidelines for breast and cervical cancer screening. | Cross-sectional study | Journal article | USA | PROSPER | Multicenter | 2014 | Demographics, PCP access | No |
| Hanske 2016 (577) | Breast cancer, Cervical cancer, Colorectal cancer | To examine the impact of marital status on the use of screening for breast, cervical, and colorectal cancer. | Cross-sectional study | Journal article | USA | 2012 Behavioral Risk Factor Surveillance System Survey age-appropriate screening cohorts | Nationwide study | 2012 | Demographics | No |
| Harcourt 2014 (578) | Breast cancer, Cervical cancer | To determine the rates of participation in breast and cervical cancer screening among age eligible female African immigrants in the Twin Cities and to examine barriers associated with these cancer screening procedures | Cross-sectional study | Journal article | USA | NR | Community wide study | 2006-2007 | Demographics, Economic, Patient access, Patient behavior, Social | No |
| Herriges 2021 (579) | Breast cancer, Cervical cancer, Prostate cancer | To investigate whether SO affects prevalent gender-specific cancer screening and prevalence, including prostate (PCa), breast (BC), and cervical cancer (CC) | Cross-sectional study | Conference abstract | USA | Health Information National Trends Survey (HINTS) database (part of the National Cancer Institute’s division of cancer control and population sciences) between 2017-2019 | Nationwide study | 2017-2019 | Demographics | No |
| Holden 2014 (580) | Cancer not specified, Colorectal cancer, Ovarian cancer | To hypothesize that 1) rates of depressive symptoms in this group will exceed those in both the general population and 2) depressive symptoms as well as Latino-specific cultural aspects will be negatively associated with screening for other cancers.  To determine prevalence of depressive symptoms and its role in screening for other cancers in Latina breast cancer survivors. | Cross-sectional study | Journal article | USA | Subjects were part of a larger study population who participated in an analysis of the psychosocial needs of Latino families adjusting to breast cancer diagnosis. Latinas ranging from 25 to 79 years old were recruited through San Antonio, Texas, breast cancer clinics, organizations, and support groups that included the Susan G. Komen Breast Cancer Foundation San Antonio affiliate, Alamo Breast Cancer Foundation, and the American Cancer Society | Statewide study | NR | Demographics, PCP access | No |
| Horner-Johnson 2014a (581) | Breast cancer, Cervical cancer | To examine the relationship of disability severity to receipt of mammography and Pap smears in a nationally representative sample, and to assess the extent to which other variables account for any differences between disability groups. We evaluated the following hypotheses: 1. Compared with women without disabilities, women with basic or complex activity limitations would be less likely to be up to date with screening recommendations. 2. The magnitude of the disparity would be greatest for women with the most complex limitations. 3. Controlling for other demographic and socioeconomic characteristics would reduce the magnitude of the apparent disparity | Cross sectional study | Journal article | USA | Household component of the Medical Expenditure Panel Survey (MEPS) | National survey | 2002-2008 | Demographics, Economic, Patient access, Social | No |
| Horner-Johnson 2015 (582) | Breast cancer, Cervical cancer | To examine the combination of disability status and rurality in association with receipt of breast and cervical cancer screening among women aged 18 to 64 in the United States. | Cross-sectional study | Journal article | USA | Medical Expenditure Panel Survey using pooled annual data files | Nationwide study | 2002 to 2008 | Demographics, Economic, Patient access, Social | No |
| Huang 2021b (583) | Breast cancer, Cervical cancer, Colorectal cancer | To clarify this issue by investigating the influence of health literacy on cancer screening behaviors in Taiwanese females. | Cross-sectional study | Journal article | Taiwan | The Mandarin version of the European Health Literacy Survey Questionnaire and the Taiwanese National eHealth Database. | Nationwide study | February to October 2015 | Demographics, Economic, Patient behavior, Social | No |
| Ishii 2021 (584) | Breast cancer, Cervical cancer, Colorectal cancer, Multiple cancers | To examine combined patterns of participation in these three screenings and investigate the factors associated with non-participation in each. | Cross-sectional study | Journal article | Japan | 2016 Comprehensive Survey of Living Conditions of People on Health and Welfare | Nationwide study | 2016 | Demographics, Economic, Patient behavior, PCP access, Social | No |
| Jacobs 2014 (585) | Breast cancer, Cervical cancer | To understand the relationship between perceived everyday racial/ethnic and other discrimination and receipt of breast and cervical cancer screening in a multiethnic population of women. | Prospective Longitudinal cohort study | Journal article | USA | NR | Multicenter | 1996 to 2000 | Demographics, Patient behavior | No |
| Jolidon 2021 (586) | Breast cancer, Cervical cancer | To examine how welfare provision and healthcare system features modify cancer screening uptake and inequalities across European countries. | Cross-sectional study | Journal article | Europe | European  Health Interview Survey (EHIS) 2014 wave and Swiss Health Interview Survey (SHIS) 2012 | International study | 2013-2015 | Demographics, Economic, Patient access, PCP access, Social | No |
| Jones 2021 (587) | Breast cancer, Cervical cancer | To examine the effects of different types of financial anxiety on adherence to breast cancer screening in women at high risk of breast cancer. Adherence to cervical cancer screening was also examined to determine whether associations between financial anxiety and screening adherence were unique to breast cancer screening or more general. | Cross-sectional study | Journal article | USA | Participants were recruited through Facebook advertisements, targeted toward women aged 30–50 with interests in breast cancer | National survey | August to September of 2019 | Economic, Patient behavior | No |
| Kang 2014 (588) | Breast cancer, Cervical cancer, Colorectal cancer, Gastric cancer, Liver cancer, Multiple cancers | To investigate factors associated with participation in organized and opportunistic cancer screening programs, with a particular focus on socioeconomic factors | Cross-sectional study | Journal article | Korea | Korea National Health and Nutrition Examination Survey (KNHANES) | Nationwide study | 2007 to 2011 | Demographics, Economic, Patient access, Patient behavior, Social | No |
| Karadeniz 2019 (589) | Breast cancer, Cervical cancer, Colorectal cancer, Prostate cancer | To determine early cancer detection/screening behaviors of individuals and the influencing factors. | Cross-sectional study | Journal article | Turkey | NR | City wide population based | Dec 2011- 30th Jan 2012 | Demographics, Economic, Patient access, Social | No |
| Kelly 2017 (590) | Breast cancer, Cervical cancer, Colorectal cancer | To identify the socioeconomic inequalities, which persisted in screening uptake for these cancers, and to quantify these disparities over a 5-year period. | Cross-sectional study | Journal article | France | NR | Nationwide surveys | 2005-2010 | Demographics, Economic, Patient access, Social | No |
| Kim 2018 (591) | Breast cancer, Prostate cancer | To analyze the association between seeking information online about breast and prostate cancer and undergoing mammography and PSA screening | Cross-sectional study | Journal article | USA | Institutional database | Single center | 2012 to 2014 | Demographics, Economic, Patient access, PCP access, Social | Yes |
| Kim 2019a (592) | Breast cancer, Cervical cancer | To examine the association between health literacy and breast and cervical cancer screening using data from the Behavioral Risk Factor Surveillance System (BRFSS) that uses a nationally representative U.S. probability sample. | Cross-sectional, correlational study | Journal article | USA | Behavioral Risk Factor Surveillance System (BRFSS) (random digit-dialed landline and cellular telephone household survey) | Nationwide study | 2016 | Demographics, Economic, Patient access, PCP access, Social | No |
| Kim 2019b (593) | Breast cancer, Cervical cancer, Colorectal cancer, Gastric cancer | This study evaluated adherence to general medical checkup and cancer screening recommendations in heavy smokers who met NLST criteria. | Cross-sectional study | Journal article | Korea | Fifth Korea National Health and Nutrition Examination Survey (KNHANES V) | Nationwide study | 200-2012 | Patient behavior | No |
| Kindratt 2020 (594) | Breast cancer, Cervical cancer, Colorectal cancer | To estimate how associations between adults’ perceptions of specific domains of PPC quality and their likelihood of receiving cancer screenings differ by race and ethnicity | Cross-sectional study | Journal article | USA | Medical Expenditure Panel Survey (MEPS) | Nationwide study | 2011 to 2015 | PCP access | No |
| Kiran 2019 (595) | Breast cancer, Cervical cancer, Colorectal cancer | To compare rates of cervical, breast, and colorectal cancer screening between patients who are transgender and those who are cisgender (i.e., nontrans gender). | Cross-sectional study | Journal article | Canada | NA | Multicenter | June and July 2016 | Demographics | No |
| Kurani 2020 (596) | Breast cancer, Cervical cancer, Colorectal cancer | To examine the association between area deprivation, rurality, and screening for breast, cervical, and colorectal cancer in patients from an integrated health care delivery system in 3 US Midwest states (Minnesota, Iowa, and Wisconsin). | Retrospective cross-sectional study | Journal article | USA | Mayo Clinic or Mayo Clinic Health System electronic health records | Multicenter | 01 Jul 2016 to 30 Jun 2017 | Demographics, Economic | Yes |
| Kushalnagar 2019 (597) | Breast cancer, Cervical cancer | To assess whether disparities for cancer screening adherence persist for Deaf women compared with the general population and whether racial and ethnic disparities for adherence exist among Deaf women | Cross-sectional survey | Journal article | USA | Health Information National Trends survey (HINTS-ASL survey) | National survey | February 2017–August 2017 and October 2017–May 2018 | Demographics, Economic, Patient access, Social | No |
| Lee 2020 (598) | Breast cancer, Cervical cancer, Colorectal cancer | To measure the rates of receiving three types of cancer screening services, Pap test, mammogram and colorectal cancer screening, among patients seen at U.S. health centers (HCs) to investigate if cancer screening among patients varies by race/ethnicity and to test if cancer prevention provided by HCs could mitigate health disparities caused by racial/ethnic differences. | Cross-sectional survey | Journal article | USA | 2014 U.S. Health Center Patient Survey | National survey | 2014 | Demographics, Patient access, Patient behavior, Social | No |
| Lee 2021 (599) | Breast cancer, Cervical cancer, Colorectal cancer | The study assessed the impact of access to care, use of services, and health literacy on cancer screening among Korean Americans | Descriptive cross-sectional study | Journal article | USA | NR | Community wide study | September 2018 to December 2018 | Economic, Patient access, Social | Yes |
| Lo 2013 (600) | Breast cancer, Cervical cancer, Colorectal cancer | To assess the uptake of cancer screening tends to be lower for colorectal cancer (CRC) than cervical or breast cancer. Dislike of the test itself has often been identified as a barrier to CRC screening with the Fecal Occult Blood (FOB) test, but there have been no head-to-head comparisons of the three tests | Cross-sectional study | Journal article | UK | TNS omnibus survey | International wide study | 2012 | Demographics, Economic | No |
| Lofters 2015 (601) | Breast cancer, Cervical cancer, Colorectal cancer | To inform physician- targeted screening interventions by identifying the characteristics of primary care physicians in Ontario that are associated with cancer screening for their eligible patients, for their eligible immigrant patients, and for IMG physicians, for their eligible immigrant patients from the same region of the world | Retrospective cohort study | Journal article | Canada | Citizenship and Immigration Canada (CIC), Registered Persons Database, Ontario Physicians’ Claims Database, Ontario Cancer Registry, Canadian Institute of Health Information Discharge Abstract Database, Client Agency Program Enrollment database, Institute for Clinical Evaluative Sciences’ Physicians’ Database | Nationwide study | 1985-2010 | Demographics, Economic, Patient access, PCP access | No |
| Lofters 2019 (602) | Breast cancer, Cervical cancer, Colorectal cancer | To describe the Ontario population who are screen-eligible for each of breast, cervical and colorectal cancers but overdue for screening by at least five years, in order to highlight subpopulations and regions in Ontario with the most urgent needs for screening interventions. | Retrospective cohort study | Journal article | Canada | Provincial-level administrative datasets | Province wide population-based study | 2017 | Demographics, Economic, Patient access | No |
| MacDonald 2022 (603) | Breast cancer, Cervical cancer, Colorectal cancer | This study examines the rates of surveillance and screening of uninsured cancer survivors and compares to uninsured patients without a cancer history seen in free clinics. | Retrospective cohort study | Journal article | USA | Medical charts of all uninsured patients who visited ten free clinics in the Tampa Bay area over the course of 3 calendar years | Multicenter | 2016 to 2018 | Demographics, Economic | No |
| Malhotra 2017 (604) | Breast cancer, Cervical cancer, Colorectal cancer | This study assessed the role of patient–provider race, ethnicity, or gender concordance in cancer screening have been inconclusive. | Cross-sectional study | Journal article | USA | Medical Expenditure Panel Survey (MEPS) | National survey | 2003-2010 | Demographics, PCP access | No |
| Martinez-Huedo 2012 (605) | Breast cancer, Cervical cancer | To assess the adherence to breast and cervical cancer screening of women with diabetes mellitus (DM), and the associated factors and trend of use over time of these preventative services between 2006 and 2010 in Spain | Cross-sectional study | Journal article | Spain | European Health Interview Survey in Spain (EHISS, 2009). | National survey | April 2009 and March 2010 | Demographics, Economic, Patient behavior, PCP access, Social | No |
| Massat 2015 (606) | Breast cancer, Cervical cancer | To characterize districts with atypical coverage levels for cervical or breast screening. | Cross-sectional observational study | Journal article | England | Health & Social Care Information Centre (HSCIC). | Single center | Apr 2011 to Mar 2012 | Demographics, Economic, PCP access, Social | No |
| Mehta 2022 (607) | Breast cancer, Colorectal cancer | To determine the association of county-level SVI with breast and colon cancer screening rates | Retrospective cohort study | Journal article | USA | CDC 2018 SVI database, 2018 Behavioral Risk Factor Surveillance System (BRFSS) and National Health Interview Survey (NHIS) | Nationwide study | NR | Social | No |
| Mendoza 2022 (608) | Breast cancer, Colorectal cancer | To access hypothesis that FI and multiple other measures of socioeconomic status and SDOH were used to determine their unique influence on being “up to date” with colorectal cancer and breast cancer screening guidelines. | Cross-sectional study | Journal article | USA | NR | Multicenter | 2019 | Patient behavior | No |
| Menvielle 2014 (609) | Breast cancer, Cervical cancer | To investigate the association between women’s economic situation and breast and cervical cancer screening | Cross-sectional, national health survey | Journal article | France | French national health survey, telephone survey | Nationwide study | Oct 2009 to Jul 2010 | Demographics, Economic, Patient access, Patient behavior, PCP access, Social | No |
| Menvielle 2018 (610) | Breast cancer, Cervical cancer, Colorectal cancer | The objective of this study was to investigate the role of socioeconomic and health care use characteristics in the participation in breast, cervical and colorectal cancer screening in the French West Indies. | Cross sectional study | Journal article | France | National health survey for two French overseas territories | National survey | 2014 | Demographics, Economic, Patient access, PCP access, Social | No |
| Milenkov 2020 (611) | The purpose of this cross-sectional analysis was to assess differences in uptake of cervical, breast, liver, and colorectal screens across six cultural groups. | Cross-sectional study | Journal article | USA | NR | Community wide study | 2018 | Demographics | No | The purpose of this cross-sectional analysis was to assess differences in uptake of cervical, breast, liver, and colorectal screens across six cultural groups. |
| Miles 2021 (612) | Breast cancer, Colorectal cancer, Lung cancer | This study aimed to estimate the proportion of patients visiting the emergency department (ED) who were not up to date with cancer screening guidelines to assess the scope of need and potential impact of ED-based cancer screening interventions | Cross-sectional study | Journal article | USA | National Health Interview Survey (NHIS) | National survey | 2015 | Demographics, Economic, Patient access, Social | No |
| Mo 2014 (613) | Breast cancer, Cervical cancer, Colorectal cancer, Prostate cancer | To examine the utilization of breast, cervical, prostate, and colorectal cancer screening among PSMI in Hong Kong and to identify factors associated with their screening behaviors | Cross-sectional study | Journal article | Hong Kong | NR | Multicenter | NR | Demographics, Economic, Patient access, Patient behavior, Social | No |
| Moreno 2019 (614) | Breast cancer, Cervical cancer, Colorectal cancer, Prostate cancer | This study aimed to examine cancer fatalism (i.e., the belief that there is little or nothing one can do to lower his/her risk of developing cancer) as a determinant of adherence to national screening guidelines for colorectal, breast, prostate, and cervical cancer among Hispanics/Latinos. We hypothesized that lower cancer fatalism would be associated with greater adherence to cancer screenings for all four cancer types, even when adjusting for sociocultural factors that have been shown to be associated with screening behavior (i.e., health insurance, income, education, acculturation). | Cross-sectional study | Journal article | USA | National Cancer Institute (NCI) Health Interview National Trends Survey | Nationwide study | 2008-2011 | Demographics, Economic, Patient access, Social | No |
| Moss 2019 (615) | Breast cancer, Cervical cancer, Colorectal cancer, Multiple cancers, Prostate cancer | To understand how rurality and racial segregation are independently and interactively associated with cancer screening and cancer | Cross-sectional study | Journal article | USA | National Cancer Institute’s Health Information National Trends Survey, 2011–2017 | Nationwide study | 2011-2017 | Demographics, Economic, Social | No |
| Moss 2022 (616) | Cervical cancer, Colorectal cancer, Multiple cancers | To give insights to guide future interventions to increase screening uptake in primary care settings, particularly in underserved communities. | Cross-sectional study | Journal article | USA | NR | Community based survey | Between March and June 2020 | Economic, Patient access, Patient behavior, Social | No |
| Mukem 2015 (617) | Breast cancer, Cervical cancer | This study identified socioeconomic and contextual factors contributing to the variation in screening uptake and coverage | Cross sectional study | Journal article | Thailand | Health and Welfare Survey (HWS) 2007 and the Reproductive Health Survey (RHS) | National survey | HWS: 2007 and 2009 | Demographics, Economic, Patient access, Social | No |
| Murphy 2021 (618) | Breast cancer, Cervical cancer, Colorectal cancer, Prostate cancer | The study (1) examines cancer screening rates in a national sample of commercially insured adults, and (2) identifies perceived barriers and facilitators to cancer screening for the SMI and general population through interviews with primary care providers (PCPs) and psychiatrists | Retrospective study | Journal article | USA | Market Scan commercial claims data | National registry-based study | 2010–2017; Quantitative survey; February and April 2019: Qualitative survey | Demographics, Economic, Patient access, PCP access | No |
| Nguyen 2022 (619) | Colorectal cancer, Prostate cancer | To compare PSA screening to CRC screening from 2012-2020. Data was stratified by income, geographic region, race and health insurance. | Cross-sectional study | Journal article | USA | BRFSS, a national database of surveys | Nationwide study | 2012-2020 | Economic, Patient access | No |
| Nicholls 2017 (620) | Breast cancer, Cervical cancer, Colorectal cancer, Skin cancer | To identify the personal cancer screening behaviors of nurses and midwives in New South Wales, Australia, and identify factors predictive of cancer screening uptake. | Cross-sectional study | Journal article | Australia | Data were obtained from the ‘Fit for the Future’ study on working nurses and midwives in New South Wales, Australia, and analyses were conducted on subsets of age-eligible respondents | Statewide survey | June 2014 to February 2015 | PCP access | No |
| Niedzwiedz 2019 (621) | Breast cancer, Cervical cancer | To assess how depressive symptoms (overall and individual symptoms) are associated with participation in breast and cervical cancer screening within the UK | Prospective cohort study | Journal article | UK | UK Biobank study | Nationwide study | 2006- 2010 | Demographics | No |
| Niedzwiedz 2020 (622) | Breast cancer, Cervical cancer | This study had four key objectives: (a) to investigate the cross‐sectional associations between depressive symptoms (overall and item‐specific questions) and participation in breast and cervical screening; (b) to examine the cross‐sectional associations between neuroticism (overall score and item‐specific questions) and participation in breast and cervical screening; (c) to examine whether baseline depressive symptoms and neuroticism scores predict future participation in breast and cervical screening; and (d) to investigate whether baseline depressive symptoms and neuroticism scores relate to longitudinal patterns of breast and cervical screening participation | Cross-sectional prospective study | Journal article | UK | UK Biobank cohort | National survey | 2006-2010 | Demographics, Economic, Patient access, Patient behavior, Social | No |
| Nuche-Berenguer 2021 (623) | Breast cancer, Cervical cancer, Colorectal cancer | The aim of this study was to investigate the socioeconomic determinants underlying disparities in the utilization of cancer screening services in Argentina and to measure the country progress in reducing such disparities across socioeconomic levels. | Cross-sectional study | Journal article | Argentina | National Survey of Risk Factors of Argentina. | Nationwide Population-based study | 2018 | Demographics, Economic, Patient access, Social | No |
| Nuño 2012 (624) | Breast cancer, Cervical cancer | To compare breast and cervical cancer screening utilization among Hispanic and AI women that reside in rural areas of the Southwestern United States to their urban counterparts and to assess characteristics that influence cancer screening | Cohort study | Journal article | USA | Behavioral Risk Factor Surveillance System (BRFSS) | Community based survey | 2006 and 2008 | Demographics, Economic, Patient access, PCP access, Social | No |
| Oladeru 2022 (625) | Breast cancer, Cervical cancer | To examine the difference in self-reported breast and cervical cancer screening rates between TG and cisgender (CG) people | Cross-sectional study | Journal article | USA | Behavioral Risk Factor Surveillance System (BRFSS) | Nationwide study | 2014 to 2016 and 2018 | Demographics | Yes |
| Pang 2017 (626) | Breast cancer, Cervical cancer, Colorectal cancer | To evaluate the association between having a regular source of healthcare and adherence to recommended cancer screenings in the Little Haiti population of Miami. | Cross-sectional study | Journal article | USA | This secondary analysis utilized data collected from a random-sample, population-based household survey | City wide population based | Nov 2011- Dec 2012 | Demographics, Economic, Patient access, Patient behavior, Social | No |
| Park 2017 (627) | Colorectal cancer, Gastric cancer | The aim of our survey was to determine the patterns of screening for colorectal and stomach cancer screening and related factors in lung cancer survivors who were disease free in Korea. | Cross-sectional study | Journal article | Korea | NR | Multicenter | 2007 | Economic, Patient behavior, PCP access, Social | No |
| Peltzer 2014 (628) | Breast cancer, Cervical cancer | The study the authors collected information on screening for two major types of cancers: cervical and breast cancer in order to establish their prevalence estimates and correlates among older South African women who participated in the Study of Global Ageing and Adults Health (SAGE) in 2008 | Population based cross-sectional study | Journal article | South Africa | The Study of Global Ageing and Adults Health (SAGE) | National survey | 2008 | Demographics, Economic, Patient access, Social | No |
| Pengpid 2021 (629) | Breast cancer, Cervical cancer, Colorectal cancer | To estimate the prevalence and associated factors of cancer screening among men and women in the general population in Marshall Islands. | Cross sectional study | Journal article | Marshall Islands | NR | National survey | 2017/2018 | Demographics, Economic, Patient access, Patient behavior, PCP access, Social | Yes |
| Phaswana-Mafuya 2018 (630) | Breast cancer, Cervical cancer | To estimate the prevalence of breast and cervical cancer screening among women in the South African general population and assess associated factors. | National population based cross-sectional household survey | Journal article | South Africa | 2012 South African national HIV prevalence, incidence, and behavior cross-sectional survey | National survey | 2012 | Demographics, Economic, Patient access, Social | No |
| Ponce 2012 (631) | Breast cancer, Colorectal cancer | To evaluate whether breast cancer (BC) and colorectal cancer (CRC) disparities varied by family history risk using a large, multiethnic population-based survey. | Cross-sectional study | Journal article | USA | California Health Interview Survey | Statewide study | 2005 | Demographics, Economic, Patient access, PCP access, Social | No |
| Ricardo-Rodrigues 2015a (632) | Breast cancer, Cervical cancer | To estimate the uptake of breast and cervical cancer screening using data from the 2011 Spanish national health survey, to compare uptake rates with those obtained in previous Spanish national health surveys, and to identify health and lifestyle-related sociodemographic variables that are predictive of screening uptake | Cross-sectional study | Journal article | Spain | 2011 Spanish national health survey | National survey | 2011 | Demographics, Economic, Patient access, Patient behavior, Social | No |
| Riza 2017 (633) | Breast cancer, Cervical cancer | To investigate the socio-economic features of women participating in secondary prevention of breast and cervical cancer in an austerity setting. | Cross-sectional study | Journal article | Greece | NR | Single center | 5 months in 2015 (Aug to Dec) | Economic, Patient access, Social | No |
| Rondet 2014 (634) | Breast cancer, Cervical cancer | To compare breast cancer screening (BCS) and cervical cancer screening (CCS) practices of French women born to French parents with those of immigrants and nationals born to immigrants, taking their socioeconomic status into account. | Cross-sectional study | Journal article | France | SIRS (French acronym for Health, Inequalities and Social Ruptures) survey | City wide study | 2010 | Demographics, Economic, Social | No |
| Saha 2021 (635) | Breast cancer, Cervical cancer | To assess screening practices and factors affecting screening for breast and cervical cancer, amongst women aged 25–60 years, in a rural block in Vellore, Tamil Nadu. | Cross-sectional study | Journal article | India | NR | Multicenter | 2018 | Economic, Patient access | No |
| Sahoo 2020 (636) | Breast cancer, Cervical cancer, Colorectal cancer, Prostate cancer | This study examined the association between problem-solving skills in the technology-rich environment (PSTRE) and cancer screening in later life. | Cross-sectional study | Journal article | USA | The 2012/2014 Program for the International Assessment of Adult Competencies data (PIAAC) | National survey | 2012/2014 | Demographics, Economic, Patient access, Patient behavior, Social | No |
| Sakellariou 2017 (637) | Breast cancer, Cervical cancer | The aim of the study was to examine the utilization rates of the Pap test and the mammogram for women with and without disability in Chile, and the factors influencing utilization for disabled women. | Cross-sectional study | Journal article | Chile | The 2015 National Socioeconomic Characterization Survey (Encuesta Nacional de Characterization Socioeconomical–CASEN), conducted by the Ministry of Social Development of the Government of Chile | National survey | 2006-2015 | Demographics, Economic, Patient access, Patient behavior, Social | No |
| Sentell 2015 (638) | Breast cancer, Cervical cancer, Colorectal cancer | To examine the relationship between LHL and meeting US Preventive Service Task Force (USPSTF) guidelines for cervical, colorectal and breast cancer screening among Chinese Americans. | Cross-sectional study | Journal article | USA | California Health Interview Survey (CHIS) | Statewide population based | 2007 | Demographics, Economic, Patient access, PCP access, Social | No |
| Shete 2021 (639) | Breast cancer, Colorectal cancer | To compare the prevalence of breast and colorectal cancer screening adherence and to identify factors associated with screening adherence among women residing in rural vs urban areas in the United States | Cross-sectional study | Journal article | USA | NR | Nationwide study | 2017-2020 | Demographics, Economic, Patient access, Patient behavior, Social | No |
| Sicsic 2014 (640) | Breast cancer, Cervical cancer, Colorectal cancer | To analyze the obstacles to and levers for breast, cervical, and colorectal cancer screening uptake and their trends over time. | Cross-sectional study | Journal article | France | French Health Care and Health Insurance Survey (three independent, cross-sectional surveys: 2006, 2008, and 2010) | Nationwide study | 2006,2008,2010 | Demographics, Economic, Patient access, Patient behavior, Social | No |
| Subba 2022 (641) | Breast cancer, Cervical cancer | This study aimed to assess the knowledge and utilization of screening methods for cervical and breast cancer and to elaborate barriers for non-utilization amongst female HCPs, both doctors and nurses | Cross-sectional study | Journal article | India | NR | City-wide survey | April to July 2017 | Demographics, Economic, Patient behavior | No |
| Takeuchi 2020 (642) | Breast cancer, Colorectal cancer, Prostate cancer | The objective of the current study was to investigate the extent to which caregivers’ FCR predicted their cancer screening behaviors years after their relative’s initial cancer diagnosis. We first investigated the linear relations of FCR with cancer screening behaviors for colorectal, breast, and prostate cancers. We hypothesized that greater FCR would be associated with 1) a greater likelihood of undergoing cancer screening (uptake); and 2) among those undergoing a screening, repeating screenings at recommended intervals (maintenance). Second, we explored curvilinear relations (inverse “U” shape), hypothesizing that moderate levels of FCR would be associated with greater likelihoods of cancer screening uptake and maintenance, whereas low and high levels of FCR would be associated with lower likelihoods of screening uptake and maintenance. We further explored the extent to which screening behaviors were age-appropriate as per clinical guidelines, and whether FCR was associated with age-(in) appropriate screening. | Retrospective cohort study | Journal article | USA | National Quality of Life Survey for Caregivers | National based survey | NR | Demographics, Economic, Patient behavior, Social | No |
| Telvizian 2021 (643) | Breast cancer, Cervical cancer, Colorectal cancer, Multiple cancers, Skin cancer | To investigate the knowledge, beliefs, and practices related to the prevention and screening for breast, cervical, colon, lung, and skin cancers among Lebanese residents, recruited through social media advertisements and community outreach activities. | Cross-sectional study | Journal article | Lebanon | Participants filled an anonymous questionnaire either via a web-based interface or using tablets distributed at primary health clinics | Nation based study | An online self-administered questionnaire was open to the public for 42 days starting on 4 February 2019. For the community sample, members of the research team visited clinics between August and October 2019. | Demographics, Economic, Patient access, Patient behavior, PCP access, Social | No |
| Theodoropoulos 2022 (644) | Breast cancer, Colorectal cancer | To examine the trends and correlates of breast and colorectal cancer screening among females aged 50-7 | Cross-sectional study | Conference abstract | USA | Behavioral Risk Factor Surveillance System (BRFSS) data available between 2014-2019 | Nationwide study | 2014-2019 | Demographics, Economic | No |
| Towne 2014 (645) | Breast cancer, Colorectal cancer | To identify potential disparities in the availability of screening services, distance to care, and the utilization of cancer screening services for Medicare beneficiaries residing in areas with a higher concentration of AIAN individuals aged 65 and older | Cross-sectional study | Journal article | USA | Research Triangle Institute (RTI) International’s Spatial Impact Factor Data (2012) | Community based survey | 2012 | Patient access, PCP access | No |
| Tracy 2013 (646) | Cervical cancer, Colorectal cancer | This study was to examine cervical cancer screening behaviors in a national sample of lesbians | Cross-sectional study | Journal article | USA | NR | National survey | February-June 2010. | Demographics, Patient access, Patient behavior, PCP access | No |
| Trinh 2016 (647) | Breast cancer, Cervical cancer, Colorectal cancer, Prostate cancer | To determine whether AsAs are under-utilizing recommended cancer screening | Cross-sectional study | Journal article | USA | BRFSS survey | National survey | 2012 | Demographics, Economic, Patient access, PCP access, Social | No |
| Valdovinos 2016 (648) | Breast cancer, Cervical cancer, Colorectal cancer | This study examined whether perceived discrimination was associated with adherence to breast, cervical, colorectal, and prostate cancer screening guidelines in US Hispanic/ Latino adults. | Retrospective study | Journal article | USA | Hispanic Community Health Study/Study of Latinos Sociocultural Ancillary Study | Community wide study | 2008 and 2011 | Demographics, Patient access, Patient behavior | No |
| Van Manh 2020 (649) | Breast cancer, Cervical cancer, Colorectal cancer | This study examined the potential disparities for cancer screening as measured by accessing CL, MM, and PS among a sample of immigrants living in NYC | Cross-sectional study | Journal article | USA | Hepatitis Outreach Network (HONE) | Community wide survey | 2013-2015 | Demographics | No |
| Von Wagner 2013 (650) | Colorectal cancer, Multiple cancers | This study examined attitudes to continuing cancer screening among men and women aged 60 and over. Our objectives were to examine: (i) attitudes towards age-based stoppage policies, (ii) preference for communication about screening options after the end of the call–recall program and (iii) intentions to opt-in to screening after the final invitation | Cross-sectional study | Journal article | UK | TNS Research International survey | National population-based survey | July, 2011 | Demographics, Patient access, Social | No |
| Vrinten 2019 (651) | Breast cancer, Cervical cancer, Colorectal cancer | To quantify the prevalence and socio-demographic patterning of cancer stigma in the general population and to explore its association with cancer screening attendance | Cross-sectional study | Journal article | UK | Attitudes, behavior and Cancer UK Survey (ABACUS) in 2016 | National survey | 2016 | Patient behavior | No |
| Weisband 2021 (652) | Breast cancer, Cervical cancer, Colorectal cancer | To examine whether breast, colorectal, and cervical cancer screening rates in women differed by SES and age, and whether screening rates and SES disparities changed after introduction of a primary care–based national quality indicator program | Cross-sectional study | Journal article | Israel | Israel’s 4 health maintenance organizations (HMOs) using data originating from electronic health records, for the entire Israeli population | National survey | 2002-2017 | Demographics, Economic | No |
| Willems 2020 (653) | Breast cancer, Cervical cancer | To examine whether the extent of macro-level gender inequality affects the association between women’s educational attainment and their participation in cervical and breast cancer screening and how this relationship is moderated by a country’s cancer screening strategy (organized vs. opportunistic) | Cross sectional study | Journal article | Europe | European Health Interview Survey | International survey | 2013-2015 | Patient access, Social | No |
| Xiang 2015 (654) | Breast cancer, Cervical cancer | To examine the association of serious psychological distress (SPD) and cancer-screening utilization in a nationally representative sample of women aged 40 to 74 years and to identify barriers and facilitating factors to breast and cervical cancer screening among women with SPD | Cross-sectional study | Journal article | USA | Household Component of Medical Expenditure Panel Survey (MEPS-HC), a large-scale, nationally representative survey of health services and expenditures for the U. S | Nationwide study | 2007, 2009, and 2011. | Demographics, Economic, Patient access, Patient behavior, PCP access, Social | No |
| Xie 2022 (655) | Breast cancer, Cervical cancer, Colorectal cancer | To examine associations of LEP and patient-provider language concordance (PPLC) with evidence-based cancer screening utilization for cervical, breast, and colorectal cancers among Asian American adults. | Cross-sectional study | Journal article | USA | 2010–2016 and 2018 Medical Expenditure Panel Survey | Nationwide study | 2010–2016, 2018 | Demographics, Economic, Patient access, Patient behavior, PCP access, Social | No |
| Yamashita 2020 (656) | Breast cancer, Cervical cancer, Colorectal cancer | To investigate the associations between multimorbidity and the delivery of breast, cervical, and colorectal cancer screening in Japan, and to identify subgroups that did not receive cancer screening | Cross-sectional study | Journal article | Japan | Comprehensive Survey of Living Conditions | National survey | 2016 | Demographics, Economic, Social | No |

Abbreviations: ABACUS: BC: breast cancer; BRFSS: Behavioral Risk Factor Surveillance System; BSE: Breast self examination; CASEN: National Socioeconomic Characterization Survey (Nacional de Characterization Socioeconomical); CBE: Clinical breast exam; CC: Cervical cancer; CDC: Centre for Disease Control and Prevention; CHIS: California Health Interview Survey; CG: Cis-gender; CHIP: Cancer Health Impact Program; CIC: Citizenship and Immigration Canada; CL: Colonocopy; CRC: Colorectal cancer; CSLC: Comprehensive Survey of Living Conditions; DM: Diabetes mellitus; ED: Emergency department; HC: health centre; FCR: Fear of cancer recurrence; FI: Frailty index; FOBT: Faecal occult blood test; HCP: Healthcare provider; HINTS: Health Information National Trends Survey; HMO: health maintenance organisation; HONE: Hepatitis Outreach Network; HSCIC: Health & Social Care Information Centre; HONE: ; HWS: Health and Welfare Survey; IMG: International Medical Graduate; KNHANES: Korea National Health and Nutrition Examination Survey; LEP: Limited English proficiency; LHL: Low health literacy; MEPS: Medical Expenditure Panel Survey; MM: mammogram; NHIS: National Health Interview Survey; NLST: National Lung Screening Trial; NR: not reported; NYC: New York City; OS: Oncobarometro Survey; PASSI: Progressi delle Aziende Sanitarie per la Salute in Italia (Italian public health surveillance system); PCP: Primary care provider; PD: Psychological distress; PPLC: patient-provider language concordance; PS: Papanicolaou smear; PSA: Prostate-specific antigen; RHS: Reproductive Health Survey; RTI: Research Triangle Institute; SAGE: Study on Global Ageing and Adult Health; SDOH: Social determinants of health; SES: Socio-economic status; SO: Sexual orientation; SOGI: Sexual orientation and gender identity; SPD: Serious psychological distress; SVI: social vulnerability index; TG: Transgender; UK: United Kingdom; US: United States; USA: United States of America; USPSTF: US Preventive Service Task Force; WHI: Women’s health initiative; WHO: World Health Organisation; WHS: World Health Survey;

# **Appendix S6. Summary of statistically significant ORs for gastric and prostate cancer for selected factors**

**Figure 2. Summary of odds ratios according to tumor type for selected demographic characteristics in prostate and gastric cancers.**

**
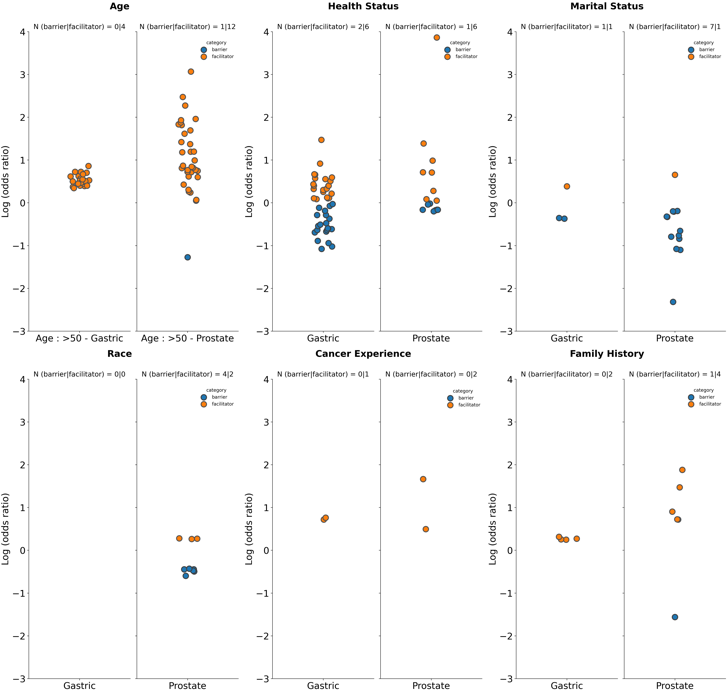
**

**(A) Age**: Datapoints represent log(ORs) for groups aged ≥50 years (vs. <50 years as reference group in studies on breast and lung cancer screening programs), ≥30 years (vs. < 30 years as reference groups for studies on screening for cervical cancer), ≥60 years (vs. <60 years as reference group in studies on screening for CRC); **(B) Health status**: Datapoints represent log(ORs) for groups with perception of health rated as “poor”, or “fair” (vs. ratings of “good”, “very good", and “normal” as reference group across included studies); **(C) Marital status**: Datapoints represents log(ORs) for “single”, “divorced”, “widowed” “or “separated” groups (vs. those who were “married” or “partnered” as reference groups); **(D) Race**: Datapoints represent log(ORs) for non-white race groups (vs. white race groups as reference group)**; (E) Cancer experience**: Datapoints represents log(ORs) for groups with any previous cancer history (vs. those with no cancer history as a reference group across included studies); **(F) Family history**: Datapoints represents log(ORs) for groups with family history of cancer (vs. those with no history of cancer as a reference group). The number of studies for which a specific factor acted as a barrier or a facilitator is shown as “N” atop each panel. A single study may be categorized as both a barrier and a facilitator if different strata within it showed opposing associations with screening uptake for the same factor. Facilitator (orange): a factor with a log(OR) >0, indicating a higher screening uptake for the comparison group vs the reference group. Barrier (blue): a factor with a log(OR) <0, indicating a lower screening uptake for the comparison group vs the reference group

**Figure 3. Summary of odds ratios according to tumor type for selected economic characteristics in prostate and gastric cancers.**

**
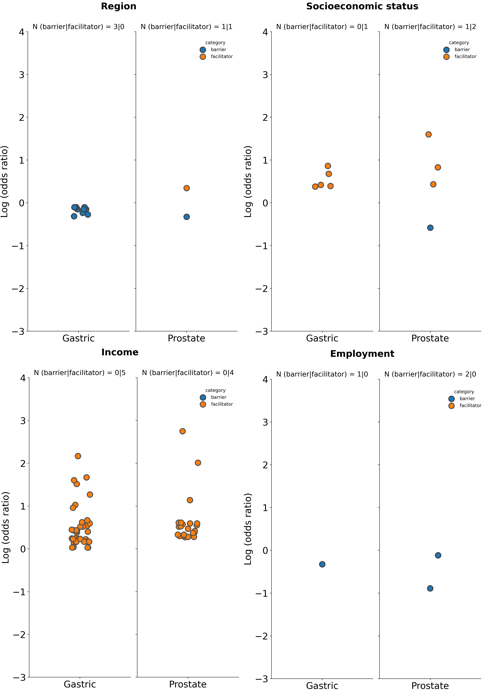
**

**(A) Region**: Datapoints represent log(ORs) for urban or suburban dwelling groups (vs. “inaccessible areas”, “rural”, or "non-metropolitan” as reference); **(B) Socioeconomic status**: Datapoints represent log(ORs) for richer or wealthier groups (vs. “poorest “, “poor, or “lower class” as reference); **(C) Income**: Datapoints represent log(Ors) for higher income groups (vs. lower income groups as reference); **(D) Employment**: Datapoints represent log(ORs) for employed individuals/groups (vs. “unemployed", “retired”, “homemaker”, “housewife”, or “student” groups as reference). Employed included “self-employed”, “part-time and full-time employment”, “contracted”. The number of studies for which a specific factor acted as a barrier (in blue) or a facilitator (in orange) is shown as “N” atop each panel. A single study may be categorized as both a barrier and a facilitator if different strata within it showed opposing associations with screening uptake for the same factor. Facilitator (orange): a factor with a log(OR) >0, indicating a higher screening uptake for the comparison group vs the reference group. Barrier (blue): a factor with a log(OR) <0, indicating a lower screening uptake for the comparison group vs the reference group.

.

**Figure 4. Summary of odds ratios according to tumor type for selected patient access characteristics in prostate and gastric cancers**

**
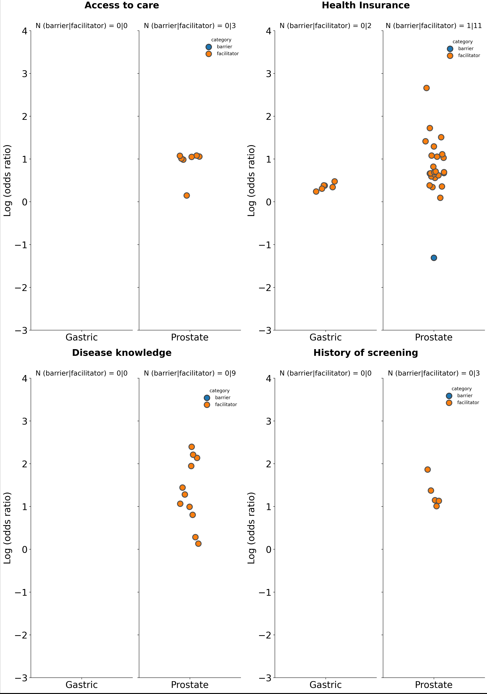
**

**(A) Access to care:** Datapoints represent log(ORs) for groups with access to care or a PCP (vs. those with “no access to care”, “no PCP", or “health access hardship” as reference); **(B) Health insurance coverage:** Datapoints represent log(ORs) for groups with any form of health insurance coverage (vs. uninsured groups as reference); **(C) Disease knowledge:** Datapoints represent log(ORs) for groups with “high”, “good’, or “sufficient” knowledge about the disease (vs. groups with “low understanding", “not knowledgeable”, “poor knowledge”, or “insufficient” knowledge as reference); **(D) History of screening:** Datapoints represent log(ORs) for groups with a history of cancer screening (vs. those with no history of screening as reference). The number of studies for which a specific factor acted as a barrier (in blue) or a facilitator (in orange) is shown as “N” atop each panel. A single study may be categorized as both a barrier and a facilitator if different strata within it showed opposing associations with screening uptake for the same factor. Facilitator (orange): a factor with a log(OR) >0, indicating a higher screening uptake for the comparison group vs the reference group. Barrier (blue): a factor with a log(OR) <0, indicating a lower screening uptake for the comparison group vs the reference group.

**Figure 5. Summary of odds ratios according to tumor type for selected patient behavior characteristics in prostate and gastric cancers**


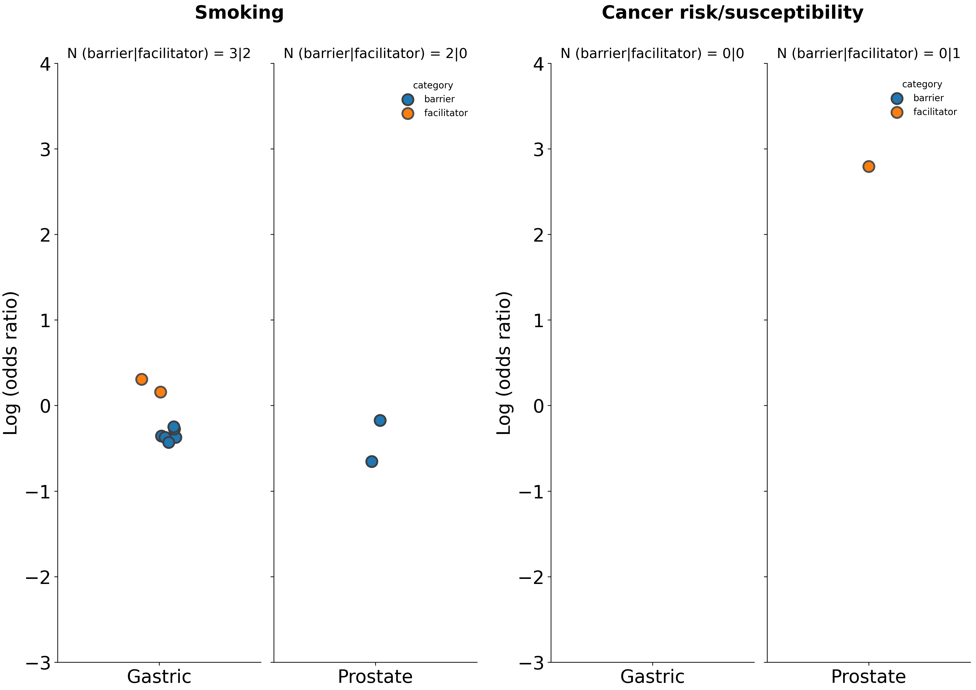


**(A) Smoking status**: Datapoints represent log(ORs) for groups of “current” smokers (vs. groups of “former”, “previous”, or “never” smokers as reference); **(B) Cancer risk:** Datapoints represent log(ORs) for groups with “higher”, “moderate” perceived cancer susceptibility (vs. those with “no” or “lower” perceived susceptibility to cancer as reference). The number of studies for which a specific factor acted as a barrier (in blue) or a facilitator (in orange) is shown as “N” atop each panel. A single study may be categorized as both a barrier and a facilitator if different strata within it showed opposing associations with screening uptake for the same factor. Facilitator (orange): a factor with a log(OR) >0, indicating a higher screening uptake for the comparison group vs the reference group. Barrier (blue): a factor with a log(OR) <0, indicating a lower screening uptake for the comparison group vs the reference group.

**Figure 6. Summary of odds ratios according to tumor type for selected PCP access characteristics in prostate and gastric cancers**

**
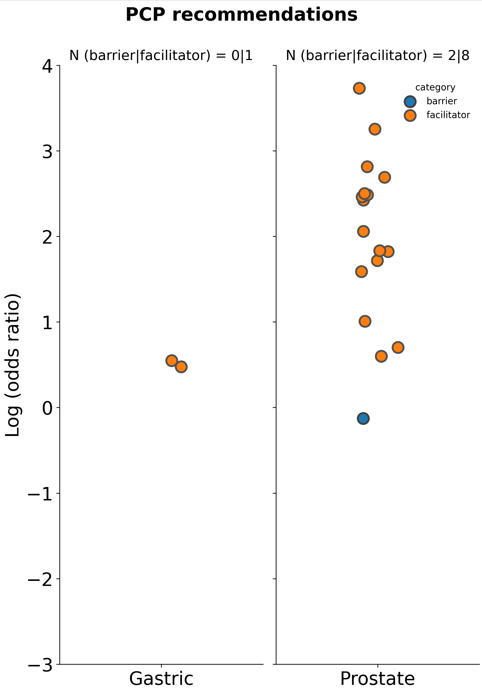
**

**PCP recommendations:** Datapoints represent log(ORs) for groups who received a cancer screening recommendation by a PCP (vs. those who did not receive such a recommendation from a PCP as reference). The number of studies for which a specific factor acted as a barrier (in blue) or a facilitator (in orange) is shown as “N” atop each panel. A single study may be categorized as both a barrier and a facilitator if different strata within it showed opposing associations with screening uptake for the same factor. Facilitator (orange: a factor with a log(OR) >0, indicating a higher screening uptake for the comparison group vs the reference group. Barrier (blue): a factor with a log(OR) <0, indicating a lower screening uptake for the comparison group vs the reference group.

**Figure 7. Summary of odds ratios according to tumor type for selected social characteristics in prostate and gastric cancers**

**
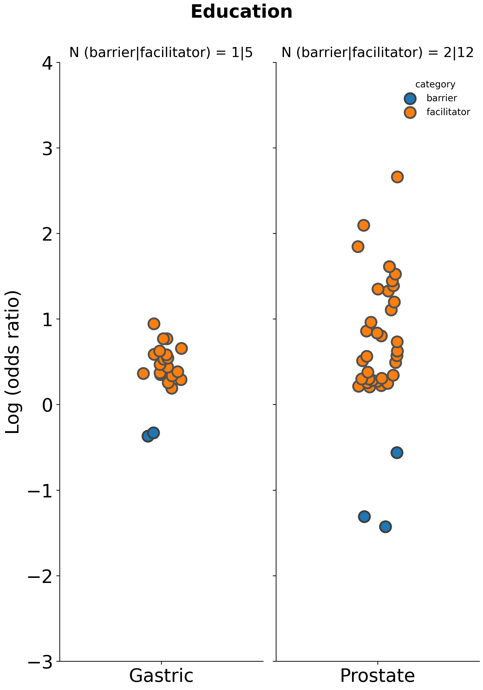
**

**Education:** Datapoints represents log(ORs) for “high education”, “≥ high school graduate”, “tertiary education”, “university/college graduate” groups (vs. groups with “low education", “no school”, “no formal education”, “illiterate”, “<high school”, “did not graduate high school”, “primary school”, or “middle school” as reference). The number of studies for which a specific factor acted as a barrier (in blue) or a facilitator (in orange) is shown as “N” atop each panel. A single study may be categorized as both a barrier and a facilitator if different strata within it showed opposing associations with screening uptake for the same factor. Facilitator (orange): a factor with a log(OR) >0, indicating a higher screening uptake for the comparison group vs the reference group. Barrier (blue): a factor with a log(OR) <0, indicating a lower screening uptake for the comparison group vs the reference group

# **Appendix S7. Quality assessment**

**Figure 8. Quality assessment for overall studies included in the analysis according to tumor type.**

**Table 6. Quality assessment of the studies reporting results for breast cancer (n=199)**

| **Study name** | **Total score** | **Interpretation** |
| --- | --- | --- |
| Abdel-Aziz 2018 (1) | 5 | Medium quality |
| Ahmadian 2012 (2) | 5 | Medium quality |
| Akinyemiju 2012 (547) | 7 | High quality |
| Akinyemiju 2016 (548) | 4 | Medium quality |
| Al Rifai 2015 (549) | 7 | High quality |
| Alatrash 2021 (3) | 5 | Medium quality |
| Al-Azri 2020 (4) | 4 | Medium quality |
| Al-Hanawi 2020 (5) | 7 | High quality |
| Anwar 2018 (550) | 5 | Medium quality |
| Aschwanden 2019 (551) | 4 | Medium quality |
| Asgary 2014 (6) | 4 | Medium quality |
| Assefa 2021 (7) | 7 | High quality |
| Azhar 2022 (552) | 5 | Medium quality |
| Balas 2020 (8) | 5 | Medium quality |
| Bao 2018a (9) | 5 | Medium quality |
| Baughman 2016 (10) | 4 | Medium quality |
| Bawazir 2019 (11) | 6 | Medium quality |
| Bcheraoui 2015 (12) | 7 | High quality |
| Beaber 2019 (13) | 6 | Medium quality |
| Berens 2014 (14) | 3 | Low quality |
| Bertaut 2018 (554) | 5 | Medium quality |
| Beyer 2016 (555) | 6 | Medium quality |
| Bhandari 2021 (15) | 5 | Medium quality |
| Bringedal 2019 (556) | 7 | High quality |
| Bussière 2014 (557) | 7 | High quality |
| Carrasco-Garrido 2014 (559) | 5 | Medium quality |
| Carrozzi 2015 (560) | 6 | Medium quality |
| Cataneo 2020 (16) | 4 | Medium quality |
| Chan 2014 (17) | 6 | Medium quality |
| Charkhchi 2019 (561) | 7 | High quality |
| Choi 2015 (18) | 5 | Medium quality |
| Choi 2017 (19) | 4 | Medium quality |
| Constantinou 2016 (562) | 7 | High quality |
| Dahlui 2012 (20) | 4 | Medium quality |
| Dallo 2015 (563) | 3 | Low quality |
| Damiani 2012 (564) | 6 | Medium quality |
| Dawidowicz 2020 (565) | 5 | Medium quality |
| Deshpande 2012 (566) | 6 | Medium quality |
| Documet 2015 (567) | 7 | High quality |
| Donley 2020 (568) | 6 | Medium quality |
| Donnelly 2012 (21) | 5 | Medium quality |
| Donnelly 2013 (22) | 5 | Medium quality |
| Donnelly 2015 (23) | 6 | Medium quality |
| Duport 2012 (24) | 7 | High quality |
| Eichholzer 2016 (25) | 6 | Medium quality |
| El Mhamdi 2013 (26) | 5 | Medium quality |
| Elobaid 2014 (27) | 4 | Medium quality |
| Eng 2020 (569) | 5 | Medium quality |
| Ezema 2021 (28) | 7 | High quality |
| Farid 2014 (29) | 5 | Medium quality |
| Farzaneh 2017 (570) | 5 | Medium quality |
| Fayanju 2014 (30) | 3 | Low quality |
| Fleming 2013 (31) | 5 | Medium quality |
| Gan 2018 (32) | 5 | Medium quality |
| Gang 2013 (33) | 6 | Medium quality |
| Gilfoyle 2019 (34) | 7 | High quality |
| Gong 2023 (35) | 4 | Medium quality |
| Guilcher 2014 (36) | 5 | Medium quality |
| Guo 2021b (574) | 6 | Medium quality |
| Haas 2016 (576) | 7 | High quality |
| Hajian-Tilaki 2014 (37) | 7 | High quality |
| Hajian-Tilaki 2015 (38) | 4 | Medium quality |
| Hanske 2016 (577) | 6 | Medium quality |
| Harcourt 2014 (578) | 5 | Medium quality |
| Hasnain 2014 (40) | 7 | High quality |
| Hassan 2017 (39) | 4 | Medium quality |
| Hippman 2016 (41) | 5 | Medium quality |
| Horner-Johnson 2014a (581) | 6 | Medium quality |
| Horner-Johnson 2015 (582) | 6 | Medium quality |
| Hsieh 2021 (42) | 6 | Medium quality |
| Huang 2021b (583) | 7 | High quality |
| Ishii 2021 (584) | 6 | Medium quality |
| Jacobs 2014 (585) | 7 | High quality |
| Jadav 2015 (43) | 6 | Medium quality |
| Jin 2019c (44) | 4 | Medium quality |
| Jin 2021 (45) | 4 | Medium quality |
| Jolidon 2021 (586) | 6 | Medium quality |
| Kadaoui 2012 (46) | 5 | Medium quality |
| Kang 2014 (588) | 7 | High quality |
| Kangmennaang 2019 (47) | 5 | Medium quality |
| Karadeniz 2019 (589) | 4 | Medium quality |
| Kardan-Souraki 2019 (48) | 4 | Medium quality |
| Kelly 2017 (590) | 6 | Medium quality |
| Khaliq 2015 (49) | 6 | Medium quality |
| Kim 2014 (50) | 7 | High quality |
| Kim 2018 (591)* | 4 | Medium quality |
| Kim 2019a (592) | 6 | Medium quality |
| Kim 2022 (51) | 7 | High quality |
| Kindratt 2020 (594) | 6 | Medium quality |
| Kirag 2019 (52) | 4 | Medium quality |
| Kiran 2019 (595) | 6 | Medium quality |
| Kırca 2018 (53) | 4 | Medium quality |
| Kiyang 2015 (54) | 4 | Medium quality |
| Kosog 2020 (55) | 4 | Medium quality |
| Kriaucioniene 2019 (56) | 5 | Medium quality |
| Kurani 2020 (596) | 5 | Medium quality |
| Kushalnagar 2019 (597) | 7 | High quality |
| Kwok 2014 (57) | 4 | Medium quality |
| Kwok 2016 (58) | 7 | High quality |
| Kwok 2022 (59) | 5 | Medium quality |
| Lam 2018 (60) | 4 | Medium quality |
| Lawson 2021 (61) | 4 | Medium quality |
| Lee 2017 (62) | 5 | Medium quality |
| Lee 2018c (63) | 5 | Medium quality |
| Lee 2020 (598) | 6 | Medium quality |
| Lee 2021 (599) | 6 | Medium quality |
| Lemogne 2018 (64) | 4 | Medium quality |
| Leung 2014 (65) | 5 | Medium quality |
| Llaneza 2022 (66) | 6 | Medium quality |
| Lo 2013 (600) | 7 | High quality |
| Lofters 2015 (601) | 6 | Medium quality |
| Lofters 2019 (602) | 8 | High quality |
| Lopez 2020 (67) | 5 | Medium quality |
| Ma 2012 (68) | 6 | Medium quality |
| MacDonald 2022 (603) | 7 | High quality |
| Malhotra 2017 (604) | 6 | Medium quality |
| Martinez-Huedo 2012 (605) | 7 | High quality |
| Martín-López 2013 (69) | 5 | Medium quality |
| Massat 2015 (606) | 5 | Medium quality |
| Mehta 2022 (607) | 3 | Low quality |
| Mendoza 2022 (608) | 7 | High quality |
| Menvielle 2014 (609) | 4 | Medium quality |
| Menvielle 2018 (610) | 7 | High quality |
| Milenkov 2020 (611) | 5 | Medium quality |
| Miles 2019 (70) | 6 | Medium quality |
| Miles 2021 (612) | 5 | Medium quality |
| Mo 2014 (613) | 6 | Medium quality |
| Mohan 2021 (71) | 6 | Medium quality |
| Moreno 2019 (614) | 7 | High quality |
| Morere 2019 (72) | 5 | Medium quality |
| Moss 2019 (615) | 6 | Medium quality |
| Mukem 2014 (73) | 7 | High quality |
| Mukem 2015 (617) | 7 | High quality |
| Murphy 2021 (618) | 6 | Medium quality |
| Nandam 2018 (74) | 6 | Medium quality |
| Ngan 2022 (75) | 7 | High quality |
| Nicholls 2017 (620) | 6 | Medium quality |
| Niedzwiedz 2020 (622) | 5 | Medium quality |
| Nojomi 2014 (76) | 6 | Medium quality |
| Nuche-Berenguer 2021 (623) | 4 | Medium quality |
| Nuño 2012 (624) | 5 | Medium quality |
| O’Hara 2018 (77) | 6 | Medium quality |
| Ogunsiji 2017 (78) | 4 | Medium quality |
| Okui 2021 (79) | 7 | High quality |
| Oladeru 2022 (625) | 6 | Medium quality |
| Pagán 2012 (80) | 4 | Medium quality |
| Pang 2017 (626) | 6 | Medium quality |
| Paranjpe 2022 (81) | 5 | Medium quality |
| Park 2020 (82) | 6 | Medium quality |
| Patel 2014 (83) | 7 | High quality |
| Peltzer 2014 (628) | 6 | Medium quality |
| Pengpid 2021 (629) | 7 | High quality |
| Phaswana-Mafuya 2018 (630) | 6 | Medium quality |
| Ponce 2012 (631) | 5 | Medium quality |
| Racine 2022 (84) | 5 | Medium quality |
| Radhakrishnan 2018 (85) | 6 | Medium quality |
| Ricardo-Rodrigues 2015a (632) | 7 | High quality |
| Riza 2017 (633) | 4 | Medium quality |
| Rollet 2021 (86) | 3 | Low quality |
| Rondet 2013 (87) | 6 | Medium quality |
| Rondet 2014 (634) | 6 | Medium quality |
| Ross 2020 (88) | 7 | High quality |
| Ross 2022 (89) | 5 | Medium quality |
| Sabgul 2021 (90) | 5 | Medium quality |
| Saha 2021 (635) | 5 | Medium quality |
| Sakellariou 2017 (637) | 7 | High quality |
| Satoh 2021 (91) | 4 | Medium quality |
| Sentell 2015 (638) | 6 | Medium quality |
| Sheppard 2015 (92) | 6 | Medium quality |
| Shete 2021 (639) | 5 | Medium quality |
| Shin 2020a (93) | 3 | Low quality |
| Sicsic 2014 (640) | 5 | Medium quality |
| Solikhah 2018 (94) | 5 | Medium quality |
| Solikhah, 2019 (95) | 5 | Medium quality |
| Son 2017 (96) | 5 | Medium quality |
| Subba 2022 (641) | 5 | Medium quality |
| Subramanian 2013 (97) | 4 | Medium quality |
| Takeuchi 2020 (642) | 5 | Medium quality |
| Tapera 2019a (98) | 5 | Medium quality |
| Telvizian 2021 (643) | 5 | Medium quality |
| Theodoropoulos 2022 (644) | 6 | Medium quality |
| Towne 2014 (645) | 5 | Medium quality |
| Tran 2021 (99) | 7 | High quality |
| Trinh 2016 (577) | 4 | Medium quality |
|  |  |  |
| Tsunematsu 2013 (100) | 4 | Medium quality |
| Valdovinos 2016 (648) | 7 | High quality |
| Van Manh 2020 (649) | 4 | Medium quality |
| Vang 2022 (101) | 5 | Medium quality |
| Vrinten 2019 (651) | 7 | High quality |
| Wang 2022 (102) | 4 | Medium quality |
| Warner 2019 (103) | 6 | Medium quality |
| Weisband 2021 (652) | 7 | High quality |
| Willems 2020 (653) | 6 | Medium quality |
| Wilson 2014 (104) | 6 | Medium quality |
| Xiang 2015 (654) | 7 | High quality |
| Xie 2022 (655) | 6 | Medium quality |
| Yamashita 2020 (656) | 6 | Medium quality |
| Yilmaz 2013 (105) | 4 | Medium quality |
| Zhang 2012 (106) | 4 | Medium quality |

**Table 7. Quality assessment of the studies reporting results for cervical cancer (n=278)**

| **Study name** | **Total score** | **Interpretation** |
| --- | --- | --- |
| Abdullah 2016 (107) | 7 | High quality |
| Abebaw 2022 (108) | 5 | Medium quality |
| Abera 2020 (109) | 6 | Medium quality |
| Agboola 2021 (110) | 6 | Medium quality |
| Agénor 2014 (111) | 6 | Medium quality |
| Akinlotan 2017 (112) | 5 | Medium quality |
| Akinlotan 2018 (113) | 5 | Medium quality |
| Akinyemiju 2012 (547) | 7 | High quality |
| Akinyemiju 2016 (548) | 4 | Medium quality |
| Aktaş 2021 (114) | 5 | Medium quality |
| Al Rifai 2015 (549) | 7 | High quality |
| Al-Amro 2020 (115) | 4 | Medium quality |
| Amin 2020 (116) | 6 | Medium quality |
| Aminisani 2012 (117) | 8 | High quality |
| Aminisani 2016 (118) | 6 | Medium quality |
| Anwar 2018 (550) | 5 | Medium quality |
| Ararsa 2021 (119) | 5 | Medium quality |
| Aredo 2021 (120) | 6 | Medium quality |
| Arulogun 2012 (121) | 3 | Low quality |
| Aschwanden 2019 (551) | 4 | Medium quality |
| Assefa 2019 (122) | 5 | Medium quality |
| Atnafu 2021 (123) | 5 | Medium quality |
| Ayanore 2020 (124) | 5 | Medium quality |
| Aynalem 2020 (125) | 7 | High quality |
| Azhar 2022 (552) | 5 | Medium quality |
| Babazadeh 2018 (126) | 4 | Medium quality |
| Badre-Esfahani 2019 (127) | 7 | High quality |
| Badre-Esfahani 2020 (128) | 4 | Medium quality |
| Bao 2018b (129) | 5 | Medium quality |
| Barrett-Harrison 2018 (130) | 5 | Medium quality |
| Baruch 2022 (131) | 8 | High quality |
| Bayu 2016 (132) | 5 | Medium quality |
| Belay 2020 (133) | 5 | Medium quality |
| Bermedo-Carrasco 2015 (134) | 6 | Medium quality |
| Bianco 2017 (135) | 7 | High quality |
| Boni 2021 (136) | 5 | Medium quality |
| Bou-Orm 2018 (137) | 5 | Medium quality |
| Bringedal 2019 (556) | 7 | High quality |
| Brown 2016 (138) | 7 | High quality |
| Bruera 2020 (140) | 4 | Medium quality |
| Brzoska 2020 (141) | 7 | High quality |
| Budkaew 2014 (142) | 5 | Medium quality |
| Bussière 2014 (557) | 7 | High quality |
| Bussière 2015 (143) | 5 | Medium quality |
| Calys-Tagoe 2020 (144) | 5 | Medium quality |
| Carrasco-Garrido 2014 (559) | 5 | Medium quality |
| Carrozzi 2015 (560) | 6 | Medium quality |
| Cerigo 2013 (145) | 4 | Medium quality |
| Chan 2022b (146) | 6 | Medium quality |
| Chandrika 2020 (147) | 4 | Medium quality |
| Chaowawanit 2016 (148) | 7 | High quality |
| Charkhchi 2019 (561) | 7 | High quality |
| Chiou 2014 (149) | 5 | Medium quality |
| Chirwa 2022 (150) | 5 | Medium quality |
| Choi 2022 (318) | 6 | Medium quality |
| Cofie 2018 (152) | 7 | High quality |
| Compaore 2016 (153) | 5 | Medium quality |
| Cunningham 2015 (154) | 5 | Medium quality |
| Da Silva 2022 (155) | 7 | High quality |
| Dallo 2015 (563) | 3 | Low quality |
| Damiani 2012 (564) | 6 | Medium quality |
| Danan 2022 (156) | 4 | Medium quality |
| Dawidowicz 2020 (565) | 5 | Medium quality |
| De Vito 2014 (158) | 5 | Medium quality |
| Deguara 2021 (159) | 6 | Medium quality |
| Desta 2022 (160) | 7 | High quality |
| Destaw 2021 (161) | 5 | Medium quality |
| Documet 2015 (567) | 7 | High quality |
| Donley 2020 (568) | 6 | Medium quality |
| Drolet 2013 (163) | 4 | Medium quality |
| Dulla 2017 (164) | 5 | Medium quality |
| Ebu 2018b (166) | 4 | Medium quality |
| El Mhamdi 2012 (167) | 5 | Medium quality |
| Elit 2012 (168) | 5 | Medium quality |
| Elit 2013 (169) | 5 | Medium quality |
| Emmanuel 2016 (170) | 4 | Medium quality |
| Eng 2020 (569) | 5 | Medium quality |
| Enyan 2022 (171) | 5 | Medium quality |
| Erku 2017 (172) | 7 | High quality |
| Eshete 2020 (173) | 6 | Medium quality |
| Farzaneh 2017 (570) | 5 | Medium quality |
| Faye 2017 (174) | 5 | Medium quality |
| Fokom 2019 (175) | 5 | Medium quality |
| Ford 2021 (176) | 7 | High quality |
| Galvin 2021 (177) | 7 | High quality |
| Gan and Dahlui 2013 (178) | 5 | Medium quality |
| Gatumo 2018 (179) | 6 | Medium quality |
| Gawron 2021 (572) | 4 | Medium quality |
| Gebisa 2022 (180) | 6 | Medium quality |
| Gebreegziabher 2016 (181) | 6 | Medium quality |
| Gemeda 2020 (182) | 5 | Medium quality |
| Gerend 2017 (183) | 5 | Medium quality |
| Getachew 2019 (184) | 4 | Medium quality |
| Ghimire 2021 (185) | 4 | Medium quality |
| González 2015 (186) | 6 | Medium quality |
| González 2022 (187) | 7 | High quality |
| Goodwin 2020 (573) | 7 | High quality |
| Gottschlich 2019 (188) | 6 | Medium quality |
| Grillo 2012 (189) | 6 | Medium quality |
| Gu 2012 (190) | 7 | High quality |
| Gyulai 2015 (191) | 7 | High quality |
| Haas 2016 (576) | 7 | High quality |
| Haas 2021 (192) | 4 | Medium quality |
| Hanske 2016 (577) | 6 | Medium quality |
| Harcourt 2014 (578) | 5 | Medium quality |
| Harder 2018a (193) | 6 | Medium quality |
| Harder 2018b (194) | 6 | Medium quality |
| Harder 2020 (195) | 4 | Medium quality |
| Harper 2022 (196) | 7 | High quality |
| Holt 2021 (197) | 7 | High quality |
| Horner-Johnson 2015 (582) | 6 | Medium quality |
| Huang 2021b (583) | 7 | High quality |
| Idehen 2017 (198) | 5 | Medium quality |
| Idehen 2020 (199) | 5 | Medium quality |
| Idowu 2016b (200) | 6 | Medium quality |
| Isabirye 2020 (201) | 5 | Medium quality |
| Ishii 2021 (584) | 6 | Medium quality |
| Islam 2015 (202) | 5 | Medium quality |
| Issa 2021 (203) | 4 | Medium quality |
| Jabbari 2019 (204) | 5 | Medium quality |
| Jacobs 2014 (585) | 7 | High quality |
| Jang 2021 (205) | 4 | Medium quality |
| Johnson 2016 (206) | 5 | Medium quality |
| Jones 2021 (587) | 4 | Medium quality |
| Judah 2022 (207) | 5 | Medium quality |
| Kaneko 2018 (208) | 4 | Medium quality |
| Karadeniz 2019 (589) | 4 | Medium quality |
| Kasim 2020 (209) | 5 | Medium quality |
| Kaso 2019 (210) | 6 | Medium quality |
| Kasting 2017 (211) | 7 | High quality |
| Kelly 2017 (590) | 6 | Medium quality |
| Khanna 2019 (212) | 7 | High quality |
| Kileo 2015 (213) | 6 | Medium quality |
| Kim 2019a (592) | 6 | Medium quality |
| Kue 2017 (215) | 6 | Medium quality |
| Kurani 2020 (596) | 5 | Medium quality |
| Kushalnagar 2019 (597) | 7 | High quality |
| Lee 2012 (216) | 5 | Medium quality |
| Lee 2013b (537) | 6 | Medium quality |
| Lee 2020 (598) | 6 | Medium quality |
| Lee 2021 (599) | 6 | Medium quality |
| Leinonen 2017 (219) | 7 | High quality |
| Lemma 2022 (220) | 6 | Medium quality |
| Liang 2022 (221) | 5 | Medium quality |
| Lin 2021 (222) | 5 | Medium quality |
| Lin 2022 (223) | 4 | Medium quality |
| Liu 2017 (224) | 5 | Medium quality |
| Lo 2013 (600) | 7 | High quality |
| Lofters 2015 (601) | 6 | Medium quality |
| Lofters 2019 (602) | 8 | High quality |
| Lyimo 2012 (225) | 4 | Medium quality |
| Mabotja 2021 (226) | 6 | Medium quality |
| MacDonald 2022 (603) | 7 | High quality |
| Maharjan 2020 (227) | 4 | Medium quality |
| Malhotra 2017 (604) | 6 | Medium quality |
| Marlow 2017a (228) | 5 | Medium quality |
| Marques 2022 (229) | 5 | Medium quality |
| Martinez-Huedo 2012 (605) | 7 | High quality |
| Martín-López 2012 (230) | 5 | Medium quality |
| Massat 2015 (606) | 5 | Medium quality |
| Mboineki 2020 (231) | 7 | High quality |
| McDaniel 2021 (232) | 5 | Medium quality |
| Mengistu 2022 (233) | 6 | Medium quality |
| Menvielle 2014 (609) | 4 | Medium quality |
| Menvielle 2018 (610) | 7 | High quality |
| Midaksa 2022 (234) | 4 | Medium quality |
| Milenkov 2020 (611) | 5 | Medium quality |
| Miles-Richardson 2017 (235) | 6 | Medium quality |
| Mo 2014 (613) | 6 | Medium quality |
| Moreno 2019 (614) | 7 | High quality |
| Moss 2019 (615) | 6 | Medium quality |
| Moss 2022 (616) | 7 | High quality |
| Mpamani 2019 (237) | 5 | Medium quality |
| Mukem 2015 (617) | 7 | High quality |
| Murphy 2021 (618) | 6 | Medium quality |
| Ncube 2015 (238) | 7 | High quality |
| Ndejjo 2016 (239) | 5 | Medium quality |
| Nega 2018 (240) | 6 | Medium quality |
| Nessler 2019 (241) | 5 | Medium quality |
| Ng'ang'a 2018 (242) | 7 | High quality |
| Ngwenya 2018 (243) | 7 | High quality |
| Nicholls 2017 (620) | 6 | Medium quality |
| Niedzwiedz 2019 (621) | 5 | Medium quality |
| Niedzwiedz 2020 (622) | 5 | Medium quality |
| Nigussie 2019 (244) | 6 | Medium quality |
| Nuche-Berenguer 2021 (623) | 4 | Medium quality |
| Nunes 2021 (245) | 6 | Medium quality |
| Nuño 2012 (624) | 5 | Medium quality |
| Nwabichie 2018 (246) | 8 | High quality |
| Olesen 2012 (247) | 6 | Medium quality |
| Oliveira 2014 (248) | 7 | High quality |
| Orang'O 2016 (249) | 5 | Medium quality |
| Östensson 2015 (251) | 3 | Low quality |
| Ouk 2020 (252) | 6 | Medium quality |
| Padela 2014 (253) | 6 | Medium quality |
| Pang 2017 (626) | 6 | Medium quality |
| Peltzer 2014 (628) | 6 | Medium quality |
| Pengpid 2021 (629) | 7 | High quality |
| Petkeviciene 2018 (255) | 6 | Medium quality |
| Phaiphichit 2022 (256) | 7 | High quality |
| Phaswana-Mafuya 2018 (630) | 6 | Medium quality |
| Poliquin 2013 (257) | 3 | Low quality |
| Pope 2021 (258) | 4 | Medium quality |
| Portero de la Cruz 2022 (259) | 6 | Medium quality |
| Perng 2013 (254) | 5 | Medium quality |
| Qayum 2021 (260) | 5 | Medium quality |
| Ranjit 2016 (261) | 7 | High quality |
| Reichheld 2020 (262) | 4 | Medium quality |
| Reiter 2015 (263) | 5 | Medium quality |
| Ricardo-Rodrigues 2015a (632) | 7 | High quality |
| Richard 2015 (264) | 5 | Medium quality |
| Riza 2017 (633) | 4 | Medium quality |
| Rondet 2014 (634) | 6 | Medium quality |
| Rouge 2019 (266) | 5 | Medium quality |
| Rosser 2015 (265) | 4 | Medium quality |
| Ruddies 2020 (267) | 4 | Medium quality |
| Saha 2021 (635) | 5 | Medium quality |
| Sakellariou 2017 (637) | 7 | High quality |
| Sallah 2019 (268) | 5 | Medium quality |
| Sarah 2022 (269) | 7 | High quality |
| Sawadogo 2014 (270) | 4 | Medium quality |
| Sentell 2015 (638) | 6 | Medium quality |
| Seo 2018 (272) | 7 | High quality |
| Shin 2018 (273) | 8 | High quality |
| Shrestha 2022 (274) | 6 | Medium quality |
| Sicsic 2014 (640) | 5 | Medium quality |
| Silvera 2020 (275) | 6 | Medium quality |
| So 2017 (276) | 6 | Medium quality |
| Solomon 2019 (277) | 5 | Medium quality |
| Songsiriphan 2020 (278) | 4 | Medium quality |
| Studts 2013 (280) | 4 | Medium quality |
| Subba 2022 (641) | 5 | Medium quality |
| Tapera 2017 (281) | 4 | Medium quality |
| Tapera 2019b (282) | 6 | Medium quality |
| Tawiah 2022 (283) | 5 | Medium quality |
| Tchounga 2019 (284) | 7 | High quality |
| Teame 2019 (285) | 4 | Medium quality |
| Tekle 2020 (286) | 7 | High quality |
| Telvizian 2021 (643) | 5 | Medium quality |
| Thapa 2018 (287) | 4 | Medium quality |
| Thompson 2020 (288) | 5 | Medium quality |
| Traoré 2020 (289) | 7 | High quality |
| Trinh 2016 (647) | 4 | Medium quality |
| Ubah 2022 (290) | 4 | Medium quality |
| Vajda 2022 (291) | 4 | Medium quality |
| Valdovinos 2016 (648) | 7 | High quality |
| Van Manh 2020 (649) | 4 | Medium quality |
| Visanuyothin 2015 (292) | 6 | Medium quality |
| Watson 2017 (294) | 7 | High quality |
| Weitlauf 2013 (295) | 7 | High quality |
| Woldetsadik 2020 (296) | 5 | Medium quality |
| Wongwatcharanukul 2014 (297) | 6 | Medium quality |
| Xiang 2015 (654) | 7 | High quality |
| Xie 2022 (655) | 6 | Medium quality |
| Yamashita 2020 (656) | 6 | Medium quality |
| Zhang 2020 (298) | 7 | High quality |
| Brown 2019 (139) | 6 | Medium quality |
| De Prez 2021 (157) | 7 | High quality |
| Do 2015 (162) | 5 | Medium quality |
| Ebu 2018a (165) | 4 | Medium quality |
| Gutarra 2021 (575) | 4 | Medium quality |
| Herriges 2021 (579) | 5 | Medium quality |
| Horner-Johnson 2014a (581) | 6 | Medium quality |
| Jolidon 2021 (586) | 6 | Medium quality |
| Kang 2014 (588) | 7 | High quality |
| Kindratt 2020 (594) | 6 | Medium quality |
| Kiran 2019 (595) | 6 | Medium quality |
| Kouyoumdjian 2018 (214) | 7 | High quality |
| Vora 2020 (293) | 3 | Low quality |
| Lee 2019c (218) | 5 | Medium quality |
| Moore 2015 (236) | 6 | Medium quality |
| Oladeru 2022 (625) | 6 | Medium quality |
| Osingada 2015 (250) | 4 | Medium quality |
| Seay 2017 (271) | 3 | Low quality |
| Stenzel 2022 (279) | 5 | Medium quality |
| Vrinten 2019 (651) | 7 | High quality |
| Weisband 2021 (652) | 7 | High quality |
| Willems 2020 (653) | 6 | Medium quality |

**Table 8. Quality assessment of the studies reporting results for colorectal cancer (n=246)**

| **Study name** | **Total score** | **Interpretation** |
| --- | --- | --- |
| Ahmed 2013 (299) | 6 | Medium quality |
| Almadi 2019 (300) | 5 | Medium quality |
| Aschwanden 2019 (551) | 4 | Medium quality |
| Azimi 2020 (301) | 3 | Low quality |
| Bae 2014 (302) | 4 | Medium quality |
| Bardach 2012 (303) | 6 | Medium quality |
| Batai 2020 (553) | 6 | Medium quality |
| Bernardo 2018 (304) | 5 | Medium quality |
| Beyer 2016 (555) | 6 | Medium quality |
| Bhimla 2020 (305) | 6 | Medium quality |
| Bocci 2017 (306) | 6 | Medium quality |
| Brandt 2012 (307) | 6 | Medium quality |
| Brown 2015 (308) | 7 | High quality |
| Bui 2018b (309) | 7 | High quality |
| Bujang 2021 (310) | 5 | Medium quality |
| Buron 2017 (311) | 5 | Medium quality |
| Bynum 2012 (312) | 6 | Medium quality |
| Calo 2015 (313) | 6 | Medium quality |
| Camacho-Rivera 2019 (558) | 5 | Medium quality |
| Cardoso 2020 (314) | 4 | Medium quality |
| Carrasco-Garrido 2014 (559) | 5 | Medium quality |
| Carrozzi 2015 (560) | 6 | Medium quality |
| Castañeda-Avila 2021 (315) | 6 | Medium quality |
| Cataneo 2022 (316) | 4 | Medium quality |
| Chaiarch 2021 (317) | 4 | Medium quality |
| Chan 2022a (318) | 5 | Medium quality |
| Changoor 2018 (319) | 7 | High quality |
| Charkhchi 2019 (561) | 7 | High quality |
| Chatterjee 2015 (320) | 6 | Medium quality |
| Chido-Amajuoyi 2019 (321) | 5 | Medium quality |
| Choi 2018c (322) | 5 | Medium quality |
| Chuang 2021 (323) | 5 | Medium quality |
| Chudy-Onwugaje 2020 (324) | 6 | Medium quality |
| Clarke 2021 (325) | 7 | High quality |
| Cofie 2020 (326) | 6 | Medium quality |
| Cohen 2012 (327) | 5 | Medium quality |
| Courtney 2013a (328) | 5 | Medium quality |
| Courtney 2013b (329) | 3 | Low quality |
| Davis 2017 (330) | 4 | Medium quality |
| Dawidowicz 2020 (565) | 5 | Medium quality |
| Decker 2015b (331) | 6 | Medium quality |
| Decker 2016 (332) | 3 | Low quality |
| Deding 2019 (333) | 6 | Medium quality |
| Deshpande 2012 (566) | 6 | Medium quality |
| Diaz 2013 (334) | 6 | Medium quality |
| Dodd 2019 (335) | 5 | Medium quality |
| Douma 2019 (336) | 5 | Medium quality |
| Eke 2019 (337) | 4 | Medium quality |
| Elangovan 2021 (338) | 7 | High quality |
| Ellis 2018 (339) | 6 | Medium quality |
| Eng 2020 (569) | 5 | Medium quality |
| Eze 2019 (340) | 6 | Medium quality |
| Farr 2022 (341) | 6 | Medium quality |
| Fawns-Ritchie 2022 (342) | 7 | High quality |
| Fiala 2022 (343) | 5 | Medium quality |
| Fujiwara 2018 (571) | 6 | Medium quality |
| Gale 2015 (344) | 5 | Medium quality |
| Gawron 2021 (572) | 4 | Medium quality |
| Ghai 2020 (345) | 5 | Medium quality |
| Gofine 2018 (346) | 7 | High quality |
| Gonzalez 2020 (347) | 5 | Medium quality |
| Gray 2021 (348) | 5 | Medium quality |
| Greene 2012 (349) | 6 | Medium quality |
| Guo 2021b (574) | 6 | Medium quality |
| Gutarra 2021 (575) | 4 | Medium quality |
| Halbert 2016 (350) | 4 | Medium quality |
| Hanske 2016 (577) | 6 | Medium quality |
| Hategekimana 2016 (351) | 6 | Medium quality |
| He 2020 (352) | 5 | Medium quality |
| Holden 2014 (580) | 7 | High quality |
| Homayoon 2013 (353) | 5 | Medium quality |
| Horner-Johnson 2014b (354) | 6 | Medium quality |
| Huang 2019a (355) | 4 | Medium quality |
| Huang 2019b (356) | 6 | Medium quality |
| Huang 2019c (357) | 3 | Low quality |
| Huang 2020a (358) | 7 | High quality |
| Huang 2020b (359) | 5 | Medium quality |
| Huang 2021a (360) | 6 | Medium quality |
| Huang 2021b (583) | 7 | High quality |
| Huang 2021c (361) | 5 | Medium quality |
| Hughes 2015 (362) | 5 | Medium quality |
| Hughes 2018 (363) | 5 | Medium quality |
| Idowu 2016a (364) | 6 | Medium quality |
| Ilgaz 2018 (365) | 4 | Medium quality |
| Ishii 2021 (584) | 6 | Medium quality |
| Jin 2019a (366) | 6 | Medium quality |
| Jin 2019b (367) | 4 | Medium quality |
| Jun 2013 (368) | 7 | High quality |
| Juon 2018 (369) | 5 | Medium quality |
| Kang 2014 (588) | 7 | High quality |
| Kang 2017 (370) | 5 | Medium quality |
| Karadeniz 2019 (589) | 4 | Medium quality |
| Kearns 2018 (371) | 3 | Low quality |
| Kelly 2017 (590) | 6 | Medium quality |
| Kendall 2013 (372) | 7 | High quality |
| Khoja 2018 (373) | 6 | Medium quality |
| Kim 2019b (593) | 7 | High quality |
| Kindratt 2020 (594) | 6 | Medium quality |
| Kiran 2019 (595) | 6 | Medium quality |
| Klabunde 2015 (374) | 6 | Medium quality |
| Knight 2015 (375) | 5 | Medium quality |
| Kobayashi 2014 (376) | 3 | Low quality |
| Koo 2012 (377) | 7 | High quality |
| Kroupa 2019 (378) | 7 | High quality |
| Kurani 2020 (596) | 5 | Medium quality |
| Laiyemo 2019 (379) | 5 | Medium quality |
| Le Breton 2012 (380) | 4 | Medium quality |
| Lee 2018b (381) | 4 | Medium quality |
| Lee 2018d (382) | 4 | Medium quality |
| Lee 2019b (383) | 6 | Medium quality |
| Lee 2020 (598) | 6 | Medium quality |
| Lee 2021(599) | 6 | Medium quality |
| Leung 2016 (384) | 4 | Medium quality |
| Lin 2013 (385) | 5 | Medium quality |
| Lin 2017 (386) | 5 | Medium quality |
| Llanos 2015 (387) | 5 | Medium quality |
| Lo 2013 (600) | 7 | High quality |
| Lo 2015 (388) | 6 | Medium quality |
| Lofters 2015 (601) | 6 | Medium quality |
| Lofters 2019 (602) | 8 | High quality |
| MacDonald 2022 (603) | 7 | High quality |
| Majeed 2022 (389) | 4 | Medium quality |
| Malhotra 2017 (604) | 6 | Medium quality |
| Maly 2014 (390) | 5 | Medium quality |
| Mansfield 2018 (391) | 4 | Medium quality |
| Mansouri 2013 (392) | 5 | Medium quality |
| Marucci 2022 (393) | 5 | Medium quality |
| Mastrokostas 2018 (394) | 4 | Medium quality |
| May 2017 (395) | 7 | High quality |
| May 2014 (397) | 4 | Medium quality |
| May 2019 (396) | 7 | High quality |
| Mayhand 2021 (398) | 6 | Medium quality |
| McEvoy 2021 (399) | 5 | Medium quality |
| McKinney 2014 (400) | 4 | Medium quality |
| Mehta 2022 (607) | 3 | Low quality |
| Mendoza 2022 (608) | 7 | High quality |
| Menéndez 2020 (401) | 4 | Medium quality |
| Menéndez 2022 (402) | 4 | Medium quality |
| Menvielle 2018 (610) | 7 | High quality |
| Miles 2021 (612) | 5 | Medium quality |
| Mitsutake 2012 (403) | 7 | High quality |
| Mo 2014 (613) | 6 | Medium quality |
| Molina-Barceló 2014 (404) | 6 | Medium quality |
| Momplaisir 2012 (405) | 7 | High quality |
| Monet 2021 (406) | 6 | Medium quality |
| Moreno 2019 (614) | 7 | High quality |
| Mosli 2017 (407) | 4 | Medium quality |
| Moss 2019 (615) | 6 | Medium quality |
| Murphy 2021 (618) | 6 | Medium quality |
| Myong 2012a (408) | 6 | Medium quality |
| Myong 2012b (409) | 7 | High quality |
| Nagelhout 2017 (410) | 5 | Medium quality |
| Nápoles 2014 (411) | 6 | Medium quality |
| Narayan 2021a (412) | 6 | Medium quality |
| Nguyen 2022 (619) | 3 | Low quality |
| Nicholls 2017 (620) | 6 | Medium quality |
| Nieves-Jimenez 2022 (413) | 5 | Medium quality |
| Nuche-Berenguer 2021 (623) | 4 | Medium quality |
| Ojinnaka 2015 (414) | 5 | Medium quality |
| Ooi 2019 (415) | 5 | Medium quality |
| Otiniano 2013 (416) | 6 | Medium quality |
| Pancar 2021 (417) | 6 | Medium quality |
| Pang 2017 (626) | 6 | Medium quality |
| Park 2017 (627) | 7 | High quality |
| Parsons 2012 (418) | 5 | Medium quality |
| Patel 2012 (419) | 7 | High quality |
| Pausawasdi 2022 (420) | 6 | Medium quality |
| Pengpid 2021 (629) | 7 | High quality |
| Ponce 2012 (631) | 5 | Medium quality |
| Poroes 2020 (421) | 5 | Medium quality |
| Puthashanan 2021 (422) | 4 | Medium quality |
| Qumseya 2014 (423) | 6 | Medium quality |
| Ramai 2019 (424) | 3 | Low quality |
| Ramazani 2021 (425) | 5 | Medium quality |
| Rastogi 2019 (426) | 6 | Medium quality |
| Swaminathan 2020 (427) | 4 | Medium quality |
| Ricardo-Rodrigues 2015b (428) | 4 | Medium quality |
| Rogers 2020 (429) | 5 | Medium quality |
| Rogers 2021 (430) | 5 | Medium quality |
| Rogers 2022 (431) | 6 | Medium quality |
| Ross 2020 (88) | 6 | Medium quality |
| Samuel 2021 (432) | 5 | Medium quality |
| Schonberg 2015 (433) | 7 | High quality |
| Seibert 2017 (434) | 6 | Medium quality |
| Sekhon 2021 (435) | 3 | Low quality |
| Sentell 2013 (436) | 6 | Medium quality |
| Sentell 2015 (638) | 6 | Medium quality |
| Shahidi 2013 (437) | 6 | Medium quality |
| Shariff-Marco 2013 (438) | 4 | Medium quality |
| Shete 2021 (639) | 5 | Medium quality |
| Shin 2017 (439) | 5 | Medium quality |
| Shin 2020b (440) | 4 | Medium quality |
| Siantz 2017 (441) | 6 | Medium quality |
| Sicsic 2014 (640) | 5 | Medium quality |
| Simkin 2019 (442) | 6 | Medium quality |
| Sing 2013 (443) | 6 | Medium quality |
| Singal 2013 (444) | 5 | Medium quality |
| Skau 2022 (445) | 5 | Medium quality |
| So 2012 (446) | 6 | Medium quality |
| Solís-Ibinagagoitia 2020 (447) | 5 | Medium quality |
| Stanley 2019 (448) | 4 | Medium quality |
| Stevens 2019 (449) | 5 | Medium quality |
| Suh 2015 (450) | 6 | Medium quality |
| Swaminathan 2019 (451) | 3 | Low quality |
| Swaminathan 2020 (427) | 4 | Medium quality |
| Sy 2018 (452) | 4 | Medium quality |
| Tabaac 2018 (453) | 6 | Medium quality |
| Taheri-Kharameh 2015 (454) | 5 | Medium quality |
| Takeuchi 2020 (642) | 5 | Medium quality |
| Taş 2019 (455) | 3 | Low quality |
| Tastan 2013 (456) | 5 | Medium quality |
| Telvizian 2021 (643) | 5 | Medium quality |
| Theodoropoulos 2022 (644) | 6 | Medium quality |
| Thompson 2013 (457) | 5 | Medium quality |
| Thompson 2014 (458) | 5 | Medium quality |
| Todorov 2018 (459) | 4 | Medium quality |
| Torosian 2021 (460) | 5 | Medium quality |
| Towne 2014 (645) | 5 | Medium quality |
| Tracy 2013 (646) | 6 | Medium quality |
| Trinh 2016 (647) | 4 | Medium quality |
| Valdovinos 2016 (648) | 7 | High quality |
| Van Manh 2020 (649) | 4 | Medium quality |
| Vanaclocha-Espi 2017 (461) | 5 | Medium quality |
| Varlow 2014 (462) | 6 | Medium quality |
| Viramontes 2019 (463) | 5 | Medium quality |
| Viramontes 2020 (464) | 7 | High quality |
| Von Wagner 2013 (650) | 6 | Medium quality |
| Von Wagner 2019 (465) | 3 | Low quality |
| Von Wagner 2020 (466) | 6 | Medium quality |
| Vrinten 2019 (651) | 7 | High quality |
| Wallace 2012 (467) | 3 | Low quality |
| Walsh 2013 (468) | 6 | Medium quality |
| Wang 2017 (469) | 6 | Medium quality |
| Wangmar 2018 (470) | 5 | Medium quality |
| Weisband 2021 (652) | 7 | High quality |
| Weiss 2013 (471) | 5 | Medium quality |
| Wilcox 2015 (472) | 5 | Medium quality |
| Wong 2013a (473) | 7 | High quality |
| Wong 2013c (474) | 5 | Medium quality |
| Xie 2022 (655) | 6 | Medium quality |
| Yager 2014 (475) | 5 | Medium quality |
| Yamashita 2020 (656) | 6 | Medium quality |
| Zajac 2017 (476) | 6 | Medium quality |
| Zamorano-Leon 2020 (477) | 5 | Medium quality |
| Zhu 2021 (478) | 5 | Medium quality |
| Zhu 2022 (479) | 7 | High quality |

**Table 9. Quality assessment of the studies reporting results for lung cancer (n=37)**

| **Study name** | **Total score** | **Interpretation** |
| --- | --- | --- |
| Abeyweera 2019 (489) | 4 | Medium quality |
| Bringedal 2019 (556) | 7 | High quality |
| Barta 2021 (490) | 4 | Medium quality |
| Broadbent 2022 (491) | 4 | Medium quality |
| Bui 2018a (492) | 5 | Medium quality |
| Cam 2015 (493) | 6 | Medium quality |
| Cataldo 2016 (494) | 6 | Medium quality |
| Delmerico 2014 (495) | 5 | Medium quality |
| Doria-Rose 2012 (496) | 7 | High quality |
| Ferguson 2020 (497) | 3 | Low quality |
| Fujiwara 2018 (571) | 6 | Medium quality |
| Gudina 2021 (498) | 3 | Low quality |
| Guo 2020 (499) | 4 | Medium quality |
| Kim 2021 (500) | 3 | Low quality |
| Lake 2020 (501) | 5 | Medium quality |
| Lewis 2019 (502) | 6 | Medium quality |
| Lowenstein 2022 (503) | 6 | Medium quality |
| Mukthinuthalapati 2020 (504) | 5 | Medium quality |
| Narayan 2021b (412) | 6 | Medium quality |
| Neslund-Dudas 2021 (505) | 4 | Medium quality |
| Núñez 2021 (507) | 5 | Medium quality |
| Nunez 2021 (506) | 3 | Low quality |
| Quaife 2018 (509) | 7 | High quality |
| Quaife 2021a (510) | 5 | Medium quality |
| Percac-Lima 2019 (508) | 6 | Medium quality |
| Raju 2020 (511) | 5 | Medium quality |
| Rajupet 2017 (512) | 5 | Medium quality |
| Raz 2018 (513) | 5 | Medium quality |
| Rennert 2020 (514) | 4 | Medium quality |
| Rustagi 2022 (515) | 6 | Medium quality |
| See 2020 (516) | 6 | Medium quality |
| Spalluto 2022 (517) | 7 | High quality |
| Stowell 2020 (518) | 6 | Medium quality |
| Stowell 2021 (519) | 4 | Medium quality |
| Tanner 2013 (520) | 6 | Medium quality |
| Veliz 2019 (521) | 6 | Medium quality |
| Xie 2021 (522) | 3 | Low quality |

**Table 10. Quality assessment of the studies reporting results for gastric cancer (n=12)**

| **Study name** | **Total score** | **Interpretation** |
| --- | --- | --- |
| Chang 2015 (480) | 5 | Medium quality |
| Fujiwara 2018 (571) | 6 | Medium quality |
| Kang 2014 (588) | 7 | High quality |
| Kim 2020 (481) | 6 | Medium quality |
| Lee 2015 (482) | 4 | Medium quality |
| Liu 2019 (483) | 4 | Medium quality |
| Park 2013 (484) | 6 | Medium quality |
| Park 2017 (627) | 7 | High quality |
| Shim 2019 (485) | 6 | Medium quality |
| Shin 2012 (486) | 6 | Medium quality |
| Shin 2016 (487) | 7 | High quality |
| Yu 2022 (488) | 4 | Medium quality |

**Table 11. Quality assessment of the studies reporting results for prostate cancer (n=38)**

| **Study name** | **Total score** | **Interpretation** |
| --- | --- | --- |
| Abuadas 2015 (523) | 5 | Medium quality |
| Arega 2020 (524) | 4 | Medium quality |
| Aschwanden 2019 (551) | 4 | Medium quality |
| Batai 2020 (553) | 6 | Medium quality |
| Bilgili 2019 (525) | 4 | Medium quality |
| Bugoye 2019 (526) | 7 | High quality |
| Burns 2012 (527) | 5 | Medium quality |
| Camacho-Rivera 2019 (558) | 5 | Medium quality |
| Carrasco-Garrido 2014 (559) | 5 | Medium quality |
| Cobran 2014 (528) | 5 | Medium quality |
| Ddumba 2019 (529) | 4 | Medium quality |
| Dean 2015 (530) | 7 | High quality |
| Frego 2022 (531) | 6 | Medium quality |
| Gift 2020 (532) | 5 | Medium quality |
| Glenn 2012 (533) | 7 | High quality |
| Goodwin 2020 (573) | 7 | High quality |
| Haider 2017 (534) | 5 | Medium quality |
| Herriges 2021 (579) | 5 | Medium quality |
| Kangmennaang 2016a (535) | 6 | Medium quality |
| Kangmennaang 2016b (536) | 5 | Medium quality |
| Karadeniz 2019 (589) | 4 | Medium quality |
| Kim 2018 (591) | 4 | Medium quality |
| Lee 2013a (537) | 4 | Medium quality |
| Ma 2020 (538) | 6 | Medium quality |
| Mbugua 2021 (539) | 5 | Medium quality |
| Moreno 2019 (614) | 7 | High quality |
| Morlando 2017 | 4 | Medium quality |
| Moss 2019 (615) | 6 | Medium quality |
| Murphy 2021 (618) | 6 | Medium quality |
| Ogunsanya 2016 (541) | 5 | Medium quality |
| Opondo 2022 (542) | 5 | Medium quality |
| Sahoo 2020 (636) | 6 | Medium quality |
| So 2014 (543) | 6 | Medium quality |
| Takeuchi 2020 (642) | 5 | Medium quality |
| Tasian 2012 (544) | 5 | Medium quality |
| Trinh 2016 (647) | 4 | Medium quality |
| Wong 2020 (545) | 6 | Medium quality |
| Yeboah-Asiamah 2017 (546) | 7 | High quality |

# **References**

1. Abdel-Aziz S, Amin TT, Al-Gadeeb MB, Alhassar A, Al-Ramadan A, Al-Helal M, et al. Perceived barriers to breast cancer screening among Saudi women at primary care setting. Journal of Preventive Medicine Hygiene. 2018;59(1):E20; doi:10.22034/APJCP.2017.18.9.2409.

2. Ahmadian M, Samah AA, Redzuan Mr, Emby Z. Predictors of mammography screening among Iranian women attending outpatient clinics in Tehran, Iran. Asian Pacific Journal of Cancer Prevention. 2012;13(3):969-74; doi:10.7314/APJCP.2012.13.3.969.

3. Alatrash M. Determinants of breast cancer screening in three Arab American women subgroups. Journal of Transcultural Nursing. 2021;32(6):749-56; doi:10.1177/10436596211008215.

4. Al-Azri M, Al-Rubaie K, Al-Ghafri S, Al-Hinai M, Panchatcharam SM. Barriers and attitudes toward breast cancer screening among Omani women. Asian Pacific Journal of Cancer Prevention. 2020;21(5):1339; doi:10.31557/APJCP.2020.21.5.1339.

5. Al-Hanawi MK, Hashmi R, Almubark S, Qattan AM, Pulok MH. Socioeconomic inequalities in uptake of breast cancer screening among Saudi women: A cross-sectional analysis of a national survey. International Journal of Environmental Research Public Health. 2020;17(6):2056; doi:10.3390/ijerph17062056.

6. Asgary R, Garland V, Sckell B. Breast cancer screening among homeless women of New York City shelter-based clinics. Women's Health Issues. 2014;24(5):529-34; doi:10.1016/j.whi.2014.06.002.

7. Assefa AA, Abera G, Geta M. Breast cancer screening practice and associated factors among women aged 20–70 years in urban settings of SNNPR, Ethiopia. Breast Cancer: Targets Therapy. 2021:9-19; doi:10.2147/BCTT.S286441.

8. Balas N, Yun H, Jaeger BC, Aung M, Jolly PE. Factors associated with breast cancer screening behaviors in a sample of Jamaican women in 2013. Women Health. 2020;60(9):1032-9; doi:10.1080/03630242.2020.1789260.

9. Bao Y, Kwok C, Lee CF. Breast cancer screening behaviors among Chinese women in Mainland China. Nursing Health Sciences. 2018;20(4):445-51; doi:10.1111/nhs.12533.

10. Baughman AW, Brawarsky P, Onega T, Tosteson TD, Wang Q, Tosteson AN, et al. Medical home transformation and breast cancer screening. The American Journal of Managed Care. 2016;22(11):e382.

11. Bawazir A, Bashateh N, Jradi H, Breik AB. Breast cancer screening awareness and practices among women attending primary health care centers in the Ghail Bawazir District of Yemen. Clinical Breast Cancer. 2019;19(1):e20-e9; doi:10.1016/j.clbc.2018.09.005.

12. El Bcheraoui C, Basulaiman M, Wilson S, Daoud F, Tuffaha M, AlMazroa MA, et al. Breast cancer screening in Saudi Arabia: free but almost no takers. PloS One. 2015;10(3):e0119051.

13. Beaber EF, Sprague BL, Tosteson AN, Haas JS, Onega T, Schapira MM, et al. Multilevel predictors of continued adherence to breast cancer screening among women ages 50–74 years in a screening population. Journal of Women's Health. 2019;28(8):1051-9; doi:10.1089/jwh.2018.6997.

14. Berens E-M, Stahl L, Yilmaz-Aslan Y, Sauzet O, Spallek J, Razum O. Participation in breast cancer screening among women of Turkish origin in Germany–a register-based study. BMC Women's Health. 2014;14:1-6; doi:10.1186/1472-6874-14-24.

15. Bhandari D, Shibanuma A, Kiriya J, Hirachan S, Ong KIC, Jimba M. Factors associated with breast cancer screening intention in Kathmandu Valley, Nepal. PloS One. 2021;16(1):e0245856; doi:10.1371/journal.pone.0245856.

16. Cataneo JL, Raicu OA, Schwarzova K, Meidl H, Cruz C. Disparities in Screening for Breast Cancer Based on Limited Language Proficiency. A Retrospective Cohort Propensity Score Matched Study. The American Surgeon. 2020;231(4):S36-S7; doi:10.1016/j.jamcollsurg.2020.07.012.

17. Chan W, Yun L, Austin P, Jaakkimainen R, Booth G, Hux J, et al. Impact of socio‐economic status on breast cancer screening in women with diabetes: a population‐based study. Diabetic Medicine. 2014;31(7):806-12; doi:10.1111/dme.12422.

18. Choi E, Lee YY, Yoon HJ, Lee S, Suh M, Park B, et al. Relationship between cancer worry and stages of adoption for breast cancer screening among Korean women. PloS One. 2015;10(7):e0132351; doi:10.1371/journal.pone.0132351.

19. Choi G-Y, Koh E, Choi S, Cho J-Y. Understanding breast cancer screening behaviors of Korean American women in sociocultural contexts. Social Work in Health Care. 2017;56(1):45-63.

20. Dahlui M, Gan DEH, Taib NA, Pritam R, Lim J. Predictors of breast cancer screening uptake: a pre intervention community survey in Malaysia. Asian Pacific Journal of Cancer Prevention. 2012;13(7):3443-9; doi:10.7314/APJCP.2012.13.7.3443.

21. Donnelly TT, Al Khater A-H, Al-Bader SB, Al Kuwari MG, Al-Meer N, Malik M, et al. Breast cancer screening among Arabic women living in the State of Qatar: Awareness, knowledge, and participation in screening activities. Avicenna. 2012;2012(1):2; doi:10.5339/avi.2012.2.

22. Donnelly TT, Al Khater A-H, Al-Bader SB, Al Kuwari MG, Al-Meer N, Malik M, et al. Beliefs and attitudes about breast cancer and screening practices among Arab women living in Qatar: a cross-sectional study. BMC Women's Health. 2013;13(1):1-16; doi:10.1186/1472-6874-13-49.

23. Donnelly TT, Al Khater A-H, Al Kuwari MG, Al-Bader SB, Al-Meer N, Abdulmalik M, et al. Do socioeconomic factors influence breast cancer screening practices among Arab women in Qatar? BMJ open. 2015;5(1):e005596.

24. Duport N. Characteristics of women using organized or opportunistic breast cancer screening in France. Analysis of the 2006 French Health, Health Care and Insurance Survey. Revue d'épidémiologie et de santé publique. 2012;60(6):421-30; doi:10.1016/j.respe.2012.05.006.

25. Eichholzer M, Richard A, Rohrmann S, Schmid S, Güth U. Overweight, obesity, and breast cancer screening. European Journal of Cancer Prevention. 2016;25(2):130-6; doi:10.1097/CEJ.0000000000000148.

26. El Mhamdi S, Bouanene I, Mhirsi A, Sriha A, Salem KB, Soltani MS. Women’s knowledge, attitudes and practice about breast cancer screening in the region of Monastir (Tunisia). Australian Journal of Primary Health. 2013;19(1):68-73; doi:10.1071/PY11123.

27. Elobaid YE, Aw TC, Grivna M, Nagelkerke N. Breast cancer screening awareness, knowledge, and practice among Arab women in the United Arab Emirates: a cross-sectional survey. PloS One. 2014;9(9):e105783; doi:10.1371/journal.pone.0105783.

28. Ezema RN, Igbokwe CC, Iwuagwu TE, Agbaje OS, Ofuebe JI, Abugu LI, et al. Association of Sociodemographic Factors, Breast Cancer Fear, and Perceived Self-Efficacy With Breast Cancer Screening Behaviors Among Middle-Aged Nigerian Women. Breast Cancer: Basic Clinical Research. 2021;15:11782234211043651; doi:10.1177/11782234211043651.

29. Nik Farid ND, Abdul Aziz N, Al-Sadat N, Jamaludin M, Dahlui M. Clinical breast examination as the recommended breast cancer screening modality in a rural community in Malaysia; what are the factors that could enhance its uptake? PloS One. 2014;9(9):e106469; doi:10.3390/cancers14133076.

30. Fayanju OM, Kraenzle S, Drake BF, Oka M, Goodman MS. Perceived barriers to mammography among underserved women in a Breast Health Center Outreach Program. The American Journal of Surgery. 2014;208(3):425-34.

31. Fleming P, O'Neill S, Owens M, Mooney T, Fitzpatrick P. Intermittent attendance at breast cancer screening. Journal of Public Health Research. 2013;2(2):jphr. 2013. e14; doi:10.4081/jphr.2013.e14.

32. Gan YX, Lao C-K, Chan A. Breast cancer screening behavior, attitude, barriers among middle-aged Chinese women in Macao, China. Journal of Public Health. 2018;40(4):e560-e70; doi:10.1093/pubmed/fdy077.

33. Gang M, Kim JI, Oh KO, Li CY, Song Y. Factors associated with mammography adherence among married Chinese women in Yanbian, China. Asian Pacific Journal of Cancer Prevention. 2013;14(12):7207-13; doi:10.7314/apjcp.2013.14.12.7207.

34. Gilfoyle M, Garcia J, Chaurasia A, Oremus M. Perceived susceptibility to developing cancer and mammography screening behaviour: a cross-sectional analysis of Alberta's Tomorrow Project. Public Health. 2019;177:135-42; doi:10.1016/j.puhe.2019.08.004.

35. Gong J, Kampadellis G, Kong Q, Spijker WJHPI. Factors determining non-attendance in breast cancer screening among women in the Netherlands: a national study. Health Promotion International. 2023;38(3):daac009; doi:10.1093/heapro/daac009.

36. Guilcher SJ, Lofters A, Glazier RH, Jaglal SB, Voth J, Bayoumi AM. Level of disability, multi-morbidity and breast cancer screening: does severity matter? Preventive Medicine. 2014;67:193-8; doi:10.1016/j.ypmed.2014.07.025.

37. Hajian-Tilaki K, Auladi S. Health belief model and practice of breast self-examination and breast cancer screening in Iranian women. Breast cancer. 2014;21:429-34; doi:10.1007/s12282-012-0409-3.

38. Tilaki KH, Auladi S. Awareness, attitude, and practice of breast cancer screening women, and the associated socio-demographic characteristics, in northern Iran. Iranian Journal of Cancer Prevention. 2015;8(4); doi:10.17795/ijcp.3429.

39. Hassan EE, Seedhom AE, Mahfouz EM. Awareness about breast cancer and its screening among rural Egyptian women, Minia District: A population-based study. Asian Pacific Journal of Cancer Prevention. 2017;18(6):1623; doi:10.22034/APJCP.2017.18.6.1623.

40. Hasnain M, Menon U, Ferrans CE, Szalacha L. Breast cancer screening practices among first-generation immigrant muslim women. Journal of Women's Health. 2014;23(7):602-12; doi:10.1089/jwh.2013.4569.

41. Hippman C, Moshrefzadeh A, Lohn Z, Hodgson ZG, Dewar K, Lam M, et al. Breast Cancer and mammography screening: knowledge, beliefs and predictors for Asian immigrant women attending a specialized Clinic in British Columbia, Canada. Journal of Immigrant Minority Health. 2016;18(6):1441-8; doi:10.1007/s10903-015-0332-8.

42. Hsieh H-M, Chang W-C, Shen C-T, Liu Y, Chen F-M, Kang Y-T. Mediation effect of health beliefs in the relationship between health knowledge and uptake of mammography in a National Breast Cancer screening program in Taiwan. Journal of Cancer Education. 2021;36:832-43; doi:10.1007/s13187-020-01711-7.

43. Jadav S, Rajan SS, Abughosh S, Sansgiry SS. The role of socioeconomic status and health care access in breast cancer screening compliance among Hispanics. Journal of Public Health Management Practice. 2015;21(5):467-76; doi:10.1097/PHH.0000000000000235.

44. Jin SW, Lee HY, Lee J. Analyzing factors of breast cancer screening adherence among Korean American women using Andersen’s behavioral model of healthcare services utilization. Journal of Transcultural Nursing. 2019;29(Suppl 2):427; doi:10.1177/1043659618811910.

45. Jin SW, Lee J, Yun Lee H. Analyzing factors associated with decisional stage of adopting breast cancer screening among Korean American women using precaution adoption process model. Ethnicity Health. 2021;26(3):431-47; doi:10.1080/13557858.2018.1520813.

46. Kadaoui N, Guay M, Baron G, St-Cerny J, Lemaire J. Breast cancer screening practices for women aged 35 to 49 and 70 and older. Canadian Family Physician. 2012;58(1):e47-e53.

47. Kangmennaang J, Mkandawire P, Luginaah I. Breast cancer screening among women in Namibia: explaining the effect of health insurance coverage and access to information on screening behaviours. Glob Health Promot. 2019;26(3):50-61; doi:10.1177/1757975917727017.

48. Kardan-Souraki M, Moosazadeh M, Khani S, Hamzehgardeshi Z. Factors related to breast cancer screening in women in the northern part of Iran: A cross-sectional study. Open access Macedonian Journal of Medical Sciences. 2019;7(4):637; doi:10.3889/oamjms.2019.045.

49. Khaliq W, Aamar A, Wright SM. Predictors of non-adherence to breast cancer screening among hospitalized women. PloS One. 2015;10(12):e0145492; doi:10.1371/journal.pone.0145492.

50. Kim J, Lee SK, Lee J, Choi M-Y, Jung SP, Kim MK, et al. Breast cancer screening knowledge and perceived health beliefs among immigrant women in Korea. Journal of Breast Cancer. 2014;17(3):279-86; doi:10.4048/jbc.2014.17.3.279.

51. Kim SE, Bachorik AE, Bertrand KA, Gunn CM. Differences in breast cancer screening practices by diabetes status and race/ethnicity in the United States. Journal of Women's Health. 2022;31(6):848-55; doi:10.1089/jwh.2021.0396.

52. Kirag N, Kızılkaya M. Application of the Champion Health Belief Model to determine beliefs and behaviors of Turkish women academicians regarding breast cancer screening: A cross sectional descriptive study. BMC Women's Health. 2019;19:1-10; doi:10.1186/s12905-019-0828-9.

53. Kırca N, Tuzcu A, Gözüm S. Breast cancer screening behaviors of first degree relatives of women receiving breast cancer treatment and the affecting factors. European journal of breast health. 2018;14(1):23; doi:10.5152/ejbh.2017.3272.

54. Kiyang L-N, Labrecque M, Doualla-Bell F, Turcotte S, Farley C, Cionti Bas M, et al. Family physicians’ intention to support women in making informed decisions about breast cancer screening with mammography: a cross-sectional survey. BMC Research Notes. 2015;8(1):1-7; doi:10.1186/s13104-015-1608-8.

55. Kosog K, Earle M, Stellon E, Nolan C, Wainwright MK, Webb T, et al. Identifying an association between socio‐demographic factors and breast cancer screening adherence in a federally qualified health centre sample in the United States. A retrospective, cross‐sectional study. Health Social Care in the Community. 2020;28(5):1772-9; doi:10.1111/hsc.13002.

56. Kriaucioniene V, Petkeviciene J. Predictors and trend in attendance for breast cancer screening in Lithuania, 2006–2014. International Journal of Environmental Research Public Health. 2019;16(22):4535; doi:10.3390/ijerph16224535.

57. Kwok C, Fong DY. Breast cancer screening practices among Hong Kong Chinese women. Cancer Nursing. 2014;37(1):59-65; doi:10.1097/NCC.0b013e31827f0a9d.

58. Kwok C, Endrawes G, Lee CF. Cultural beliefs and attitudes about breast cancer and screening practices among Arabic women in Australia. Cancer Nursing. 2016;39(5):367-74; doi:10.1097/NCC.0000000000000325.

59. Kwok C, Lee M-J, Lee CF. The Role of Education in Breast Cancer Beliefs and Screening Practices Among Korean Women–A quantitative study. Journal of Transcultural Nursing. 2022;33(3):287-96; doi:10.1177/10436596211066812.

60. Lam M, Kwok C, Lee MJ. Prevalence and sociodemographic correlates of routine breast cancer screening practices among migrant‐Australian women. Australian New Zealand Journal of Public Health. 2018;42(1):98-103; doi:10.1111/1753-6405.12752.

61. Lawson MB, Lee CI, Hippe DS, Chennupati S, Fedorenko CR, Malone KE, et al. Receipt of Screening Mammography by Insured Women Diagnosed With Breast Cancer and Impact on Outcomes. Journal of the National Comprehensive Cancer Network. 2021;19(10):1156-64; doi:10.6004/jnccn.2020.7801.

62. Lee HY, Lee MH, Jang YJ, Lee DK. Breast cancer screening disparity among Korean American immigrant women in midwest. Asian Pacific Journal of Cancer Prevention. 2017;18(10):2663; doi:10.22034/APJCP.2017.18.10.2663.

63. Lee S-Y, Lee E, Natipagon-Shah B, Toyama J. Factors associated with breast cancer screening among Korean American women in California: Results from the California Health Interview Survey 2015–2016. Asian Pacific Journal of Cancer Prevention. 2018;19(11):3271; doi:10.31557/apjcp.2018.19.11.3271.

64. Lemogne C, Turinici M, Panjo H, Ngo C, Canoui‐Poitrine F, Chauvet‐Gelinier JC, et al. Personality and breast cancer screening in women of the GAZEL cohort study. Cancer Medicine. 2018;7(2):515-24; doi:10.1002/cam4.1268.

65. Leung J, McKenzie S, Martin J, Dobson A, McLaughlin D. Longitudinal patterns of breast cancer screening: mammography, clinical, and breast self-examinations in a rural and urban setting. Women's Health Issues. 2014;24(1):e139-e46; doi:10.1016/j.whi.2013.11.005.

66. Llaneza DH, Kim H, Correa-Fernández V. A Health Inequity: Associations Between Cigarette Smoking Status and Mammogram Screening Among Women of Color. Nicotine Tobacco Research. 2023;25(1):66-72; doi:10.1093/ntr/ntac175.

67. Lopez D, Miles RC, Flores EJ, Lehman CD, Narayan AK. Breast cancer screening in Puerto Rico and other US territories: findings from the 2016 behavioral risk factor surveillance system survey. Journal of Health Care for the Poor Underserved. 2020;31(1):340-52; doi:10.1353/hpu.2020.0026.

68. Ma GX, Gao W, Lee S, Wang M, Tan Y, Shive SE. Health seeking behavioral analysis associated with breast cancer screening among Asian American women. International Journal of Women's Health. 2012:235-43; doi:10.2147/IJWH.S30738.

69. Martín-López R, Jiménez-García R, Lopez-de-Andres A, Hernández-Barrera V, Jiménez-Trujillo I, Gil-de-Miguel A, et al. Inequalities in uptake of breast cancer screening in Spain: analysis of a cross-sectional national survey. Public Health. 2013;127(9):822-7; doi:10.1016/j.puhe.2013.03.006.

70. Miles RC, Lehman CD, Mercaldo SF, Tamimi RM, Dontchos BN, Narayan AK. Obesity and breast cancer screening: Cross‐sectional survey results from the behavioral risk factor surveillance system. Cancer. 2019;125(23):4158-63; doi:10.1002/cncr.32430.

71. Mohan D, Su TT, Donnelly M, Hoe WMK, Schliemann D, Tan MM, et al. Breast cancer screening in semi-rural Malaysia: Utilisation and barriers. International Journal of Environmental Research Public Health. 2021;18(23):12293; doi:10.3390/ijerph182312293.

72. Morère JF, Eisinger F, Couraud S, Greillier L, Touboul C, Lhomel C, et al. Abstract P1-02-05: Who drops out of breast cancer screening? Results from the EDIFICE 6 survey. Cancer Research. 2019;79:P1-02; doi:10.1158/1538-7445.SABCS18-P1-02-05.

73. Mukem S, Sriplung H, McNeil E, Tangcharoensathien V. Breast cancer screening among women in Thailand: analyses of population-based household surveys. J Med Assoc Thai. 2014;97(11):1106-18.

74. Nandam N, Gaebler-Spira D, Byrne R, Wolfman J, Reis JP, Hung CW, et al. Breast cancer screening in women with cerebral palsy: Could care delivery be improved? Disability Health Journal. 2018;11(3):435-41; doi:10.1016/j.dhjo.2018.02.002.

75. Ngan TT, Jenkins C, Minh HV, Donnelly M, O’Neill C. Breast cancer screening practices among Vietnamese women and factors associated with clinical breast examination uptake. PLoS One. 2022;17(5):e0269228; doi:10.1371/journal.pone.0269228.

76. Nojomi M, Namiranian N, Myers RE, Razavi-Ratki S-K, Alborzi F. Factors associated with breast cancer screening decision stage among women in Tehran, Iran. International Journal of Preventive Medicine. 2014;5(2):196.

77. O’Hara J, McPhee C, Dodson S, Cooper A, Wildey C, Hawkins M, et al. Barriers to breast cancer screening among diverse cultural groups in Melbourne, Australia. International Journal of Environmental Research Public Health. 2018;15(8):1677; doi:10.3390/ijerph15081677.

78. Ogunsiji OO, Kwok C, Fan LC. Breast cancer screening practices of African migrant women in Australia: a descriptive cross-sectional study. BMC Women's Health. 2017;17:1-10; doi:10.1186/s12905-017-0384-0.

79. Okui T. Analysis of predictors of breast cancer screening among Japanese women using nationally representative survey data, 2001–2013. Asian Pacific Journal of Cancer Prevention. 2021;22(1):171; doi:10.31557/APJCP.2021.22.1.171.

80. Pagán JA, Brown CJ, Asch DA, Armstrong K, Bastida E, Guerra C. Health literacy and breast cancer screening among Mexican American women in South Texas. Journal of Cancer Education. 2012;27:132-7; doi:10.1007/s13187-011-0239-6.

81. Paranjpe A, Zheng C, Chagpar AB. Disparities in breast cancer screening between Caucasian and Asian American women. Journal of Surgical Research. 2022;277:110-5; doi:10.1016/j.jss.2022.03.032.

82. Park C, Ma X, Park SK, Lawson KA. Association of depression with adherence to breast cancer screening among women aged 50 to 74 years in the United States. Journal of Evaluation in Clinical Practice. 2020;26(6):1677-88; doi:10.1111/jep.13356.

83. Patel K, Kanu M, Liu J, Bond B, Brown E, Williams E, et al. Factors influencing breast cancer screening in low-income African Americans in Tennessee. Journal of Community Health. 2014;39:943-50; doi:10.1007/s10900-014-9834-x.

84. Racine L, Andsoy I, Maposa S, Vatanparast H, Fowler-Kerry S. Examination of breast cancer screening knowledge, attitudes, and beliefs among Syrian refugee women in a Western Canadian province. Canadian Journal of Nursing Research. 2022;54(2):177-89; doi:10.1177/08445621211013200.

85. Radhakrishnan A, Nowak SA, Parker AM, Visvanathan K, Pollack CE. Linking physician attitudes to their breast cancer screening practices: a survey of US primary care providers and gynecologists. Preventive Medicine. 2018;107:90-102; doi:10.1016/j.ypmed.2017.11.010.

86. Rollet Q, Guillaume É, Launay L, Launoy G. Socio-Territorial Inequities in the French National Breast Cancer Screening Programme—A Cross-Sectional Multilevel Study. Cancers. 2021;13(17):4374; doi:10.3390/cancers13174374.

87. Rondet C, Soler M, Ringa V, Parizot I, Chauvin P. The role of a lack of social integration in never having undergone breast cancer screening: Results from a population-based, representative survey in the Paris metropolitan area in 2010. Preventive Medicine. 2013;57(4):386-91; doi:10.1016/j.ypmed.2013.06.016.

88. Ross JD, Ibecheozor C, Ezeofor A, Morales J, Williams C, Brim H, et al. Su1069 the influence of trust in source of health information and colorectal cancer screening uptake: analysis of a national survey in the United States. Gastrointestinal Endoscopy. 2020;91(6):AB290; doi:10.1016/j.gie.2020.03.1914.

89. Ross RL, Rubio K, Rodriguez HP. Mammography and decision aid use for breast cancer screening in older women. American Journal of Preventive Medicine. 2022;63(4):630-5; doi:10.1016/j.amepre.2022.04.014.

90. Sabgul AA, Qattan AM, Hashmi R, Al-Hanawi MK. Husbands’ knowledge of breast cancer and their wives’ attitudes and practices related to breast cancer screening in Saudi Arabia: Cross-sectional online survey. Journal of Medical Internet Research. 2021;23(2):e25404; doi:10.2196/25404.

91. Satoh M, Sato N. Relationship of attitudes toward uncertainty and preventive health behaviors with breast cancer screening participation. BMC Women's Health. 2021;21(1):1-11; doi:10.1186/s12905-021-01317-1.

92. Sheppard VB, Hurtado-de-Mendoza A, Song M, Hirpa F, Nwabukwu I. The role of knowledge, language, and insurance in endorsement of cancer screening in women of African origin. Preventive Medicine Reports. 2015;2:517-23; doi:10.1016/j.pmedr.2015.05.012.

93. Shin DW, Yu J, Cho J, Lee SK, Jung JH, Han K, et al. Breast cancer screening disparities between women with and without disabilities: A national database study in South Korea. Cancer. 2020;126(7):1522-9; doi:10.1002/cncr.32693.

94. SolikHaH S, Sangruangake M, Promthet S. Awareness of breast cancer and its screening among Indonesian women. Journal of Clinical Diagnostic Research. 2018;12(11):36-41; doi:10.7860/JCDR/2018/36943.12297.

95. Solikhah S, Promthet S, Hurst C. Awareness level about breast cancer risk factors, barriers, attitude and breast cancer screening among Indonesian women. Asian Pacific Journal of Cancer Prevention. 2019;20(3):877; doi:10.31557/apjcp.2019.20.3.877.

96. Son H, Kang Y. Breast cancer screening among shift workers: a nationwide population-based survey in Korea. International Journal of Occupational Environmental Health. 2017;23(2):94-7; doi:10.1080/10773525.2018.1425656.

97. Subramanian P, Oranye NO, Masri AM, Taib NA, Ahmad N. Breast cancer knowledge and screening behaviour among women with a positive family history: a cross sectional study. Asian Pacific Journal of Cancer Prevention. 2013;14(11):6783-90; doi:10.7314/APJCP.2013.14.11.6783.

98. Tapera R, Senabye PK, Mhaka-Mutepfa M, January J, Apau SG. The use of the Health Belief Model (HBM) in determining the factors associated with breast cancer screening among female students in Botswana. International Journal of Health Promotion Education. 2019;57(4):203-16; doi:10.1080/14635240.2019.1601026.

99. Tran ATN, Hwang JH, Choi E, Lee YY, Suh M, Lee CW, et al. Impact of awareness of breast density on perceived risk, worry, and intentions for future breast cancer screening among Korean women. Cancer Research

Treatment. 2021;53(1):55-64; doi:10.4143/CRT.2020.495.

100. Tsunematsu M, Kawasaki H, Masuoka Y, Kakehashi M. Factors affecting breast cancer screening behavior in Japan-assessment using the health belief model and conjoint analysis. Asian Pacific Journal of Cancer Prevention. 2013;14(10):6041-8; doi:10.7314/APJCP.2013.14.10.6041.

101. Vang S, Margolies LR, Jandorf L. Screening mammogram adherence in medically underserved women: Does language preference matter? Journal of Cancer Education. 2022;37(4):1076-82; doi:10.1007/s13187-020-01922-y.

102. Wang L, Mackenzie L, Hossain ZJN, Sciences H. Breast cancer screening practices and associated factors among Chinese‐Australian women living in Sydney: A cross‐sectional survey study. Nursing Health Sciences. 2022;24(1):293-303; doi:10.1111/nhs.12925.

103. Warner DF, Koroukian SM, Schiltz NK, Smyth KA, Cooper GS, Owusu C, et al. Complex multimorbidity and breast cancer screening among midlife and older women: The role of perceived need. The Gerontologist. 2019;59(Supplement_1):S77-S87; doi:10.1093/geront/gny180.

104. Wilson FA, Wang Y, Stimpson JP. The role of sick leave in increasing breast cancer screening among female employees in the US. Journal of Cancer Policy. 2014;2(3):89-92; doi:10.1016/j.jcpo.2014.07.003.

105. Yilmaz D, Bebis H, Ortabag T. Determining the awareness of and compliance with breast cancer screening among Turkish residential women. Asian Pacific Journal of Cancer Prevention. 2013;14(5):3281-8; doi:10.7314/APJCP.2013.14.5.3281.

106. Zhang LR, Chiarelli AM, Glendon G, Mirea L, Knight JA, Andrulis IL, et al. Worry is good for breast cancer screening: a study of female relatives from the ontario site of the breast cancer family registry. Journal of Cancer Epidemiology. 2012;2012; doi:10.1155/2012/545062.

107. Abdullah NN, Daud S, Al-Kubaisy W, Saari IS, Saad SR. Cervical cancer screening after 50: near extinction? European Journal of Obstetrics Gynecology Reproductive Biology. 2016;206:136-40; doi:10.1016/j.ejogrb.2016.09.011.

108. Abebaw E, Tesfa M, Gezimu W, Bekele F, Duguma A. Female healthcare providers’ knowledge, attitude, and practice towards cervical cancer screening and associated factors in public hospitals of Northwest Ethiopia. SAGE Open Medicine. 2022;10:20503121221095931; doi:10.1177/20503121221095931.

109. Abera GB, Abebe SM, Werku AG. Demand for Cervical Cancer Screening in Tigray Region of Ethiopia in 2018: A Community-Based Cross-Sectional Study. International Journal of Women's Health. 2020:795-804; doi:10.2147/IJWH.S255548.

110. Agboola AM, Bello OO. The determinants of knowledge of cervical cancer, attitude towards screening and practice of cervical cancer prevention amongst antenatal attendees in Ibadan, Southwest Nigeria. Ecancermedicalscience. 2021;15; doi:10.3332/ECANCER.2021.1225.

111. Agénor M, Krieger N, Austin SB, Haneuse S, Gottlieb BR. At the intersection of sexual orientation, race/ethnicity, and cervical cancer screening: assessing Pap test use disparities by sex of sexual partners among black, Latina, and white US women. Social Science Medicine. 2014;116:110-8; doi:10.1016/j.socscimed.2014.06.039.

112. Akinlotan M, Bolin JN, Helduser J, Ojinnaka C, Lichorad A, McClellan D. Cervical cancer screening barriers and risk factor knowledge among uninsured women. Journal of Community Health. 2017;42:770-8; doi:10.1007/s10900-017-0316-9.

113. Akinlotan M, Weston C, Bolin J. Individual-and county-level predictors of cervical cancer screening: a multi-level analysis. Public Health. 2018;160:116-24; doi:10.1016/j.puhe.2018.03.026.

114. Aktaş BA, Toptaş T, Üreyen I, Doğan S, Uysal A. Obstetrician-gynecologists’ practice patterns regarding HPV testing in cervical cancer screening in Turkey. Turkish Journal of Obstetrics and Gynecology. 2021;18(1):15; doi:10.4274/tjod.galenos.2021.36418.

115. Al-Amro SQ, Gharaibeh MK, Oweis AI. Factors associated with cervical cancer screening uptake: implications for the health of women in Jordan. Infectious Diseases in Obstetrics Gynecology. 2020;2020; doi:10.1155/2020/9690473.

116. Amin R, Kolahi A-A, Jahanmehr N, Abadi A-R, Sohrabi M-R. Disparities in cervical cancer screening participation in Iran: a cross-sectional analysis of the 2016 nationwide STEPS survey. BMC Public Health. 2020;20(1):1-8; doi:10.1186/s12889-020-09705-2.

117. Aminisani N, Armstrong BK, Canfell K. Cervical cancer screening in Middle Eastern and Asian migrants to Australia: a record linkage study. Cancer Epidemiology. 2012;36(6):e394-e400; doi:10.1016/j.canep.2012.08.009.

118. Aminisani N, Fattahpour R, Abedi L, Shamshirgaran S. Determinants of cervical cancer screening uptake in Kurdish women living in Western Iran, 2014. Asian Pacific Journal of Cancer Prevention. 2016;17(8):3763-7.

119. Ararsa T, Tadele N, Ayalew Y, Gela D. Knowledge towards cervical cancer screening and associated factors among urban health extension workers at Addis Ababa, Ethiopia: facility based cross-sectional survey. BMC Cancer. 2021;21:1-9; doi:10.1186/s12885-021-07952-z.

120. Aredo MA, Sendo EG, Deressa JT. Knowledge of cervical cancer screening and associated factors among women attending maternal health services at Aira Hospital, West Wollega, Ethiopia. SAGE Open Medicine. 2021;9:20503121211047063; doi:10.1177/20503121211047063.

121. Arulogun OS, Maxwell OO. Perception and utilization of cervical cancer screening services among female nurses in University College Hospital, Ibadan, Nigeria. Pan African Medical Journal. 2012;11(1).

122. Assefa AA, Astawesegn FH, Eshetu B. Cervical cancer screening service utilization and associated factors among HIV positive women attending adult ART clinic in public health facilities, Hawassa town, Ethiopia: a cross-sectional study. BMC Health Services Research. 2019;19:1-11; doi:10.1186/s12913-019-4718-5.

123. Atnafu T, Daka DW, Debela TF, Ergiba MS. Women’s Satisfaction with Cervical Cancer Screening Services and Associated Factors in Maternal Health Clinics of Jimma Town Public Health Facilities, Southwest Ethiopia. Journal of Medical Internet Research. 2021:7685-96; doi:10.2147/CMAR.S327369.

124. Ayanore MA, Adjuik M, Ameko A, Kugbey N, Asampong R, Mensah D, et al. Self-reported breast and cervical cancer screening practices among women in Ghana: predictive factors and reproductive health policy implications from the WHO study on global AGEing and adult health. BMC Women's Health. 2020;20:1-10; doi:10.1186/s12905-020-01022-5.

125. Aynalem BY, Anteneh KT, Enyew MM. Utilization of cervical cancer screening and associated factors among women in Debremarkos town, Amhara region, Northwest Ethiopia: Community based cross-sectional study. PloS One. 2020;15(4):e0231307; doi:10.1371/journal.pone.0231307.

126. Babazadeh T, Nadrian H, Rezakhani Moghaddam H, Ezzati E, Sarkhosh R, Aghemiri S. Cognitive determinants of cervical cancer screening behavior among housewife women in Iran: An application of Health Belief Model. Health Care for Women International. 2018;39(5):555-70; doi:10.1080/07399332.2018.1425873.

127. Badre-Esfahani S, Larsen MB, Seibæk L, Petersen LK, Blaakær J, Støvring H, et al. Non-adherence to childhood HPV vaccination is associated with non-participation in cervical Cancer screening–a Nationwide Danish register-based cohort study. Clinical Epidemiology. 2019:969-80; doi:10.2147/CLEP.S203023.

128. Badre-Esfahani S, Larsen M, Seibæk L, Petersen L, Blaakær J, Andersen B. Low attendance by non-native women to human papillomavirus vaccination and cervical cancer screening–A Danish nationwide register-based cohort study. Preventive Medicine Reports. 2020;19:101106; doi:10.1016/j.pmedr.2020.101106.

129. Bao H, Zhang L, Wang L, Zhang M, Zhao Z, Fang L, et al. Significant variations in the cervical cancer screening rate in China by individual‐level and geographical measures of socioeconomic status: a multilevel model analysis of a nationally representative survey dataset. Cancer Medicine. 2018;7(5):2089-100; doi:10.1002/cam4.1321.

130. Barrett-Harrison K, Priestley S. Determinants of cervical cancer screening among Jamaican women. West Indian Medical Journal. 2018;67(1):9-17; doi:10.7727/wimj.2017.001.

131. Baruch L, Bilitzky-Kopit A, Rosen K, Adler L. Cervical cancer screening among patients with physical disability. Journal of Women's Health. 2022;31(8):1173-8; doi:10.1089/jwh.2021.0447.

132. Bayu H, Berhe Y, Mulat A, Alemu A. Cervical cancer screening service uptake and associated factors among age eligible women in Mekelle Zone, Northern Ethiopia, 2015: a community based study using health belief model. PloS One. 2016;11(3):e0149908; doi:10.1371/journal.pone.0149908.

133. Belay Y, Dheresa M, Sema A, Desalew A, Assefa N. Cervical cancer screening utilization and associated factors among women aged 30 to 49 years in Dire Dawa, Eastern Ethiopia. Cancer Control. 2020;27(1):1073274820958701; doi:10.1177/1073274820958701.

134. Bermedo-Carrasco S, Pena-Sanchez JN, Lepnurm R, Szafron M, Waldner C. Inequities in cervical cancer screening among Colombian women: a multilevel analysis of a nationwide survey. Cancer Epidemiology. 2015;39(2):229-36; doi:10.1016/j.canep.2015.01.011.

135. Bianco A, Larosa E, Pileggi C, Nobile CG, Pavia M. Cervical and breast cancer screening participation and utilisation of maternal health services: a cross-sectional study among immigrant women in Southern Italy. BMJ Open. 2017;7(10); doi:10.1136/bmjopen-2017-016306.

136. Boni SP, Gnahatin F, Comoé J-C, Tchounga B, Ekouevi D, Horo A, et al. Barriers and facilitators in cervical cancer screening uptake in Abidjan, Côte d'Ivoire in 2018: a cross-sectional study. BMC Cancer. 2021;21:1-8; doi:10.1186/s12885-021-08650-6.

137. Bou-Orm I, Sakr R, Adib S. Cervical cancer screening among Lebanese women. Revue d'epidemiologie et de sante publique. 2018;66(1):1-6; doi:10.1016/j.respe.2017.10.004.

138. Brown H, Plourde N, Ouellette‐Kuntz H, Vigod S, Cobigo V. Brief report: cervical cancer screening in women with intellectual and developmental disabilities who have had a pregnancy. Journal of Intellectual Disability Research. 2016;60(1):22-7; doi:10.1111/jir.12225.

139. Brown RF, Muller TR, Olsen A. Australian women's cervical cancer screening attendance as a function of screening barriers and facilitators. Soc Sci Med. 2019;220:396-402; doi:10.1016/j.socscimed.2018.11.038.

140. Bruera S, Zogala R, Lei X, Pundole X, Zhao H, Giordano S, et al. FRI0516 FACTORS ASSOCIATED WITH DECREASED CERVICAL CANCER SCREENING IN WOMEN WITH SYSTEMIC LUPUS ERYTHEMATOSUS. Annals of the Rheumatic Diseases. 2020; doi:10.1136/annrheumdis-2020-eular.3824.

141. Brzoska P, Aksakal T, Yilmaz-Aslan Y. Utilization of cervical cancer screening among migrants and non-migrants in Germany: results from a large-scale population survey. BMC Public Health. 2020;20:1-9; doi:10.1186/s12889-019-8006-4.

142. Budkaew J, Chumworathayi B. Factors associated with decisions to attend cervical cancer screening among women aged 30-60 years in Chatapadung Contracting Medical Unit, Thailand. Asian Pacific Journal of Cancer Prevention. 2014;15(12):4903-7.

143. Bussière C, Le Vaillant M, Pelletier-Fleury N. Screening for cervical cancer: What are the determinants among adults with disabilities living in institutions? Findings from a National Survey in France. Health Policy. 2015;119(6):794-801; doi:10.1016/j.healthpol.2015.02.004.

144. Calys-Tagoe BN, Aheto JM, Mensah G, Biritwum RB, Yawson AE. Cervical cancer screening practices among women in Ghana: evidence from wave 2 of the WHO study on global AGEing and adult health. BMC Women's Health. 2020;20(1):1-9; doi:10.1186/s12905-020-00915-9.

145. Cerigo H, Coutlée F, Franco EL, Brassard P. Factors associated with cervical cancer screening uptake among Inuit women in Nunavik, Quebec, Canada. BMC Public Health. 2013;13:1-8; doi:10.1186/1471-2458-13-438.

146. Chan DN, So WK. Influential barriers perceived by South Asians in Hong Kong to undergoing cervical cancer screening. European Journal of Cancer Care. 2022;31(2):e13556; doi:10.1111/ecc.13556.

147. Chandrika K, Naik BN, Kanungo S. Awareness on cancer cervix, willingness, and barriers for screening of cancer cervix among women: A community-based cross-sectional study from urban Pondicherry. Indian Journal of Public Health. 2020;64(4):374-80; doi:10.4103/ijph.IJPH_29_20.

148. Chaowawanit W, Tangjitgamol S, Kantathavorn N, Phoolcharoen N, Kittisiam T, Khunnarong J, et al. Knowledge, attitudes and behavior of Bangkok metropolitan women regarding cervical cancer screening. Asian Pacific Journal of Cancer Prevention. 2016;17(3):945-52.

149. Chiou S-T, Wu C-Y, Hurng B-S, Lu T-H. Changes in the magnitude of social inequality in the uptake of cervical cancer screening in Taiwan, a country implementing a population-based organized screening program. International Journal for Equity in Health. 2014;13(1):1-8; doi:10.1186/1475-9276-13-4.

150. Chirwa GC. Explaining socioeconomic inequality in cervical cancer screening uptake in Malawi. BMC Public Health. 2022;22(1):1-14; doi:10.1186/s12889-022-13750-4.

151. Choi Y, Ibrahim S, Park LP, Cohen CR, Bukusi EA, Huchko MJ. Uptake and correlates of cervical cancer screening among women attending a community-based multi-disease health campaign in Kenya. BMC Women's Health. 2022;22(1):1-12; doi:10.1186/s12905-022-01702-4.

152. Cofie LE, Hirth JM, Wong R. Chronic comorbidities and cervical cancer screening and adherence among US-born and foreign-born women. Cancer Causes Control. 2018;29:1105-13; doi:10.1007/s10552-018-1084-2.

153. Compaore S, Ouedraogo CM, Koanda S, Haynatzki G, Chamberlain RM, Soliman AS. Barriers to cervical cancer screening in Burkina Faso: needs for patient and professional education. Journal of Cancer Education. 2016;31:760-6; doi:10.1007/s13187-015-0898-9.

154. Cunningham MS, Skrastins E, Fitzpatrick R, Jindal P, Oneko O, Yeates K, et al. Cervical cancer screening and HPV vaccine acceptability among rural and urban women in Kilimanjaro Region, Tanzania. BMJ Open. 2015;5(3); doi:10.1136/bmjopen-2014-005828.

155. Da Silva DC, Garnelo L, Herkrath FJ. Barriers to access the pap smear test for cervical cancer screening in rural riverside populations covered by a fluvial primary healthcare team in the amazon. International Journal of Environmental Research Public Health. 2022;19(7):4193; doi:10.3390/ijerph19074193.

156. Danan ER, Brunner J, Bergman A, Spoont M, Chanfreau C, Canelo I, et al. The relationship between sexual assault history and cervical cancer screening completion among women veterans in the Veterans Health Administration. Journal of Women's Health. 2022;31(7):1040-7; doi:10.1089/jwh.2021.0237.

157. De Prez V, Jolidon V, Willems B, Cullati S, Burton-Jeangros C, Bracke P. Cervical cancer screening programs and their context-dependent effect on inequalities in screening uptake: a dynamic interplay between public health policy and welfare state redistribution. International Journal for Equity in Health. 2021;20(1):1-14; doi:10.1186/s12939-021-01548-6.

158. De Vito C, Angeloni C, De Feo E, Marzuillo C, Lattanzi A, Ricciardi W, et al. A large cross-sectional survey investigating the knowledge of cervical cancer risk aetiology and the predictors of the adherence to cervical cancer screening related to mass media campaign. BioMed Research International. 2014;2014; doi:10.1155/2014/304602.

159. Deguara M, Calleja N, England K. Cervical cancer and screening: knowledge, awareness and attitudes of women in Malta. Journal of Preventive Medicine Hygiene. 2020;61(4):E584; doi:10.15167/2421-4248/jpmh2020.61.4.1521.

160. Desta AA, Endale ZM, Aklil MB. Cervical cancer screening utilization and associated factors among women of 30–65 years in Girar Jarsoo district North shoa, Ethiopia, 2021. Clinical Epidemiology Global Health. 2022;15:101048; doi:10.1016/j.cegh.2022.101048.

161. Destaw A, Midaksa M, Addissie A, Kantelhardt EJ, Gizaw M. Cervical cancer screening “see and treat approach”: real-life uptake after invitation and associated factors at health facilities in Gondar, Northwest Ethiopia. BMC Cancer. 2021;21:1-9; doi:10.1186/s12885-021-08761-0.

162. Do M. Predictors of cervical cancer screening among Vietnamese American women. Journal of Immigrant Minority Health. 2015;17:756-64; doi:10.1007/s10903-013-9925-2.

163. Drolet M, Boily M-C, Greenaway C, Deeks SL, Blanchette C, Laprise J-F, et al. Sociodemographic inequalities in sexual activity and cervical cancer screening: implications for the success of human papillomavirus vaccination. Cancer Epidemiology, Biomarkers Prevention. 2013;22(4):641-52; doi:10.1158/1055-9965.EPI-12-1173.

164. Dulla D, Daka D, Wakgari N. Knowledge about cervical cancer screening and its practice among female health care workers in southern Ethiopia: a cross-sectional study. International Journal of Women's Health. 2017:365-72; doi:10.2147/IJWH.S132202.

165. Ebu NI, Ogah JK. Predictors of cervical cancer screening intention of HIV-positive women in the central region of Ghana. BMC Women's Health. 2018;18(1):1-7; doi:10.1186/s12905-018-0534-z.

166. Ebu NI. Socio-demographic characteristics influencing cervical cancer screening intention of HIV-positive women in the central region of Ghana. Gynecologic Oncology Research Practice. 2018;5:1-7; doi:10.1186/s40661-018-0060-6.

167. El Mhamdi S, Bouanene I, Mhirsi A, Bouden W, Soltani MS. Cervical cancer screening: women's knowledge, attitudes, and practices in the region of Monastir (Tunisia). Revue d'épidémiologie et de santé publique. 2012;60(6):431-6; doi:10.1016/j.respe.2012.03.009.

168. Elit L, Krzyzanowska M, Saskin R, Barbera L, Razzaq A, Lofters A, et al. Sociodemographic factors associated with cervical cancer screening and follow-up of abnormal results. Can Fam Physician. 2012;58(1):e22-31; doi:10.1016/j.ygyno.2012.10.006.

169. Elit L, Saskin R, Raut R, Elliott L, Murphy J, Marrett L. Sociodemographic factors associated with cervical cancer screening coverage and follow-up of high grade abnormal results in a population-based cohort. Gynecol Oncol. 2013;128(1):95-100; doi:10.1016/j.ygyno.2012.10.006.

170. Emmanuel T, Oluwafolahan S, Sinat B. Predictors and factors related to the uptake of cervical cancer screening test among female secondary school teachers in Sagamu, Ogun State, Nigeria. Annals of Tropical Medicine Public Health. 2016;9(4); doi:10.4103/1755-6783.184788.

171. Enyan NIE, Davies AE, Opoku-Danso R, Annor F, Obiri-Yeboah D. Correlates of cervical cancer screening participation, intention and self-efficacy among Muslim women in southern Ghana. BMC Women's Health. 2022;22(1):1-11; doi:10.1186/s12905-022-01803-0.

172. Erku DA, Netere AK, Mersha AG, Abebe SA, Mekuria AB, Belachew SA. Comprehensive knowledge and uptake of cervical cancer screening is low among women living with HIV/AIDS in Northwest Ethiopia. Gynecologic Oncology Research Practice. 2017;4:1-7; doi:10.1186/s40661-017-0057-6.

173. Eshete M, Abdulwuhab Atta M, Yeshita HY. Cervical cancer screening acceptance among women in Dabat district, Northwest Ethiopia, 2017: an institution-based cross-sectional study. Obstetrics Gynecology International. 2020;2020; doi:10.1155/2020/2805936.

174. Faye A, Diagne N, Niang K, Dia A. Screening for cervical cancer in Senegal: Contributing factors. Annals of Tropical Medicine Public Health. 2017;10(6); doi:10.4103/ATMPH.ATMPH-354-17.

175. Fokom Domgue J, Cunningham SA, Yu RK, Shete S. Prevalence and determinants of cervical cancer screening with a combination of cytology and human papillomavirus testing. Annals of Epidemiology. 2019;36:40-7; doi:10.1016/j.annepidem.2019.06.002.

176. Ford S, Tarraf W, Williams KP, Roman LA, Leach R. Differences in cervical cancer screening and follow-up for black and white women in the United States. Gynecologic Oncology. 2021;160(2):369-74; doi:10.1016/j.ygyno.2020.11.027.

177. Galvin AM, Garg A, Matthes S, Thompson EL. Utilizing a multidimensional health literacy framework to assess cervical cancer screening nonadherence. Health Education Behavior. 2021;48(5):710-8; doi:10.1177/10901981211001851.

178. Gan DEH, Dahlui M. Cervical screening uptake and its predictors among rural women in Malaysia. Singapore Medical Journal. 2013;54(3):163-8; doi:10.11622/smedj.2013047.

179. Gatumo M, Gacheri S, Sayed A-R, Scheibe A. Women’s knowledge and attitudes related to cervical cancer and cervical cancer screening in Isiolo and Tharaka Nithi counties, Kenya: a cross-sectional study. BMC Cancer. 2018;18:1-9; doi:10.1186/s12885-018-4642-9.

180. Gebisa T, Bala ET, Deriba BS. Knowledge, attitude, and practice toward cervical cancer screening among women attending health facilities in Central Ethiopia. Cancer Control. 2022;29:10732748221076680; doi:10.1177/10732748221076680.

181. Gebreegziabher M, Asefa NG, Berhe S. Factors affecting the practices of cervical cancer screening among female nurses at public health institutions in Mekelle town, Northern Ethiopia, 2014: a cross-sectional study. Journal of Cancer Research. 2016;2016:1-7; doi:10.1155/2016/4743075.

182. Gemeda EY, Kare BB, Negera DG, Bona LG, Derese BD, Akale NB, et al. Prevalence and predictor of cervical cancer screening service uptake among women aged 25 years and above in Sidama Zone, Southern Ethiopia, Using Health Belief Model. Cancer Control. 2020;27(1):1073274820954460; doi:10.1177/1073274820954460.

183. Gerend MA, Shepherd MA, Kaltz EA, Davis WJ, Shepherd JE. Understanding women's hesitancy to undergo less frequent cervical cancer screening. Preventive Medicine. 2017;95:96-102; doi:10.1016/j.ypmed.2016.11.028.

184. Getachew S, Getachew E, Gizaw M, Ayele W, Addissie A, Kantelhardt EJ. Cervical cancer screening knowledge and barriers among women in Addis Ababa, Ethiopia. PloS One. 2019;14(5):e0216522; doi:10.1371/journal.pone.0216522.

185. Ghimire B, Pathak P. Determinants of uptake of cervical cancer screening among women attending tertiary level hospital. . J Nepal Health Res Coun. 2020; doi:10.33314/jnhrc.v18i4.2848.

186. González D, Suárez EL, Ortiz AP. Cervical cancer screening and sexual risky behaviors among a population of Hispanic origin. Women's Health Issues. 2015;25(3):254-61; doi:10.1016/j.whi.2015.01.002.

187. González A, Sánchez R, Camargo M, Soto-De León SC, Del Río-Ospina L, Mora LH, et al. Cervical cancer screening programme attendance and compliance predictors regarding Colombia’s Amazon region. PloS One. 2022;17(1):e0262069; doi:10.1371/journal.pone.0262069.

188. Gottschlich A, Nuntadusit T, Zarins KR, Hada M, Chooson N, Bilheem S, et al. Barriers to cervical cancer screening and acceptability of HPV self-testing: a cross-sectional comparison between ethnic groups in Southern Thailand. BMJ Open. 2019;9(11):e031957; doi:10.1136/bmjopen-2019-031957.

189. Grillo F, Vallée J, Chauvin P. Inequalities in cervical cancer screening for women with or without a regular consulting in primary care for gynaecological health, in Paris, France. Preventive Medicine. 2012;54(3-4):259-65; doi:10.1016/j.ypmed.2012.01.013.

190. Gu C, Chan CW, Twinn S, Choi KC. The influence of knowledge and perception of the risk of cervical cancer on screening behavior in mainland Chinese women. Psycho‐Oncology. 2012;21(12):1299-308; doi:10.1002/pon.2037.

191. Gyulai A, Nagy A, Pataki V, Tonté D, Ádány R, Vokó Z. Survey of participation in organised cervical cancer-screening programme in hungary. Central European Journal of Public Health. 2015;23(4):360-4; doi:10.21101/cejph.a4068.

192. Haas JS, Vogeli C, Yu L, Atlas SJ, Skinner CS, Harris KA, et al. Patient, provider, and clinic factors associated with the use of cervical cancer screening. Prev Med Rep. 2021;23:101468; doi:10.1016/j.pmedr.2021.101468.

193. Harder E, Thomsen LT, Hertzum-Larsen R, Albieri V, Hessner MV, Juul KE, et al. Determinants for participation in human papillomavirus self-sampling among nonattenders to cervical cancer screening in Denmark. Cancer Epidemiology, Biomarkers Prevention. 2018;27(11):1342-51; doi:10.1158/1055-9965.EPI-18-0480.

194. Harder E, Juul KE, Jensen SM, Thomsen LT, Frederiksen K, Kjaer SK. Factors associated with non-participation in cervical cancer screening–a nationwide study of nearly half a million women in Denmark. Preventive Medicine. 2018;111:94-100; doi:10.1016/j.ypmed.2020.106119.

195. Harder E, Hertzum-Larsen R, Frederiksen K, Kjær SK, Thomsen LT. Non-participation in cervical cancer screening according to health, lifestyle and sexual behavior: A Population-Based Study of nearly 15,000 Danish women aged 23–45 years. Preventive Medicine. 2020;137:106119; doi:10.1016/j.ypmed.2020.106119.

196. Harper DM, Tariq M, Alhawli A, Syed N, Patel MR, Resnicow K. Comparative predictors for cervical Cancer screening in southeast Michigan for middle Eastern-North african (MENA), White and african american/Black women. Preventive Medicine. 2022;159:107054; doi:10.1016/j.ypmed.2022.107054.

197. Holt HK, Zhang X, Hu S-Y, Zhao F-H, Smith JS, Qiao Y-L. Inequalities in cervical cancer screening uptake between Chinese migrant women and local women: a cross-sectional study. Cancer Control. 2021;28:1073274820985792; doi:10.1177/1073274820985792.

198. Idehen EE, Korhonen T, Castaneda A, Juntunen T, Kangasniemi M, Pietilä A-M, et al. Factors associated with cervical cancer screening participation among immigrants of Russian, Somali and Kurdish origin: a population-based study in Finland. BMC Women's Health. 2017;17:1-10; doi:10.1186/s12905-017-0375-1.

199. Idehen EE, Virtanen A, Lilja E, Tuomainen T-P, Korhonen T, Koponen P. Cervical Cancer Screening Participation among Women of Russian, Somali, and Kurdish Origin Compared with the General Finnish Population: A Register-Based Study. International Journal of Environmental Research Public Health. 2020;17(21):7899; doi:10.3390/ijerph17217899.

200. Idowu A, Olowookere SA, Fagbemi AT, Ogunlaja OA. Determinants of cervical cancer screening uptake among women in Ilorin, North Central Nigeria: a community-based study. Journal of Cancer Epidemiology. 2016;2016; doi:10.1155/2016/6469240.

201. Isabirye A, Mbonye MK, Kwagala BJPO. Predictors of cervical cancer screening uptake in two districts of Central Uganda. PLoS One. 2020;15(12):e0243281; doi:10.1371/journal.pone.0243281.

202. Islam RM, Bell RJ, Billah B, Hossain MB, Davis SR. Lack of understanding of cervical cancer and screening is the leading barrier to screening uptake in women at midlife in Bangladesh: population-based cross-sectional survey. The Oncologist. 2015;20(12):1386-92; doi:10.1634/theoncologist.2015-0235.

203. Issa T, Babi A, Azizan A, Alibekova R, Khan SA, Issanov A, et al. Factors associated with cervical cancer screening behaviour of women attending gynaecological clinics in Kazakhstan: A cross-sectional study. Women's Health. 2021;17:17455065211004135; doi:10.1177/17455065211004135.

204. Jabbari H, Piri R, Mohammadi S, Naghavi-Behzad M. Cervical Cancer Screening Behaviors Among Post-Menopausal Women. International Journal of Cancer Management. 2019;12(4); doi:10.5812/ijcm.80026.

205. Jang SH, Meischke H, Ko LK. The impact of medical tourism on cervical cancer screening among immigrant women in the US. BMC Women's Health. 2021;21(1):1-9; doi:10.1186/s12905-021-01558-0.

206. Johnson MJ, Mueller M, Eliason MJ, Stuart G, Nemeth LS. Quantitative and mixed analyses to identify factors that affect cervical cancer screening uptake among lesbian and bisexual women and transgender men. Journal of Clinical Nursing. 2016;25(23-24):3628-42; doi:10.1111/jocn.13414.

207. Judah G, Dilib F, Darzi A, Huf S. A population survey on beliefs around cervical cancer screening: determining the barriers and facilitators associated with attendance. BMC Cancer. 2022;22(1):522; doi:10.1186/s12885-022-09529-w.

208. Kaneko N. Factors associated with cervical cancer screening among young unmarried Japanese women: results from an internet-based survey. BMC Women's Health. 2018;18:1-9; doi:10.1186/s12905-018-0623-z.

209. Kasim J, Kalu A, Kamara B, Alema HB. Cervical cancer screening service utilization and associated factors among women in the Shabadino District, Southern Ethiopia. Journal of Cancer Epidemiology. 2020;2020; doi:10.1155/2020/6398394.

210. Kaso M, Takahashi Y, Nakayama T. Factors related to cervical cancer screening among women of childrearing age: a cross-sectional study of a nationally representative sample in Japan. International Journal of Clinical Oncology. 2019;24:313-22; doi:10.1007/s10147-018-1350-z.

211. Kasting ML, Wilson S, Zollinger TW, Dixon BE, Stupiansky NW, Zimet GD. Differences in cervical cancer screening knowledge, practices, and beliefs: An examination of survey responses. Preventive Medicine Reports. 2017;5:169-74; doi:10.1016/j.pmedr.2016.12.013.

212. Khanna D, Vashist S, Khanna A, Khanna AK. Determinants of Uptake of Cervical Cancer Screening in Northern India. Indian Journal of Public Health Research Development. 2019;10(8); doi:10.5958/0976-5506.2019.01946.6.

213. Kileo NM, Michael D, Neke NM, Moshiro C. Utilization of cervical cancer screening services and its associated factors among primary school teachers in Ilala Municipality, Dar es Salaam, Tanzania. BMC Health Services Research. 2015;15:1-9; doi:10.1186/s12913-015-1206-4.

214. Kouyoumdjian FG, McConnon A, Herrington ER, Fung K, Lofters A, Hwang SW. Cervical cancer screening access for women who experience imprisonment in Ontario, Canada. JAMA Network Open. 2018;1(8):e185637-e; doi:10.1001/jamanetworkopen.2018.5637.

215. Kue J, Hanegan H, Tan A. Perceptions of cervical cancer screening, screening behavior, and post-migration living difficulties among Bhutanese–Nepali refugee women in the United States. Journal of Community Health. 2017;42:1079-89; doi:10.1007/s10900-017-0355-2.

216. Lee EE, Eun Y, Lee S-Y, Nandy K. Age-related differences in health beliefs regarding cervical cancer screening among Korean American women. Journal of Transcultural Nursing. 2012;23(3):237-45; doi:10.1177/1043659612441015.

217. Lee M, Park E-C, Chang H-S, Kwon JA, Yoo KB, Kim TH. Socioeconomic disparity in cervical cancer screening among Korean women: 1998–2010. BMC Public Health. 2013;13:1-8; doi:10.1186/1471-2458-13-553.

218. Lee HY, Beltran R, Kim NK, Lee DK. Racial disparities in cervical cancer screening: Implications for relieving cervical cancer burden in Asian American Pacific Islander women. Cancer Nursing. 2019;42(6):458-67; doi:10.1097/NCC.0000000000000642.

219. Leinonen MK, Campbell S, Klungsøyr O, Lönnberg S, Hansen BT, Nygård M. Personal and provider level factors influence participation to cervical cancer screening: A retrospective register-based study of 1.3 million women in Norway. Preventive Medicine. 2017;94:31-9; doi:10.1016/j.ypmed.2016.11.018.

220. Lemma D, Aboma M, Girma T, Dechesa A. Determinants of utilization of cervical cancer screening among women in the age group of 30–49 years in Ambo Town, Central Ethiopia: a case-control study. PloS One. 2022;17(7):e0270821; doi:10.1371/journal.pone.0270821.

221. Liang LA, Zeissig SR, Schauberger G, Merzweiler S, Radde K, Fischbeck S, et al. Colposcopy non-attendance following an abnormal cervical cancer screening result: a prospective population-based cohort study. BMC Women's Health. 2022;22(1):1-13; doi:10.1186/s12905-022-01851-6.

222. Lin W, Chen B, Wu B, Yuan S, Zhong C, Huang W, et al. Cervical cancer screening rate and willingness among female migrants in Shenzhen, China: three-year changes in citywide surveys. Cancer Research Treatment: Official Journal of Korean Cancer Association. 2021;53(1):212-22; doi:10.4143/CRT.2020.219.

223. Lin S, Chen WT, Gu C, Cheng HL, Wang H, Tang S. Knowledge, perception of HIV symptom severity and cervical cancer screening behaviour among women living with HIV in China. European Journal of Cancer Care. 2022;31(2):e13542; doi:10.1111/ecc.13542.

224. Liu T, Li S, Ratcliffe J, Chen G. Assessing knowledge and attitudes towards cervical cancer screening among rural women in Eastern China. International Journal of Environmental Research Public Health. 2017;14(9):967; doi:10.3390/ijerph14090967.

225. Lyimo FS, Beran TN. Demographic, knowledge, attitudinal, and accessibility factors associated with uptake of cervical cancer screening among women in a rural district of Tanzania: three public policy implications. BMC Public Health. 2012;12(1):1-8; doi:10.1186/1471-2458-12-22.

226. Chisale Mabotja M, Levin J, Kawonga M. Beliefs and perceptions regarding cervical cancer and screening associated with Pap smear uptake in Johannesburg: A cross-sectional study. PloS One. 2021;16(2):e0246574; doi:10.1371/journal.pone.0246574

227. Maharjan M, Thapa N, Panthi D, Maharjan N, Petrini MA, Jiong Y. Health beliefs and practices regarding cervical cancer screening among women in Nepal: A descriptive cross‐sectional study. Nursing Health Sciences. 2020;22(4):1084-93; doi:10.1111/nhs.12775.

228. Marlow LA, Chorley AJ, Haddrell J, Ferrer R, Waller J. Understanding the heterogeneity of cervical cancer screening non-participants: data from a national sample of British women. European Journal of Cancer. 2017;80:30-8; doi:10.1016/j.ejca.2017.04.017.

229. Marques P, Geraldes M, Gama A, Heleno B, Dias S. Non-attendance in cervical cancer screening among migrant women in Portugal: A cross-sectional study. Women's Health. 2022;18:17455057221093034; doi:10.1177/17455057221093034.

230. Martín-López R, Hernández-Barrera V, de Andres AL, Carrasco-Garrido P, de Miguel AG, Jimenez-Garcia R. Trend in cervical cancer screening in Spain (2003–2009) and predictors of adherence. European Journal of Cancer Prevention. 2012;21(1):82-8; doi:10.1097/CEJ.0b013e32834a7e46.

231. Mboineki JF, Wang P, Dhakal K, Getu MA, Millanzi WC, Chen C. Predictors of uptake of cervical cancer screening among women in Urban Tanzania: community-based cross-sectional study. International Journal of Public Health. 2020;65:1593-602; doi:10.1007/s00038-020-01515-y.

232. McDaniel CC, Hallam HH, Cadwallader T, Lee H-Y, Chou C. Disparities in cervical cancer screening with HPV test among females with diabetes in the deep south. Cancers. 2021;13(24):6319; doi:10.3390/cancers13246319.
[truncated: 108,196 more chars]
